# AgBF_4_‐Induced Site‐Selective Synthesis of 4‐Sulfonylindoles in Au‐Catalyzed Cyclization‐Sulfonyl Migration Reactions

**DOI:** 10.1002/chem.202503038

**Published:** 2026-01-19

**Authors:** Itaru Nakamura, Chunbo Jia, Masahiro Terada

**Affiliations:** ^1^ Institute For Excellence in Higher Education Tohoku University Sendai Japan; ^2^ Department of Chemistry Graduate School of Science Tohoku University, Sendai Aoba‐ku Japan

**Keywords:** cyclization, gold, indole, rearrangement, regioselectivity

## Abstract

The reactions of *ortho*‐alkynyl‐*N*‐arenesulfonylanilines in the presence of SPhosAuCl (2 mol %) and AgBF_4_ (6 mol %) produced the corresponding 4‐sulfonylindoles in good yields with satisfactory site‐selectivity at the C4‐position of the indole ring. This reaction proceeded via cyclization followed by sequential [1,2]‐rearrangement of the sulfonyl group. The selective migration of the sulfonyl group to the C4‐position was induced by using an excess amount of AgBF_4_ relative to the gold catalyst to form a dinuclear Au─Ag complex to decelerate the migration to C3‐position. Furthermore, the tetrafluoroborate anion with its suitable gold affinity and hydrogen bonding characteristics, plays a crucial role in enabling selective migration to the C4‐position.

## Graphical abstract

Site‐selective synthesis 4‐suflonylindoles was achieved by a cyclization‐rearrangement strategy by π‐Lewis acidic gold catalysts and the aid of silver cocatalyst. Particularly, the use of 3 equivalents of AgBF_4_ to SPhosAuCl is crucial for C4‐selective migration of the sulfonyl group.

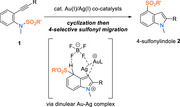

## 1 Introduction

**SCHEME 1:**
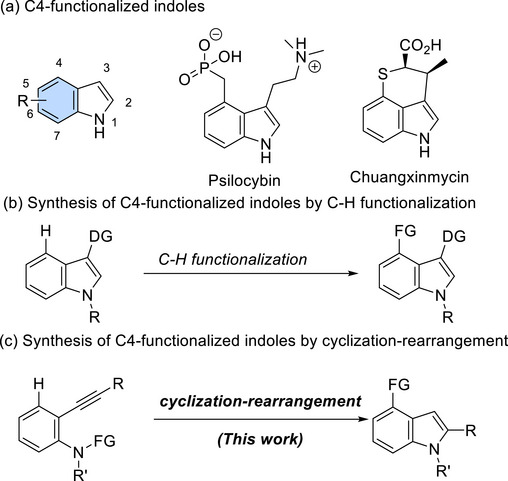
Benzene core‐functionalized indoles.

Functionalized indoles are one of the most important motifs in pharmaceutical science (Scheme 1) [1, 2, 3]. Substitutions in the five‐membered ring are a typical way to functionalize indoles because of the electron‐rich nature of the five‐membered ring. In contrast, site‐selective substitution at the C4‐, C5‐, C6‐, and C7‐positions remain unexplored owing to the much lower reactivity of the benzene core than the five‐membered ring [4]. In general, stepwise protocols have been adopted to construct a five‐membered ring from the anilines, of which the benzene ring is prefunctionalized [5]. Specifically, C4‐functionalized indoles, which are often found in bioactive compounds, such as Psilcybin, and Chuangxinmycin (Scheme 1a), have been synthesized based on the catalytic C─H functionalization, mediated by directing groups at the C3 ‐position (Scheme 1b). [6, 7, 8, 9, 10] However, designing effective methods to synthesize indoles with various functional groups on the benzene ring is still challenging [11].

**SCHEME 2:**
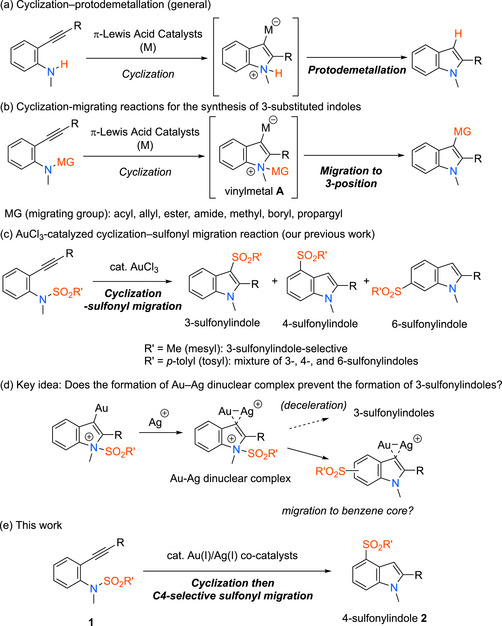
π‐Lewis acidic metal‐catalyzed cyclization reactions for the synthesis of indoles.

To resolve this problem, we focused on a migration strategy using π‐Lewis acidic metal catalysts (Scheme 1c and 2). Cyclization reactions of *ortho*‐alkynylanilines are frequently utilized to synthesize indoles owing to the high compatibility of the functional groups under the mild reaction conditions of π‐Lewis acidic metal catalysis (Scheme 2a) [12, 13, 14, 15]. *ortho*‐Alkynylanilines, which have a migrating group, such as acyl [16, 17, 18]. allyl [19, 20], propargyl [21, 22], alkoxycarbonyl [23], carbamoyl [23], methyl [24], or boryl group [25, 26], on the nitrogen atom, undergo cascade reactions involving cyclization followed by rearrangement of the migrating group to the C3‐position of the indole ring of resulting cyclized vinylmetal intermediate **A** (Scheme 2b). In particular, we developed gold‐catalyzed reactions of *ortho*‐alkynyl‐*N*‐sulfonylanilides that yielded the corresponding 3‐sulfonylindoles through cyclization followed by rearrangement of the sulfonyl group (Scheme 2c) [19, 20, 21, 22, 23, 24, 25, 26, 27, 28, 29, 30, 31, 32]. Our findings that the reaction of *N‐*tosylanilines afforded a mixture of 3‐, 4‐, and 6‐sulfonylindoles, whereas that of *N*‐mesylanilines selectively afforded 3‐sulfonylindoles, encouraged us to explore the site‐selective synthesis of indoles having a sulfonyl group at the benzene core by establishing the appropriate reaction conditions for the gold‐catalyzed cyclization–migration strategy. Specifically, we expected that using a silver cocatalyst would decelerate the unfavorable migration of the sulfonyl group to the most nucleophilic C3‐position by forming a dinuclear Au─Ag complex [33]. The so‐called “silver effect” [34, 35, 36, 37, 38, 39] would lead the sulfonyl group to the benzene core of the indole ring (Scheme 2d). Herein, we report that the reactions of *N*‐sulfonyl*‐ortho*‐alkynylanilines **1** in the presence of Au and Ag cocatalysts afforded 4‐sulfonylindoles **2** in good yields with high site selectivity (Scheme 2e).

## 2 Results and Discussion

**SCHEME 3:**
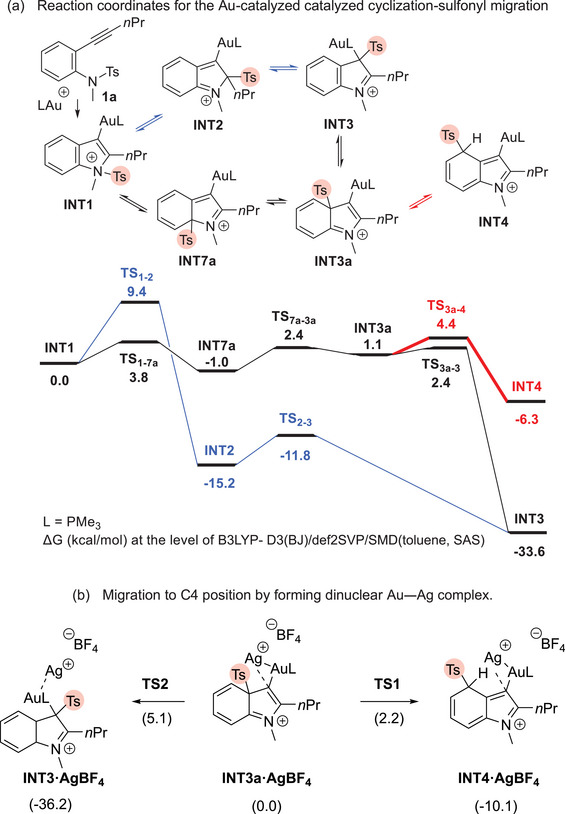
Au‐catalyzed reaction coordinates.

Prior to experimental trials, we calculated the migration process of the sulfonyl group of cyclized intermediate **INT1**, derived from the PMe_3_Au^+^‐catalyzed cyclization of *N*‐tosylaniline **1a**, to the carbon atoms on the indole ring at the level of B3LYP‐D3(BJ)/def2SVP/SMD(toluene, SAS) (Scheme 3a). Contrary to our previously proposed intermediacy of ion pair species, the calculations suggest that the sulfonyl group migrates through sequential [1, 2]‐rearrangement, akin to what is described as “sulfonyl walking” (see ESI) by Wang and Huang [40]. To be precise, the sulfonyl group migrates to the C3‐position of the indole ring through two pathways: (a) N1 “C2 (**INT2**)” C3 (**INT3**) and (b) N1 “C7a (**INT7a**)” C3a (**INT3a**) “C3 (**INT3**)”. More importantly, the calculations suggest that the energy barrier for the migration from C3a (**INT3a**) to C4 (**INT4**) is sufficiently low to enable moving to the benzene core from the C3a‐position, although **TS_3a‐4_** is slightly higher than **TS_3a‐3_**. Thus, we postulated that indoles having a sulfonyl group on the benzene core could be generated as kinetic products by decelerating the migration of the sulfonyl group from C3a to C3 through the silver effect (Scheme 2d). Our preliminary calculations indicate that the formation of dinuclear Au─Ag complex from the cyclized intermediate **INT1** is thermodynamically favorable (Scheme S3). Moreover, our DFT calculations suggest that, upon formation of the dinuclear Au─Ag complex, the activation energy for migration of tosyl group from C3a position to C4 position becomes lower than that for migration to the C3 position (Scheme 3b).

Based on these computational results, we surveyed the reaction conditions using *N*‐tosylaniline **1a** as a substrate. When the reaction of *N*‐tosylaniline **1a** was carried out in the presence of 2 mol % of SPhosAuCl (SPhos: 2‐dicyclohexylphosphino‐2‘,6’‐dimethoxybiphenyl) and 4 mol % of AgBF_4_ in toluene at 0°C, corresponding 4‐tosylindole **2a** [40] was obtained as a major product (65%) along with the formation of 3‐tosylindole **3a** (16%) 6‐tosylindole **4a** (7%), and 7‐tosylindole **5a** (trace) as byproducts (Table 1, entry 2). As we hypothesized, increasing the loading amount of AgBF_4_ improved the product selectivity of **2a** (entry 3), although using fivefold AgBF_4_ than SPhosAuCl decelerated the reaction (entry 4). When an equal amount of AgBF_4_ to the gold complex was used, the product ratio of the three isomers **2a, 3a**, and **4a**, was unreproducible (entry 1), indicating that the product selectivity was very sensitive to the number of silver ions that were not precipitated as AgCl. Notably, the product ratio was significantly affected by the counteranion. The reaction of **1a** using AgSbF_6_, AgNTf_2_, and Ag(tfa), instead of AgBF_4_, diminished the product selectivity of 4‐sulfonylindole **2a**, and 3‐sulfonylindole **3a** was obtained as a major product (entries 5–7). In contrast, the reaction using AgOTs hardly proceeded (entry 8). Solvents had a strong influence on the product ratio; the use of less coordinative solvents, such as chlorobenzene, resulted in the highest selectivity to yield **2a** (entry 9, standard conditions), whereas the use of more coordinative solvents, such as acetonitrile, yielded **3a** as a major product (entry 10). It should be noted that the addition of tetrabutylammonium tetrafluoroborate (TBABF_4_) to the reaction using SPhosAuCl and AgSbF_6_ resulted in an improved product selectivity to yield **2a**, suggesting that the tetrafluoroborate anion plays a crucial role in the C4 selectivity (entry 11 vs. entry 5). In contrast, the reaction under the Ag‐free conditions using SPhosAuBF_4_ resulted in diminishing C4‐selectivity (entry 12), suggesting that the silver atom is indispensable for the regioselectivity (entry 12). On the other hand, the reaction in the absence of SPhosAuCl resulted in a decrease in the chemical yields of the sulfonylindoles, although the major product was the 4‐sulfonylindole **2a**, suggesting that the gold complex is essential to promote the transformation efficiently (entry 13). The ligand on the gold complex also affected product selectivity. The gold complex having a less electron‐donating phosphine ligand, such as P(C_6_F_5_)_3_ and P(OPh)_3_, afforded 3‐tosylindole **3a** as a major product (entries 15 and 16), whereas PCy_3_AuCl yielded **2a** preferentially (entry 17). Recently, Arisawa reported that the Au‐catalyzed cyclization reactions of *ortho*‐allenyl‐*N*‐tosylanilines gave 4‐sulfonylindoles [41]. However, the reaction of *ortho*‐alkynyl‐*N*‐tosylaniline **1a** under the conditions reported by Arisawa yielded 3‐tosylindole **3a** as a major product (entry 19).

**TABLE 1:** Optimization of reaction conditions.

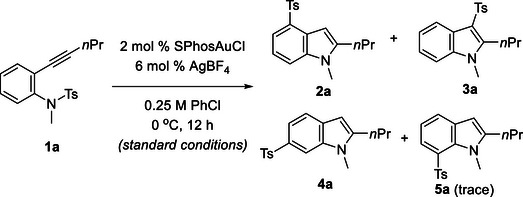				
Entry	Variation from the standard conditions	**2a** (%)^a^	**3a** (%)^a^	**4a** (%)^a^
1^b^	AgBF_4_ (2 mol %) in toluene	46	30	12
2^b^	AgBF_4_ (4 mol %) in toluene	65	16	7
3	In toluene	70	10	5
4	AgBF_4_ (10 mol %) in toluene	51	6	4
5	AgSbF_6_ (2 mol %) instead of AgBF_4_ in toluene	23	46	20
6	AgNTf_2_ (2 mol %), instead of AgBF_4_ in toluene	17	56	21
7	Ag(tfa) (2 mol %), instead of AgBF_4_ in toluene	20	46	20
8	AgOTs (2 mol %), instead of AgBF_4_ in toluene	3	8	3
9	none	76	10	5
10	In MeCN	25	46	25
11	AgSbF_6_ instead of AgBF_4_, with TBABF_4_	64	21	11
12	SPhosAuBF_4_, instead of SPhosAuCl, without AgBF_4_	21	45	30
13	Without SPhosAuCl	17	4	3
14	PPh_3_AuCl, instead of SPhosAuCl	56	33	6
15	P(C_6_F_5_)_3_AuCl, instead of SPhosAuCl	33	44	6
16	P(OPh)_3_AuCl, instead of SPhosAuCl	31	52	6
17	PCy_3_AuCl, instead of SPhosAuCl	66	22	6
18	XPhosAuCl, instead of SPhosAuCl	44	36	13
19	Conditions reported by Arisawa^c^	20	51	18

^a^ Yields were determined by ^1^H NMR spectroscopy using CH_2_Br_2_ as the internal standard.

^b^ 6 h.

^c^ (Me_2_S)AuCl (10 mol %), PCy_3_ (10 mol%), AgOTf (10 mol %), CH_2_Cl_2_, 0°C, 12 h.

Next, the substituent on the migrating sulfonyl group was examined to clarify the tendency of the product selectivity under the optimal conditions for the site‐selective synthesis of 4‐sulfonylindole **2a** (Table 1, entry 9). Substrates having various functional groups, such as *p*‐Me (**1a**, Table 1, entry 9), *p*‐F (**1e**, Table 2, entry 4), *p*‐Cl (**1f**, entry 5), *p*‐CF_3_ (**1** **g**, entry 6), *p*‐NO_2_ (**1** **h**, entry 7), and *m*‐Me (**1c** entry 2), were preferentially converted into 4‐sulfonylindoles **2** under the reaction conditions. Regarding the electronic effects, a highly electron‐rich *p*‐anisyl group led to a decrease in the C4 selectivity, whereas the highly electron‐deficient *p*‐nitrophenyl group did not significantly affect product selectivity (entry 1 vs. entry 7). Additionally, a bulky *ortho*‐tolyl group (**1d**, entry 3) on the sulfur atom also reduced the C4 selectivity. The reaction of *N*‐mesylaniline **1i** yielded 4‐mesylindole **2i** as a major product (entry 8), although the product selectivity was much lower than *N*‐tosylindole.

**TABLE 2:** Substituent effect of the sulfonyl group.

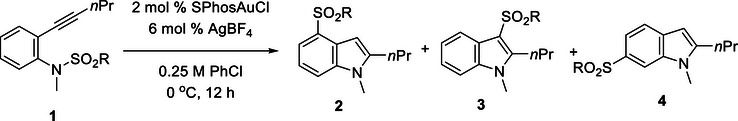					
Entry	**1**	R	**2** (%)^a^	**3** (%)^a^	**4** (%)^a^
1	**1b**	*p*‐MeOC_6_H_4_	**2b** (40)	**3b** (29)	**4b** (13)
2	**1c**	*m*‐MeC_6_H_4_	**2c** (69)	**3c** (16)	**4c** (7)
3	**1d**	*o*‐MeC_6_H_4_	**2d** (44)	**3d** (29)	**4d** (13)
4	**1e**	*p*‐FC_6_H_4_	**2e** (80)	**3e** (12)	**4e** (6)
5	**1f**	*p*‐ClC_6_H_4_	**2f** (74)	**3f** (13)	**4f** (6)
6	**1g**	*p*‐F_3_CC_6_H_4_	**2 g** (68)	**3 g** (21)	**4 g** (10)
7	**1h**	*p*‐O_2_NC_6_H_4_	**2 h** (64)	**3 h** (16)	**4 h** (9)
8	**1i**	Me	**2i** (38)	**3i** (19)	**4i** (22)

^a^ Yields were determined by ^1^H NMR spectroscopy using CH_2_Br_2_ as the internal standard.

The bulkiness of the substituent at the alkyne terminus also affected the product selectivity. Substrates **1j** and **1k,** which have a *n*‐hexyl and cyclohexyl group, respectively, yielded **2j** and **2k** with good C4 selectivity (Table 3, entries 1 and 2). In contrast, the reactions of substrates **1l** and **1m**, which have a smaller cyclopropyl group and a methyl group, respectively, decreased C4 selectivity (entries 3 and 4). The reaction of **1n**, which has a phenyl group at the alkyne terminus, yielded 4‐sulfonylindole **2n** as a major product, although the considerable amount of 3‐sulfonylindole (**3n**) was obtained as a byproduct (entry 5). The reactions of substrates **1o** and **1p**, which have a trimethylsilyl alkyne and a terminal alkyne, respectively, did not yield corresponding desired products **2o** and **2p** (entries 6 and 7). A benzyl group (**1q**) was tolerated by increasing loading amounts of both Au complex and AgBF_4_ to the afford corresponding 4‐sulfonylindole **2q** as a major product (entry 8).

**TABLE 3:** Substrate scope.

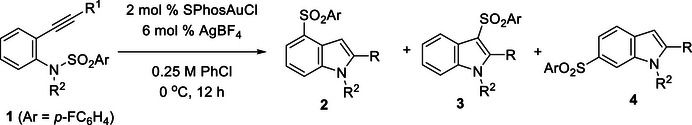						
Entry	**1**	R^1^	R^2^	**2** (%)^a^	**3** (%)^a^	**4** (%)^a^
1	**1j**	*n*Hex	Me	**2j** (76)	**3j** (15)	**4j** (7)
2	**1k**	Cy	Me	**2k** (77)	**3k** (10)	**4k** (6)
3	**1l**	Cyclopropyl	Me	**2l** (42)	**3l** (26)	**4l** (14)
4	**1m**	Me	Me	**2m** (53)	**3m** (16)	**4m** (9)
5	**1n**	Ph	Me	**2n** (47)	**3n** (27)	**4n** (12)
6	**1o**	SiMe_3_	Me	<1^b^	<1	<1
7	**1p**	H	Me	<1^c^	<1	<1
8^d^	**1q**	*n*Pr	Bn	**2q** (71)	**3q** (14)	**4q** (9)

^a^ Yields were determined by ^1^H NMR spectroscopy using CH_2_Br_2_ as the internal standard.

^b^ 92% of **1o** was recovered.

^c^ 8% of **1p** was recovered.

^d^ 5 mol % of SPhosAuCl and 15 mol % of AgBF_4_ were employed.

**SCHEME 4:**
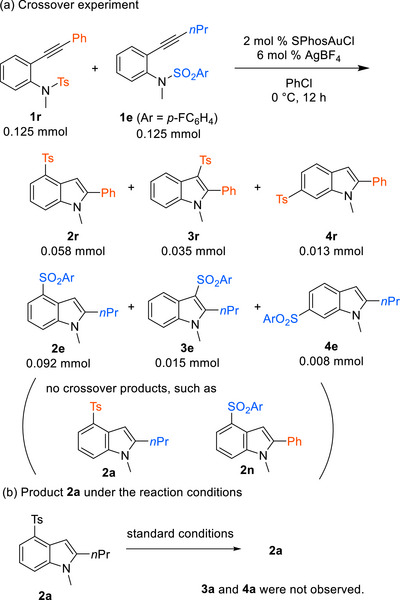
Mechanistic studies.

The reaction of a mixture of equally reactive substrates **1r** and **1e** under standard reaction conditions afforded only two sets of products (**2r, 3r**, and **4r**) and (**2e**, **3e**, and **4e**) derived from the starting material. No crossover products, such as **2a** and **2n**, were detected by GCMS (Scheme 4a). The results suggest that sulfonyl group migration proceeds in an intramolecular manner. In addition, when isolated 4‐sulfonylindole **2a** was treated under the standard reaction conditions, 3‐sulfonylindole (**3a**) and 6‐sulfonylindole (**4a**) were not produced, suggesting that the interconversion among sulfonylindoles **2**, **3**, and **4** does not occur in the present transformation (Scheme 4b).

**SCHEME 5:**
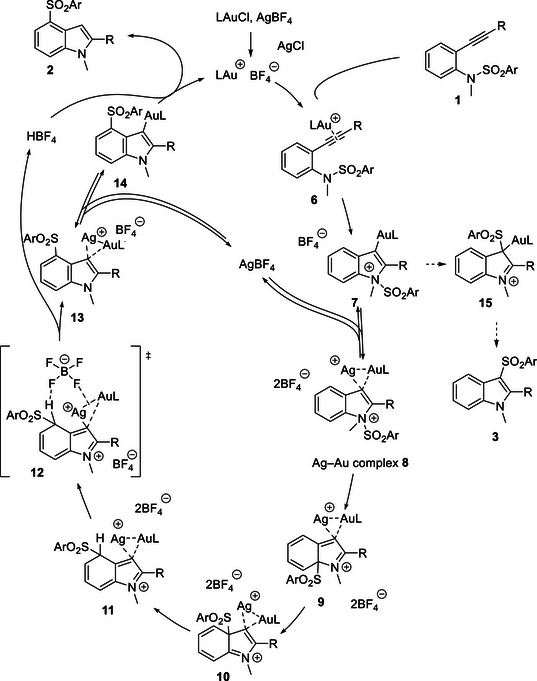
Proposed mechanism.

We propose a reaction mechanism for the selective synthesis of 4‐sulfonylindole **2** as illustrated in Scheme 5. First, the cationic gold catalyst, generated by the reaction between the gold chloride complex and AgBF_4_, coordinates to the alkyne moiety of substrate **1** to form π‐complex **6**. Then, the nucleophilic attack of the aniline nitrogen atom on the electrophilically activated carbon–carbon triple bond resulted in the formation of cyclized vinylgold species **7**. This intermediate **7** reacts with excess AgBF_4_ to form dinuclear Au─Ag complex **8**. Then, the sulfonyl group migrates to the C4‐position of the indole ring through successive [1, 2]‐rearrangement, namely, sulfonyl walking via **9** and **10**, to 4‐sulfonylated intermediate **11**. Deprotonation takes place through the interaction with tetrafluoroborate anion via transition state **12**. Eliminating AgBF_4_ in dinuclear Au─Ag complex **13** yields vinylgold intermediate **14**. Finally, the protodeauration yields 4‐sulfonylindole **2**. The key to the selective synthesis of 4‐sulfonylindole **2** is using AgBF_4_ in an excess amount relative to the gold complex SPhosAuCl. The formation of dinculear Au─Ag complex **8** decelerates the migration of the sulfonyl group to the most nucleophilic C3‐position of the indole ring to form **15**, suppressing the formation of 3‐sulfonylindole **3**. It is known that there is an equilibrium between vinylgold species and dinulear Au─Ag complex [37]. Thus, it is necessary to use excess amounts of silver salt, shifting the equilibrium to the dinuclear Au─Ag complex side to suppress unfavorable migration to C3‐position. More importantly, tetrafluoroborate is crucial for the selective formation of 4‐sulfonylindole **2**. We consider that the tetrafluoroborate anion functions as a Brønsted base to facilitate the deprotonation. Indeed, the hydrogen bond basicity index (HBI) [42] of BF_4_^−^ (5.2) is much higher than that of SbF_6_^−^ (2.8) or Tf_2_N^−^ (1.0), supporting our hypothesis for the acceleration of the proton elimination by the tetrafluoroborate anion as shown in transition state **12**. In addition, electrostatic interaction between the tetrafluoroborate anion and the cationic Au─Ag binuclear moiety at the C3‐position of the indole ring would facilitate the deprotonation at the C4‐position. It is also essential that the gold affinity index (GAI) of the tetrafluoroborate anion (0.5) is as high as that of SbF_6_^−^, to maintain the catalytic activity of the gold catalyst system, regardless of the high Brønsted basic nature of BF_4_^−^ [43]. The superiority of the tetrafluoroborate anion can be explained by the fact that the reaction using a tosylate anion, of which HBI and GAI are 4.3 and 3.8, respectively, instead of a tetrafluoroborate anion (HBI: 5.2, GAI: 0.5), is inert (Table 1, entry 8). It should be noted that the formation of 6‐sulfonylindoles **4** and 7‐sulfonylindoles **5** through sulfonyl walking on the benzene ring was supported by DFT calculations (Scheme S2). Further improvements in product selectivity by designing counteranions are being pursued in our laboratory. Furthermore, given the wide range of known transformations of aryl sulfones [44], this strategy provides a valuable platform for accessing C4‐functionalized indoles. Our present study on migratory cyclization proves the uniqueness of the migration strategy for synthesizing indoles functionalized at the benzene core [45].

## 3 Conclusion

We have developed an entirely new approach for synthesizing 4‐sulfonylindoles in a site‐selective manner. Our investigations revealed that the Au─Ag cocatalysts play a crucial role for the selective synthesis via the formation of dinuclear Au─Ag complex to decelerate migration of a sulfonyl group at the C3‐position. Remarkably, this Au‐catalyzed cascade reaction exhibits high compatibility of functional groups owing to the mild reaction conditions of gold catalysis. Thus, migratory cyclization has the potential to synthesize heteroarenes having functional groups at the less reactive benzene core in a site‐selective manner.

## 4 Experimental Section

General procedure for the Au‐catalyzed reactions of **1**. To an oven‐dried 2.5 mL glass reaction vessel, substrate **1** (0.25 mmol) and a magnetic stirrer bar were added under Ar atmosphere. Then, 0.5 mL of chlorobenzene was added to the vessel, and the vessel was put into a 0°C cooling bath with magnetic stirrer for 15 min. SPhosAuCl (3.2 mg, 0.005 mmol) and AgBF_4_ (2.9 mg, 0.0015 mmol) were dissolved and mixed in another 0.5 mL solvent to form a uniform suspension. Then, the catalyst mixture was injected into the vessel to start the reaction. After designated reaction time, the reaction was quenched using 1 mL of 1N Na_2_S_2_O_3_ solution, extracted with EtOAc, dried over Na_2_SO_4_ and passed through a short silica gel pad to remove catalyst. Yield was determined by ^1^H NMR using CH_2_Br_2_ as an internal standard. Purification was conducted using medium‐pressure liquid chromatography using silica gel as a stationary phase and hexane and EtOAc as eluent. Further purification, if necessary, was conducted gel permeation chromatography using chloroform as the solvent.

## Conflicts of Interest

The authors declare no conflict of interest.

## Supporting information



The authors have cited additional references within the Supporting Information [46, 47, 48]. **Supporting File 1**: chem70696‐sup‐0001‐SuppMat.pdf
