# Supplementary material for: AgBF4‐Induced Site‐Selective Synthesis of 4‐Sulfonylindoles in Au‐Catalyzed Cyclization‐Sulfonyl Migration Reactions
Source: Chemistry. 2026 Jan 19;32(12):e03038. doi: 10.1002/chem.202503038 (PMC13037354; doi:10.1002/chem.202503038)
Supplement: Supplementary file 1 — The authors have cited additional references within the Supporting Information [46, 47, 48]. Supporting File 1: chem70696‐sup‐0001‐SuppMat.pdf [file CHEM-32-e03038-s001.pdf]

$AgBF_4$-Induced Site-Selective Synthesis of 4-Sulfonylindoles in Au-catalyzed Cyclization-Sulfonyl Migration Reactions

Itaru Nakamura,* Chunbo Jia, and Masahiro Terada

Supporting Information

1. **General Information** **S2**
2. **Preparation of catalysts and substrates** **S3**
3. **General procedure** **S5**
4. **Optimization of reaction conditions** **S6**
5. **1 mmol-scale experiments** **S8**
6. **Mechanistic studies** **S9**
7. **Computational analysis** **S14**
8. **Crystallographic data of 2a** **S64**
9. **Analytical data of substrates and products** **S66**
10. **NMR charts of $SPhosAuBF_4$** **S97**
11. **NMR charts of substrates and products** **S98**
12. **Determination of ^{1}H NMR yield** **S166**

1. General information

^{1}H, ^{13}C, ^{31}P and ^{19}F NMR spectra were recorded on JEOL JNM-ECS400 (400 MHz for ^{1}H and 100 MHz for ^{13}C and 376 MHz for ^{19}F) spectrometer or JEOL ECA-600 (600 MHz for ^{1}H, 150 MHz for ^{13}C, 565MHz for ^{19}F and 243MHz for ^{31}P) spectrometer. Chemical shifts are reported in ppm relative to TMS (for ^{1}H, δ 0.0), $CDCl_3$ (for ^{13}C, δ 77.0) and $C_6H_5CF_3$ (for ^{19}F, δ -63.72), respectively. ^{1}H NMR data are reported as follows: chemical shift, integration, multiplicity (s = singlet, d = doublet, t = triplet, q = quartet, sext = sextet, br = broad, m = multiplet) and coupling constants (Hz). Infrared (IR) spectra were recorded on a JASCO FT/IR- 4100 spectrometer with ATR Pro-410 S attachment and ZnSe prism. GC-MS measurements were carried out on Agilent 8860 gas chromatograph system with Agilent 5977C mass selective detector. High-resolution mass spectra analysis was performed on a Bruker Daltonics APEX III FT-ICR-MS spectrometer and Bruker Daltonics solariX FT-ICR-MS spectrometer at Research and Analytical Center for Giant Molecules, Graduate School of Science, Tohoku University. Flash column chromatography was performed on silica gel 60N (Merck 40-63 µm or Kanto 40-50 µm) manually or use YAMAZEN Smart Flash EPCLC AI-580S Automated Flash Chromatography System. JAI LaboACE LC-5060 Plus recycling preparative HPLC system equipped with two JAIGEL 2HR-Plus GPC columns was used for purification. Analytical thin layer chromatography (TLC) was performed on Merck pre-coated TLC plates (silica gel 60 F254).

Materials

Anhydrous chlorobenzene was purchased from Sigma-Aldrich. Anhydrous toluene and other solvents were purchased from WAKO. SPhos and Cy_3P were purchased from BLD Pharm inc. $CDCl_3$ was purchased from Sigma Aldrich. These reagents were used as received.

Alkynes were purchased from TCI or WAKO except cyclohexylacetylene, which was prepared from cyclohexanecarboxaldehyde using literature reported procedure.[1]

Unless specified, all air- and moisture-sensitive manipulations were performed under argon atmosphere using oven-dried glassware, including glovebox techniques.

2. Preparation of catalysts and substrates

Unless specifically mentioned, all gold (I) catalyst was prepared according to literature reported procedure.[2, 3]

2.1 Preparation of SPhosAuBF4

Under argon atmosphere, $AgBF_4$ (21.0 mg, 0.108 mmol) was added to a solution of SPhosAuCl (69.4 mg, 0.108 mmol) in CH_2Cl_2 (5 mL) and stirred at room temperature for 20 minutes. The obtained mixture was passed through a short celite pad and concentrated under reduced pressure. $SPhosAuBF_4$ was obtained quantitively as light-yellow solid.

^{1}H-NMR (400 MHz, CHLOROFORM-D) δ 7.55 (dt, J = 30.5, 7.7 Hz, 4H), 7.21 (q, J = 3.7 Hz, 1H), 6.71 (q, J = 8.7 Hz, 2H), 3.70 (s, 6H), 2.17-2.12 (m, 2H), 1.94 (s, 2H), 1.74 (d, J = 31.6 Hz, 8H), 1.34-1.16 (m, 10H)

^{13}C-NMR (101 MHz, CHLOROFORM-D) δ 158.0, 133.3, 133.2, 131.7, 131.6, 129.7, 127.6, 127.5, 104.1, 104.0, 55.6, 36.4, 36.3, 36.1, 35.9, 31.6, 30.7, 29.3, 26.6, 26.5, 26.4, 25.8, 25.7, 22.7, 14.1.

^{31}P-NMR (243 MHz, CHLOROFORM-D) δ 34.5.

2.2 General procedure for the synthesis of substrates

N,4-dimethyl-*N*-(2-(pent-1-yn-1-yl)phenyl)benzenesulfonamide **1a** was synthesized using following procedure:

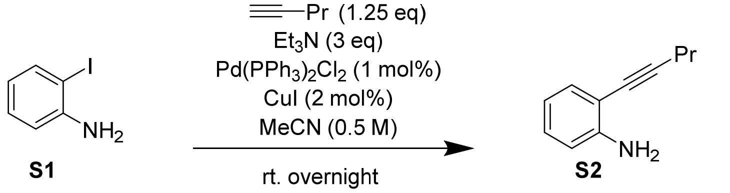

To an oven-dried 100 mL round bottom flask **S1** (4.40 g, 20 mmol), $Pd(PPh_3)_2Cl_2$ (140 mg, 0.2 mmol) and CuI (76 mg, 0.4 mmol) was charged, followed by addition of MeCN (40 mL) and Et_3N (8.4 mL, 60 mmol). Freeze-Pump-Thaw was conducted, and the flask was filled with nitrogen. Then, alkyne was added, and the mixture was warmed up to room temperature and stirred overnight. After fully consumption of starting material monitored by TLC, 20 mL of sat. NH_4Cl was added to quench the reaction. The mixture was extracted with EtOAc, washed with brine, dried over Na_2SO_4 and evaporated under reduced pressure. Then, the crude mixture was purified by flash silica gel column chromatography using hexane/EtOAc (50:1 to 10:1) as eluent. **S2** was obtained as an orange oil (3.28 g, >99%).

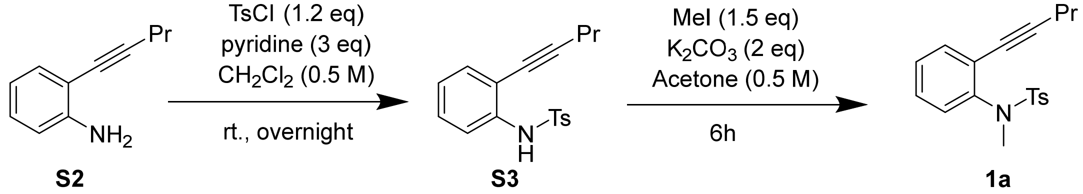

The obtained **S2** (3.28 g, ~20 mmol), TsCl (4.28 g, 25 mmol) was charged in a 100 mL round bottom flask. Dichloromethane (50 mL) was added, followed by the dropwise addition of pyridine (4.1 mL, 50 mmol). The reaction was stirred at room temperature overnight. After reaction, 20 mL of sat. $NaHCO_3$ was added to the mixture, and the mixture was stirred at room temperature for 1 hour until

no bubble was released. Then, the mixture was extracted with EtOAc, washed with water, 1N HCl and brine, dried over Na_2SO_4 and evaporated under reduced pressure. The crude mixture was washed by small amount of 10:1 of Hexane:EtOAc and hexane under cool bath. **S3** was obtained as colorless crystal and used in the next step without further purification.

S3 (est. 20 mmol) and anhydrous K_2CO_3 (5.60 g, 40 mmol) were charged into a 100 mL round bottle flask with a magnetic stirrer. 50 mL of acetone was added to the mixture, and then MeI (2 mL, 30 mmol) was added to the vessel. After stirring at room temperature for 6 h, 20 mL of water was added to the flask. The mixture was then extracted with EtOAc, washed with brine, dried over Na_2SO_4 and evaporated under reduced pressure. The crude mixture was purified by flash silica gel column chromatography using hexane/EtOAc (10:1 to 4:1) as eluent, and further recrystallized from hexane/EtOAc (about 10/1) under dryice-acetone bath to remove colored impurity. *N*-Methyl-2-(1-pentynyl)-*N*-tosylaniline (**1a)** was obtained as colorless crystal (5.10 g, 78 % yield).

[1] J. P. A. Harrity*, et al.*, *J. Organomet. Chem.*, **1997**, *532*, 219-227.
[2] S. S. Zalesskiy*, et al.*, *J. Am. Chem. Soc.*, **2013**, *135*, 3550-3559.
[3] R. E. Ebule*, et al.*, *Adv. Synth. Catal.*, **2016**, *358*, 1478-1481.

3. General procedure

To an oven-dried 2.5 mL glass reaction vessel, substrate 1 (0.25 mmol) and a magnetic stirrer bar were added under Ar atmosphere. Then, 0.5 mL of chlorobenzene was added to the vessel, and the vessel was put into a 0 °C cooling bath with magnetic stirrer for 15 minutes. SPhosAuCl (3.2 mg, 0.005 mmol) and $AgBF_4$ (2.9 mg, 0.0015 mmol) were dissolved and mixed in another 0.5 mL solvent to form a uniform suspension. Then, the catalyst mixture was injected into the vessel to start the reaction. After designated reaction time, the reaction was quenched using 1 mL of 1N $Na_2S_2O_3$ solution, extracted with EtOAc, dried over Na_2SO_4 and passed through a short silica gel pad to remove catalyst. Yield was determined by ^{1}H NMR using CH_2Br_2 as internal standard. Purification was conducted using medium-pressure liquid chromatography using silica gel as a stationary phase and hexane and EtOAc as eluent. Further purification, if necessary, was conducted gel permeation chromatography using chloroform as the solvent.

4. Optimization of reaction conditions

4.1 Counterions using 1:1 of Ag:Au

Table S1. Counteranion effect

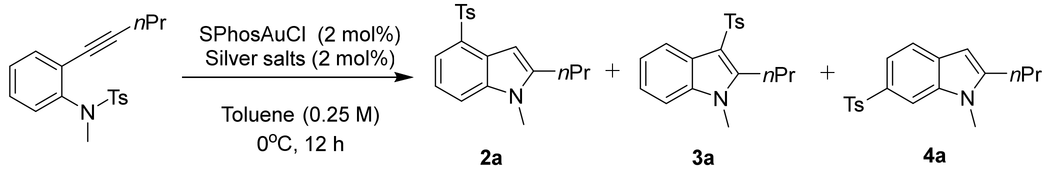

1a 2a 3a 4a

Entry	Silver salt	Yields[a]		
		**2a** (%)	**3a** (%)	**4a** (%)
1	$AgSbF_6$	23	46	20
2	$AgNTf_2$	17	56	21
3	$AgBF_4$	46	30	12
4	$Ag(CF_3COO)$	20	46	20
5	AgOTs	3	8	3

[a] NMR yield. CH_2Br_2 was used as internal standard.

4.2 Silver to gold (Ag:Au) ratio

Table S2. Silver to gold ratio

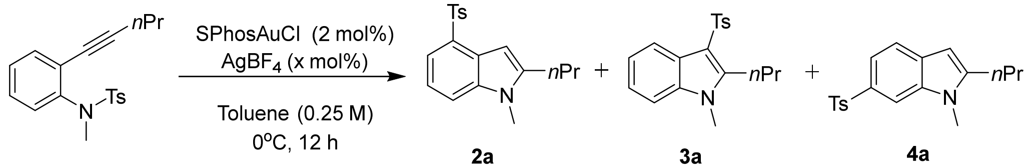

1a 2a 3a 4a

Entry	$AgBF_4$ (mol%)	Ag/Au	Time (h)	Yields[a]		
				**2a** (%)	**3a** (%)	**4a** (%)
1[b]	2	0.5	0.5	20	41	33
2	2	1	6	46-24[c]	30-44	12-27
4	4	2	6	65	16	7
5	6	3	12	70	10	5
6	8	4	12	74	10	4
7[d]	10	5	12	51	6	4
8[e]	20	10	12	37	10	6

[a] NMR yield. CH_2Br_2 was used as internal standard.

[b] 4 mol% of SPhosAuCl was used.

[c] Chemical yields of **2a**, **3a**, and **4a** varied in the reaction of which Ag:Au ratio was 1.

[d] 28 % of unreacted **1a** was recovered.

[e] 34% of unreacted **1a** was recovered.

4.3. Solvent effect

Table S3. Solvent effect

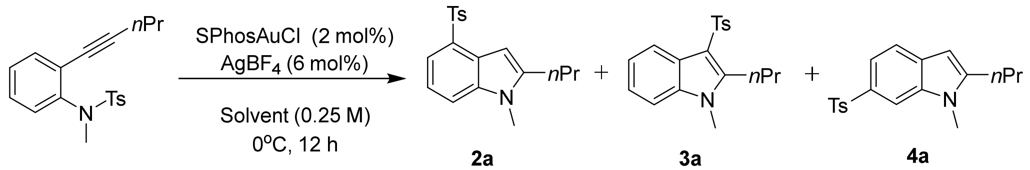

Entry	Solvents	Yields[a]		
		**2a** (%)	**3a** (%)	**4a** (%)
1	Toluene	70	10	5
2	PhF	68	18	10
3	PhCl	76	10	5
4	PhBr	75	11	8
5	MeCN	25	46	25
6	Acetone	24	46	24
7	1,2-DCE	40	37	20
8	EtOAc	7	4	2

4.4 Temperature effect

Table S4. Temperature effect

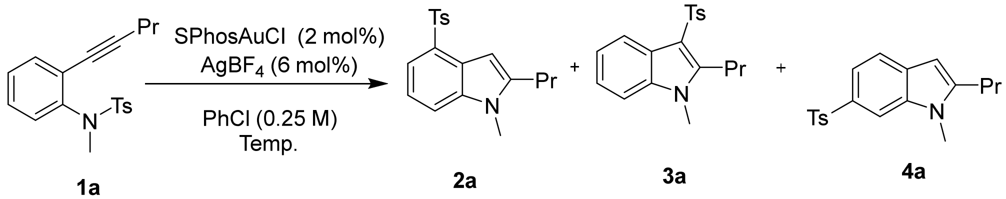

Entry	Temp. (°C)	Time (h)	Yields[a]		
			**2a** (%)	**3a** (%)	**4a** (%)
1	40	0.25	49	28	17
2	r.t. (21)	1	67	18	11
3	0	12	76	10	5
4[b]	-20	60	59	6	3

[a] NMR yield. CH_2Br_2 was used as internal standard.

[b] 31 % of unreacted **1a** recovered

5. 1 mmol-scale experiments

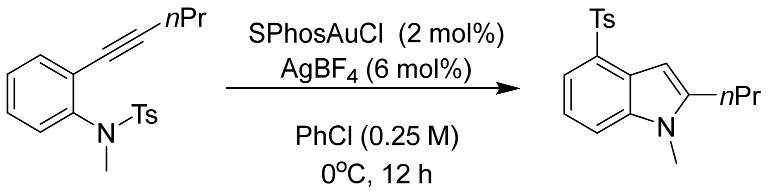

1a
1.0 mmol

2a
209.0 mg
(64% isolated yield)

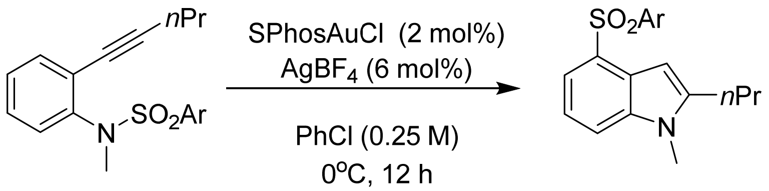

1e
(Ar = *p*-FC_6H_4)
1.0 mmol

2e
220.7 mg
(67% isolated yield)

6. Mechanistic studies

6.1 Stability of 4-sulfonylindole under reaction condition

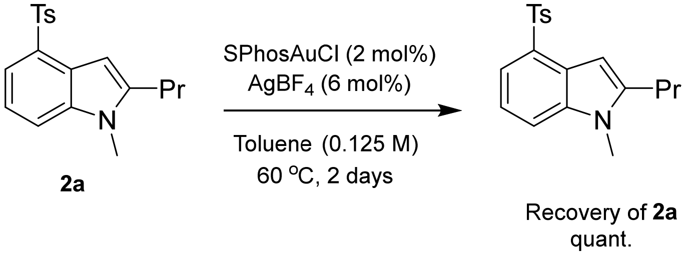

When 4-sulfonylindole **2a** was treated with catalyst at 60 Celsius, no significant conversion of 4-sulfonylindole was observed.

6.2 Control experiments

Table S5. Mechanism probing experiments

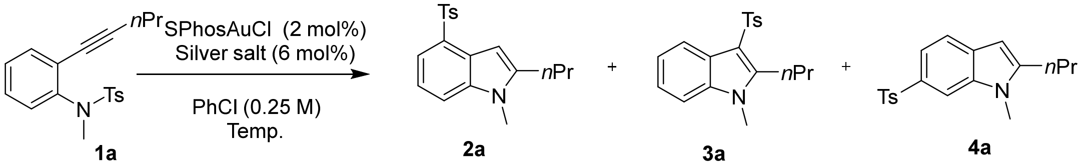

Entry	Au Cat. (2 mol%)	Activator (6 mol%)	Yields[a]		
			**2a** (%)	**3a** (%)	**4a** (%)
1	SPhosAuCl	$AgBF_4$	76	10	5
2	$SPhosAuBF_4$	-	21	45	30
3	SPhosAuCl	$AgSbF_6$	34	41	22
4	$SPhosAuBF_4$	$AgSbF_6$	38	36	15
5	$SPhosAuBF_4$	$AgBF_4$	77	13	5
6	SPhosAuCl	-	3	7	4
7[b]	-	$AgBF_4$	17	4	3

[a] NMR yield. CH_2Br_2 was used as internal standard.

[b] Reaction was carried at 30 Celsius for 2 Days. ~70 % Unreacted SM recovered

5.3 Crossover experiment

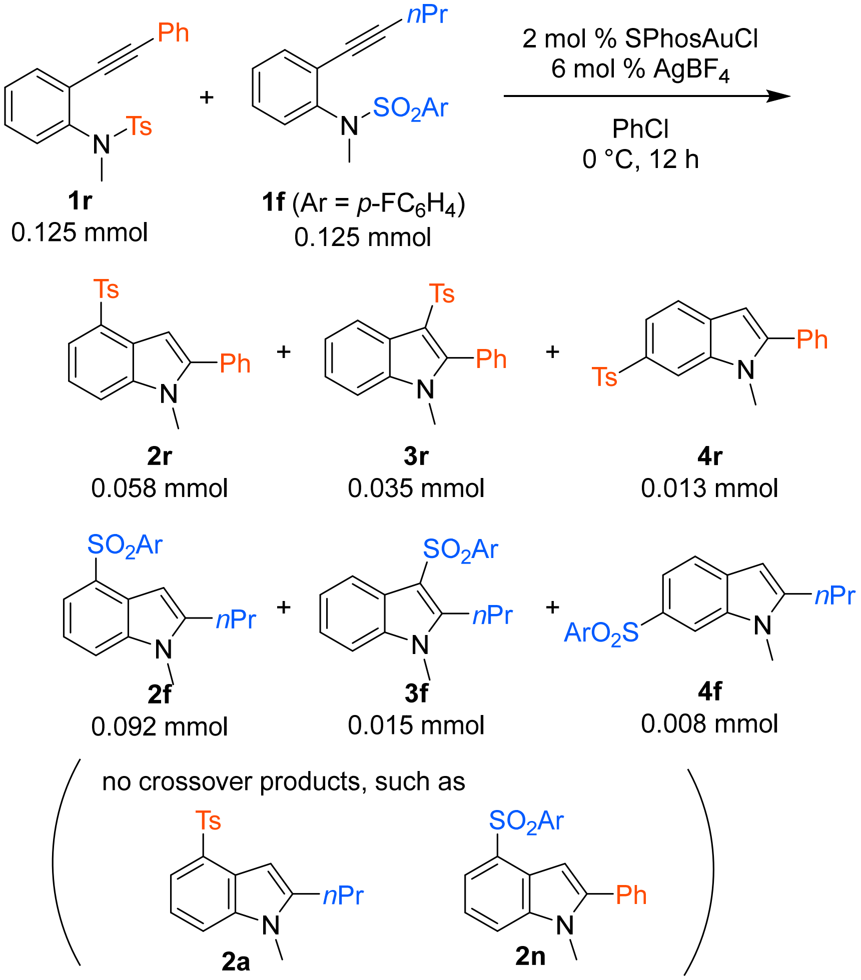

Cf.

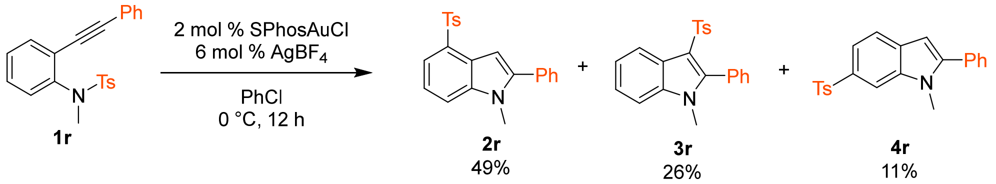

Scheme S1. Crossover experiments

TIC scan

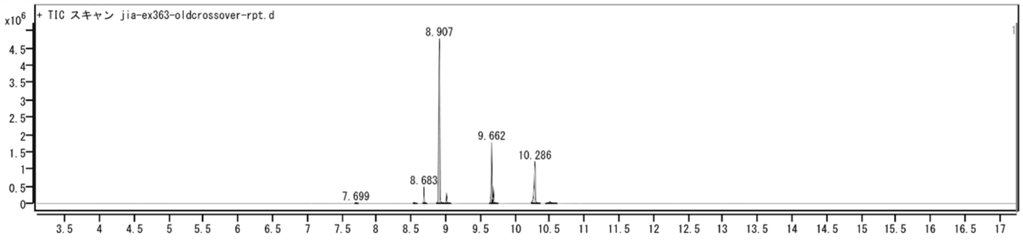

Retention time

Retention time = 7.694-7.711: a product derived from **1e**

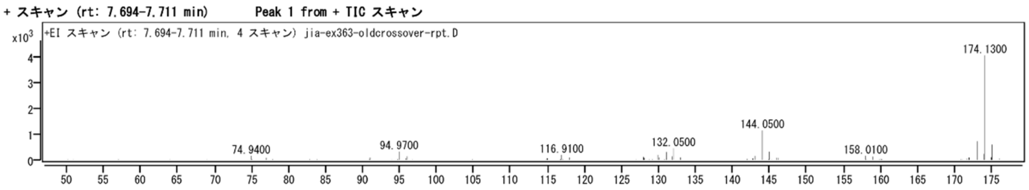

m/z

Retention time = 8.535-8.552: a product derived from **1e**

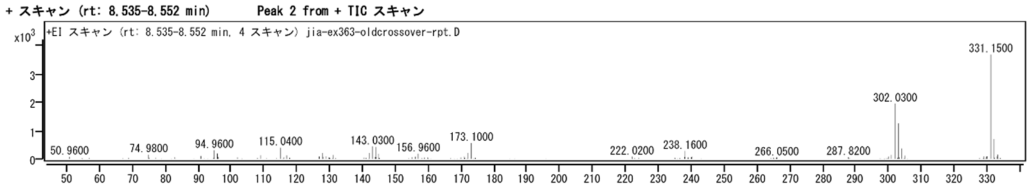

m/z

Retention time = 8.678-8.689: a product derived from **1e**

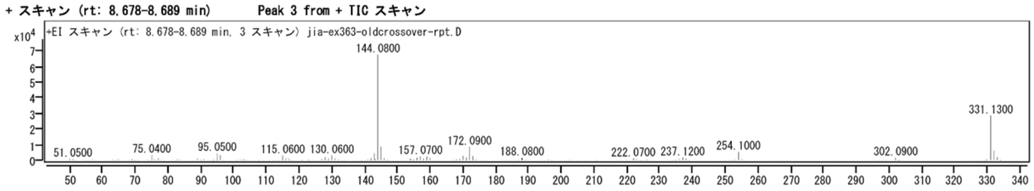

m/z

Retention time = 8.889-8.912: a product derived from **1e**

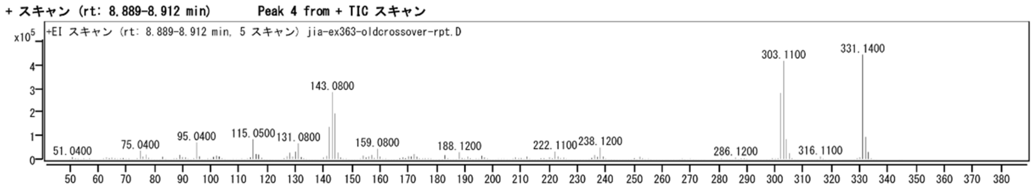

m/z

Retention time = 9.004-9.021: a product derived from **1e**

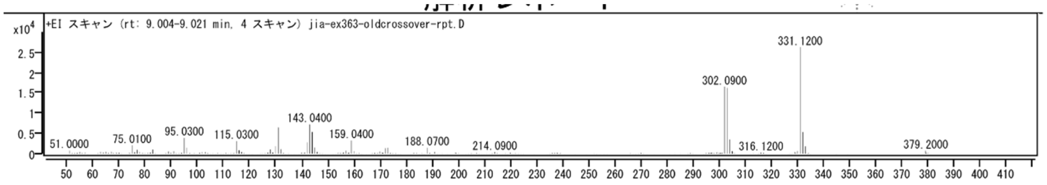

m/z

Retention time = 9.650-9.668: a product derived from **1r**

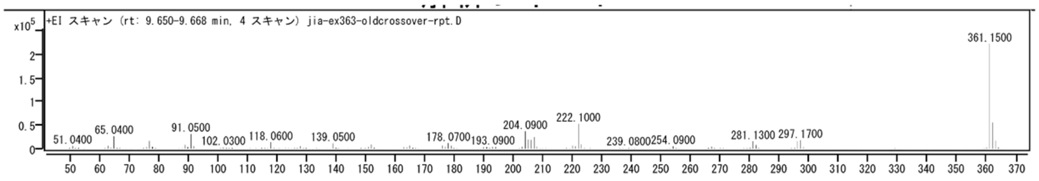

m/z

Retention time = 9.673-9.696: a product derived from **1r**

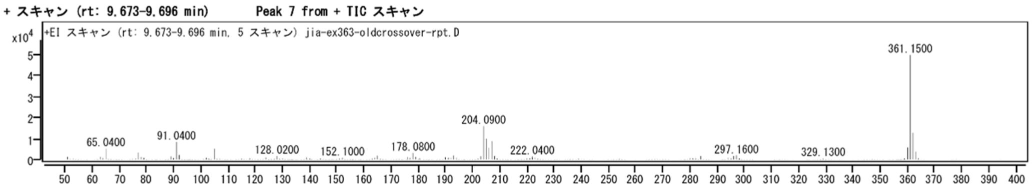

m/z

Retention time = 10.263-10.297: a product derived from **1r**

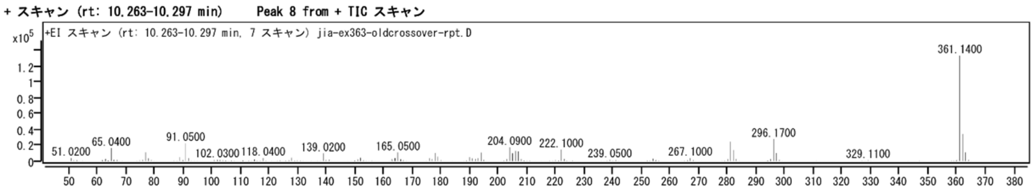

m/z

Retention time = 10.474-10.549: a product derived from **1r**

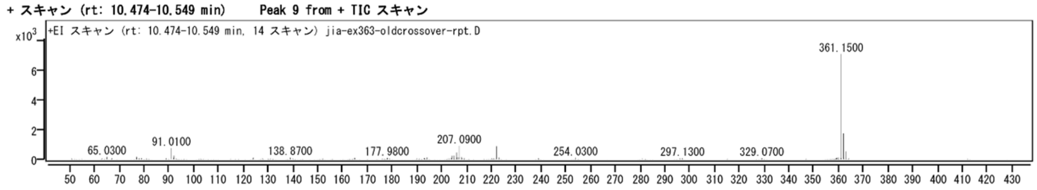

m/z

7. Computational analysis

7.1 General

Method: Structures were optimized under B3LYP-D3(BJ)/def2SVP level. SMD solvation model using toluene as the solvent and Solvent Accessible Surface (SAS) was also applied to describe the solvation effect. Free energy was obtained from output files.

Me_3PAu^+-catalyzed cycloisomerization reactions

Cartesian coordinates of selected optimized structures are shown below. All calculated transition states have only one imaginary frequency, and Intrinsic Reaction Coordinate was calculated to ensure the transition states are correct.

7.2 Preliminary calculations for sulfonyl walking mechanism

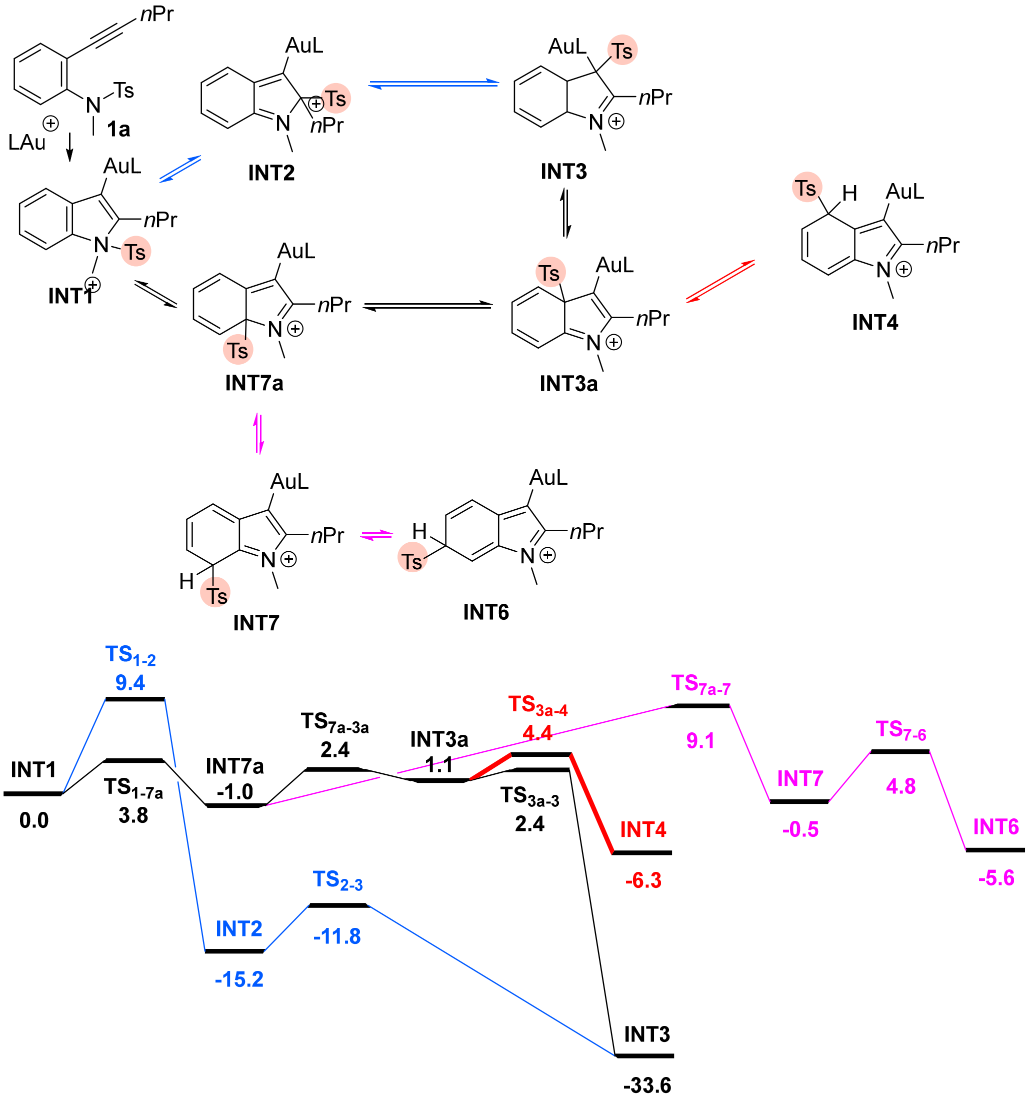

L = PMe_3

ΔG (kcal/mol) at the level of B3LYP-D(BJ)/def2SVP,SMD(toluene,SAS)

Scheme S2. Au-catalyzed reaction coordinates

Table S6. Calculated energies of intermediates and transition states

Compound	SCF energy / a.u.	E(ZPVE) / a.u.	H / a.u.	G(298) / a.u.	Imaginary frequency /cm^{-1}
**INT1**	-1935.912769	-1935.436077	-1935.403120	-1935.501159	-
**TS1-2**	-1935.895951	-1935.420822	-1935.388038	-1935.486145	-169.58
**INT2**	-1935.938452	-1935.461290	-1935.428378	-1935.525468	-
**TS2-3**	-1935.931651	-1935.455469	-1935.422872	-1935.519973	-82.19
**INT3**	-1935.966024	-1935.488045	-1935.455087	-1935.5546	-
**TS1-7a**	-1935.904159	-1935.429090	-1935.396178	-1935.495181	-41.86
**INT7a**	-1935.913570	-1935.437514	-1935.404310	-1935.502810	-

**TS7a-3a**	-1935.903296	-1935.428709	-1935.395550	-1935.495610	-59.30
**INT3a**	-1935.911072	-1935.435008	-1935.401757	-1935.499354	-
**TS3-3a**	-1935.908137	-1935.432993	-1935.400189	-1935.497404	-142.19
**TS3a-4**	-1935.902480	-1935.427753	-1935.394606	-1935.494070	-110.74
**INT4**	-1935.921198	-1935.444482	-1935.411257	-1935.511234	-
**TS7a-7**	-1935.895565	-1935.420605	-1935.387638	-1935.486638	-208.33
**INT7**	-1935.912494	-1935.435960	-1935.402877	-1935.501902	-
**TS7-6**	-1935.904407	-1935.428963	-1935.396177	-1935.493576	-88.48
**INT6**	-1935.920098	-1935.443495	-1935.410329	-1935.510068	-

Cartesian Coordinates

INT1

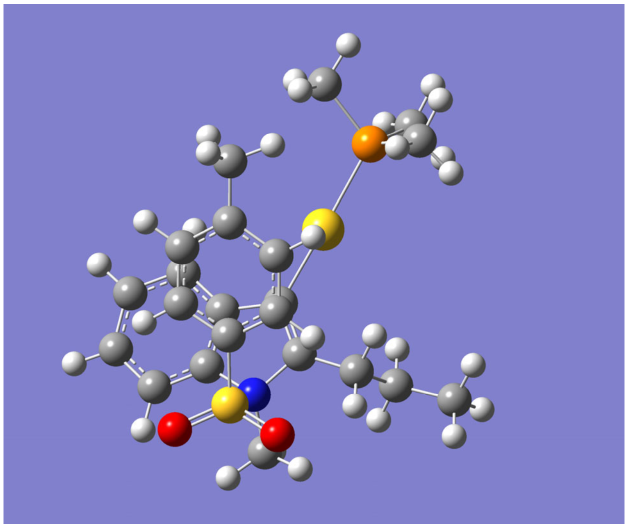

Center Number	Atomic Number	Atomic type	X	Y	Z
1	6	0	3.647855	-0.819692	-2.040025
2	6	0	2.608111	-0.156560	-1.404576
3	6	0	0.919726	-1.530974	-2.439831
4	6	0	1.949647	-2.219406	-3.092221
5	6	0	3.293156	-1.869604	-2.899226
6	1	0	4.693305	-0.559541	-1.877777
7	1	0	-0.126622	-1.799685	-2.596515
8	1	0	1.704562	-3.039936	-3.769912
9	1	0	4.077449	-2.416941	-3.425096
10	6	0	1.252611	-0.480108	-1.583649
11	7	0	2.683991	0.905949	-0.421446
12	6	0	0.432998	0.428974	-0.769585
13	6	0	1.281242	1.290755	-0.146522
14	6	0	3.679873	1.972020	-0.663694
15	1	0	3.445300	2.464416	-1.616958
16	1	0	3.647923	2.695035	0.157983
17	1	0	4.681170	1.525512	-0.711563
18	6	0	0.978530	2.470957	0.722589
19	1	0	0.089015	2.212317	1.319516
20	1	0	1.788948	2.649772	1.444605
21	6	0	0.677124	3.762259	-0.060940
22	1	0	1.549511	4.036313	-0.678403
23	1	0	-0.141887	3.553610	-0.769256
24	6	0	0.305924	4.923275	0.855380
25	1	0	1.122886	5.162826	1.555862

26	1	0	0.085602	5.834174	0.278049
27	1	0	-0.586579	4.687500	1.458614
28	16	0	3.374862	-0.031582	1.315186
29	6	0	2.006523	-1.073152	1.642807
30	6	0	1.911304	-2.297656	0.969444
31	6	0	0.999261	-0.608851	2.497358
32	6	0	0.765430	-3.061615	1.157806
33	1	0	2.711139	-2.633249	0.309450
34	6	0	-0.132017	-1.396381	2.670179
35	1	0	1.105271	0.349090	3.006102
36	6	0	-0.274257	-2.623619	1.997811
37	1	0	0.671155	-4.015221	0.634012
38	1	0	-0.927007	-1.048367	3.332839
39	6	0	-1.503192	-3.465609	2.187500
40	1	0	-1.307257	-4.276738	2.910007
41	1	0	-2.344475	-2.876606	2.579878
42	1	0	-1.812984	-3.943988	1.246101
43	8	0	4.543858	-0.756544	0.838701
44	8	0	3.460982	1.086219	2.246196
45	79	0	-1.602175	0.307796	-0.504932
46	15	0	-3.920718	0.113410	-0.155094
47	6	0	-4.941662	1.140518	-1.273196
48	1	0	-6.015200	1.010766	-1.065086
49	1	0	-4.673567	2.199982	-1.145540
50	1	0	-4.738635	0.859696	-2.317453
51	6	0	-4.564620	-1.586472	-0.373718
52	1	0	-4.056983	-2.265655	0.327590
53	1	0	-5.650382	-1.628772	-0.194816
54	1	0	-4.354813	-1.930904	-1.397444
55	6	0	-4.459912	0.590585	1.528491
56	1	0	-5.548973	0.478084	1.647127
57	1	0	-3.949831	-0.040591	2.271712
58	1	0	-4.183162	1.638097	1.720364

TS1-2

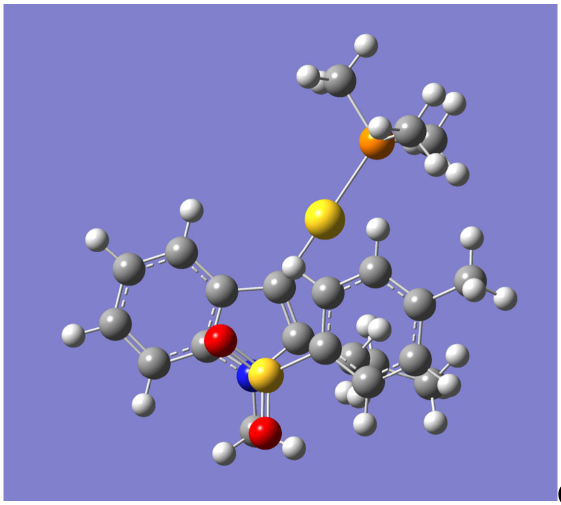

(Mirror inverted)

Center Number	Atomic Number	Atomic type	X	Y	Z
1	6	0	2.675296	-4.363632	-1.115563
2	6	0	3.234348	-3.437388	-0.249960
3	6	0	2.423814	-2.357737	0.142137
4	6	0	1.082749	-2.190299	-0.321508
5	6	0	0.554232	-3.139603	-1.209233
6	6	0	1.349754	-4.213110	-1.592506
7	1	0	3.265706	-5.222180	-1.440769
8	1	0	-0.465647	-3.030974	-1.582828
9	1	0	0.949432	-4.962313	-2.278962
10	6	0	0.530246	-0.985120	0.269142
11	6	0	1.510388	-0.462151	1.069782
12	6	0	1.454920	0.664309	2.044557
13	1	0	0.763609	1.415366	1.631555
14	1	0	2.431416	1.166907	2.135754
15	7	0	2.738099	-1.276706	0.934760
16	6	0	3.812869	-1.307446	1.914877
17	1	0	4.743651	-1.627767	1.429954
18	1	0	3.976174	-0.306446	2.325978
19	1	0	3.562051	-2.003375	2.731264
20	16	0	3.388687	0.333175	-0.711247
21	8	0	3.076830	-0.543151	-1.838290
22	8	0	4.736190	0.555895	-0.178582
23	6	0	0.964249	0.246955	3.444193
24	1	0	1.637397	-0.522936	3.858279
25	1	0	-0.020067	-0.236615	3.333336
26	6	0	0.873284	1.429878	4.402129

27	1	0	1.855449	1.908913	4.549558
28	1	0	0.182852	2.200575	4.020939
29	1	0	0.507099	1.115472	5.391216
30	6	0	2.486350	1.845520	-0.806255
31	6	0	2.954596	2.956231	-0.093586
32	6	0	1.305706	1.876322	-1.556679
33	6	0	2.218289	4.134282	-0.159023
34	1	0	3.881121	2.898451	0.478242
35	6	0	0.593110	3.069072	-1.603599
36	1	0	0.964359	0.988554	-2.087625
37	6	0	1.029390	4.212579	-0.908415
38	1	0	2.575372	5.015560	0.378622
39	1	0	-0.323263	3.115898	-2.195443
40	6	0	0.264343	5.502619	-0.982017
41	1	0	-0.752938	5.356604	-1.371585
42	1	0	0.195422	5.986946	0.004180
43	1	0	0.776115	6.213661	-1.653274
44	1	0	4.258571	-3.547744	0.108376
45	79	0	-1.336016	-0.172046	-0.037761
46	15	0	-3.471039	0.753342	-0.379560
47	6	0	-4.358410	1.167248	1.167116
48	6	0	-3.471675	2.318935	-1.332130
49	6	0	-4.624563	-0.334803	-1.292847
50	1	0	-5.349386	1.599208	0.956921
51	1	0	-4.482643	0.256782	1.772439
52	1	0	-3.766285	1.887835	1.750989
53	1	0	-3.032136	2.145279	-2.325762
54	1	0	-4.493562	2.710034	-1.457113
55	1	0	-2.863513	3.068097	-0.803773
56	1	0	-5.609058	0.142330	-1.418682
57	1	0	-4.206223	-0.567661	-2.283501
58	1	0	-4.748965	-1.279691	-0.742759

INT2

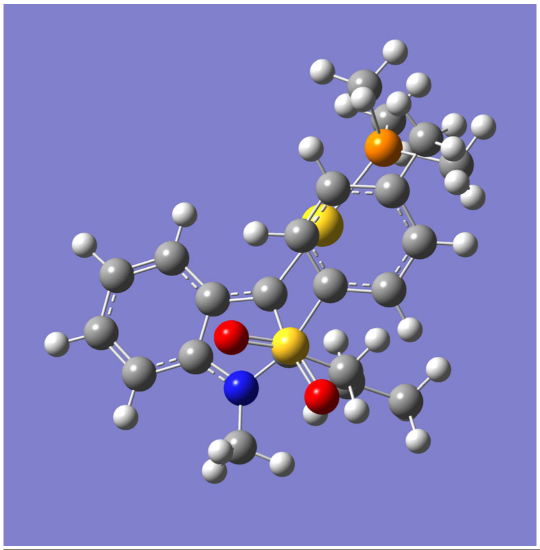

Center Number	Atomic Number	Atomic type	X	Y	Z
1	6	0	-4.523897	-1.998289	-2.143656
2	6	0	-4.656508	-1.019570	-1.178022
3	6	0	-3.482971	-0.622455	-0.502267
4	6	0	-2.196296	-1.199411	-0.835437
5	6	0	-2.118452	-2.212642	-1.838885
6	6	0	-3.268813	-2.600721	-2.476684
7	1	0	-5.415540	-2.327643	-2.682919
8	1	0	-5.625249	-0.573058	-0.955468
9	1	0	-1.148733	-2.650610	-2.082689
10	1	0	-3.242168	-3.368006	-3.251929
11	6	0	-1.215020	-0.594544	-0.059960
12	6	0	-1.914091	0.445822	0.743493
13	6	0	-1.501868	0.632011	2.192966
14	1	0	-0.417194	0.822545	2.203146
15	1	0	-1.980336	1.536648	2.596058
16	7	0	-3.330655	0.274468	0.490619
17	6	0	-4.383789	1.138955	0.993941
18	1	0	-4.636692	1.921205	0.260795
19	1	0	-4.053666	1.624223	1.918175
20	1	0	-5.277718	0.538679	1.214563
21	16	0	-1.580492	2.145527	-0.224307
22	8	0	-2.264783	1.991133	-1.512818
23	8	0	-1.984269	3.176558	0.742483
24	79	0	0.795218	-0.983144	0.053037
25	15	0	3.095742	-1.436918	0.253095
26	6	0	3.479692	-3.137409	0.805081

27	1	0	4.566577	-3.285763	0.901864
28	1	0	3.079835	-3.860553	0.078605
29	1	0	3.000852	-3.326641	1.777373
30	6	0	4.038005	-1.230982	-1.300304
31	1	0	5.108683	-1.434600	-1.144102
32	1	0	3.911875	-0.206103	-1.674478
33	1	0	3.646560	-1.924721	-2.059299
34	6	0	3.931801	-0.353668	1.466636
35	1	0	3.743991	0.697910	1.208802
36	1	0	5.016293	-0.542445	1.489741
37	1	0	3.513991	-0.538662	2.467627
38	6	0	0.171672	2.283659	-0.507754
39	6	0	0.986705	2.831888	0.485819
40	6	0	0.686554	1.870259	-1.737406
41	6	0	2.353969	2.940730	0.241024
42	1	0	0.550810	3.186085	1.420694
43	6	0	2.056083	1.999082	-1.965101
44	1	0	0.019121	1.470690	-2.501423
45	6	0	2.910660	2.528607	-0.983652
46	1	0	3.000169	3.378790	1.005987
47	1	0	2.465418	1.698368	-2.932228
48	6	0	4.389453	2.661683	-1.227532
49	1	0	4.968890	2.029407	-0.533594
50	1	0	4.725513	3.697811	-1.063466
51	1	0	4.663953	2.378362	-2.253206
52	6	0	-1.821887	-0.589998	3.060097
53	1	0	-2.907692	-0.781378	3.031831
54	1	0	-1.341389	-1.481025	2.622099
55	6	0	-1.362728	-0.405740	4.503035
56	1	0	-1.843817	0.468511	4.971057
57	1	0	-0.272140	-0.253892	4.560796
58	1	0	-1.608277	-1.286943	5.114856

TS2-3

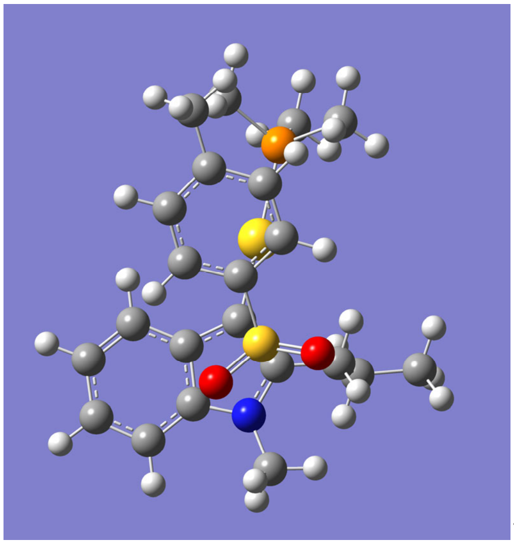

’**Mirror-inverted image**

Center Number	Atomic Number	Atomic type	X	Y	Z
1	6	0	3.581534	2.297026	-2.631731
2	6	0	4.055436	1.874998	-1.392367
3	6	0	3.193054	1.095269	-0.618205
4	6	0	1.879524	0.752828	-1.062268
5	6	0	1.431372	1.200725	-2.326345
6	6	0	2.285718	1.966630	-3.099855
7	1	0	4.230181	2.907035	-3.264553
8	1	0	0.428914	0.938400	-2.670714
9	1	0	1.968449	2.323393	-4.081197
10	6	0	1.243320	-0.003033	-0.041776
11	6	0	2.216325	-0.058817	1.041559
12	6	0	2.159479	-0.992668	2.203613
13	1	0	1.100429	-1.145467	2.456577
14	1	0	2.622642	-0.536645	3.090821
15	7	0	3.384382	0.548333	0.640900
16	6	0	4.540326	0.838298	1.469908
17	1	0	4.555906	1.906094	1.738534
18	1	0	4.498754	0.243333	2.388285
19	1	0	5.463498	0.580345	0.931552
20	16	0	1.094861	1.865523	1.820812
21	8	0	1.943848	2.894055	1.195916
22	8	0	1.166010	1.555846	3.259400
23	6	0	-0.606118	2.149787	1.378753
24	6	0	-1.608155	1.666759	2.221674
25	6	0	-0.891853	2.796566	0.173791
26	6	0	-2.936411	1.844176	1.838058

27	1	0	-1.347036	1.175176	3.159369
28	6	0	-2.226114	2.952264	-0.190856
29	1	0	-0.083474	3.167930	-0.456297
30	6	0	-3.267752	2.481367	0.629721
31	1	0	-3.734447	1.496525	2.498918
32	1	0	-2.467683	3.461829	-1.126642
33	6	0	2.813773	-2.348210	1.877770
34	1	0	3.871874	-2.189358	1.609877
35	1	0	2.331244	-2.769720	0.980016
36	6	0	2.708873	-3.327933	3.041859
37	1	0	3.203470	-2.935213	3.945025
38	1	0	1.657011	-3.530470	3.301856
39	1	0	3.182367	-4.289941	2.793690
40	6	0	-4.704545	2.696627	0.240884
41	1	0	-5.372416	1.957892	0.707623
42	1	0	-5.045609	3.693107	0.571143
43	1	0	-4.844000	2.657853	-0.849859
44	1	0	5.051400	2.152647	-1.046999
45	79	0	-0.609883	-0.888375	-0.084938
46	15	0	-2.737080	-1.886936	-0.173053
47	6	0	-2.737617	-3.577460	-0.870273
48	6	0	-3.544637	-2.053667	1.459270
49	6	0	-3.937769	-0.958788	-1.193604
50	1	0	-3.755709	-3.996610	-0.891724
51	1	0	-2.334241	-3.554199	-1.893660
52	1	0	-2.091871	-4.227583	-0.261227
53	1	0	-3.646351	-1.059792	1.918390
54	1	0	-4.540428	-2.513932	1.364200
55	1	0	-2.921483	-2.676484	2.118315
56	1	0	-4.920230	-1.455897	-1.200445
57	1	0	-4.047547	0.058880	-0.792871
58	1	0	-3.563900	-0.884891	-2.225706

INT3

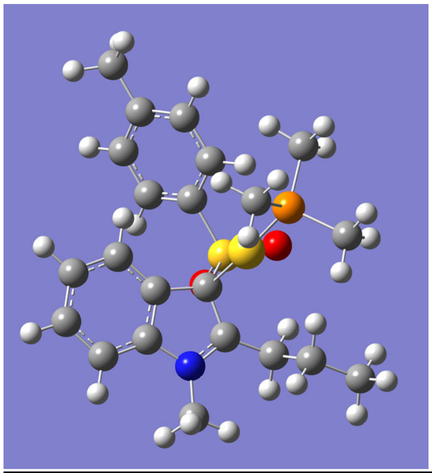

Center Number	Atomic Number	Atomic type	X	Y	Z
1	6	0	0.737436	-3.248517	3.306007
2	6	0	-0.436475	-3.448606	2.579328
3	6	0	-0.583121	-2.716395	1.401434
4	6	0	0.376285	-1.788265	0.948995
5	6	0	1.538761	-1.588679	1.703090
6	6	0	1.710337	-2.333086	2.870892
7	1	0	0.897910	-3.808495	4.229221
8	1	0	-1.197511	-4.149688	2.923624
9	1	0	2.290708	-0.864358	1.394517
10	1	0	2.616224	-2.194556	3.464365
11	6	0	-0.191748	-1.154598	-0.267022
12	6	0	-1.438609	-1.866236	-0.515981
13	6	0	-2.352814	-1.704459	-1.683437
14	1	0	-1.763830	-1.317141	-2.524946
15	1	0	-2.733551	-2.693689	-1.982867
16	7	0	-1.655174	-2.727900	0.485996
17	6	0	-2.827594	-3.565454	0.683272
18	1	0	-3.368249	-3.250146	1.588263
19	1	0	-3.497884	-3.490674	-0.178214
20	1	0	-2.514532	-4.612766	0.799569
21	16	0	0.822414	-0.972977	-1.789622
22	8	0	0.144120	0.028804	-2.633591
23	8	0	1.056351	-2.328932	-2.301074
24	79	0	-0.813382	0.864017	0.290643
25	15	0	-1.362921	3.054975	0.809783
26	6	0	-2.996214	3.546627	0.157792
27	1	0	-3.214749	4.598633	0.399502
28	1	0	-3.009296	3.416101	-0.934560

29	1	0	-3.776713	2.906274	0.594936
30	6	0	-0.177478	4.248206	0.096800
31	1	0	-0.469823	5.281782	0.339505
32	1	0	0.827958	4.055611	0.499457
33	1	0	-0.147735	4.129800	-0.996688
34	6	0	-1.412252	3.421522	2.597231
35	1	0	-0.431177	3.203708	3.044782
36	1	0	-1.665600	4.478735	2.773184
37	1	0	-2.163590	2.782773	3.084796
38	6	0	2.372906	-0.300474	-1.229162
39	6	0	2.491034	1.068512	-0.980896
40	6	0	3.454084	-1.168360	-1.062840
41	6	0	3.709758	1.566403	-0.523692
42	1	0	1.644656	1.733440	-1.159557
43	6	0	4.666449	-0.650421	-0.612188
44	1	0	3.333446	-2.229814	-1.282604
45	6	0	4.814915	0.718949	-0.330678
46	1	0	3.814671	2.636235	-0.328004
47	1	0	5.518083	-1.321823	-0.478158
48	6	0	6.139080	1.264894	0.127203
49	1	0	6.628928	0.588093	0.843607
50	1	0	6.825626	1.379194	-0.729310
51	1	0	6.034318	2.252014	0.599653
52	6	0	-3.534656	-0.745889	-1.427082
53	1	0	-3.122713	0.242090	-1.157353
54	1	0	-4.113170	-1.086290	-0.550959
55	6	0	-4.442192	-0.622183	-2.645605
56	1	0	-5.267250	0.080984	-2.454576
57	1	0	-3.883195	-0.256463	-3.521527
58	1	0	-4.889150	-1.592306	-2.916895

TS1-7a

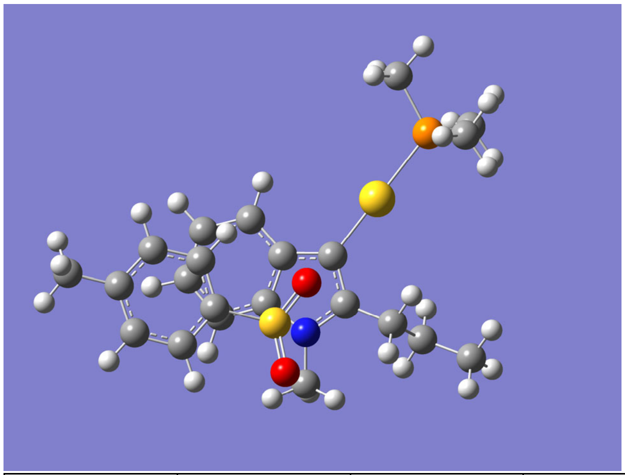

Center Number	Atomic Number	Atomic type	X	Y	Z
1	6	0	-0.605213	2.781731	-0.727865
2	6	0	-1.332542	1.662306	-0.306349
3	6	0	-0.604412	0.625768	0.262912
4	6	0	0.792947	0.691622	0.488207
5	6	0	1.495811	1.831997	0.058369
6	6	0	0.789553	2.864152	-0.552415
7	1	0	-1.136157	3.610656	-1.200077
8	1	0	2.573818	1.899106	0.218332
9	1	0	1.316567	3.757994	-0.891425
10	6	0	1.205632	-0.515826	1.156290
11	6	0	0.034712	-1.289888	1.321078
12	6	0	-0.084010	-2.641920	1.941395
13	1	0	0.890428	-3.138646	1.821738
14	1	0	-0.810039	-3.252922	1.380032
15	7	0	-1.038140	-0.651034	0.748117
16	6	0	-2.440388	-0.956942	0.982409
17	1	0	-3.038719	-0.644210	0.118284
18	1	0	-2.570427	-2.036579	1.110201
19	1	0	-2.795869	-0.429094	1.881524
20	16	0	-0.690768	-1.432973	-1.606699
21	8	0	-1.862700	-2.325813	-1.671502
22	8	0	0.671746	-1.913266	-1.317694
23	6	0	-0.701781	-0.263607	-2.933735
24	6	0	-1.895239	-0.064514	-3.637304
25	6	0	0.448110	0.492198	-3.182073
26	6	0	-1.909904	0.893537	-4.646477
27	1	0	-2.777375	-0.664735	-3.411794
28	6	0	0.404511	1.439117	-4.199099

29	1	0	1.351908	0.335609	-2.592924
30	6	0	-0.767292	1.657122	-4.947346
31	1	0	-2.827769	1.049690	-5.217946
32	1	0	1.298328	2.028787	-4.414824
33	6	0	-0.789508	2.665846	-6.059724
34	1	0	-1.795480	3.083038	-6.211738
35	1	0	-0.091745	3.494965	-5.871814
36	1	0	-0.485172	2.193271	-7.010109
37	6	0	-0.457409	-2.611532	3.434642
38	1	0	-1.424764	-2.096561	3.565216
39	1	0	0.287544	-1.997885	3.968666
40	6	0	-0.526534	-4.007754	4.044461
41	1	0	-1.284195	-4.630822	3.541471
42	1	0	0.440398	-4.530230	3.957951
43	1	0	-0.787489	-3.963950	5.112895
44	1	0	-2.412887	1.611267	-0.446808
45	79	0	3.097737	-1.049890	1.728319
46	15	0	5.277236	-1.681159	2.367911
47	6	0	5.576194	-1.609879	4.171977
48	6	0	5.715029	-3.393508	1.893382
49	6	0	6.600605	-0.647201	1.640721
50	1	0	6.605252	-1.916265	4.416939
51	1	0	5.408508	-0.583883	4.532303
52	1	0	4.868479	-2.274670	4.689420
53	1	0	5.629654	-3.506024	0.802143
54	1	0	6.741442	-3.643128	2.204581
55	1	0	5.013360	-4.096761	2.366502
56	1	0	7.595567	-0.985785	1.969449
57	1	0	6.546505	-0.698294	0.542938
58	1	0	6.457774	0.400681	1.944366

INT7a

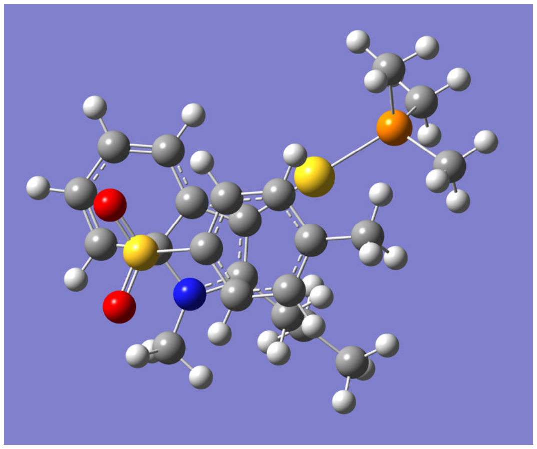

Center Number	Atomic Number	Atomic type	X	Y	Z
1	6	0	3.951641	-1.011568	-2.634237
2	6	0	4.014112	0.031048	-1.756818
3	6	0	2.834253	0.263093	-0.949301
4	6	0	1.538608	-0.286720	-1.368986
5	6	0	1.546132	-1.385084	-2.272529
6	6	0	2.729132	-1.741574	-2.868409
7	1	0	4.844748	-1.298463	-3.192589
8	1	0	4.938847	0.578498	-1.572215
9	1	0	0.607912	-1.883339	-2.523484
10	1	0	2.752431	-2.572433	-3.576235
11	6	0	0.519553	0.447452	-0.753880
12	6	0	1.180269	1.516522	-0.070443
13	6	0	0.514012	2.624625	0.668929
14	1	0	-0.414147	2.221759	1.100254
15	1	0	1.136079	2.970836	1.508662
16	7	0	2.527142	1.436739	-0.211816
17	6	0	3.541860	2.395846	0.182648
18	1	0	4.375768	1.868110	0.664744
19	1	0	3.121937	3.117490	0.891667
20	1	0	3.913320	2.940860	-0.700731
21	16	0	3.334545	-1.062820	0.710108
22	8	0	3.567037	-2.378408	0.116755
23	8	0	4.393584	-0.322627	1.407755
24	79	0	-1.498450	0.050695	-0.689033
25	15	0	-3.802273	-0.406353	-0.579308
26	6	0	-4.617698	0.283851	0.907701
27	6	0	-4.768818	0.257947	-1.983537
28	6	0	-4.217690	-2.188732	-0.538081

29	1	0	-5.692595	0.045198	0.921759
30	1	0	-4.491672	1.376821	0.922499
31	1	0	-4.144555	-0.129312	1.810845
32	1	0	-5.838997	0.020605	-1.878687
33	1	0	-4.393222	-0.171762	-2.924220
34	1	0	-4.643428	1.349997	-2.032202
35	1	0	-3.827467	-2.679901	-1.441980
36	1	0	-5.306794	-2.343145	-0.485998
37	1	0	-3.746217	-2.657492	0.338498
38	6	0	0.169984	3.814199	-0.252343
39	1	0	1.096255	4.202834	-0.708709
40	1	0	-0.449993	3.442910	-1.084784
41	6	0	-0.555404	4.928820	0.493508
42	1	0	0.059583	5.334846	1.313134
43	1	0	-1.499726	4.568704	0.933733
44	1	0	-0.799527	5.762225	-0.182561
45	6	0	1.844734	-1.070232	1.668485
46	6	0	1.635452	-0.065189	2.618099
47	6	0	0.880991	-2.044219	1.400074
48	6	0	0.427605	-0.045014	3.309319
49	1	0	2.414856	0.670621	2.818921
50	6	0	-0.313955	-2.010872	2.112552
51	1	0	1.071072	-2.809288	0.646853
52	6	0	-0.566036	-1.010345	3.066129
53	1	0	0.252003	0.732286	4.056994
54	1	0	-1.068900	-2.776145	1.921423
55	6	0	-1.861830	-0.971687	3.826653
56	1	0	-1.697797	-1.211418	4.890539
57	1	0	-2.588737	-1.696806	3.434059
58	1	0	-2.313582	0.032533	3.794682

TS7a-3a

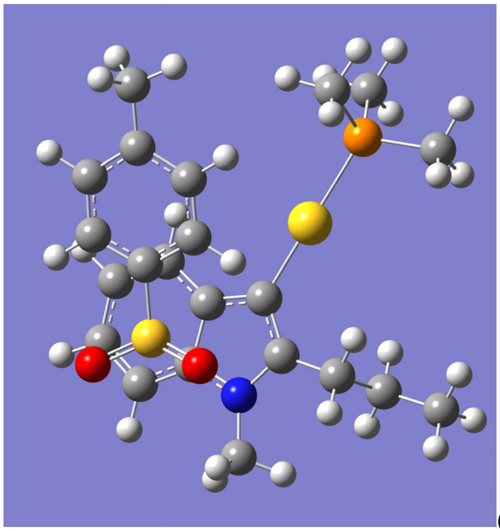

(Mirror-inverted image)

Center Number	Atomic Number	Atomic type	X	Y	Z
1	6	0	-3.699172	-1.518172	-1.378205
2	6	0	-3.820944	-0.193392	-1.038112
3	6	0	-2.635546	0.484202	-0.650056
4	6	0	-1.322277	-0.148706	-0.708223
5	6	0	-1.266408	-1.535571	-1.055567
6	6	0	-2.426273	-2.191188	-1.380051
7	1	0	-4.591114	-2.085093	-1.651806
8	1	0	-0.300001	-2.042617	-1.067873
9	1	0	-2.397005	-3.247730	-1.653318
10	6	0	-0.343404	0.821371	-0.396255
11	6	0	-1.066144	2.009265	-0.166101
12	6	0	-0.519673	3.340436	0.209930
13	1	0	0.432815	3.169082	0.732734
14	1	0	-1.189115	3.845708	0.924212
15	7	0	-2.429204	1.812020	-0.369422
16	6	0	-3.491861	2.761521	-0.094941
17	1	0	-3.135376	3.783635	-0.264176
18	1	0	-4.332957	2.583973	-0.778437
19	1	0	-3.839674	2.667616	0.946182
20	16	0	-2.458393	-0.335960	1.769369
21	8	0	-3.779254	-0.967083	1.883605
22	8	0	-2.128642	1.004222	2.285726
23	6	0	-1.183854	-1.476300	2.222752
24	6	0	-1.456799	-2.848575	2.162889
25	6	0	0.071477	-0.974439	2.574685
26	6	0	-0.434844	-3.733635	2.482403

27	1	0	-2.449827	-3.202342	1.885617
28	6	0	1.075570	-1.887137	2.891401
29	1	0	0.245709	0.099614	2.611906
30	6	0	0.845874	-3.271914	2.847408
31	1	0	-0.631989	-4.808003	2.456936
32	1	0	2.058300	-1.513841	3.185547
33	6	0	1.927637	-4.252241	3.201867
34	1	0	2.897263	-3.758275	3.355203
35	1	0	1.675181	-4.789267	4.131599
36	1	0	2.047066	-5.015537	2.416496
37	6	0	-0.263563	4.250830	-1.010377
38	1	0	-1.206836	4.406679	-1.561626
39	1	0	0.408600	3.719905	-1.704433
40	6	0	0.338588	5.594306	-0.612942
41	1	0	-0.329903	6.154598	0.060988
42	1	0	1.300656	5.463053	-0.090949
43	1	0	0.522527	6.223116	-1.497533
44	1	0	-4.792245	0.300173	-1.019770
45	79	0	1.689311	0.543662	-0.245131
46	15	0	4.015458	0.227522	-0.119517
47	6	0	4.733946	-0.545022	-1.615464
48	6	0	4.967996	1.775293	0.099641
49	6	0	4.577657	-0.846071	1.255154
50	1	0	5.824237	-0.666188	-1.518824
51	1	0	4.273469	-1.530967	-1.778801
52	1	0	4.516215	0.083171	-2.492198
53	1	0	4.656606	2.271047	1.031341
54	1	0	6.050489	1.577084	0.140036
55	1	0	4.757183	2.456668	-0.738240
56	1	0	5.671655	-0.971977	1.239799
57	1	0	4.284739	-0.397255	2.215981
58	1	0	4.101582	-1.833867	1.167496

INT3a

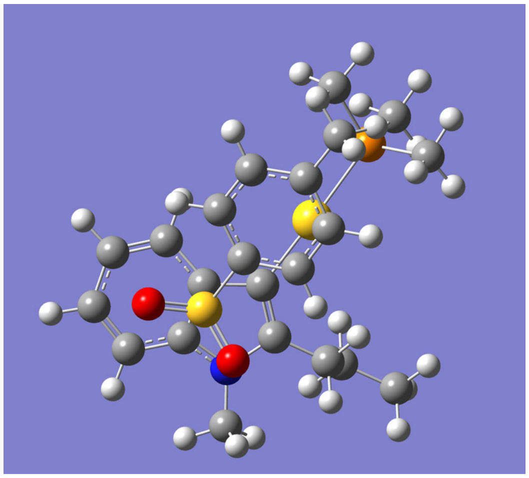

Center Number	Atomic Number	Atomic type	X	Y	Z
1	6	0	4.020508	-1.756584	-2.148563
2	6	0	4.366372	-0.824152	-1.195274
3	6	0	3.318967	-0.070016	-0.624583
4	6	0	1.914341	-0.374988	-0.917885
5	6	0	1.630987	-1.252686	-2.025844
6	6	0	2.664684	-1.946798	-2.587863
7	1	0	4.804749	-2.360815	-2.608977
8	1	0	5.407909	-0.667921	-0.914581
9	1	0	0.598275	-1.378757	-2.355046
10	1	0	2.474864	-2.659741	-3.392177
11	6	0	1.095228	0.652345	-0.258432
12	6	0	1.981816	1.404869	0.464389
13	6	0	1.719196	2.577146	1.351183
14	1	0	0.678793	2.494194	1.699836
15	1	0	2.350955	2.527526	2.254209
16	7	0	3.321087	0.954801	0.239082
17	6	0	4.482577	1.426406	0.970825
18	1	0	4.487672	1.000174	1.985946
19	1	0	4.472865	2.521202	1.034621
20	1	0	5.397990	1.120278	0.450812
21	16	0	1.885520	-1.898452	0.779677
22	8	0	2.515429	-3.096561	0.222904
23	8	0	2.429316	-1.178698	1.935974
24	79	0	-0.944503	0.841996	-0.426459
25	15	0	-3.272834	1.021322	-0.665240
26	6	0	-4.174914	1.209597	0.917945
27	6	0	-3.834455	2.441655	-1.673121

28	6	0	-4.037881	-0.435839	-1.467548
29	1	0	-5.264301	1.234205	0.759735
30	1	0	-3.861561	2.144347	1.406639
31	1	0	-3.920458	0.376533	1.587664
32	1	0	-4.932538	2.467179	-1.752626
33	1	0	-3.400284	2.369646	-2.681459
34	1	0	-3.482820	3.378634	-1.216160
35	1	0	-3.652177	-0.529492	-2.493957
36	1	0	-5.134639	-0.343840	-1.503012
37	1	0	-3.763231	-1.346830	-0.916972
38	6	0	1.904912	3.938571	0.655199
39	1	0	2.930227	4.017956	0.253608
40	1	0	1.237455	3.971421	-0.222051
41	6	0	1.619060	5.113429	1.584644
42	1	0	2.299899	5.117980	2.451791
43	1	0	0.588801	5.072952	1.975321
44	1	0	1.739546	6.075104	1.062772
45	6	0	0.149286	-2.183371	0.997462
46	6	0	-0.524486	-1.494638	2.009690
47	6	0	-0.495672	-3.085844	0.150740
48	6	0	-1.882867	-1.740970	2.180524
49	1	0	0.012994	-0.796530	2.650795
50	6	0	-1.861965	-3.299277	0.330815
51	1	0	0.062582	-3.620859	-0.617974
52	6	0	-2.574328	-2.639259	1.346179
53	1	0	-2.417496	-1.231677	2.985944
54	1	0	-2.380434	-4.009352	-0.316970
55	6	0	-4.042137	-2.890641	1.557720
56	1	0	-4.461389	-3.562275	0.795541
57	1	0	-4.617258	-1.951172	1.539701
58	1	0	-4.218600	-3.351388	2.543635

TS3-3a

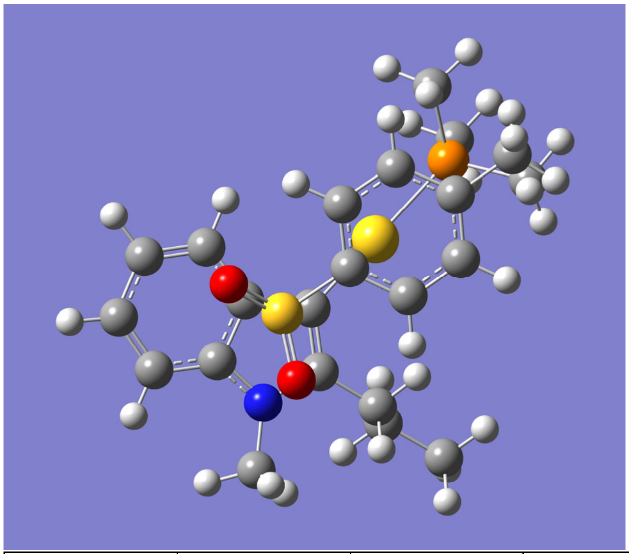

Center Number	Atomic Number	Atomic type	X	Y	Z
1	6	0	-4.410978	-1.402239	-2.165759
2	6	0	-4.576216	-0.502531	-1.132008
3	6	0	-3.413609	0.113187	-0.624842
4	6	0	-2.097075	-0.233116	-1.104632
5	6	0	-1.979044	-1.088390	-2.223180
6	6	0	-3.123933	-1.681418	-2.715349
7	1	0	-5.283717	-1.907929	-2.582504
8	1	0	-0.996120	-1.303806	-2.645716
9	1	0	-3.053975	-2.381762	-3.550081
10	6	0	-1.119980	0.573143	-0.339390
11	6	0	-1.885325	1.316181	0.544380
12	6	0	-1.422339	2.305299	1.564305
13	1	0	-0.385624	2.041287	1.823916
14	1	0	-2.008009	2.207168	2.493821
15	7	0	-3.246024	1.032622	0.362461
16	6	0	-4.316646	1.525404	1.211374
17	1	0	-4.135026	2.572869	1.478656
18	1	0	-5.270539	1.467830	0.673737
19	1	0	-4.379844	0.917496	2.127160
20	16	0	-1.867380	-1.826381	0.766467
21	8	0	-2.359097	-3.002281	0.045103
22	8	0	-2.597219	-1.211209	1.883028
23	6	0	-0.157684	-2.059093	1.174576
24	6	0	0.598717	-2.940504	0.397900
25	6	0	0.392245	-1.322996	2.225195
26	6	0	1.949035	-3.088516	0.701588

27	1	0	0.133986	-3.503390	-0.411890
28	6	0	1.743069	-1.497179	2.513089
29	1	0	-0.227723	-0.637840	2.803199
30	6	0	2.542871	-2.374160	1.758608
31	1	0	2.551956	-3.788257	0.117449
32	1	0	2.185336	-0.945645	3.345672
33	6	0	4.000061	-2.564743	2.075970
34	1	0	4.345609	-1.875539	2.858904
35	1	0	4.189316	-3.591649	2.430322
36	1	0	4.630087	-2.419493	1.183532
37	6	0	-1.450989	3.764903	1.074813
38	1	0	-2.476717	4.037332	0.770782
39	1	0	-0.839869	3.833315	0.159229
40	6	0	-0.942734	4.745494	2.126239
41	1	0	-1.559620	4.715371	3.039567
42	1	0	0.094324	4.514468	2.421220
43	1	0	-0.958526	5.779607	1.749570
44	1	0	-5.565859	-0.280544	-0.732082
45	79	0	0.913419	0.653165	-0.619875
46	15	0	3.236315	0.763735	-0.955125
47	6	0	3.768650	2.105609	-2.079303
48	6	0	4.198399	1.046756	0.576273
49	6	0	3.956632	-0.753715	-1.682135
50	1	0	4.862847	2.112093	-2.203460
51	1	0	3.293239	1.972183	-3.062539
52	1	0	3.444972	3.075142	-1.672204
53	1	0	3.982232	0.248659	1.299934
54	1	0	5.280063	1.069714	0.371141
55	1	0	3.897014	2.005753	1.023638
56	1	0	5.049945	-0.670080	-1.782801
57	1	0	3.710897	-1.616693	-1.047410
58	1	0	3.516544	-0.926280	-2.675751

INT4

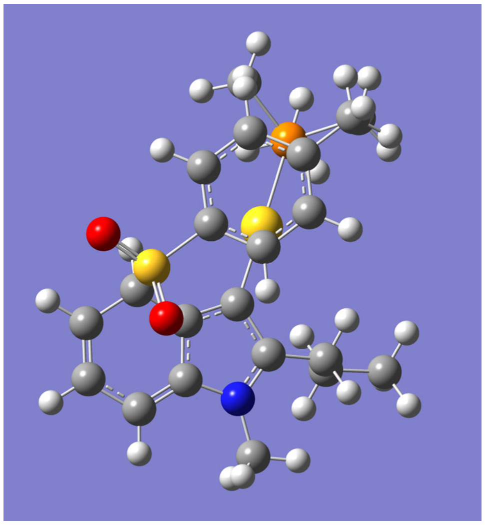

Center Number	Atomic Number	Atomic type	X	Y	Z
1	6	0	4.398863	-1.624477	-1.642591
2	6	0	4.504772	-0.601800	-0.634819
3	6	0	3.362551	0.003538	-0.190035
4	6	0	2.053271	-0.341170	-0.681995
5	6	0	1.930021	-1.531044	-1.527532
6	6	0	3.189287	-2.031794	-2.115662
7	1	0	5.316292	-2.066694	-2.034033
8	1	0	5.491607	-0.307494	-0.271249
9	1	0	1.057842	-1.553167	-2.192784
10	1	0	3.122685	-2.828811	-2.857546
11	6	0	1.115610	0.532053	-0.139296
12	6	0	1.877196	1.400208	0.718069
13	6	0	1.342380	2.559542	1.481843
14	1	0	0.294994	2.332696	1.731271
15	1	0	1.880734	2.690678	2.434021
16	7	0	3.198140	1.077179	0.686209
17	6	0	4.300207	1.748292	1.354156
18	1	0	3.913537	2.433938	2.115470
19	1	0	4.901530	2.324963	0.634317
20	1	0	4.943534	1.009169	1.852416
21	16	0	1.500440	-3.054067	-0.312346
22	8	0	1.303466	-4.184361	-1.225296
23	8	0	2.539851	-3.045008	0.720617
24	79	0	-0.888936	0.715791	-0.579294
25	15	0	-3.133723	1.052768	-1.189780
26	6	0	-4.282090	1.336547	0.206958

27	6	0	-3.353998	2.521998	-2.259691
28	6	0	-3.894370	-0.313047	-2.140004
29	1	0	-5.306470	1.522977	-0.151548
30	1	0	-3.942510	2.204514	0.791807
31	1	0	-4.285120	0.458068	0.867182
32	1	0	-4.411189	2.660873	-2.534790
33	1	0	-2.754764	2.403413	-3.174888
34	1	0	-2.997684	3.418923	-1.731381
35	1	0	-3.307780	-0.497048	-3.052414
36	1	0	-4.931696	-0.073198	-2.420579
37	1	0	-3.884225	-1.230157	-1.534856
38	6	0	-0.056103	-2.638006	0.438661
39	6	0	-1.236941	-3.006697	-0.208832
40	6	0	-0.065929	-2.008396	1.685988
41	6	0	-2.451990	-2.745112	0.422105
42	1	0	-1.197614	-3.521980	-1.169643
43	6	0	-1.291907	-1.744718	2.291040
44	1	0	0.874643	-1.754180	2.173623
45	6	0	-2.500657	-2.116002	1.677562
46	1	0	-3.381534	-3.065011	-0.054929
47	1	0	-1.312455	-1.257469	3.268715
48	6	0	-3.812543	-1.888645	2.377373
49	1	0	-3.994162	-2.684593	3.120051
50	1	0	-3.822311	-0.935190	2.926861
51	1	0	-4.661275	-1.903300	1.678301
52	6	0	1.392077	3.873514	0.671387
53	1	0	2.438954	4.098898	0.405431
54	1	0	0.859934	3.715340	-0.281241
55	6	0	0.779588	5.044139	1.432584
56	1	0	-0.277497	4.852330	1.679120
57	1	0	1.311320	5.235326	2.378884
58	1	0	0.820744	5.967871	0.835530

TS3a-4

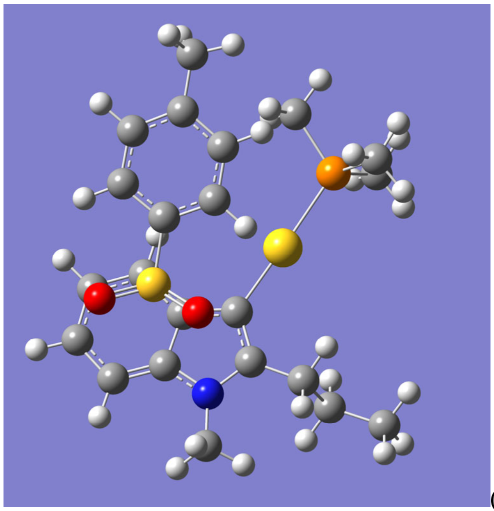

(Mirror-inverted image)

Center Number	Atomic Number	Atomic type	X	Y	Z
1	6	0	-3.649349	-1.584876	-1.377285
2	6	0	-3.770283	-0.222740	-1.041160
3	6	0	-2.608783	0.480914	-0.772332
4	6	0	-1.296081	-0.138927	-0.844091
5	6	0	-1.237720	-1.544202	-1.123261
6	6	0	-2.405749	-2.235091	-1.428545
7	1	0	-4.557000	-2.155478	-1.583037
8	1	0	-0.263941	-2.038262	-1.140320
9	1	0	-2.365055	-3.299336	-1.664995
10	6	0	-0.318217	0.817328	-0.502884
11	6	0	-1.050192	1.982619	-0.190777
12	6	0	-0.503650	3.295629	0.246003
13	1	0	0.457888	3.103083	0.744484
14	1	0	-1.164257	3.762228	0.994708
15	7	0	-2.409797	1.788795	-0.388444
16	6	0	-3.480437	2.715778	-0.076602
17	1	0	-3.119697	3.748257	-0.143668
18	1	0	-4.296030	2.600255	-0.803876
19	1	0	-3.874766	2.538400	0.937531
20	16	0	-1.782655	-1.204201	1.544960
21	8	0	-3.059583	-1.932635	1.561464
22	8	0	-1.593145	0.116933	2.164743
23	6	0	-0.406953	-2.245450	1.929931
24	6	0	-0.500938	-3.620167	1.680064
25	6	0	0.765990	-1.646052	2.397449
26	6	0	0.615288	-4.409145	1.925756
27	1	0	-1.433293	-4.053471	1.315707

28	6	0	1.867566	-2.466433	2.641194
29	1	0	0.799119	-0.573584	2.584920
30	6	0	1.817570	-3.849078	2.405578
31	1	0	0.556729	-5.486186	1.751814
32	1	0	2.783879	-2.021918	3.035763
33	6	0	3.010517	-4.726855	2.658249
34	1	0	3.874901	-4.152577	3.019624
35	1	0	2.777852	-5.497092	3.411849
36	1	0	3.310183	-5.260523	1.741296
37	6	0	-0.272693	4.269759	-0.929601
38	1	0	-1.225389	4.443929	-1.458606
39	1	0	0.393910	3.781003	-1.658945
40	6	0	0.322642	5.597477	-0.473215
41	1	0	-0.341489	6.116666	0.237093
42	1	0	1.293229	5.450006	0.028177
43	1	0	0.488052	6.271776	-1.327522
44	1	0	-4.755417	0.240171	-0.973431
45	79	0	1.699770	0.481315	-0.317876
46	15	0	4.000406	0.033953	-0.123177
47	6	0	4.414185	-1.745582	-0.257710
48	6	0	5.042403	0.838959	-1.394626
49	6	0	4.748115	0.554856	1.465880
50	1	0	5.496145	-1.915731	-0.142838
51	1	0	3.872258	-2.307927	0.516880
52	1	0	4.093477	-2.122116	-1.240652
53	1	0	4.922278	1.930812	-1.329740
54	1	0	6.105373	0.583471	-1.262229
55	1	0	4.714245	0.517961	-2.394536
56	1	0	5.820225	0.306473	1.505279
57	1	0	4.625686	1.641252	1.590164
58	1	0	4.232139	0.057670	2.300649

TS7a-7

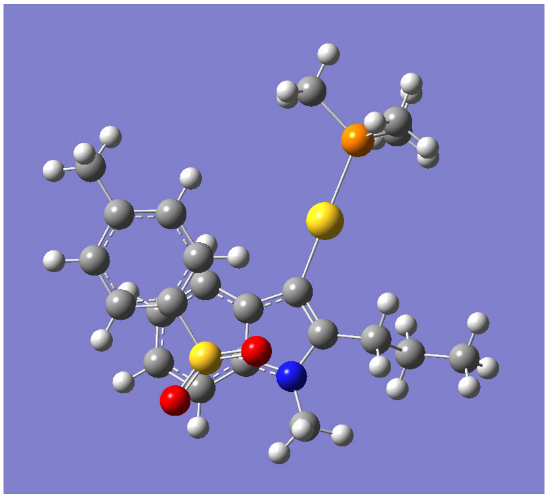

1	6	0	-3.564003	0.584241	2.244543
2	6	0	-3.182223	1.715076	1.521216
3	6	0	-1.847690	1.764324	1.006156
4	6	0	-0.951677	0.625989	1.127382
5	6	0	-1.388973	-0.483302	1.848025
6	6	0	-2.678052	-0.493458	2.406982
7	1	0	-4.568763	0.535395	2.666200
8	1	0	-3.865460	2.556925	1.401769
9	1	0	-0.729130	-1.344722	1.967255
10	1	0	-3.007005	-1.368722	2.970646
11	6	0	0.258031	0.939126	0.416033
12	6	0	0.076664	2.222413	-0.065850
13	6	0	1.019671	3.076133	-0.842540
14	1	0	1.685578	2.403128	-1.403311
15	1	0	0.473969	3.671849	-1.593375
16	7	0	-1.192145	2.733730	0.335860
17	6	0	-1.755265	4.010426	-0.060507
18	1	0	-2.167268	3.963094	-1.080834
19	1	0	-0.986901	4.792327	-0.020137
20	1	0	-2.554759	4.293952	0.636475
21	16	0	-3.354878	0.877870	-0.996203
22	8	0	-4.735748	1.360011	-1.023439
23	8	0	-2.312524	1.408455	-1.878060
24	79	0	1.914443	-0.254983	0.176782
25	15	0	3.835658	-1.589259	-0.082029
26	6	0	4.538923	-1.582905	-1.773091
27	6	0	5.233442	-1.087693	0.989383
28	6	0	3.593433	-3.364939	0.298404
29	1	0	5.442981	-2.209018	-1.833088

30	1	0	4.794853	-0.551946	-2.060216
31	1	0	3.790044	-1.959936	-2.485674
32	1	0	6.109316	-1.737545	0.835963
33	1	0	4.924956	-1.138800	2.044332
34	1	0	5.512006	-0.047091	0.765192
35	1	0	3.249742	-3.474764	1.337973
36	1	0	4.527395	-3.933323	0.165996
37	1	0	2.821710	-3.782038	-0.365924
38	6	0	1.878412	4.006380	0.037783
39	1	0	1.224327	4.660197	0.640572
40	1	0	2.429434	3.383773	0.762026
41	6	0	2.847358	4.852976	-0.780942
42	1	0	2.313653	5.504617	-1.492351
43	1	0	3.535227	4.220431	-1.365971
44	1	0	3.459252	5.499276	-0.132972
45	6	0	-3.249490	-0.861233	-0.805894
46	6	0	-2.044811	-1.501212	-1.128179
47	6	0	-4.353593	-1.548325	-0.287198
48	6	0	-1.979219	-2.878440	-0.973849
49	1	0	-1.189134	-0.926169	-1.483716
50	6	0	-4.254777	-2.928683	-0.139779
51	1	0	-5.268414	-1.013433	-0.031641
52	6	0	-3.076650	-3.615603	-0.482499
53	1	0	-1.055076	-3.398679	-1.235091
54	1	0	-5.110257	-3.485392	0.248299
55	6	0	-2.992966	-5.108315	-0.353413
56	1	0	-1.981406	-5.436278	-0.071759
57	1	0	-3.229551	-5.585239	-1.320900
58	1	0	-3.707253	-5.496903	0.386165

INT7

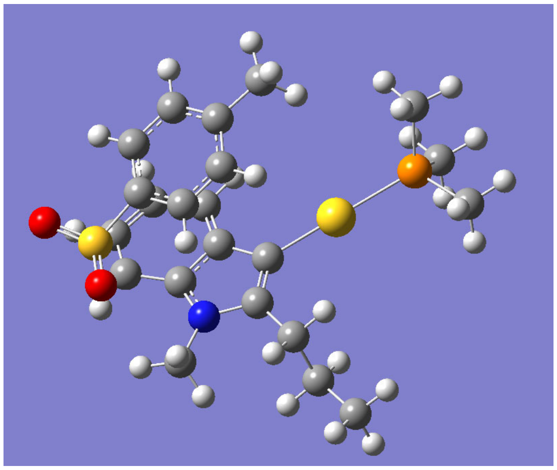

1	6	0	-3.593883	-0.452958	2.331950
2	6	0	-3.589334	0.620289	1.323385
3	6	0	-2.261433	1.054141	0.902342
4	6	0	-1.093050	0.247132	1.158660
5	6	0	-1.206484	-0.825893	2.012805
6	6	0	-2.463494	-1.152521	2.628598
7	1	0	-4.556742	-0.728656	2.764000
8	1	0	-4.351673	1.396635	1.473801
9	1	0	-0.325489	-1.435693	2.228493
10	1	0	-2.506073	-1.983333	3.334819
11	6	0	0.023584	0.816837	0.425113
12	6	0	-0.489445	1.931800	-0.195271
13	6	0	0.204180	2.966028	-1.021071
14	1	0	1.045042	2.466884	-1.526785
15	1	0	-0.456545	3.340437	-1.820753
16	7	0	-1.885670	2.062079	0.112049
17	6	0	-2.731533	3.134610	-0.388518
18	1	0	-3.710874	3.097716	0.099317
19	1	0	-2.893137	3.025834	-1.469648
20	1	0	-2.257837	4.103558	-0.182690
21	16	0	-4.413766	-0.225022	-0.310505
22	8	0	-5.572431	-0.968559	0.178530
23	8	0	-4.536253	0.900518	-1.241814
24	79	0	1.927297	0.054182	0.284334
25	15	0	4.112847	-0.803253	0.111203
26	6	0	5.248912	0.275789	-0.835300
27	6	0	4.947130	-1.044319	1.722469
28	6	0	4.244313	-2.433530	-0.714370
29	1	0	6.261449	-0.154696	-0.882599
30	1	0	5.299755	1.264869	-0.355788

31	1	0	4.865473	0.409414	-1.858000
32	1	0	5.969162	-1.432034	1.588772
33	1	0	4.369676	-1.752550	2.335345
34	1	0	4.991914	-0.084725	2.259032
35	1	0	3.664524	-3.180796	-0.152554
36	1	0	5.293063	-2.765131	-0.770318
37	1	0	3.836258	-2.366761	-1.733763
38	6	0	0.751904	4.150801	-0.202738
39	1	0	-0.071810	4.638290	0.347494
40	1	0	1.431328	3.750570	0.568193
41	6	0	1.475108	5.176892	-1.068517
42	1	0	0.803644	5.612271	-1.826900
43	1	0	2.325029	4.721452	-1.603192
44	1	0	1.870131	6.005186	-0.460536
45	6	0	-3.124566	-1.312728	-0.854464
46	6	0	-2.162107	-0.814451	-1.739092
47	6	0	-3.021915	-2.587453	-0.294815
48	6	0	-1.054171	-1.599808	-2.028518
49	1	0	-2.282033	0.174450	-2.179825
50	6	0	-1.900199	-3.359029	-0.602147
51	1	0	-3.799769	-2.960660	0.371787
52	6	0	-0.892784	-2.871977	-1.449706
53	1	0	-0.290060	-1.215237	-2.707552
54	1	0	-1.803226	-4.356302	-0.167480
55	6	0	0.349773	-3.670235	-1.728105
56	1	0	0.523247	-3.775260	-2.810792
57	1	0	0.301194	-4.676799	-1.290115
58	1	0	1.231603	-3.156890	-1.308357

TS7-6

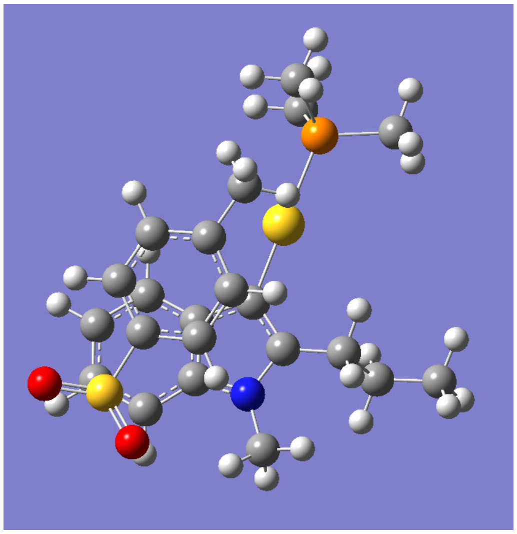

1	6	0	3.591857	-0.487544	-2.367435
2	6	0	3.475616	0.709084	-1.592108
3	6	0	2.222317	0.998199	-1.043283
4	6	0	1.100116	0.127384	-1.205281
5	6	0	1.267650	-1.049479	-1.950767
6	6	0	2.510931	-1.353095	-2.527629
7	1	0	4.551326	-0.719494	-2.832601
8	1	0	4.303441	1.418654	-1.557739
9	1	0	0.420818	-1.728632	-2.076201
10	1	0	2.636042	-2.268380	-3.107674
11	6	0	-0.013237	0.682239	-0.491840
12	6	0	0.474707	1.869001	0.077539
13	6	0	-0.277465	2.873770	0.887085
14	1	0	-1.155865	2.360678	1.306732
15	1	0	0.323614	3.213110	1.748871
16	7	0	1.813063	2.043794	-0.249673
17	6	0	2.675895	3.146616	0.135058
18	1	0	3.669205	2.763774	0.408339
19	1	0	2.256252	3.665677	1.003912
20	1	0	2.784603	3.870944	-0.688086
21	16	0	4.659775	-0.705994	0.211373
22	8	0	5.432786	-1.849196	-0.278789
23	8	0	5.272745	0.469060	0.843012
24	79	0	-1.914102	-0.075748	-0.331757
25	15	0	-4.110345	-0.912389	-0.172745
26	6	0	-5.290095	0.258270	0.595243
27	6	0	-4.863766	-1.323254	-1.789659
28	6	0	-4.291495	-2.445428	0.814123

29	1	0	-6.304638	-0.167786	0.639027
30	1	0	-5.315358	1.189703	0.009958
31	1	0	-4.957309	0.502828	1.615184
32	1	0	-5.893309	-1.694735	-1.668340
33	1	0	-4.259525	-2.091908	-2.294325
34	1	0	-4.875519	-0.425480	-2.425738
35	1	0	-3.669975	-3.241694	0.378097
36	1	0	-5.339881	-2.782206	0.834973
37	1	0	-3.955288	-2.264270	1.845640
38	6	0	-0.749029	4.095410	0.076372
39	1	0	0.123352	4.601877	-0.371419
40	1	0	-1.357515	3.735157	-0.769821
41	6	0	-1.546681	5.084690	0.919486
42	1	0	-0.944983	5.483632	1.752857
43	1	0	-2.441118	4.609936	1.355709
44	1	0	-1.885799	5.940562	0.316056
45	6	0	3.177627	-1.176267	1.016391
46	6	0	2.557826	-0.250713	1.868325
47	6	0	2.565456	-2.383518	0.650438
48	6	0	1.296211	-0.551888	2.355713
49	1	0	3.068654	0.671862	2.141979
50	6	0	1.306405	-2.666336	1.172357
51	1	0	3.074298	-3.077370	-0.018409
52	6	0	0.645062	-1.754182	2.010432
53	1	0	0.798339	0.153650	3.024051
54	1	0	0.818547	-3.606634	0.907260
55	6	0	-0.716765	-2.064005	2.560609
56	1	0	-0.628880	-2.539723	3.553101
57	1	0	-1.266665	-2.757450	1.909362
58	1	0	-1.314563	-1.149653	2.684206

INT6

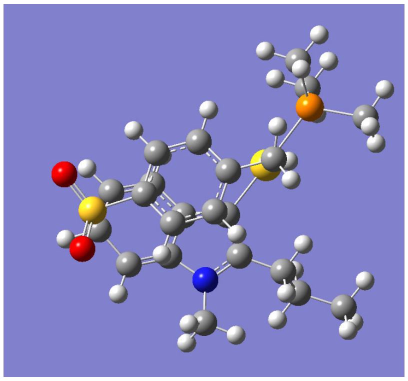

1	6	0	3.819046	-0.184997	-1.752802
2	6	0	3.470412	1.140519	-1.230377
3	6	0	2.170032	1.358592	-0.918738
4	6	0	1.113774	0.390236	-1.180004
5	6	0	1.435857	-0.788675	-1.931276
6	6	0	2.732364	-1.046731	-2.246094
7	1	0	4.746433	-0.220547	-2.340422
8	1	0	4.272120	1.836884	-0.980241
9	1	0	0.632453	-1.466279	-2.225679
10	1	0	3.015892	-1.952962	-2.782968
11	6	0	-0.059458	0.835052	-0.572806
12	6	0	0.281458	2.096722	0.019873
13	6	0	-0.651944	3.001413	0.742782
14	1	0	-1.420521	2.372145	1.215312
15	1	0	-0.135808	3.544158	1.550241
16	7	0	1.585207	2.411268	-0.208061
17	6	0	2.319506	3.588079	0.219377
18	1	0	3.147724	3.304156	0.886864
19	1	0	1.655410	4.269965	0.759855
20	1	0	2.729450	4.120491	-0.651713
21	16	0	4.550815	-1.133132	-0.131752
22	8	0	4.851309	-2.487023	-0.598496
23	8	0	5.579041	-0.229151	0.391564
24	79	0	-1.851428	-0.162573	-0.402783
25	15	0	-3.870693	-1.348132	-0.175784
26	6	0	-5.105139	-0.518388	0.890663
27	6	0	-4.749532	-1.649277	-1.751757
28	6	0	-3.669283	-3.010643	0.564785
29	1	0	-6.029626	-1.112038	0.965489
30	1	0	-5.345549	0.471221	0.473971
31	1	0	-4.686821	-0.375467	1.898316
32	1	0	-5.685270	-2.205441	-1.585134

33	1	0	-4.102711	-2.225517	-2.430013
34	1	0	-4.981068	-0.687481	-2.233310
35	1	0	-2.998048	-3.615444	-0.063448
36	1	0	-4.637940	-3.526529	0.656220
37	1	0	-3.216661	-2.917127	1.563392
38	6	0	-1.342059	4.007482	-0.205696
39	1	0	-0.574868	4.625997	-0.701940
40	1	0	-1.845718	3.442021	-1.006656
41	6	0	-2.341862	4.895289	0.527159
42	1	0	-1.853415	5.492809	1.314019
43	1	0	-3.133227	4.296642	1.007007
44	1	0	-2.829268	5.595543	-0.168007
45	6	0	3.145848	-1.149787	0.957254
46	6	0	2.945017	-0.069069	1.820260
47	6	0	2.210234	-2.182614	0.849268
48	6	0	1.766578	-0.010965	2.560091
49	1	0	3.711667	0.701386	1.911415
50	6	0	1.039521	-2.105595	1.598822
51	1	0	2.403781	-3.025888	0.185964
52	6	0	0.788341	-1.013897	2.446905
53	1	0	1.603314	0.825462	3.243851
54	1	0	0.300475	-2.906103	1.519666
55	6	0	-0.514403	-0.910973	3.188713
56	1	0	-0.447926	-0.226192	4.046055
57	1	0	-0.849026	-1.892347	3.557568
58	1	0	-1.300141	-0.528251	2.512489

7.3 Calculations for the formation of dinuclear Au-Ag complex

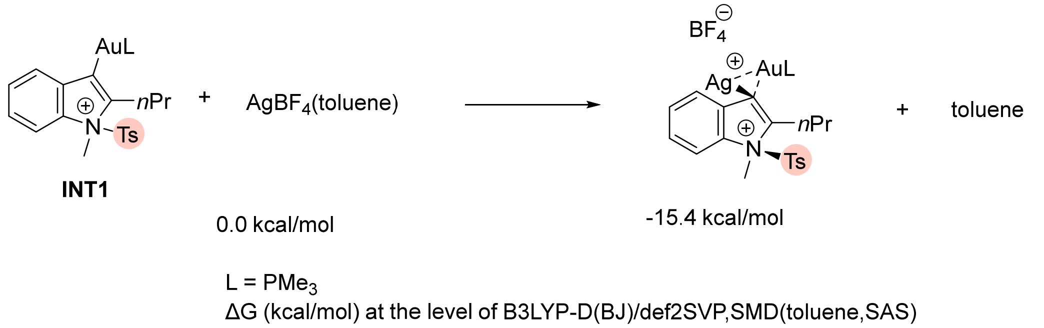

Scheme S3. DFT calculations for the formation of dinuclear Au-Ag complex

Table S7. Calculated energies of intermediates and transition states

Compound	SCF energy / a.u.	E(ZPVE) / a.u.	H / a.u.	G(298) / a.u.
**INT1**	-1935.912769	-1935.436077	-1935.403120	-1935.501159
**$AgBF_4$(toluene)**	-842.576123	-842.431889	-842.416460	-842.479192
**INT1·$AgBF_4$**	-2507.116109	-2506.622827	-2506.581766	-2506.698506
**Toluene**	-271.403479	-271.275619	-271.268479	-271.306429

$AgBF_4$(toluene)

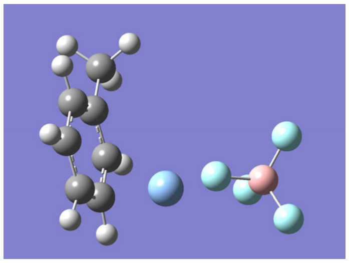

1	47	0	0.284574	-0.543439	-0.166159
2	5	0	2.933990	0.563699	0.130953
3	9	0	4.056331	-0.076571	-0.296925
4	9	0	1.996175	0.709712	-0.981833
5	9	0	3.141545	1.776195	0.715008
6	9	0	2.192383	-0.301681	1.047111
7	6	0	-1.781997	-1.649272	0.201453
8	6	0	-2.287818	-0.993033	1.346710
9	6	0	-2.887354	0.254362	1.228162
10	6	0	-3.003496	0.902168	-0.021569
11	6	0	-2.500888	0.256425	-1.154235
12	6	0	-1.894720	-1.013979	-1.058949
13	1	0	-1.427757	-2.680949	0.271466
14	1	0	-2.218698	-1.480103	2.321359
15	1	0	-3.279684	0.747887	2.120858

16	1	0	-2.588815	0.731313	-2.133751
17	1	0	-1.612977	-1.548503	-1.969980
18	6	0	-3.640267	2.261678	-0.112229
19	1	0	-4.611891	2.282239	0.406350
20	1	0	-3.002544	3.023450	0.367250
21	1	0	-3.801228	2.568818	-1.155172

INT1·$AgBF_4$

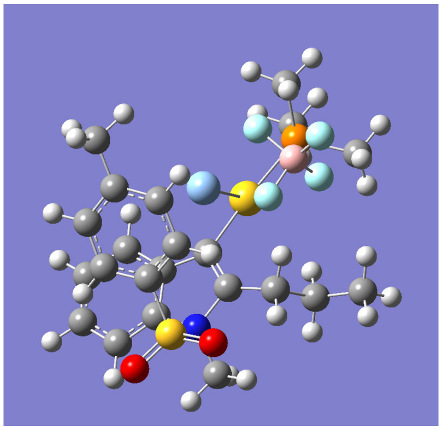

1	6	0	-3.768556	-2.530912	-0.899658
2	6	0	-2.629376	-1.958754	-0.350794
3	6	0	-1.528007	-1.781143	-2.490934
4	6	0	-2.673179	-2.339800	-3.072125
5	6	0	-3.774008	-2.714933	-2.290071
6	1	0	-4.630151	-2.811887	-0.295208
7	1	0	-0.663140	-1.507670	-3.098531
8	1	0	-2.706125	-2.498868	-4.151943
9	1	0	-4.648546	-3.163855	-2.764149
10	6	0	-1.505013	-1.592423	-1.108481
11	7	0	-2.376846	-1.599970	1.034696
12	6	0	-0.470936	-1.071604	-0.200003
13	6	0	-0.985434	-1.095303	1.070083
14	6	0	-2.741322	-2.616760	2.054117
15	1	0	-2.156231	-3.526449	1.864553
16	1	0	-2.522579	-2.223849	3.052613
17	1	0	-3.812705	-2.836696	1.973783
18	6	0	-0.327044	-0.751339	2.368800
19	1	0	0.236798	0.180022	2.219961
20	1	0	-1.077099	-0.529419	3.140242
21	6	0	0.662112	-1.818791	2.881076
22	1	0	0.127980	-2.753947	3.122675
23	1	0	1.356694	-2.064066	2.058728

24	6	0	1.446546	-1.316778	4.088065
25	1	0	0.778389	-1.062677	4.927119
26	1	0	2.154501	-2.079465	4.447272
27	1	0	2.017187	-0.412138	3.825979
28	16	0	-3.688777	-0.028339	1.457582
29	6	0	-3.186751	1.164028	0.275338
30	6	0	-3.675156	1.074055	-1.036231
31	6	0	-2.275966	2.153951	0.671178
32	6	0	-3.216204	1.989600	-1.976435
33	1	0	-4.390477	0.299321	-1.310845
34	6	0	-1.840723	3.074834	-0.292150
35	1	0	-1.910258	2.215110	1.696646
36	6	0	-2.296790	3.000810	-1.629905
37	1	0	-3.581763	1.925754	-3.003217
38	1	0	-1.165830	3.877247	0.015690
39	6	0	-1.841021	4.007717	-2.644329
40	1	0	-2.535910	4.865425	-2.658760
41	1	0	-0.842480	4.402109	-2.407469
42	1	0	-1.825279	3.584732	-3.659006
43	8	0	-4.965745	-0.637754	1.120836
44	8	0	-3.304142	0.336269	2.811202
45	79	0	1.599711	-0.984217	-0.550524
46	15	0	3.939079	-0.806503	-0.726375
47	6	0	4.767471	-2.040795	-1.790059
48	1	0	5.855797	-1.873570	-1.806998
49	1	0	4.565415	-3.053856	-1.411373
50	1	0	4.376798	-1.969834	-2.816095
51	6	0	4.441157	0.823476	-1.373317
52	1	0	3.977413	1.606362	-0.756678
53	1	0	5.537194	0.928429	-1.356616
54	1	0	4.081938	0.937042	-2.407052
55	6	0	4.735441	-0.944517	0.909102
56	1	0	5.826280	-0.821523	0.822323
57	1	0	4.320178	-0.160474	1.558329
58	1	0	4.515864	-1.928200	1.350384
59	47	0	0.064270	1.312016	-0.533846
60	5	0	1.944669	2.426420	1.422157
61	9	0	2.499594	1.180375	1.744741
62	9	0	2.008568	2.565596	-0.025295
63	9	0	2.554892	3.465602	2.034282
64	9	0	0.542144	2.375992	1.712331

toluene

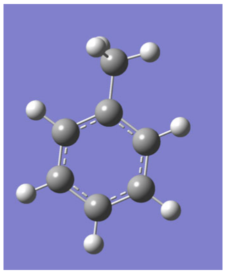

1	6	0	-1.906029	-0.006509	-0.000003
2	6	0	-1.194373	-1.210232	-0.000001
3	6	0	0.200641	-1.200223	0.000004
4	6	0	0.916271	0.008512	0.000005
5	6	0	0.189497	1.207098	0.000004
6	6	0	-1.208504	1.202371	-0.000001
7	1	0	-2.998673	-0.012790	-0.000006
8	1	0	-1.730656	-2.162670	-0.000001
9	1	0	0.748790	-2.147005	0.000007
10	1	0	0.726929	2.159455	0.000007
11	1	0	-1.753653	2.149697	-0.000001
12	6	0	2.423622	0.003404	-0.000004
13	1	0	2.823218	-0.518702	0.885656
14	1	0	2.823205	-0.518482	-0.885800
15	1	0	2.834086	1.023967	0.000117

7.4 Migration of tosyl group from INT3a-dinuclear Au-Ag complex

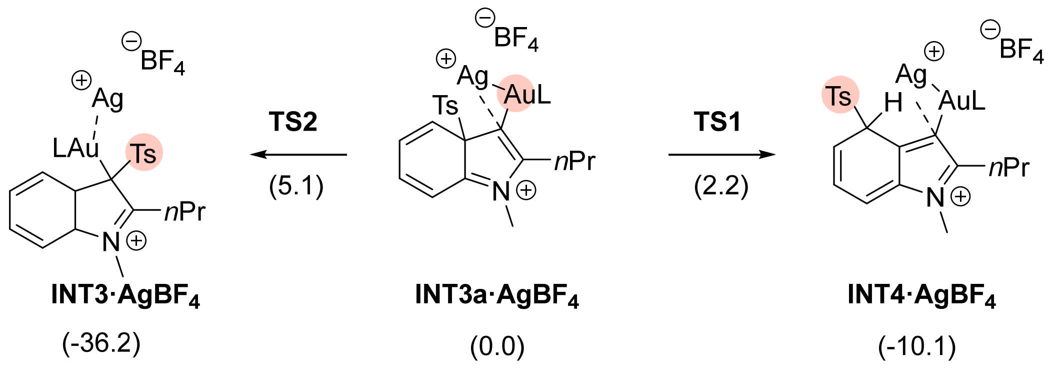

Scheme S4. DFT calculations for migration of tosyl group from INT3a-dinuclear Au-Ag complex

Table S8. Calculated energies of intermediates and transition states

Compound	SCF energy / a.u.	E(ZPVE) / a.u.	H / a.u.	G(298) / a.u.	Imaginary frequency /cm^{-1}
**INT1·AgBF$_4$**	-2507.109356	-2506.617039	-2506.575395	-2506.695879	
**TS1**	-2507.104015	-2506.613359	-2506.571879	-2506.692405	-105.58
**INT4·AgBF$_4$**	-2507.129566	-2506.635655	-2506.594684	-2506.711903	
**TS2**	-2507.104154	-2506.612262	-2506.571408	-2506.687829	-215.07
**INT3·AgBF$_4$**	-2507.171350	-2506.676024	-2506.635281	-2506.753531	-

INT 3a·AgBF$_4$

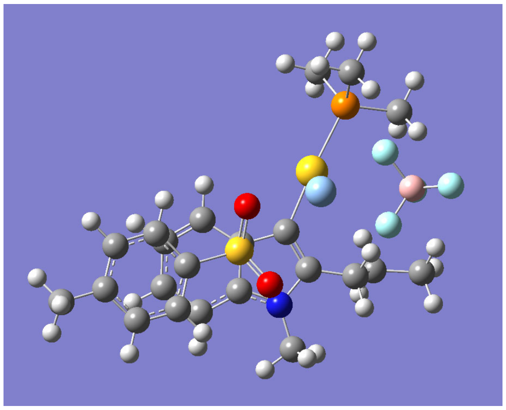

1	6	0	4.100771	0.486576	-2.435527
2	6	0	3.572019	1.656675	-1.937896
3	6	0	2.263922	1.599946	-1.405540
4	6	0	1.564568	0.313554	-1.233348
5	6	0	2.124710	-0.849927	-1.892932
6	6	0	3.370849	-0.755966	-2.439439
7	1	0	5.103701	0.500180	-2.867184
8	1	0	4.127698	2.592504	-1.998440
9	1	0	1.555744	-1.780965	-1.891810
10	1	0	3.833748	-1.628373	-2.903582
11	6	0	0.171639	0.626437	-0.861923
12	6	0	0.151746	1.984606	-0.659149
13	6	0	-0.989446	2.848673	-0.241446
14	1	0	-1.575022	2.295556	0.502901
15	1	0	-0.627394	3.754436	0.268491
16	7	0	1.425838	2.553940	-0.993357
17	6	0	1.753343	3.958929	-0.827811
18	1	0	1.778874	4.213071	0.242474
19	1	0	1.001456	4.582406	-1.329706
20	1	0	2.734309	4.167426	-1.269397
21	16	0	2.295578	-0.003655	0.829978
22	8	0	2.109948	1.280875	1.513290
23	8	0	1.525401	-1.196199	1.270520
24	79	0	-1.502507	-0.584004	-0.967971
25	15	0	-3.499401	-1.826904	-1.102674
26	6	0	-4.968354	-0.746879	-1.147093
27	6	0	-3.626378	-2.884084	-2.591545
28	6	0	-3.770159	-2.952901	0.307761
29	1	0	-5.892344	-1.344947	-1.178947
30	1	0	-4.922265	-0.100373	-2.036347
31	1	0	-4.957619	-0.107124	-0.253171

32	1	0	-4.590180	-3.416555	-2.613085
33	1	0	-2.809121	-3.620917	-2.596420
34	1	0	-3.539053	-2.263537	-3.496010
35	1	0	-2.936291	-3.667357	0.378386
36	1	0	-4.713106	-3.508245	0.184878
37	1	0	-3.805640	-2.355368	1.229582
38	6	0	-1.923609	3.235822	-1.405451
39	1	0	-1.372186	3.829375	-2.156501
40	1	0	-2.233401	2.307740	-1.915742
41	6	0	-3.152658	3.992701	-0.913711
42	1	0	-2.873196	4.929881	-0.404708
43	1	0	-3.721020	3.377319	-0.199637
44	1	0	-3.818311	4.255350	-1.750451
45	6	0	3.999891	-0.438226	0.743339
46	6	0	4.356634	-1.775643	0.534973
47	6	0	4.956928	0.571674	0.898632
48	6	0	5.709002	-2.099753	0.499293
49	1	0	3.591156	-2.543872	0.424888
50	6	0	6.301136	0.218851	0.862988
51	1	0	4.649604	1.603585	1.068759
52	6	0	6.701449	-1.115872	0.662727
53	1	0	6.002637	-3.140788	0.349359
54	1	0	7.058939	0.993168	1.001427
55	6	0	8.156711	-1.483513	0.661654
56	1	0	8.776397	-0.688594	0.221306
57	1	0	8.342570	-2.418190	0.113933
58	1	0	8.510000	-1.633441	1.696877
59	47	0	-0.688873	-0.500042	1.758740
60	5	0	-3.223713	0.792074	2.309285
61	9	0	-4.110229	1.143921	3.266652
62	9	0	-1.923567	1.362078	2.543600
63	9	0	-3.625091	1.140508	1.015903
64	9	0	-2.978585	-0.640777	2.319679

TS1

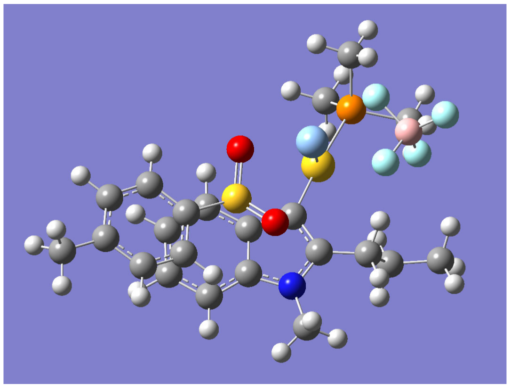

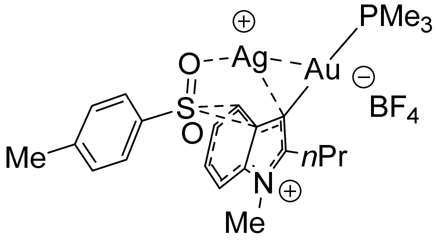

$TS_{3a\text{-}4\text{-}Ag}$

2.2

1	6	0	3.878211	0.564688	-2.427678
2	6	0	3.340458	1.740469	-1.877066
3	6	0	2.056064	1.678930	-1.356229
4	6	0	1.258975	0.461044	-1.401056
5	6	0	1.891748	-0.727632	-1.897299
6	6	0	3.170442	-0.653761	-2.430795
7	1	0	4.887488	0.594146	-2.843269
8	1	0	3.927007	2.659568	-1.863394
9	1	0	1.337614	-1.668333	-1.877382
10	1	0	3.645131	-1.548420	-2.835050
11	6	0	-0.020641	0.732173	-0.880235
12	6	0	0.050522	2.093980	-0.440987
13	6	0	-1.021237	2.850821	0.244777
14	1	0	-1.463099	2.186355	1.001160
15	1	0	-0.613903	3.721482	0.777596
16	7	0	1.269711	2.650616	-0.768521
17	6	0	1.724038	3.989334	-0.433485
18	1	0	0.896205	4.703093	-0.519238
19	1	0	2.504063	4.298223	-1.140826
20	1	0	2.128068	4.021788	0.590786
21	16	0	2.569953	-0.282723	0.829797
22	8	0	2.250193	0.944045	1.588598
23	8	0	1.859692	-1.544590	1.208543
24	79	0	-1.781534	-0.354736	-1.038657
25	15	0	-3.849185	-1.475206	-1.150532
26	6	0	-3.994400	-2.842558	0.048889
27	6	0	-5.239722	-0.355931	-0.773272
28	6	0	-4.245400	-2.213668	-2.777489
29	1	0	-4.989661	-3.309934	-0.011192
30	1	0	-3.826707	-2.439881	1.057955
31	1	0	-3.224968	-3.601207	-0.159431
32	1	0	-6.197545	-0.898550	-0.799072

33	1	0	-5.265247	0.461874	-1.509080
34	1	0	-5.073895	0.077031	0.223496
35	1	0	-4.246735	-1.430591	-3.550555
36	1	0	-5.233189	-2.700302	-2.757016
37	1	0	-3.482568	-2.960737	-3.043452
38	6	0	-2.154061	3.300099	-0.712652
39	1	0	-1.748796	3.996129	-1.467089
40	1	0	-2.512166	2.415048	-1.264712
41	6	0	-3.308245	3.934733	0.054894
42	1	0	-2.980074	4.823973	0.617325
43	1	0	-3.734004	3.215836	0.770725
44	1	0	-4.106124	4.253313	-0.633985
45	6	0	4.301110	-0.548924	0.678005
46	6	0	4.765121	-1.826413	0.329738
47	6	0	5.160782	0.550879	0.799214
48	6	0	6.130966	-2.004062	0.162809
49	1	0	4.069449	-2.659604	0.224152
50	6	0	6.525902	0.341700	0.624206
51	1	0	4.767432	1.533488	1.060149
52	6	0	7.035022	-0.929521	0.306002
53	1	0	6.513177	-2.998727	-0.077835
54	1	0	7.212517	1.182565	0.742011
55	6	0	8.508454	-1.156398	0.140282
56	1	0	9.064899	-0.212297	0.063577
57	1	0	8.722678	-1.763525	-0.752983
58	1	0	8.908307	-1.711849	1.006280
59	47	0	-0.415646	-0.805485	1.392244
60	5	0	-2.820848	0.285283	2.639102
61	9	0	-3.450065	0.875494	1.534490
62	9	0	-1.500826	0.844710	2.746396
63	9	0	-2.588301	-1.109187	2.318425
64	9	0	-3.520556	0.415357	3.788872

INT4·AgBF$_4$

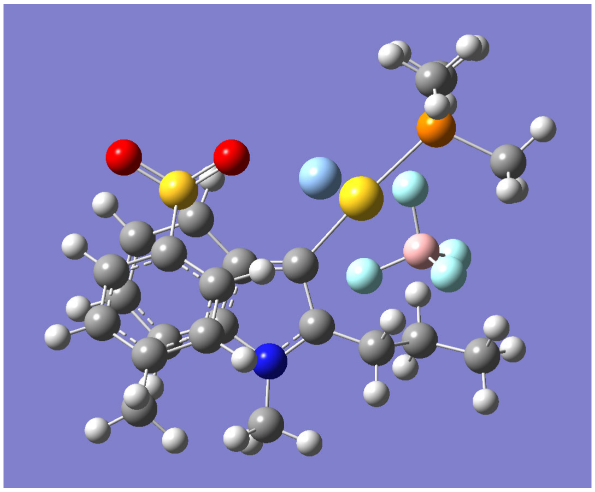

1	6	0	3.986718	-0.851074	-2.827453
2	6	0	3.632981	0.528731	-2.571016
3	6	0	2.408048	0.796494	-2.041801
4	6	0	1.433453	-0.224514	-1.725158
5	6	0	1.880350	-1.636097	-1.762992
6	6	0	3.157696	-1.871413	-2.495806
7	1	0	4.941451	-1.060023	-3.313178
8	1	0	4.336348	1.321225	-2.833328
9	1	0	1.083282	-2.354036	-2.008954
10	1	0	3.447488	-2.906705	-2.677194
11	6	0	0.249379	0.380121	-1.348020
12	6	0	0.531144	1.811621	-1.378929
13	6	0	-0.429784	2.874959	-1.006577
14	1	0	-0.847678	2.581649	-0.032044
15	1	0	0.071598	3.842128	-0.865401
16	7	0	1.798260	2.032580	-1.790849
17	6	0	2.477265	3.302800	-1.996956
18	1	0	3.364160	3.365297	-1.349538
19	1	0	1.804759	4.130529	-1.753344
20	1	0	2.784113	3.401937	-3.048344
21	16	0	2.287406	-2.230084	0.032381
22	8	0	0.990616	-2.322704	0.764505
23	8	0	3.069651	-3.457279	-0.105567
24	79	0	-1.618186	-0.373892	-0.890787
25	15	0	-3.796871	-1.060328	-0.304895
26	6	0	-3.911699	-1.929803	1.299035
27	6	0	-4.911973	0.374138	-0.137594
28	6	0	-4.594591	-2.176219	-1.514122
29	1	0	-4.951142	-2.232851	1.500407

30	1	0	-3.572146	-1.251331	2.095261
31	1	0	-3.275484	-2.827951	1.288543
32	1	0	-5.923650	0.053981	0.156301
33	1	0	-4.965416	0.914167	-1.094832
34	1	0	-4.493389	1.051702	0.620581
35	1	0	-4.622566	-1.692021	-2.501842
36	1	0	-5.622222	-2.421826	-1.203802
37	1	0	-4.012539	-3.105976	-1.601382
38	6	0	-1.601235	3.035108	-2.002382
39	1	0	-1.212827	3.361588	-2.981831
40	1	0	-2.064528	2.047079	-2.164755
41	6	0	-2.644105	4.014200	-1.474400
42	1	0	-2.217493	5.019993	-1.329452
43	1	0	-3.037773	3.674226	-0.504564
44	1	0	-3.484757	4.109923	-2.178821
45	6	0	3.279447	-0.932998	0.731132
46	6	0	4.666489	-1.109207	0.726442
47	6	0	2.694202	0.236733	1.233848
48	6	0	5.473789	-0.090660	1.229742
49	1	0	5.097762	-2.034408	0.342869
50	6	0	3.520492	1.238118	1.733890
51	1	0	1.616256	0.394005	1.237800
52	6	0	4.920199	1.094898	1.741878
53	1	0	6.557930	-0.224402	1.233852
54	1	0	3.062248	2.143947	2.137767
55	6	0	5.797578	2.165939	2.325690
56	1	0	6.796962	2.171196	1.866915
57	1	0	5.936347	1.995865	3.407510
58	1	0	5.353276	3.165537	2.211214
59	47	0	-0.470711	-0.940213	1.680437
60	5	0	-1.697312	1.636330	2.404089
61	9	0	-0.339839	1.549738	1.946507
62	9	0	-2.025803	0.268458	2.801655
63	9	0	-2.511491	1.956783	1.314989
64	9	0	-1.824443	2.482599	3.448384

TS2

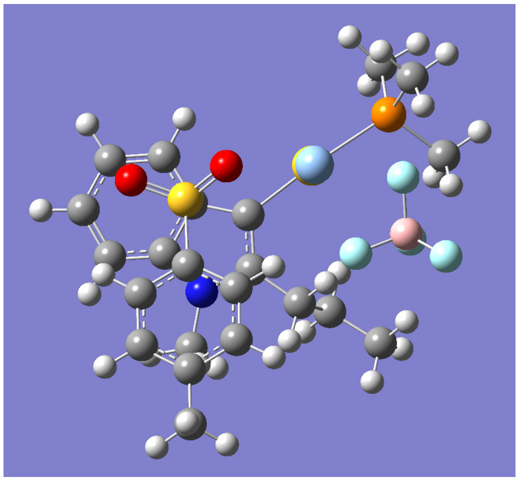

1	6	0	-3.549577	-3.989757	0.350151
2	6	0	-3.709165	-2.818165	1.062950
3	6	0	-2.610029	-1.935342	1.100776
4	6	0	-1.385598	-2.211130	0.389066
5	6	0	-1.226407	-3.457115	-0.245469
6	6	0	-2.315059	-4.310087	-0.288383
7	1	0	-4.377341	-4.697856	0.284881
8	1	0	-4.644616	-2.597618	1.577991
9	1	0	-0.284953	-3.708868	-0.736257
10	1	0	-2.233427	-5.260376	-0.819695
11	6	0	-0.466654	-1.071892	0.639584
12	6	0	-1.176981	-0.207088	1.468698
13	6	0	-0.704478	1.062725	2.096197
14	1	0	-0.065650	1.583425	1.372534
15	1	0	-1.551689	1.735260	2.300669
16	7	0	-2.440335	-0.740416	1.736775
17	6	0	-3.441611	-0.080478	2.556679
18	1	0	-3.761452	0.859517	2.084057
19	1	0	-3.026235	0.141288	3.549233
20	1	0	-4.312382	-0.733219	2.676353
21	16	0	-2.078367	-0.865863	-1.605273
22	8	0	-0.936974	-0.366258	-2.423386
23	8	0	-2.907801	-1.926971	-2.178221
24	79	0	1.569262	-0.982810	0.348267
25	15	0	3.922210	-0.814780	0.194929
26	6	0	4.557435	-0.131798	-1.375366
27	6	0	4.595178	0.271144	1.496320
28	6	0	4.772347	-2.421655	0.397268
29	1	0	5.658669	-0.130326	-1.374731

30	1	0	4.191156	0.898349	-1.489505
31	1	0	4.201791	-0.739802	-2.220922
32	1	0	5.690266	0.349741	1.412225
33	1	0	4.335049	-0.134780	2.485458
34	1	0	4.133821	1.263956	1.394965
35	1	0	4.488378	-2.873460	1.359451
36	1	0	5.865615	-2.292098	0.369574
37	1	0	4.470585	-3.106627	-0.409332
38	6	0	0.107388	0.840284	3.387486
39	1	0	-0.510980	0.315372	4.137185
40	1	0	0.943910	0.159941	3.152064
41	6	0	0.645518	2.149438	3.954041
42	1	0	-0.171838	2.846939	4.201396
43	1	0	1.303560	2.643608	3.223602
44	1	0	1.222567	1.976264	4.875469
45	6	0	-3.090055	0.467230	-1.017399
46	6	0	-4.460674	0.222765	-0.864189
47	6	0	-2.508126	1.698112	-0.695117
48	6	0	-5.267731	1.262398	-0.415555
49	1	0	-4.877907	-0.753867	-1.111840
50	6	0	-3.339769	2.719364	-0.246881
51	1	0	-1.437548	1.880602	-0.776367
52	6	0	-4.727143	2.525748	-0.101188
53	1	0	-6.342667	1.096354	-0.314518
54	1	0	-2.895101	3.687275	-0.005379
55	6	0	-5.612327	3.649265	0.351449
56	1	0	-5.091646	4.324372	1.046207
57	1	0	-6.528582	3.281277	0.834661
58	1	0	-5.922340	4.256420	-0.517298
59	47	0	0.935465	0.741054	-1.822248
60	5	0	1.853968	3.004171	-0.228471
61	9	0	2.197770	2.311488	0.932294
62	9	0	2.179513	4.312822	-0.206646
63	9	0	0.453831	2.800945	-0.478448
64	9	0	2.509440	2.321521	-1.344756

INT 3a·$AgBF_4$

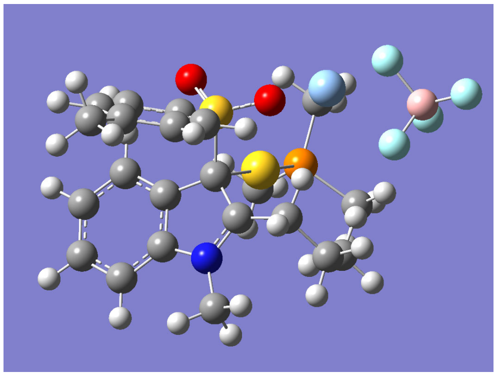

1	6	0	-3.391327	-4.105193	-0.439439
2	6	0	-3.148862	-3.542069	0.813860
3	6	0	-2.478132	-2.318667	0.837297
4	6	0	-2.023789	-1.680317	-0.331867
5	6	0	-2.275449	-2.257118	-1.582271
6	6	0	-2.968341	-3.467547	-1.618876
7	1	0	-3.918552	-5.058963	-0.502581
8	1	0	-3.474004	-4.044466	1.725165
9	1	0	-1.931761	-1.768492	-2.494090
10	1	0	-3.178927	-3.934503	-2.582877
11	6	0	-1.291243	-0.469474	0.112271
12	6	0	-1.468094	-0.414243	1.559098
13	6	0	-1.008143	0.652352	2.500677
14	1	0	-0.565409	1.449283	1.892916
15	1	0	-1.902927	1.088007	2.980603
16	7	0	-2.133488	-1.512061	1.943595
17	6	0	-2.494383	-1.878849	3.306831
18	1	0	-3.487745	-2.345074	3.294750
19	1	0	-2.540497	-0.986972	3.939043
20	1	0	-1.767983	-2.591402	3.725167
21	16	0	-1.546254	1.006978	-0.881724
22	8	0	-0.719898	2.111122	-0.277743
23	8	0	-1.294125	0.630468	-2.275405
24	79	0	0.843512	-1.006202	-0.241506
25	15	0	3.003945	-1.732469	-0.706643
26	6	0	4.059715	-1.774306	0.773287
27	6	0	2.956194	-3.449003	-1.332270
28	6	0	3.848981	-0.775252	-2.002075
29	1	0	5.066750	-2.128141	0.503582
30	1	0	3.615594	-2.457572	1.511950
31	1	0	4.138534	-0.764677	1.192680
32	1	0	3.977548	-3.805303	-1.540277

33	1	0	2.363628	-3.493434	-2.257908
34	1	0	2.490232	-4.109147	-0.586110
35	1	0	3.256462	-0.828486	-2.927572
36	1	0	4.848945	-1.199635	-2.181828
37	1	0	3.963626	0.272373	-1.698497
38	6	0	-0.007679	0.231236	3.606164
39	1	0	0.289013	1.160902	4.116634
40	1	0	-0.519584	-0.372303	4.370338
41	6	0	1.237280	-0.502522	3.121884
42	1	0	1.817494	0.097007	2.405971
43	1	0	0.988055	-1.468451	2.650138
44	1	0	1.900712	-0.721498	3.972378
45	6	0	-3.235989	1.484896	-0.664334
46	6	0	-4.194933	0.938157	-1.524580
47	6	0	-3.585068	2.374425	0.356157
48	6	0	-5.529985	1.281359	-1.336337
49	1	0	-3.896104	0.269674	-2.332049
50	6	0	-4.928889	2.703324	0.522579
51	1	0	-2.817748	2.819580	0.989987
52	6	0	-5.921268	2.163913	-0.313235
53	1	0	-6.286658	0.863795	-2.004646
54	1	0	-5.212520	3.405520	1.309531
55	6	0	-7.367474	2.536689	-0.145915
56	1	0	-7.541350	3.101291	0.780518
57	1	0	-8.010677	1.642704	-0.132117
58	1	0	-7.705940	3.162097	-0.989114
59	47	0	1.469886	2.010700	-0.467651
60	5	0	4.276785	1.966171	0.432133
61	9	0	4.738703	3.076382	1.048414
62	9	0	3.173535	1.385310	1.168583
63	9	0	5.215297	0.994030	0.186997
64	9	0	3.646306	2.326372	-0.850656

8. Crystallographic data of 4a

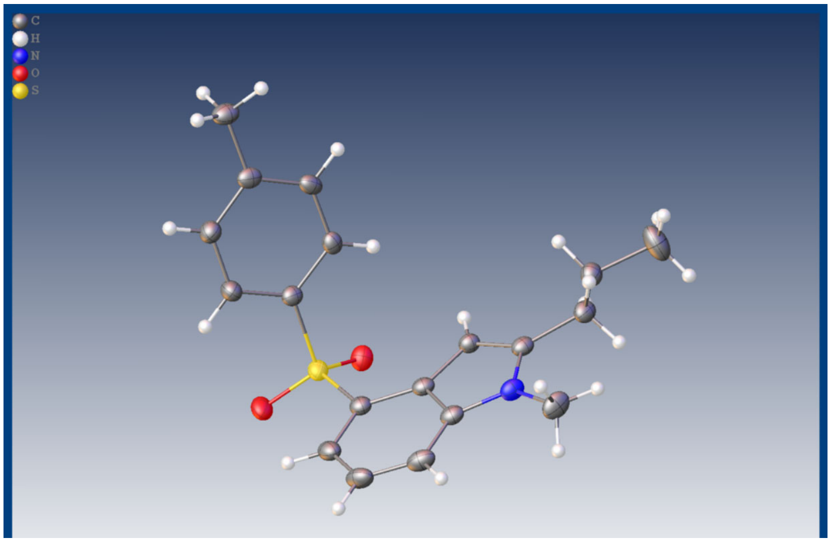

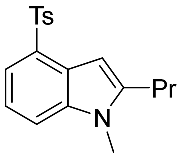

1-methyl-2-propyl-4-tosyl-1H-indole (**4a**) **CCDC 2402**

Single-crystal X-ray diffraction data of **4a** isomer were collected on Bruker D8 Venture iμs diamond duo diffractometer (graphite-monochromated Mo Kα_1 radiation, λ = 0.71073 Å). The crystallographic data and structure refinement of **4a** is summarized in **Table S7**.

Table S7. Crystallographic data and structure refinement for 4a.

Compound	**4a**
Formula	$C_{19}H_{21}NO_2S$
Identification code	230602_a
Formula weight	327.43
Temperature (K)	100.82
Wavelength (Å)	0.71073
Crystal System	Monoclinic
Space group	*P*2$_1$/*c*
a (Å)	11.7575(9)
b (Å)	14.3844(9)
c (Å)	10.1883(8)
α (°)	90
β (°)	107.058(3)
γ (°)	90

V (Å^3)	1647.3(2)
Z	4
Reflns collected	41641
GOF on F^2	1.073
R_1 (I>2σ (I))[a]	0.0428
wR_2 (all data)[b]	0.1097

[a] $R_1 = \Sigma||F_o| - |F_c||/\Sigma|F_o|$

[b] $wR_2 = [\Sigma[w(F_o^2 - F_c^2)^2]/\Sigma w(F_o^2)^2]^{1/2}$

9. Analytical data of substrates and products

1a

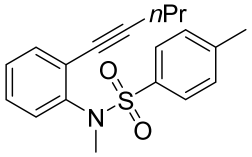

Colorless crystal. Yield: 78 % (3 steps). R_f = 0.73 (Hexane : EtOAc = 4:1).

^{1}H-NMR (400 MHz, $CDCl_3$) δ 7.64 (d, J = 8.2 Hz, 2H), 7.36 (dd, J = 6.2, 2.1 Hz, 1H), 7.28-7.20 (m, 5H), 3.27 (s, 3H), 2.42 (s, 3H), 2.11 (t, J = 7.3 Hz, 2H), 1.45 (td, J = 14.4, 7.3 Hz, 2H), 0.95 (t, J = 7.3 Hz, 3H).

^{13}C-NMR (101 MHz, $CDCl_3$) δ 143.0, 142.1, 136.5, 133.6, 130.3, 129.3, 128.1, 127.8, 127.8, 123.7, 95.4, 37.9, 21.9, 21.5, 21.4, 13.6.

IR(neat): 3065, 3029, 2963, 2933, 2872, 2834, 2584, 2233, 1921, 1813, 1727, 1598, 1486, 1446, 1401, 1380, 1346, 1305, 1288, 1233, 1153, 1107, 1090, 1068, 1036, 1019, 956, 892, 866, 814, 765, 747, 721, 672, 650.

HRMS (APCI+) calcd. for $C_{19}H_{21}NO_2S$ ($[M+H]^+$): 328.1366, found: 328.1366.

1b

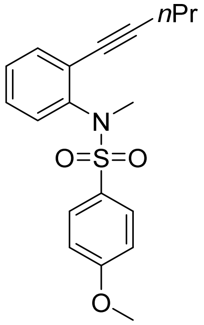

White solid. Yield: 74 % (3 steps). R_f = 0.61 (Hexane : EtOAc = 4:1).

^{1}H-NMR (400 MHz, $CDCl_3$) δ 7.69 (dt, J = 9.5, 2.4 Hz, 2H), 7.37 (dd, J = 6.6, 2.1 Hz, 1H), 7.29-7.20 (m, 3H), 6.93 (dt, J = 9.5, 2.4 Hz, 2H), 3.86 (s, 3H), 3.26 (s, 3H), 2.15 (t, J = 7.1 Hz, 2H), 1.47 (td, J = 14.4, 7.3 Hz, 2H), 0.96 (t, J = 7.3 Hz, 3H). ^{13}C-NMR (101 MHz, $CDCl_3$) δ 162.7, 142.2, 133.7, 131.1, 130.3, 129.9, 128.1, 127.7, 123.7, 113.8, 95.4, 55.5, 37.9, 21.9, 21.5, 13.6.

IR(neat): 3098, 3066, 2963, 2934, 2904, 2872, 2840, 2564, 2232, 2056, 1908, 1595, 1578, 1542, 1496, 1487, 1458, 1444, 1416, 1345, 1303, 1256, 1180, 1149, 1109, 1092, 1068, 1025, 891, 866, 833, 803, 766, 747, 726, 711, 675, 654, 629.

HRMS (APCI+) calcd. for $C_{19}H_{21}NO_3S$ ($[M+H]^+$): 344.1315, found: 344.1315.

1c

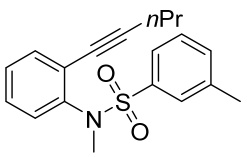

Colorless oil. Yield: 80 % (3 steps). R_f = 0.73 (Hexane : EtOAc = 4:1).
^{1}H-NMR (400 MHz, $CDCl_3$) δ 7.58-7.55 (m, 2H), 7.38-7.32 (m, 3H), 7.28-7.21 (m, 3H), 3.28 (s, 3H), 2.39 (s, 3H), 2.10 (t, J = 7.1 Hz, 2H), 1.45 (td, J = 14.4, 7.3 Hz, 2H), 0.95 (t, J = 7.3 Hz, 3H).
^{13}C-NMR (101 MHz, $CDCl_3$) δ 142.1, 139.3, 138.7, 133.6, 133.1, 130.3, 128.5, 128.1, 127.8, 125.0, 123.7, 95.5, 77.2, 38.0, 21.9, 21.4, 21.3, 13.6.
IR(neat): 3064, 3028, 2962, 2933, 2872, 2835, 2233, 1959, 1810, 1598, 1485, 1446, 1420, 1379, 1347, 1305, 1285, 1224, 1171, 1149, 1095, 1085, 1068, 1036, 998, 957, 894, 870, 786, 765, 747, 722, 687, 663.
HRMS (APCI+) calcd. for $C_{19}H_{21}NO_2S$ ($[M+H]^+$): 328.1366, found: 328.1366.

1d

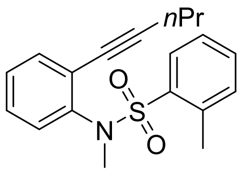

Light yellow oil. Yield: 67% (3 steps). R_f = 0.72 (Hexane : EtOAc = 4:1).
^{1}H-NMR (400 MHz, $CDCl_3$) δ 7.90 (d, J = 7.8 Hz, 1H), 7.43-7.34 (m, 3H), 7.27-7.19 (m, 4H), 3.32 (s, 3H), 2.45 (s, 3H), 2.14 (t, J = 7.1 Hz, 2H), 1.45 (td, J = 14.5, 7.2 Hz, 2H), 0.93 (t, J = 7.3 Hz, 3H).
^{13}C-NMR (101 MHz, $CDCl_3$) δ 142.1, 138.3, 137.9, 133.6, 132.6, 132.5, 130.4, 130.3, 128.2, 127.7, 125.8, 123.6, 95.8, 38.3, 21.9, 21.4, 21.1, 13.6.
IR(neat): 3063, 2963, 2934, 2903, 2872, 2833, 2232, 1942, 1822, 1715, 1595, 1569, 1486, 1446, 1380, 1340, 1280, 1236, 1155, 1131, 1106, 1065, 1035, 956, 890, 865, 806, 761, 748, 724, 709, 689, 662.
HRMS (APCI+) calcd. for $C_{19}H_{21}NO_2S$ ($[M+H]^+$): 328.1366, found: 328.1366.

1e

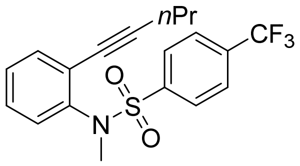

Colorless oil. Yield: 58 % (3 steps). R_f = 0.78 (Hexane : EtOAc = 4:1).
^{1}H-NMR (400 MHz, $CDCl_3$) δ 7.90 (d, J = 7.8 Hz, 1H), 7.43-7.34 (m, 3H), 7.27-7.19 (m, 4H), 3.32 (s, 3H), 2.45 (s, 3H), 2.14 (t, J = 7.1 Hz, 2H), 1.45 (td, J = 14.5, 7.2 Hz, 2H), 0.93 (t, J = 7.3 Hz, 3H)
^{13}C-NMR (101 MHz, $CDCl_3$) δ 142.1, 138.3, 137.9, 133.6, 132.6, 132.5, 130.4, 130.3, 128.2, 127.7, 125.8, 123.6, 95.8, 38.3, 21.9, 21.4, 21.1, 13.6.
^{19}F-NMR (376 MHz, $CDCl_3$) δ -64.0 (s).
IR(neat): 3105, 3072, 2967, 2936, 2875, 2834, 2235, 1938, 1608, 1486, 1447, 1405, 1356, 1321, 1296, 1287, 1236, 1162, 1129, 1106, 1091, 1061, 1037, 1016, 960, 893, 869, 843, 786, 766, 739, 722, 710, 673, 607.
HRMS (APCI+) calcd. for $C_{19}H_{18}F_3NO_2S$ ($[M+H]^+$): 382.1083, found: 328.1084.

1f

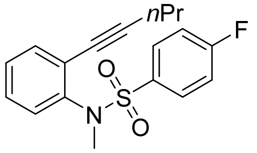

Yellow oil. Yield: 70 % (3 steps). R_f = 0.75 (Hexane : EtOAc = 4:1).

^{1}H-NMR (400 MHz, $CDCl_3$) δ 7.75 (tt, J = 7.3, 2.2 Hz, 2H), 7.36 (dd, J = 7.1, 2.1 Hz, 1H), 7.32-7.22 (m, 3H), 7.16-7.11 (m, 2H), 3.30 (s, 3H), 2.12 (t, J = 7.1 Hz, 2H), 1.46 (td, J = 14.5, 7.2 Hz, 2H), 0.96 (t, J = 7.3 Hz, 3H).

^{13}C-NMR (101 MHz, $CDCl_3$) δ 163.8, 141.6, 135.5, 133.8, 130.6, 130.4, 130.3, 128.3, 128.0, 123.5, 115.9, 115.7, 95.6, 77.2, 38.0, 21.9, 21.4, 13.5.

^{19}F-NMR (376 MHz, $CDCl_3$) δ -107.1 - -107.2 (m).

IR(neat): 3104, 3071, 2964, 2935, 2873, 2835, 2232, 1908, 1591, 1492, 1446, 1404, 1350, 1291, 1234, 1175, 1151, 1089, 1068, 1035, 1013, 956, 893, 868, 837, 818, 765, 747, 725, 674, 652.

HRMS (APCI+) calcd. for $C_{18}H_{18}FNO_2S$ ($[M+H]^+$): 332.1115, found: 332.1115.

1g

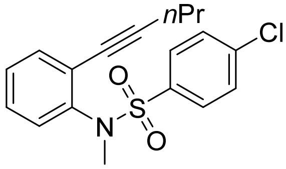

Colorless oil. Yield: 72 % (3 steps). R_f = 0.79 (Hexane : EtOAc = 4:1).

^{1}H-NMR (400 MHz, $CDCl_3$) δ 7.69-7.65 (m, 2H), 7.44 (dd, J = 11.0, 2.3 Hz, 2H), 7.36 (dd, J = 7.3, 1.8 Hz, 1H), 7.33-7.22 (m, 3H), 3.30 (s, 3H), 2.09 (t, J = 7.1 Hz, 2H), 1.45 (td, J = 14.4, 7.3 Hz, 2H), 0.95 (t, J = 7.3 Hz, 3H)

^{13}C-NMR (101 MHz, $CDCl_3$) δ 141.5, 138.9, 138.0, 133.9, 130.7, 129.2, 129.0, 128.3, 128.1, 123.4, 95.7, 77.1, 38.0, 21.9, 21.4, 13.6.

IR(neat): 3091, 3069, 2963, 2934, 2872, 2834, 2232, 1917, 1585, 1485, 1446, 1394, 1352, 1279, 1234, 1177, 1157, 1090, 1069, 1036, 1013, 954, 894, 868, 828, 758, 718, 667, 617.

HRMS (APCI+) calcd. for $C_{18}H_{18}ClNO_2S$ ($[M+H]^+$): 348.0820, found: 348.0820.

1h

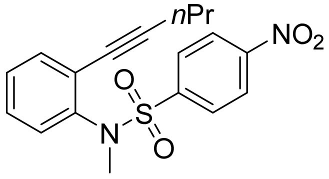

White flake crystal. Yield: 57 % (3 steps). R_f = 0.62 (Hexane : EtOAc = 4:1).

^{1}H-NMR (400 MHz, $CDCl_3$) δ 8.30 (dt, J = 9.0, 2.1 Hz, 2H), 7.90 (dt, J = 9.2, 2.3 Hz, 2H), 7.37-7.26 (m, 4H), 3.36 (s, 3H), 2.01 (t, J = 7.1 Hz, 2H), 1.40 (td, J = 14.4, 7.3 Hz, 2H), 0.91 (t, J = 7.3 Hz, 3H)^{13}C-NMR (101 MHz, $CDCl_3$) δ 150.0, 145.3, 140.8, 134.0, 130.8, 128.9, 128.6, 128.5, 123.9,

123.3, 95.8, 38.2, 21.9, 21.4, 13.5.

IR(neat): 3108, 3068, 3034, 2961, 2932, 2901, 2868, 2833, 2232, 1945, 1606, 1524, 1486, 1447, 1400, 1352, 1306, 1283, 1225, 1189, 1157, 1111, 1088, 1067, 1036, 1011, 979, 958, 893, 874, 856, 774, 741, 724, 712, 684, 665, 608.

HRMS (APCI+) calcd. for $C_{18}H_{18}N_2O_4S$ ($[M+H]^+$): 359.1060, found: 359.1060.

1i

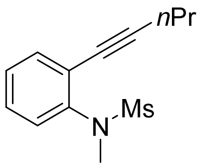

Colorless oil, yield: 72% (3 steps). R_f = 0.54 (Hexane : EtOAc = 4:1).

^{1}H-NMR (400 MHz, $CDCl_3$) δ 7.43 (ddd, *J* = 16.9, 7.6, 1.6 Hz, 2H), 7.34-7.25 (m, 2H), 3.38 (s, 3H), 3.00 (s, 3H), 2.43 (t, *J* = 7.1 Hz, 2H), 1.65 (td, *J* = 14.7, 7.3 Hz, 2H), 1.06 (t, *J* = 7.3 Hz, 3H).

^{13}C-NMR (101 MHz, $CDCl_3$) δ 141.6, 133.8, 131.3, 128.8, 128.2, 123.1, 96.0, 77.9, 39.0, 37.9, 22.1, 21.5, 13.7.

IR(neat): 3067, 3021, 2963, 2934, 2873, 2231, 1595, 1486, 1448, 1380, 1335, 1287, 1235, 1177, 1145, 1109, 1069, 1037, 961, 894, 869, 766, 747, 704, 660.

HRMS (APCI+) calcd. for $C_{13}H_{17}NO_2S$ ($[M+H]^+$): 252.1053, found: 252.1053.

1j

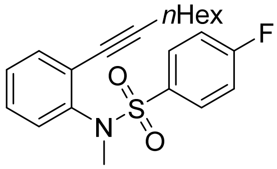

Yellow oil. Yield: 67 % (3 steps). R_f = 0.76 (Hexane : EtOAc = 4:1).

^{1}H-NMR (400 MHz, $CDCl_3$) δ 7.75 (tt, *J* = 7.3, 2.3 Hz, 2H), 7.36-7.22 (m, 4H), 7.16-7.11 (m, 2H), 3.29 (s, 3H), 2.12 (t, *J* = 7.1 Hz, 2H), 1.45-1.25 (m, 8H), 0.90 (t, *J* = 6.9 Hz, 3H).

^{13}C-NMR (101 MHz, $CDCl_3$) δ 166.3, 163.8, 141.6, 135.5, 133.8, 130.7, 130.4, 130.3, 128.3, 128.0, 123.5, 115.9, 115.7, 95.8, 37.9, 31.3, 28.6, 28.4, 22.5, 19.4, 14.0.

^{19}F-NMR (376 MHz, $CDCl_3$) δ -107.0 - -107.1 (m).

IR(neat): 3104, 3070, 2954, 2930, 2858, 2231, 1904, 1592, 1492, 1446, 1404, 1352, 1291, 1234, 1175, 1151, 1089, 1069, 1036, 1013, 949, 890, 868, 837, 818, 765, 747, 724, 675, 653.

HRMS (APCI+) calcd. for $C_{21}H_{24}FNO_2S$ ($[M+H]^+$): 374.1584, found: 374.1585.

1k

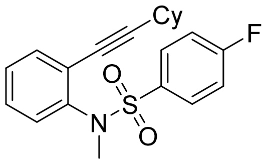

Colorless oil. Yield: 65 % (3 steps). R_f = 0.74 (Hexane : EtOAc = 4:1).

^{1}H-NMR (400 MHz, CDCl$_3$) δ 7.75 (dq, J = 9.4, 2.5 Hz, 2H), 7.38-7.21 (m, 4H), 7.17-7.11 (m, 2H), 3.30 (s, 3H), 2.30 (s, 1H), 1.70 (d, J = 6.4 Hz, 4H), 1.53 (s, 1H), 1.35-1.26 (m, 5H)

^{13}C-NMR (101 MHz, CDCl$_3$) δ 166.3, 163.8, 141.6, 135.6, 133.8, 130.4, 130.3, 128.2, 127.9, 123.5, 116.0, 115.8, 99.7, 37.9, 32.3, 29.7, 25.7, 24.8.

^{19}F-NMR (376 MHz, CDCl$_3$) δ -107.1 - -107.2 (m).

IR(neat): 3104, 3070, 2929, 2853, 2662, 2592, 2226, 1905, 1591, 1492, 1447, 1404, 1351, 1291, 1231, 1175, 1151, 1089, 1069, 1035, 1013, 955, 891, 868, 837, 818, 790, 764, 742, 711, 688, 654.

HRMS (APCI+) calcd. for $C_{21}H_{22}FNO_2S$ ([M+H]$^+$): 3372.1428, found: 372.1428.

1l

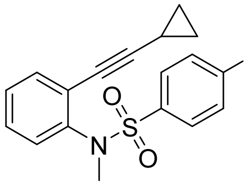

Colorless oil. Yield: 61 % (3 steps). R_f = 0.66 (Hexane : EtOAc = 4:1).

^{1}H-NMR (400 MHz, CDCl$_3$) δ 7.75 (tt, J = 7.3, 2.2 Hz, 2H), 7.34-7.20 (m, 4H), 7.18-7.12 (m, 2H), 3.27 (s, 3H), 1.20-1.13 (m, 1H), 0.81-0.77 (m, 2H), 0.60-0.56 (m, 2H)

^{13}C-NMR (101 MHz, CDCl$_3$) δ 166.4, 163.9, 141.8, 135.5, 133.7, 130.7, 130.4, 130.3, 128.2, 128.0, 123.4, 116.0, 115.8, 98.7, 72.3, 37.9, 8.6, 0.2

^{19}F-NMR (376 MHz, CDCl$_3$) δ -107.0 - -107.1 (m).

IR(neat): 3103, 3072, 3012, 2940, 2816, 2319, 2229, 1905, 1736, 1591, 1492, 1446, 1421, 1404, 1349, 1291, 1234, 1175, 1150, 1117, 1089, 1065, 1033, 1013, 956, 894, 870, 833, 817, 765, 747, 721, 704, 671, 651.

HRMS (APCI+) calcd. for $C_{18}H_{16}FNO_2S$ ([M+H]$^+$): 330.0958, found: 330.0959.

1m

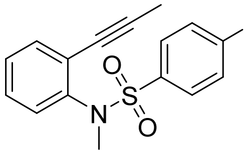

Colorless oil. Yield: 49% (3 steps). R_f = 0.68 (Hexane : EtOAc = 4:1).

^{1}H-NMR (400 MHz, CDCl$_3$) δ 7.76 (dq, J = 11.9, 2.7 Hz, 2H), 7.35-7.22 (m, 4H), 7.18-7.12 (m, 2H), 3.29 (s, 3H), 1.77 (s, 3H).

^{13}C-NMR (101 MHz, CDCl$_3$) δ 166.3, 163.8, 141.7, 135.5, 135.4, 133.7, 130.8, 130.4, 130.3, 128.4, 128.0, 123.3, 115.9, 115.7, 91.0, 76.3, 37.9, 4.3.

^{19}F-NMR (376 MHz, CDCl$_3$) δ -107.1- -107.2 (m).

IR(neat): 3104, 3071, 2917, 2853, 2818, 2239, 1908, 1591, 1492, 1445, 1405, 1348, 1291, 1232, 1150, 1088, 1068, 1036, 1013, 950, 889, 868, 837, 818, 766, 747, 730, 676, 652.

HRMS (APCI+) calcd. for $C_{16}H_{14}FNO_2S$ ([M+H]$^+$): 304.0802, found: 304.0802.

1n

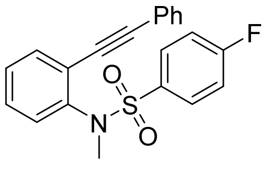

White solid. Yield: 80 % (3 steps). R_f = 0.56 (Hexane : EtOAc = 4:1).

^{1}H-NMR (400 MHz, $CDCl_3$) δ 7.77-7.73 (m, 2H), 7.51-7.49 (m, 1H), 7.41-7.25 (m, 8H), 6.99-6.94 (m, 2H), 3.39 (s, 3H).

^{13}C-NMR (101 MHz, $CDCl_3$) δ 166.3, 163.7, 141.9, 135.2, 133.5, 131.3, 130.8, 130.3, 130.3, 129.1, 128.7, 128.3, 128.2, 122.8, 122.5, 116.1, 115.9, 94.2, 85.9, 38.1.

^{19}F-NMR (376 MHz, $CDCl_3$) δ -106.7 (m).

IR(neat): 3103, 3065, 3032, 2979, 2939, 2884, 2817, 2324, 2221, 1907, 1748, 1591, 1542, 1493, 1479, 1444, 1419, 1404, 1350, 1291, 1235, 1150, 1088, 1065, 1034, 1013, 951, 899, 835, 818, 755, 723, 689, 680, 653.

HRMS (APCI+) calcd. for $C_{21}H_{16}FNO_2S$ ($[M+H]^+$): 366.0958, found: 366.0959.

1o

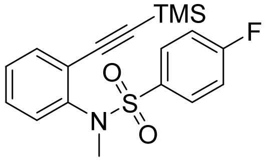

Colorless crystal. Yield: 84% (3 steps). R_f = 0.69 (Hexane : EtOAc = 4:1).

^{1}H-NMR (400 MHz, $CDCl_3$) δ 7.75-7.71 (m, 2H), 7.44 (dd, J = 7.6, 1.6 Hz, 1H), 7.40-7.31 (m, 2H), 7.26 (td, J = 7.6, 1.4 Hz, 1H), 7.15-7.11 (m, 2H), 3.31 (s, 3H), 0.13 (s, 9H)

^{13}C-NMR (101 MHz, $CDCl_3$) δ 166.4, 163.9, 142.1, 135.3, 134.3, 130.7, 130.4, 130.3, 129.3, 127.9, 122.4, 116.1, 115.9, 101.3, 99.9, 37.9, -0.3.

^{19}F-NMR (376 MHz, $CDCl_3$) δ -106.6 - -106.7 (m).

IR(neat): 3105, 3072, 2959, 2899, 2818, 2159, 1903, 1592, 1493, 1484, 1445, 1406, 1353, 1292, 1250, 1234, 1175, 1152, 1089, 1067, 1036, 1014, 899, 858, 836, 760, 714, 697, 658, 642.

HRMS (APCI+) calcd. for $C_{18}H_{20}FNO_2SSi$ ($[M+H]^+$): 362.1041, found: 362.1041.

1p

1p was synthesized using following procedure:

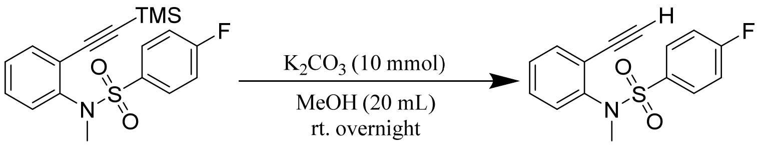

1o **1p**

1o (1.50 g, 4.2 mmol) and anhydrous K_2CO_3 (1.38 g, 10 mmol) were charged into a 50 mL round bottle flask with a magnetic stirrer. 20 mL of MeOH was added to the mixture. The mixture was stirred at room temperature overnight, then 10 mL of water was added to the mixture. The mixture was extracted with EtOAc, washed with brine, dried over Na_2SO_4 and evaporated under reduced

pressure. **1p** was obtained as white needle crystal (1.08 g, 90 %). R_f = 0.53 (Hexane : EtOAc = 4:1).
^{1}H-NMR (400 MHz, $CDCl_3$) δ 7.74 (tt, J = 7.3, 2.3 Hz, 2H), 7.48-7.46 (m, 1H), 7.38-7.34 (m, 1H), 7.31-7.26 (m, 2H), 7.18-7.12 (m, 2H), 3.30 (s, 3H), 2.93 (s, 1H).
^{13}C-NMR (101 MHz, $CDCl_3$) δ 166.5, 164.0, 142.6, 134.9, 134.4, 130.5, 130.4, 129.7, 128.2, 122.0, 116.1, 115.9, 81.9, 79.9, 38.2.
^{19}F-NMR (376 MHz, $CDCl_3$) δ -106.6 - -106.7 (m).
IR(neat): 3273, 3104, 3071, 2980, 2943, 2885, 1592, 1493, 1446, 1405, 1349, 1292, 1234, 1176, 1151, 1088, 1065, 1035, 1013, 895, 872, 838, 819, 772, 749, 713, 689, 654.
HRMS (APCI+) calcd. for $C_{15}H_{12}FNO_2S$ ($[M+H]^+$): 290.0646, found: 290.0646.

1q

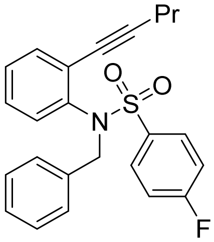

Colorless oil. yield: 81% (3 steps). R_f = 0.75 (Hexane : EtOAc = 4:1).
^{1}H-NMR (400 MHz, $CDCl_3$) δ 7.76-7.72 (m, 2H), 7.30 (dd, J = 7.3, 1.8 Hz, 1H), 7.26-7.05 (m, 10H), 4.92 (s, 2H), 2.13 (t, J = 7.1 Hz, 2H), 1.50 (td, J = 14.5, 7.2 Hz, 2H), 0.99 (t, J = 7.3 Hz, 3H).
^{13}C-NMR (101 MHz, $CDCl_3$) δ 166.3, 163.7, 138.8, 136.6, 136.6, 136.2, 133.7, 132.8, 130.4, 130.3, 128.8, 128.2, 128.1, 127.6, 123.9, 115.9, 115.7, 95.8, 77.4, 53.7, 21.9, 21.5, 13.6.
^{19}F-NMR (376 MHz, $CDCl_3$) δ -107.1 - -107.2 (m).
IR(neat): 3104, 3066, 3032, 2964, 2934, 2904, 2872, 2834, 2232, 1905, 1591, 1493, 1455, 1446, 1404, 1349, 1291, 1234, 1212, 1166, 1153, 1092, 1064, 1039, 1029, 1013, 911, 860, 837, 818, 773, 741, 726, 698, 657, 608.
HRMS (APCI+) calcd. for $C_{24}H_{22}FNO_2S$ ($[M+H]^+$):408.1428, found:408.1428.

1r

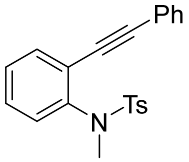

White flake crystal, yield: 81% (3 steps). R_f = 0.69 (Hexane : EtOAc = 4:1).
^{1}H-NMR (400 MHz, $CDCl_3$) δ 7.65 (d, J = 8.2 Hz, 2H), 7.50 (dd, J = 7.3, 1.8 Hz, 1H), 7.40-7.23 (m, 8H), 7.12 (d, J = 7.8 Hz, 2H), 3.36 (s, 3H), 2.22 (s, 3H).
^{13}C-NMR (101 MHz, $CDCl_3$) δ 143.3, 142.3, 136.3, 133.5, 131.4, 130.8, 129.4, 129.0, 128.4, 128.1, 127.9, 127.7, 122.8, 122.8, 94.1, 86.2, 38.0, 21.3.
IR(neat): 3062, 3031, 2974, 2937, 2881, 2815, 2221, 1922, 1748, 1598, 1571, 1495, 1479, 1444, 1419, 1346, 1305, 1289, 1250, 1153, 1090, 1065, 1035, 951, 899, 836, 814, 755, 720, 690, 678, 650.
HRMS (APCI+) calcd. for $C_{22}H_{19}NO_2S$ ($[M+H]^+$): 362.1209, found: 362.1210.

1-Methyl-2-propyl-4-tosyl-1*H*-indole (2a)

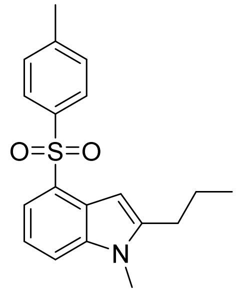

Light yellow solid. 209.0 mg (64% yield, 0.64 mmol).

R_f = 0.37 (Hexane : EtOAc = 4:1).

^{1}H-NMR (400 MHz, $CDCl_3$) δ 7.88 (d, *J* = 8.2 Hz, 2H), 7.81-7.79 (m, 1H), 7.43 (d, *J* = 8.2 Hz, 1H), 7.24-7.20 (m, 3H), 6.75 (s, 1H), 3.66 (s, 3H), 2.72 (t, *J* = 7.6 Hz, 2H), 2.34 (s, 3H), 1.77 (td, *J* = 15.1, 7.3 Hz, 2H), 1.05 (t, *J* = 7.3 Hz, 3H).

^{13}C-NMR (101 MHz, $CDCl_3$) δ 144.5, 143.3, 139.6, 138.3, 130.3, 129.5, 127.3, 124.7, 120.7, 119.7, 113.9, 98.8, 29.7, 29.0, 21.7, 21.5, 14.0.

IR(neat): 3060, 3026, 2960, 2932, 2872, 1915, 1734, 1597, 1562, 1536, 1494, 1454, 1402, 1346, 1313, 1301, 1287, 1183, 1158, 1145, 1122, 1081, 1018, 941, 913, 859, 813, 772, 745, 707, 693, 665.

HRMS (APCI+) calcd. for $C_{19}H_{21}NO_2S$ ($[M+H]^+$): 328.1366, found: 328.1366.

1-Methyl-2-propyl-3-tosyl-1*H*-indole (3a)

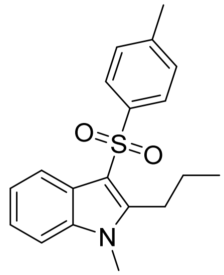

Colorless solid. The pure form of **3a** was obtained more than 3 mg after recrystallization followed by GPC.

R_f = 0.38 (Hexane : EtOAc = 4:1)

^{1}H-NMR (400 MHz, $CDCl_3$) δ 8.13-8.09 (m, 1H), 7.84 (d, *J* = 8.2 Hz, 2H), 7.25-7.18 (m, 5H), 3.64 (d, *J* = 1.4 Hz, 3H), 3.13-3.09 (m, 2H), 2.32 (s, 3H), 1.64-1.58 (m, 2H), 1.05 (t, *J* = 7.3 Hz, 3H).

^{13}C-NMR (101 MHz, $CDCl_3$) δ 146.0, 142.8, 141.7, 136.3, 129.4, 126.0, 124.9, 122.7, 122.1, 119.8, 110.8, 109.5, 77.3, 77.0, 76.7, 29.9, 26.8, 23.0, 21.4, 14.2.

IR(neat): 3053, 3028, 2962, 2931, 2872, 2254, 1916, 1598, 1514, 1494, 1470, 1396, 1340, 1323, 1297, 1249, 1227, 1182, 1139, 1120, 1081, 1038, 1018, 976, 912, 871, 813, 774, 745, 706, 682, 661, 647.

HRMS (APCI+) calcd. for $C_{19}H_{21}NO_2S$ ($[M+H]^+$): 328.1366, found: 328.1366.

1-Methyl-2-propyl-6-tosyl-1*H*-indole (4a)

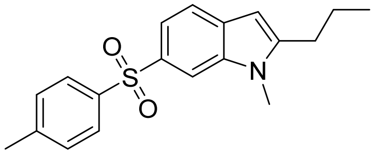

Pale red solid. The pure form of **4a** was obtained more than 3 mg after recrystallization followed by GPC.

R_f = 0.43 (Hexane : EtOAc = 4:1)

^{1}H-NMR (400 MHz, $CDCl_3$) δ 7.95 (s, 1H), 7.84-7.82 (m, 2H), 7.55 (d, *J* = 1.4 Hz, 2H), 7.24 (d, *J* = 8.2 Hz, 2H), 6.30 (s, 1H), 3.73 (s, 3H), 2.73 (t, *J* = 7.6 Hz, 2H), 2.36 (s, 3H), 1.78-1.71 (m, 2H), 1.04 (t, *J* = 7.6 Hz, 3H).

^{13}C-NMR (101 MHz, $CDCl_3$) δ 145.9, 143.2, 140.2, 136.2, 132.8, 131.4, 129.6, 127.3, 120.2, 118.3, 109.2, 99.7, 29.8, 29.0, 21.7, 21.5, 13.9.

IR(neat): 3060, 3028, 2960, 2932, 2872, 1732, 1597, 1532, 1469, 1404, 1334, 1312, 1300, 1289, 1226, 1152, 1116, 1085, 1055, 1017, 912, 869, 815, 773, 738, 710, 688, 664, 622, 608.

HRMS (APCI+) calcd. for $C_{19}H_{21}NO_2S$ ($[M+H]^+$): 328.1366, found: 328.1366.

4-((4-Methoxyphenyl)sulfonyl)-1-methyl-2-propyl-1*H*-indole (2b)

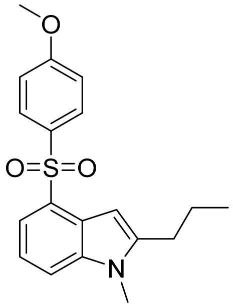

White solid. The pure form of **2b** was obtained more than 5 mg after recrystallization followed by GPC.

R_f = 0.28 (Hexane : EtOAc = 4:1)

^{1}H-NMR (400 MHz, $CDCl_3$) δ 7.93 (d, *J* = 9.2 Hz, 2H), 7.78 (d, *J* = 7.3 Hz, 1H), 7.42 (d, *J* = 8.2 Hz, 1H), 7.20 (t, *J* = 7.8 Hz, 1H), 6.89 (d, *J* = 8.7 Hz, 2H), 6.74 (s, 1H), 3.78 (s, 3H), 3.65 (s, 3H), 2.71 (t, *J* = 7.8 Hz, 2H), 1.77 (td, *J* = 14.9, 7.5 Hz, 2H), 1.05 (t, *J* = 7.3 Hz, 3H).

^{13}C-NMR (101 MHz, $CDCl_3$) δ 162.8, 144.5, 138.3, 134.2, 130.7, 129.3, 124.5, 120.4, 119.7, 114.0, 113.8, 98.7, 55.5, 29.7, 28.9, 21.7, 14.0.

IR(neat): 3095, 3071, 2961, 2932, 2872, 2841, 2576, 2254, 2056, 1906, 1594, 1577, 1537, 1496, 1454, 1427, 1403, 1363, 1348, 1314, 1295, 1277, 1256, 1202, 1176, 1156, 1142, 1121, 1082, 1024, 941, 910, 857, 833, 808, 771, 732, 693, 677, 666, 629.

HRMS (APCI+) calcd. for $C_{19}H_{21}NO_3S$ ($[M+H]^+$): 344.1315, found: 344.1315.

3-((4-Methoxyphenyl)sulfonyl)-1-methyl-2-propyl-1*H*-indole (3b)

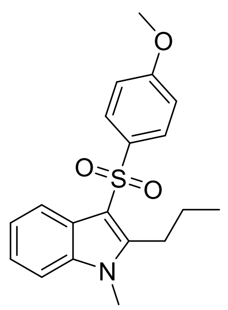

White solid. The pure form of **3b** was obtained more than 3 mg after recrystallization followed by GPC.

R_f = 0.30 (Hexane : EtOAc = 4:1)

^{1}H-NMR (400 MHz, $CDCl_3$) δ 8.12-8.09 (m, 1H), 7.91 (d, *J* = 8.9 Hz, 2H), 7.29-7.24 (m, 3H), 6.89 (d, *J* = 8.7 Hz, 2H), 3.79 (s, 3H), 3.69 (s, 3H), 3.16-3.12 (m, 2H), 1.63 (td, *J* = 15.3, 7.5 Hz, 2H), 1.07 (t, *J* = 7.3 Hz, 3H).

^{13}C-NMR (101 MHz, $CDCl_3$) δ 162.5, 145.8, 136.6, 136.3, 128.2, 124.9, 122.8, 122.2, 119.9, 114.0, 111.3, 109.5, 55.5, 29.9, 26.8, 23.1, 14.2.

IR(neat): 3096, 3069, 3054, 2962, 2932, 2872, 2841, 2570, 2060, 1907, 1712, 1594, 1577, 1515, 1496, 1469, 1396, 1340, 1323, 1308, 1292, 1256, 1227, 1182, 1136, 1120, 1082, 1019, 975, 871, 833, 803, 774, 746, 722, 684, 665, 629.

HRMS (APCI+) calcd. for $C_{19}H_{21}NO_3S$ ($[M+H]^+$): 344.13149, found: 344.13152.

6-((4-Methoxyphenyl)sulfonyl)-1-methyl-2-propyl-1*H*-indole (4b)

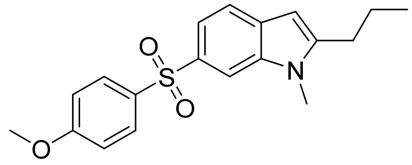

Yellow oil. The pure form of **4b** was obtained more than 3 mg after GPC.

R_f = 0.37 (Hexane : EtOAc = 4:1).

^{1}H-NMR (400 MHz, $CDCl_3$) δ 7.95 (s, 1H), 7.89 (d, *J* = 8.7 Hz, 2H), 7.54 (dd, *J* = 12.1, 8.5 Hz, 2H), 6.92 (d, *J* = 8.7 Hz, 2H), 6.30 (s, 1H), 3.81 (s, 3H), 3.73 (s, 3H), 2.73 (t, *J* = 7.5 Hz, 2H), 1.80-1.73 (m, 2H), 1.04 (t, *J* = 7.3 Hz, 3H).

^{13}C-NMR (101 MHz, $CDCl_3$) δ 162.8, 145.9, 136.2, 134.8, 133.2, 131.3, 129.4, 120.2, 118.2, 114.2, 109.0, 99.7, 55.5, 29.8, 29.0, 21.6, 13.9.

IR(neat): 3096, 3071, 2961, 2932, 2872, 2842, 1671, 1594, 1578, 1532, 1497, 1469, 1406, 1334, 1313, 1293, 1258, 1177, 1150, 1116, 1087, 1055, 1025, 913, 834, 804, 774, 719, 703, 690, 664, 609.

HRMS (APCI+) calcd. for $C_{19}H_{21}NO_3S$ ($[M+H]^+$): 344.1315, found: 344.1315.

1-Methyl-2-propyl-4-(*m*-tolylsulfonyl)-1*H*-indole (2c)

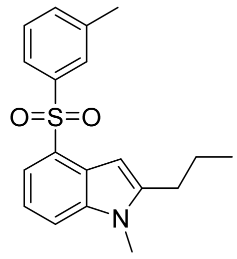

Light yellow solid. The pure form of **2c** was obtained more than 5 mg after recrystallization followed by GPC.

R_f = 0.45 (Hexane : EtOAc = 4:1).

^{1}H-NMR (400 MHz, $CDCl_3$) δ 7.71-7.90 (3H), 7.41-7.51 (1H), 7.19-7.40 (4H), 6.65-6.92 (1H), 3.60-3.69 (3H), 2.66-2.80 (2H), 2.29-2.46 (3H), 1.69-1.87 (2H), 0.96-1.12 (3H).

^{13}C-NMR (101 MHz, $CDCl_3$) δ 144.6, 142.2, 139.0, 138.3, 133.4, 130.0, 128.7, 127.5, 124.7, 124.3, 120.8, 119.7, 114.0, 98.8, 29.7, 28.9, 21.6, 21.3, 13.9.

IR(neat): 3063, 2952, 2926, 2868, 1889, 1834, 1731, 1660, 1603, 1560, 1531, 1475, 1455, 1431, 1405, 1374, 1363, 1347, 1315, 1292, 1276, 1222, 1203, 1156, 1141, 1121, 1083, 1059, 998, 944, 908, 877, 864, 795, 768, 731, 703, 689, 618, 602.

HRMS (APCI+) calcd. for $C_{19}H_{21}NO_2S$ ($[M+H]^+$): 328.1366, found: 328.1366.

1-Methyl-2-propyl-3-(*m*-tolylsulfonyl)-1*H*-indole (3c)

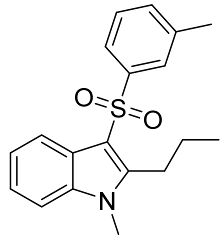

White solid. The pure form of **3c** was obtained more than 5 mg after recrystallization followed by GPC.

R_f = 0.48 (Hexane : EtOAc = 4:1).

^{1}H-NMR (400 MHz, $CDCl_3$) δ 8.15-8.11 (m, 1H), 7.78-7.75 (m, 2H), 7.32-7.24 (m, 5H), 3.70 (s, 3H), 3.17-3.13 (m, 2H), 2.36 (s, 3H), 1.67-1.57 (m, 2H), 1.07 (t, J = 7.3 Hz, 3H).

^{13}C-NMR (101 MHz, $CDCl_3$) δ 146.2, 144.4, 139.0, 136.3, 133.0, 128.7, 126.4, 125.1, 123.3, 122.8, 122.3, 120.0, 110.7, 109.5, 29.9, 26.9, 23.1, 21.4, 14.2.

IR(neat): 3054, 2962, 2931, 2872, 1731, 1600, 1513, 1471, 1397, 1339, 1324, 1294, 1249, 1226, 1182, 1135, 1119, 1081, 1018, 976, 861, 775, 746, 699, 687, 603.

HRMS (APCI+) calcd. for $C_{19}H_{21}NO_2S$ ($[M+H]^+$): 328.1366, found: 328.1366.

1-Methyl-2-propyl-6-(*m*-tolylsulfonyl)-1*H*-indole e (4c)

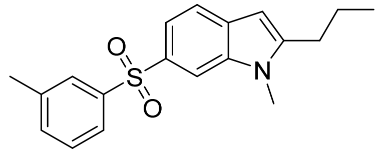

Yellow oil. The pure form of **4c** was obtained more than 3 mg after GPC.

R_f = 0.50 (Hexane : EtOAc = 4:1).

^{1}H-NMR (400 MHz, $CDCl_3$) δ 7.96 (s, 1H), 7.75 (d, *J* = 6.4 Hz, 2H), 7.56 (s, 2H), 7.35-7.27 (m, 2H), 6.30 (s, 1H), 3.73 (s, 3H), 2.73 (t, *J* = 7.6 Hz, 2H), 2.37 (s, 3H), 1.75 (td, *J* = 15.0, 7.6 Hz, 2H), 1.04 (t, *J* = 7.3 Hz, 3H).

^{13}C-NMR (101 MHz, $CDCl_3$) δ 146.0, 142.9, 139.2, 136.2, 133.2, 132.5, 131.5, 128.9, 127.5, 124.4, 120.2, 118.4, 109.3, 99.7, 29.8, 29.0, 21.6, 21.3, 13.9.

IR(neat): 3061, 2960, 2931, 2872, 1731, 1680, 1603, 1531, 1470, 1405, 1334, 1314, 1294, 1222, 1149, 1115, 1083, 1055, 862, 818, 776, 704, 688, 626.

HRMS (APCI+) calcd. for $C_{19}H_{21}NO_2S$ ($[M+H]^+$): 328.1366, found: 328.1367.

1-Methyl-2-propyl-4-(*o*-tolylsulfonyl)-1*H*-indole (2d)

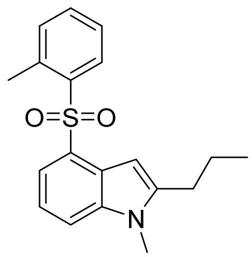

White solid. The pure form of **2d** was obtained more than 5 mg after recrystallization followed by GPC.

R_f = 0.50 (Hexane : EtOAc = 4:1).

^{1}H-NMR (400 MHz, $CDCl_3$) δ 8.30 (dd, *J* = 7.6, 2.1 Hz, 1H), 7.81 (d, *J* = 7.8 Hz, 1H), 7.47 (d, *J* = 7.8 Hz, 1H), 7.43-7.36 (m, 2H), 7.24 (t, *J* = 7.8 Hz, 1H), 7.16-7.14 (m, 1H), 6.48 (s, 1H), 3.66 (s, 3H), 2.66 (t, *J* = 7.8 Hz, 2H), 2.42 (s, 3H), 1.70 (td, *J* = 15.0, 7.6 Hz, 2H), 0.99 (t, *J* = 7.6 Hz, 3H).

^{13}C-NMR (101 MHz, $CDCl_3$) δ 144.4, 139.8, 138.3, 138.0, 132.9, 132.5, 129.3, 129.2, 126.0, 124.6, 121.2, 119.2, 114.0, 98.3, 29.7, 28.9, 21.5, 20.1, 13.9.

IR(neat): 3103, 3059, 2968, 2952, 2931, 2870, 2834, 1894, 1840, 1787, 1748, 1665, 1604, 1531, 1453, 1427, 1407, 1373, 1340, 1302, 1275, 1203, 1155, 1146, 1060, 944, 909, 857, 809, 766, 733, 711, 702, 617, 608.

HRMS (APCI+) calcd. for $C_{19}H_{21}NO_2S$ ($[M+H]^+$): 328.1366, found: 328.1366.

1-Methyl-2-propyl-3-(o-tolylsulfonyl)-1H-indole (3d)

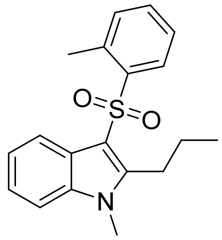

White solid. The pure form of **3d** was obtained more than 3 mg after recrystallization followed by GPC.

R_f = 0.49 (Hexane : EtOAc = 4:1).

^{1}H-NMR (400 MHz, $CDCl_3$) δ 8.12 (dd, *J* = 8.0, 1.1 Hz, 1H), 7.97 (d, *J* = 7.8 Hz, 1H), 7.39 (td, *J* = 7.6, 1.4 Hz, 1H), 7.33-7.17 (m, 5H), 3.71 (s, 3H), 3.04-3.00 (m, 2H), 2.48 (s, 3H), 1.49-1.39 (m,

2H), 0.96 (t, J = 7.3 Hz, 3H).

^{13}C-NMR (101 MHz, $CDCl_3$) δ 146.3, 142.2, 137.8, 136.0, 132.4, 132.4, 127.9, 125.9, 125.5, 122.7, 122.2, 120.1, 109.6, 109.5, 29.9, 26.9, 22.6, 20.0, 14.1.

IR(neat): 3056, 3020, 2960, 2926, 2871, 2854, 1681, 1595, 1574, 1510, 1469, 1399, 1338, 1320, 1287, 1247, 1227, 1200, 1180, 1145, 1128, 1113, 1080, 1059, 1017, 976, 920, 870, 805, 745, 711, 693, 664, 613.

HRMS (APCI+) calcd. for $C_{19}H_{21}NO_2S$ ([M+H]$^+$): 328.1366, found: 328.1366.

1-Methyl-2-propyl-4-(*o*-tolylsulfonyl)-1*H*-indole (4d)

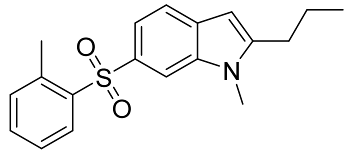

Yellow oil. The pure form of **4d** was obtained more than 3 mg after recrystallization followed by GPC.

R_f = 0.58 (Hexane : EtOAc = 4:1).

^{1}H-NMR (400 MHz, $CDCl_3$) δ 8.18 (d, J = 7.8 Hz, 1H), 7.93 (s, 1H), 7.55 (d, J = 8.2 Hz, 1H), 7.45-7.33 (m, 3H), 7.18 (d, J = 7.3 Hz, 1H), 6.32 (s, 1H), 3.72 (s, 3H), 2.73 (t, J = 7.6 Hz, 2H), 2.49 (s, 3H), 1.76 (td, J = 15.0, 7.5 Hz, 2H), 1.05 (t, J = 7.3 Hz, 3H).

^{13}C-NMR (101 MHz, $CDCl_3$) δ 146.0, 140.2, 137.6, 136.0, 132.9, 132.5, 132.0, 131.4, 128.9, 126.2, 119.9, 118.4, 109.4, 99.7, 29.8, 29.0, 21.6, 20.2, 13.9.

IR(neat): 3060, 3021, 2961, 2933, 2872, 1734, 1673, 1604, 1569, 1532, 1469, 1404, 1335, 1305, 1291, 1232, 1199, 1152, 1136, 1111, 1066, 872, 806, 760, 714, 699, 625.

HRMS (APCI+) calcd. for $C_{19}H_{21}NO_2S$ ([M+H]$^+$): 328.1366, found: 328.1366.

4-((4-Fluorophenyl)sulfonyl)-1-methyl-2-propyl-1H-indole (2e)

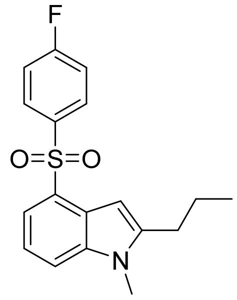

Colorless crystal. 220.7 mg (67% yield, 0.67 mmol).

R_f = 0.45 (Hexane : EtOAc = 4:1)

^{1}H-NMR (400 MHz, $CDCl_3$) δ 7.99 (qd, J = 4.8, 2.5 Hz, 2H), 7.79 (d, J = 7.3 Hz, 1H), 7.45 (d, J = 8.2 Hz, 1H), 7.23 (q, J = 7.8 Hz, 1H), 7.10-7.06 (m, 2H), 6.71 (s, 1H), 3.65 (s, 3H), 2.71 (t, J = 7.8 Hz, 2H), 1.76 (td, J = 15.1, 7.3 Hz, 2H), 1.04 (t, J = 7.3 Hz, 3H).

^{13}C-NMR (101 MHz, $CDCl_3$) δ 166.3, 163.7, 144.9, 138.6, 138.5, 138.3, 129.9, 129.8, 129.6, 124.6, 120.7, 119.7, 116.1, 115.9, 114.2, 98.5, 29.7, 28.9, 21.6, 13.9.

^{19}F-NMR (565 MHz, $CDCl_3$) δ -106.5 (m)

IR(neat): 3106, 3067, 2974, 2957, 2933, 2871, 2840, 1588, 1536, 1490, 1455, 1428, 1403, 1369, 1340, 1316, 1284, 1227, 1158, 1145, 1095, 1079, 940, 843, 819, 804, 767, 736, 712, 692, 664, 621.

HRMS (APCI+) calcd. for $C_{18}H_{18}FNO_2S$ ($[M+H]^+$): 332.1115, found: 332.1115.

3-((4-Fluorophenyl)sulfonyl)-1-methyl-2-propyl-1*H*-indole (3e)

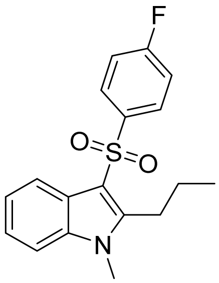

White solid. The pure form of **3e** was obtained more than 3 mg after recrystallization followed by GPC.

R_f = 0.47 (Hexane : EtOAc = 4:1)

^{1}H-NMR (400 MHz, $CDCl_3$) δ 8.11-8.07 (m, 1H), 7.97 (tt, *J* = 7.3, 2.3 Hz, 2H), 7.32-7.24 (m, 3H), 7.12-7.06 (m, 2H), 3.70 (s, 3H), 3.15-3.11 (m, 2H), 1.68-1.59 (m, 2H), 1.07 (t, *J* = 7.6 Hz, 3H). ^{13}C-NMR (101 MHz, $CDCl_3$) δ 166.0, 163.5, 146.3, 140.7, 136.4, 128.8, 128.7, 124.9, 123.0, 122.4, 119.8, 116.1, 115.9, 110.3, 109.6, 30.0, 26.8, 23.1, 14.2.

^{19}F-NMR (565 MHz, $CDCl_3$) δ -107.2 (m)

IR(neat): 3101, 3070, 2962, 2933, 2874, 1590, 1514, 1493, 1472, 1398, 1325, 1307, 1288, 1228, 1183, 1141, 1081, 1018, 977, 838, 746, 665. HRMS (APCI+) calcd. for $C_{18}H_{18}FNO_2S$ ($[M+H]^+$): 332.1115, found: 332.1115.

6-((4-Fluorophenyl)sulfonyl)-1-methyl-2-propyl-1*H*-indole (4e)

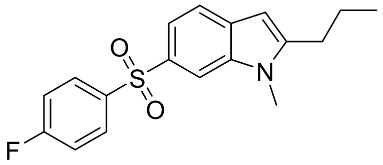

Light yellow solid. The pure form of **4e** was obtained more than 5 mg after recrystallization followed by GPC.

R_f = 0.51 (Hexane : EtOAc = 4:1)

^{1}H-NMR (400 MHz, $CDCl_3$) δ 7.98-7.94 (m, 3H), 7.59-7.52 (m, 2H), 7.14-7.10 (m, 2H), 6.32 (s, 1H), 3.74 (s, 3H), 2.73 (t, *J* = 7.6 Hz, 2H), 1.76 (td, *J* = 14.9, 7.5 Hz, 2H), 1.04 (t, *J* = 7.3 Hz, 3H). ^{13}C-NMR (101 MHz, $CDCl_3$) δ 166.2, 163.7, 146.3, 139.2, 136.2, 132.1, 131.6, 130.0, 129.9, 120.3, 118.2, 116.3, 116.1, 109.3, 99.8, 29.8, 29.0, 21.6, 13.9.

^{19}F-NMR (565 MHz, $CDCl_3$) δ -106.6 (s)

IR(neat): 3104, 3070, 2962, 2934, 2873, 1590, 1532, 1493, 1470, 1406, 1314, 1289, 1234, 1153, 1116, 1085, 1055, 837, 816, 714, 691, 665, 607.

HRMS (APCI+) calcd. for $C_{18}H_{18}FNO_2S$ ($[M+H]^+$): 332.1115, found: 332.1115.

4-((4-Chlorophenyl)sulfonyl)-1-methyl-2-propyl-1*H*-indole (2f)

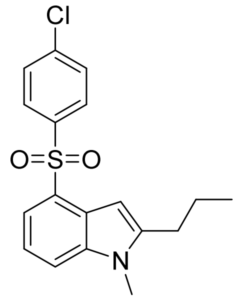

Light yellow solid. The pure form of **2f** was obtained more than 5 mg after recrystallization followed by GPC.

R_f = 0.56 (Hexane : EtOAc = 4:1).

^{1}H-NMR (400 MHz, $CDCl_3$) δ 7.93 (d, *J* = 8.7 Hz, 2H), 7.80 (d, *J* = 7.8 Hz, 1H), 7.47 (d, *J* = 7.8 Hz, 1H), 7.39 (d, *J* = 8.2 Hz, 2H), 7.26-7.21 (m, 1H), 6.72 (s, 1H), 3.68 (s, 3H), 2.73 (t, *J* = 7.8 Hz, 2H), 1.77 (td, *J* = 14.9, 7.5 Hz, 2H), 1.05 (t, *J* = 7.3 Hz, 3H).

^{13}C-NMR (101 MHz, $CDCl_3$) δ 145.0, 141.0, 139.1, 138.3, 129.4, 129.1, 128.6, 124.7, 120.9, 119.8, 114.3, 98.6, 29.8, 29.0, 21.7, 14.0.

IR(neat): 3101, 2961, 2937, 2874, 1714, 1581, 1535, 1474, 1456, 1435, 1393, 1373, 1341, 1312, 1279, 1158, 1145, 1081, 1011, 943, 834, 807, 769, 753, 707, 690, 639, 608.

HRMS (APCI+) calcd. for $C_{18}H_{18}ClNO_2S$ ($[M+H]^+$): 348.0820, found: 348.0820.

3-((4-Chlorophenyl)sulfonyl)-1-methyl-2-propyl-1*H*-indole (3f)

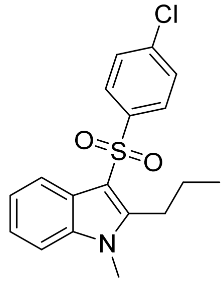

White solid. The pure form of **3f** was obtained more than 3 mg after recrystallization followed by GPC.

R_f = 0.59 (Hexane : EtOAc = 4:1).

^{1}H-NMR (400 MHz, $CDCl_3$) δ 8.09-8.07 (m, 1H), 7.90 (d, *J* = 8.7 Hz, 2H), 7.38 (d, *J* = 8.7 Hz, 2H), 7.32-7.26 (m, 3H), 3.71 (s, 3H), 3.15-3.11 (m, 2H), 1.64 (td, *J* = 15.3, 7.5 Hz, 2H), 1.07 (t, *J* = 7.3 Hz, 3H).

^{13}C-NMR (101 MHz, $CDCl_3$) δ 146.5, 143.1, 138.6, 136.4, 129.1, 127.5, 124.9, 123.1, 122.5, 119.8, 110.1, 109.7, 30.0, 26.9, 23.1, 14.2.

IR(neat): 3088, 2963, 2932, 2872, 1735, 1580, 1513, 1473, 1396, 1326, 1307, 1278, 1249, 1227, 1183, 1143, 1083, 1013, 977, 827, 777, 752, 706, 621.

HRMS (APCI+) calcd. for $C_{18}H_{18}ClNO_2S$ ($[M+H]^+$): 348.0820, found: 348.0820.

6-((4-Chlorophenyl)sulfonyl)-1-methyl-2-propyl-1*H*-indole (4f)

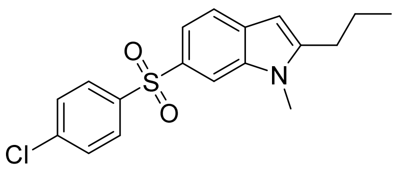

Yellow oil. The pure form of **4f** was obtained more than 3 mg after recrystallization followed by GPC.

R_f = 0.65 (Hexane : EtOAc = 4:1).

^{1}H-NMR (400 MHz, $CDCl_3$) δ 7.94 (s, 1H), 7.88 (d, *J* = 8.7 Hz, 2H), 7.55 (q, *J* = 8.5 Hz, 2H), 7.41 (d, *J* = 8.7 Hz, 2H), 6.31 (s, 1H), 3.74 (s, 3H), 2.73 (t, *J* = 7.8 Hz, 2H), 1.75 (td, *J* = 14.9, 7.3 Hz, 2H), 1.09-1.02 (m, 3H).

^{13}C-NMR (101 MHz, $CDCl_3$) δ 146.4, 141.6, 139.0, 136.2, 131.8, 131.7, 129.3, 128.7, 120.3, 118.3, 109.3, 99.8, 29.8, 29.0, 21.6, 13.9.

IR(neat): 3088, 3066, 2961, 2934, 2873, 1604, 1581, 1532, 1473, 1394, 1334, 1313, 1295, 1278, 1226, 1154, 1116, 1086, 1055, 1012, 826, 770, 753, 709, 674, 639.

HRMS (APCI+) calcd. for $C_{18}H_{18}ClNO_2S$ ($[M+H]^+$): 348.0820, found: 348.0820.

1-Methyl-2-propyl-4-((4-(trifluoromethyl)phenyl)sulfonyl)-1*H*-indole (2g)

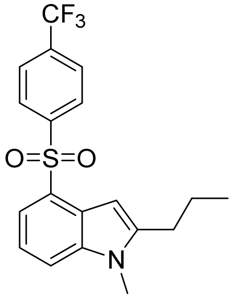

White solid. The pure form of **2g** was obtained more than 5 mg after recrystallization followed by GPC.

R_f = 0.50 (Hexane : EtOAc = 4:1).

^{1}H-NMR (400 MHz, $CDCl_3$) δ 8.12 (d, *J* = 8.2 Hz, 2H), 7.84 (d, *J* = 7.8 Hz, 1H), 7.69 (d, *J* = 8.2 Hz, 2H), 7.49 (d, *J* = 8.2 Hz, 1H), 7.26 (t, *J* = 7.8 Hz, 1H), 6.74 (s, 1H), 3.69 (s, 3H), 2.73 (t, *J* = 7.6 Hz, 2H), 1.78 (td, *J* = 15.1, 7.5 Hz, 2H), 1.05 (t, *J* = 7.3 Hz, 3H). ^{13}C-NMR (101 MHz, $CDCl_3$) δ 146.0, 145.2, 138.4, 134.3, 134.0, 128.6, 127.6, 126.0, 126.0, 124.9, 121.2, 119.8, 114.6, 98.5, 77.3, 77.0, 76.7, 29.8, 29.0, 21.7, 14.0.

^{19}F-NMR (565 MHz, $CDCl_3$) δ -64.1 (s).

IR(neat): 3099, 3066, 2963, 2935, 2874, 1682, 1606, 1561, 1536, 1454, 1428, 1402, 1320, 1278, 1160, 1130, 1107, 1083, 1062, 1015, 941, 843, 809, 773, 735, 714, 665, 626, 606.

HRMS (APCI+) calcd. for $C_{19}H_{18}F_3NO_2S$ ($[M+H]^+$): 382.1083, found: 382.1084.

1-Methyl-2-propyl-3-((4-(trifluoromethyl)phenyl)sulfonyl)-1*H*-indole (3g)

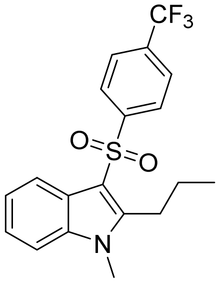

White solid. The pure form of **3g** was obtained more than 3 mg after recrystallization followed by GPC.

R_f = 0.50 (Hexane : EtOAc = 4:1).

^{1}H-NMR (400 MHz, $CDCl_3$) δ 8.12-8.07 (m, 3H), 7.68 (d, *J* = 8.7 Hz, 2H), 7.33-7.27 (m, 3H), 3.72 (s, 3H), 3.16-3.12 (m, 2H), 1.71-1.62 (m, 2H), 1.08 (t, *J* = 7.3 Hz, 3H).

^{13}C-NMR (101 MHz, $CDCl_3$) δ 148.0, 147.0, 136.5, 134.0, 133.7, 126.5, 126.1, 126.0, 124.9, 123.2, 122.7, 119.8, 109.8, 109.5, 30.1, 26.9, 23.2, 14.2.

^{19}F-NMR (565 MHz, $CDCl_3$) δ -64.0 (s).

IR(neat): 3099, 3071, 3053, 2964, 2935, 2875, 1607, 1510, 1471, 1400, 1320, 1302, 1250, 1221, 1171, 1131, 1106, 1083, 1062, 1016, 977, 909, 843, 773, 746, 712, 666, 615.

HRMS (APCI+) calcd. for $C_{19}H_{18}F_3NO_2S$ ($[M+H]^+$): 382.1083, found: 382.1083.

1-Methyl-2-propyl-6-((4-(trifluoromethyl)phenyl)sulfonyl)-1*H*-indole (4g)

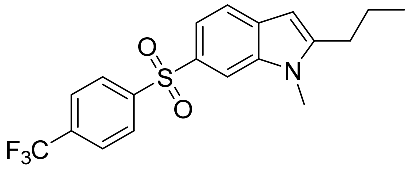

Yellow solid. The pure form of **4g** was obtained more than 5 mg after recrystallization followed by GPC.

R_f = 0.59 (Hexane : EtOAc = 4:1).

^{1}H-NMR (400 MHz, $CDCl_3$) δ 8.07 (d, *J* = 8.2 Hz, 2H), 7.96 (s, 1H), 7.71 (d, *J* = 8.2 Hz, 2H), 7.61-7.55 (m, 2H), 6.33 (s, 1H), 3.75 (s, 3H), 2.74 (t, *J* = 7.6 Hz, 2H), 1.76 (td, *J* = 14.9, 7.5 Hz, 2H), 1.05 (t, *J* = 7.6 Hz, 3H).

^{13}C-NMR (101 MHz, $CDCl_3$) δ 146.6, 136.3, 134.2, 133.9, 132.0, 131.1, 127.7, 126.2, 126.2, 120.5, 118.4, 109.6, 99.9, 29.9, 29.0, 21.6, 13.9.

^{19}F-NMR (565 MHz, $CDCl_3$) δ -64.0 (s).

IR(neat): 3099, 3065, 2963, 2935, 2874, 1604, 1531, 1471, 1403, 1320, 1293, 1227, 1155, 1131, 1106, 1086, 1061, 1014, 909, 843, 818, 795, 736, 714, 672, 632.

HRMS (APCI+) calcd. for $C_{19}H_{18}F_3NO_2S$ ($[M+H]^+$): 382.1083, found: 382.1084.

1-Methyl-4-((4-nitrophenyl)sulfonyl)-2-propyl-1*H*-indole (2h)

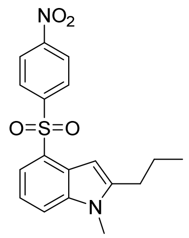

Yellow solid. The pure form of **2h** was obtained more than 5 mg after recrystallization followed by GPC.

R_f = 0.40 (Hexane : EtOAc = 4:1)

^{1}H-NMR (400 MHz, $CDCl_3$) δ 8.25 (d, J = 8.7 Hz, 2H), 8.15 (d, J = 8.7 Hz, 2H), 7.83 (d, J = 7.8 Hz, 1H), 7.51 (d, J = 8.2 Hz, 1H), 7.26 (t, J = 8.0 Hz, 1H), 6.72 (s, 1H), 3.69 (s, 3H), 2.74 (t, J = 7.6 Hz, 2H), 1.78 (td, J = 14.9, 7.5 Hz, 2H), 1.05 (t, J = 7.3 Hz, 3H).

^{13}C-NMR (101 MHz, $CDCl_3$) δ 149.9, 148.1, 145.5, 138.4, 128.3, 127.9, 125.0, 124.1, 121.4, 119.9, 114.9, 98.4, 29.8, 28.9, 21.7, 14.0.

IR(neat): 3840, 3103, 3066, 2959, 2929, 2871, 1706, 1605, 1530, 1453, 1427, 1399, 1349, 1307, 1274, 1159, 1146, 1105, 1080, 1011, 944, 855, 807, 768, 736, 689, 634, 616.

HRMS (APCI+) calcd. for $C_{18}H_{18}N_2O_4S$ ($[M+H]^+$): 359.1060, found: 359.1060.

1-Methyl-3-((4-nitrophenyl)sulfonyl)-2-propyl-1H-indole (3h)

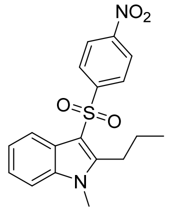

Yellow solid. The pure form of **3h** was obtained more than 3 mg after recrystallization followed by GPC.

R_f = 0.40 (Hexane : EtOAc = 4:1)

^{1}H-NMR (400 MHz, $CDCl_3$) δ 8.26 (d, J = 8.7 Hz, 2H), 8.13 (d, J = 8.7 Hz, 2H), 8.10-8.08 (m, 1H), 7.32-7.29 (m, 3H), 3.74 (s, 3H), 3.14 (t, J = 8.0 Hz, 2H), 1.67 (td, J = 15.3, 7.5 Hz, 2H), 1.09 (t, J = 7.3 Hz, 3H).

^{13}C-NMR (101 MHz, $CDCl_3$) δ 150.1, 149.7, 147.4, 136.5, 127.2, 124.9, 124.2, 123.4, 122.9, 119.6, 109.9, 108.9, 30.2, 26.9, 23.2, 14.2.

IR(neat): 3102, 3070, 3033, 2962, 2928, 2872, 1715, 1605, 1528, 1472, 1398, 1347, 1301, 1227, 1183, 1143, 1082, 1013, 977, 854, 737, 685, 618.

HRMS (APCI+) calcd. for $C_{18}H_{18}N_2O_4S$ ($[M+H]^+$): 359.1060, found: 359.1060.

1-Methyl-6-((4-nitrophenyl)sulfonyl)-2-propyl-1H-indole (4h)

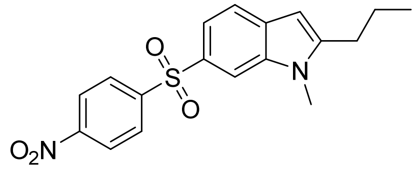

Yellow oil. The pure form of **4h** was obtained more than 3 mg after recrystallization followed by GPC.

R_f = 0.50 (Hexane : EtOAc = 4:1)

^{1}H-NMR (400 MHz, $CDCl_3$) δ 8.29 (d, J = 8.7 Hz, 2H), 8.12 (d, J = 9.2 Hz, 2H), 7.96 (s, 1H), 7.62-7.55 (m, 2H), 6.34 (s, 1H), 3.76 (s, 3H), 2.75 (t, J = 7.6 Hz, 2H), 1.77 (td, J = 15.0, 7.5 Hz, 2H), 1.05 (t, J = 7.3 Hz, 3H). ^{13}C-NMR (101 MHz, $CDCl_3$) δ 149.8, 148.9, 147.0, 136.3, 132.2, 130.3, 128.5, 124.3, 120.6, 118.5, 109.8, 100.0, 29.9, 29.0, 21.6, 13.9. IR(neat): 3103, 3065, 3035, 2961, 2931, 2872, 1714, 1604, 1528, 1471, 1403, 1347, 1307, 1226, 1154, 1116, 1084, 1054, 1012, 854, 818, 736, 687, 634.

HRMS (APCI+) calcd. for $C_{18}H_{18}N_2O_4S$ ([M+H]$^+$): 359.1060, found: 359.1060.

1-Methyl-4-(methylsulfonyl)-2-propyl-1*H*-indole (2i)

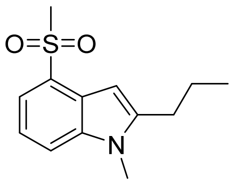

Yellow solid. The pure form of **2i** was obtained more than 3 mg after recrystallization followed by GPC.

R_f = 0.34 (Hexane : EtOAc = 4:1)

^{1}H-NMR (400 MHz, $CDCl_3$) δ 7.71 (d, J = 7.3 Hz, 1H), 7.53 (d, J = 8.2 Hz, 1H), 7.25 (t, J = 7.8 Hz, 1H), 6.71 (s, 1H), 3.74 (s, 3H), 3.11 (s, 3H), 2.76 (t, J = 7.6 Hz, 2H), 1.81 (td, J = 15.0, 7.6 Hz, 2H), 1.07 (t, J = 7.3 Hz, 3H). ^{13}C-NMR (101 MHz, $CDCl_3$) δ 144.9, 138.3, 129.2, 124.7, 120.3, 119.6, 114.3, 98.2, 43.6, 29.8, 29.0, 21.7, 14.0. IR(neat): 3063, 3014, 2961, 2930, 2872, 1536, 1455, 1429, 1405, 1348, 1303, 1158, 1143, 1114, 963, 809, 755.

HRMS (APCI+) calcd. for $C_{13}H_{17}NO_2S$ ([M+H]$^+$): 252.1053, found: 252.1053.

1-Methyl-3-(methylsulfonyl)-2-propyl-1*H*-indole (3i)

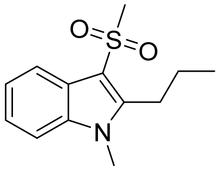

Light yellow solid. The pure form of **3i** was obtained more than 3 mg after recrystallization followed by GPC.

R_f = 0.35 (Hexane : EtOAc = 4:1)

^{1}H-NMR (400 MHz, $CDCl_3$) δ 7.99 (dd, J = 6.9, 1.8 Hz, 1H), 7.32 (tdd, J = 14.0, 7.0, 1.5 Hz, 3H), 3.75 (s, 3H), 3.18-3.14 (m, 2H), 3.11 (s, 3H), 1.72 (td, J = 15.3, 7.5 Hz, 2H), 1.08 (t, J = 7.3 Hz, 3H). ^{13}C-NMR (101 MHz, $CDCl_3$) δ 146.1, 136.3, 124.9, 123.0, 122.3, 119.5, 109.6, 45.9, 29.9, 26.4, 23.3, 14.1. IR(neat): 3053, 3019, 2962, 2929, 2872, 1731, 1608, 1519, 1471, 1397, 1340, 1320, 1295, 1250, 1228, 1183, 1132, 1111, 1089, 1018, 983, 946, 780, 751.

HRMS (APCI+) calcd. for $C_{13}H_{17}NO_2S$ ([M+H]$^+$): 252.1053, found: 252.1053.

1-Methyl-6-(methylsulfonyl)-2-propyl-1*H*-indole (4i)

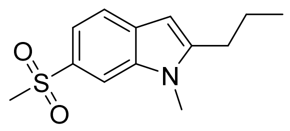

Brown solid. The pure form of **4i** was obtained more than 3 mg after recrystallization followed by GPC.

R_f = 0.29 (Hexane : EtOAc = 4:1)

^{1}H-NMR (400 MHz, $CDCl_3$) δ 7.91 (s, 1H), 7.65 (d, *J* = 8.2 Hz, 1H), 7.59-7.57 (m, 1H), 7.26 (s, 0H), 6.36 (s, 1H), 3.75 (s, 3H), 3.08 (s, 3H), 2.76 (t, *J* = 7.6 Hz, 2H), 1.79 (td, *J* = 15.1, 7.3 Hz, 2H), 1.07 (t, *J* = 7.2 Hz, 3H).

^{13}C-NMR (101 MHz, $CDCl_3$) δ 146.2, 136.1, 131.8, 131.7, 120.2, 117.6, 109.1, 99.8, 77.3, 77.0, 76.7, 45.3, 29.8, 29.0, 21.6, 13.9.

IR(neat): 3052, 3019, 2961, 2928, 2873, 1715, 1605, 1533, 1470, 1405, 1334, 1293, 1226, 1148, 1109, 1060, 961, 933, 869, 821, 756, 613.

HRMS (APCI+) calcd. for $C_{13}H_{17}NO_2S$ ($[M+H]^+$): 252.1053, found: 252.1053.

4-((4-Fluorophenyl)sulfonyl)-2-hexyl-1-methyl-1H-indole (2j)

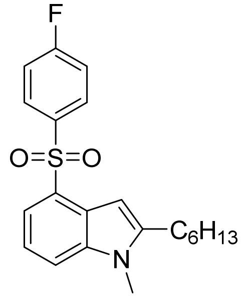

Light yellow solid. The pure form of **2j** was obtained more than 5 mg after recrystallization followed by GPC.

R_f = 0.54 (Hexane : EtOAc = 4:1).

^{1}H-NMR (400 MHz, $CDCl_3$) δ 8.01 (q, *J* = 4.6 Hz, 2H), 7.80 (d, *J* = 7.3 Hz, 1H), 7.47 (d, *J* = 8.2 Hz, 1H), 7.22 (d, *J* = 8.2 Hz, 1H), 7.10 (t, *J* = 8.7 Hz, 2H), 6.72 (s, 1H), 3.68 (s, 3H), 2.74 (t, *J* = 7.8 Hz, 2H), 1.77-1.70 (m, 2H), 1.44-1.33 (m, 6H), 0.92 (t, *J* = 6.9 Hz, 3H).

^{13}C-NMR (101 MHz, $CDCl_3$) δ 166.3, 163.8, 145.1, 138.5, 138.3, 129.9, 129.8, 129.6, 124.7, 120.8, 119.7, 116.2, 116.0, 114.2, 98.4, 31.5, 29.8, 29.1, 28.3, 26.9, 22.6, 14.1.

^{19}F-NMR (565 MHz, $CDCl_3$) δ -107.2 (m)

IR(neat): 3102, 3069, 2952, 2929, 2858, 1905, 1590, 1562, 1537, 1492, 1455, 1428, 1403, 1347, 1316, 1288, 1235, 1161, 1146, 1124, 1080, 940, 837, 819, 772, 734, 712, 694, 679.

HRMS (APCI+) calcd. for $C_{21}H_{24}FNO_2S$ ($[M+H]^+$): 374.1585, found: 374.1585.

3-((4-Fluorophenyl)sulfonyl)-2-hexyl-1-methyl-1*H*-indole (3j)

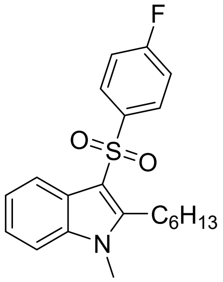

White solid. The pure form of **3j** was obtained more than 3 mg after recrystallization followed by GPC.

R_f = 0.62 (Hexane : EtOAc = 4:1).

^{1}H-NMR (400 MHz, $CDCl_3$) δ 8.11-8.09 (m, 1H), 7.99-7.96 (m, 2H), 7.31-7.25 (m, 3H), 7.11-7.07 (m, 2H), 3.70 (s, 3H), 3.14 (t, J = 8.3 Hz, 2H), 1.60-1.53 (m, 2H), 1.49-1.42 (m, 2H), 1.32 (td, J = 7.1, 3.7 Hz, 4H), 0.90 (t, J = 7.1 Hz, 3H).

^{13}C-NMR (101 MHz, $CDCl_3$) δ 166.0, 163.5, 146.6, 140.8, 136.3, 128.8, 128.7, 125.0, 123.0, 122.4, 119.8, 116.1, 115.9, 110.2, 109.6, 31.5, 30.0, 29.6, 29.5, 25.0, 22.5, 14.0.

IR(neat): 3100, 3070, 2929, 2858, 1901, 1774, 1590, 1513, 1493, 1470, 1397, 1325, 1307, 1287, 1233, 1178, 1138, 1122, 1080, 1017, 975, 835, 819, 744, 710, 686, 665, 652.

^{19}F-NMR (565 MHz, $CDCl_3$) δ -106.6 (s)

HRMS (APCI+) calcd. for $C_{21}H_{24}FNO_2S$ ($[M+H]^+$): 374.1585, found: 374.1585.

6-((4-Fluorophenyl)sulfonyl)-2-hexyl-1-methyl-1*H*-indole (4j)

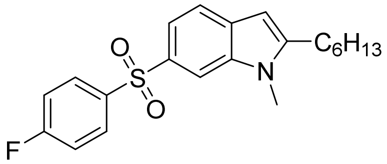

Yellow oil. The pure form of **4j** was obtained more than 3 mg after GPC.

R_f = 0.62 (Hexane : EtOAc = 4:1).

^{1}H-NMR (400 MHz, $CDCl_3$) δ 7.96 (qd, J = 4.8, 2.6 Hz, 3H), 7.59-7.52 (m, 2H), 7.15-7.09 (m, 2H), 6.31 (s, 1H), 3.74 (s, 3H), 2.74 (t, J = 7.8 Hz, 2H), 1.75-1.67 (m, 2H), 1.44-1.31 (m, 6H), 0.90 (t, J = 6.9 Hz, 3H).

^{13}C-NMR (101 MHz, $CDCl_3$) δ 166.2, 163.7, 146.5, 139.2, 139.1, 136.2, 132.0, 131.6, 130.0, 129.9, 120.3, 118.2, 116.3, 116.1, 109.2, 99.6, 31.5, 29.8, 29.0, 28.3, 26.9, 22.5, 14.0.

^{19}F-NMR (565 MHz, $CDCl_3$) δ -106.5 - -106.6 (m).

IR(neat): 3102, 3070, 2953, 2929, 2858, 1901, 1590, 1531, 1493, 1469, 1405, 1335, 1314, 1289, 1234, 1151, 1117, 1085, 1055, 1012, 933, 837, 816, 774, 714, 691, 665, 623, 607.

HRMS (APCI+) calcd. for $C_{21}H_{24}FNO_2S$ ($[M+H]^+$): 374.1585, found: 374.1585.

4-((4-Fluorophenyl)sulfonyl)-2-cyclohexyl-1-methyl-1*H*-indole (2k)

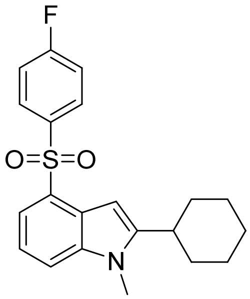

Light yellow solid. The pure form of **2k** was obtained more than 5 mg after recrystallization followed by GPC.

R_f = 0.47 (Hexane : EtOAc = 4:1).

^{1}H-NMR (400 MHz, $CDCl_3$) δ 8.01 (tt, *J* = 7.3, 2.3 Hz, 2H), 7.79 (d, *J* = 7.3 Hz, 1H), 7.47 (d, *J* = 7.8 Hz, 1H), 7.23 (t, *J* = 8.0 Hz, 1H), 7.13-7.07 (m, 2H), 6.72 (s, 1H), 3.70 (s, 3H), 2.69 (tt, *J* = 11.5, 3.1 Hz, 1H), 2.00 (d, *J* = 11.9 Hz, 2H), 1.90 (d, *J* = 12.4 Hz, 2H), 1.80 (d, *J* = 12.4 Hz, 1H), 1.57-1.30 (m, 5H).

^{13}C-NMR (101 MHz, $CDCl_3$) δ 166.3, 163.8, 150.1, 138.6, 138.3, 129.9, 129.8, 124.7, 120.8, 119.8, 116.2, 116.0, 114.3, 96.5, 36.1, 32.9, 29.8, 26.5, 26.0.

^{19}F-NMR (565 MHz, $CDCl_3$) δ -106.5 - -106.6 (m).

IR(neat): 3100, 3067, 2943, 2923, 2860, 2844, 1587, 1523, 1491, 1450, 1427, 1401, 1373, 1348, 1311, 1283, 1230, 1160, 1143, 1124, 1094, 1080, 942, 846, 825, 769, 731, 713, 684, 630.

HRMS (APCI+) calcd. for $C_{21}H_{22}FNO_2S$ ($[M+H]^+$): 372.1428, found: 372.1428.

3-((4-Fluorophenyl)sulfonyl)-2-cyclohexyl-1-methyl-1*H*-indole (3k)

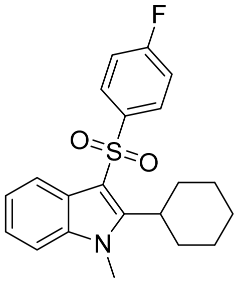

White solid. The pure form of **3k** was obtained more than 3 mg after recrystallization followed by GPC.

R_f = 0.53 (Hexane : EtOAc = 4:1).

^{1}H-NMR (400 MHz, $CDCl_3$) δ 8.23-8.21 (m, 1H), 7.94 (tt, *J* = 7.3, 2.2 Hz, 2H), 7.31-7.25 (m, 3H), 7.12-7.06 (m, 2H), 4.02 (s, 1H), 3.87 (d, *J* = 15.1 Hz, 3H), 1.89-1.23 (m, 10H).

^{13}C-NMR (101 MHz, $CDCl_3$) δ 166.0, 163.4, 149.1, 141.1, 141.0, 136.8, 128.7, 128.6, 125.0, 123.0, 122.4, 120.3, 116.1, 115.9, 110.6, 109.4, 35.9, 32.6, 30.0, 26.7, 25.8.

^{19}F-NMR (565 MHz, $CDCl_3$) δ -107.2 (s).

IR(neat): 3104, 3072, 2930, 2855, 2254, 1901, 1590, 1493, 1471, 1449, 1398, 1360, 1329, 1306, 1287, 1233, 1190, 1157, 1141, 1095, 1080, 1020, 972, 908, 878, 835, 819, 796, 786, 744, 731, 710, 665, 633.

HRMS (APCI+) calcd. for $C_{21}H_{22}FNO_2S$ ($[M+H]^+$): 372.1428, found: 372.1428.

6-((4-Fluorophenyl)sulfonyl)-2-cyclohexyl-1-methyl-1*H*-indole (4k)

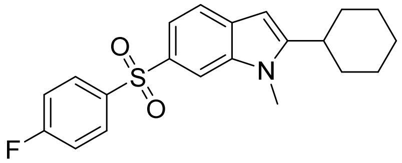

Light yellow solid. The pure form of **4k** was obtained more than 3 mg after recrystallization followed by GPC.

R_f = 0.54 (Hexane : EtOAc = 4:1).

^{1}H-NMR (400 MHz, $CDCl_3$) δ 7.96 (tt, *J* = 7.4, 2.3 Hz, 3H), 7.60-7.52 (m, 2H), 7.14-7.08 (m, 2H), 6.30 (s, 1H), 3.77 (s, 3H), 2.73-2.68 (m, 1H), 2.00 (d, *J* = 8.2 Hz, 2H), 1.88 (dd, *J* = 8.7, 3.2 Hz, 2H), 1.79 (d, *J* = 12.4 Hz, 1H), 1.50-1.38 (m, 4H), 1.34-1.27 (m, 1H).

^{13}C-NMR (101 MHz, $CDCl_3$) δ 166.2, 163.7, 151.6, 139.2, 136.1, 132.1, 131.7, 130.0, 129.9, 120.4, 118.2, 116.3, 116.1, 109.3, 97.6, 77.3, 77.0, 76.7, 36.1, 32.9, 29.8, 29.2, 26.4, 26.0.

^{19}F-NMR (565 MHz, $CDCl_3$) δ -106.6 (m).

IR(neat): 3102, 3070, 3022, 2929, 2853, 1902, 1702, 1590, 1526, 1493, 1471, 1450, 1406, 1334, 1314, 1289, 1235, 1152, 1121, 1085, 1055, 1013, 870, 838, 816, 784, 757, 721, 683, 660, 634, 608.

HRMS (APCI+) calcd. for $C_{21}H_{22}FNO_2S$ ($[M+H]^+$): 372.1428, found: 372.1428.

4-((4-Fluorophenyl)sulfonyl)-2-cyclopropyl-1-methyl-1*H*-indole (2l)

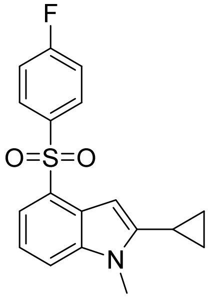

Light yellow solid. The pure form of **2l** was obtained more than 5 mg after recrystallization followed by GPC.

R_f = 0.36 (Hexane : EtOAc = 4:1).

^{1}H-NMR (400 MHz, $CDCl_3$) δ 8.01-7.97 (m, 2H), 7.79 (d, *J* = 7.3 Hz, 1H), 7.46 (d, *J* = 8.2 Hz, 1H), 7.26-7.22 (m, 1H), 7.10 (t, *J* = 8.7 Hz, 2H), 6.57 (s, 1H), 3.81 (s, 3H), 1.91-1.84 (m, 1H), 1.07-1.03 (m, 2H), 0.84-0.80 (m, 2H).

^{13}C-NMR (101 MHz, $CDCl_3$) δ 166.3, 163.8, 146.7, 138.5, 138.4, 129.9, 129.8, 124.4, 120.8, 119.9, 116.2, 116.0, 114.1, 97.0, 30.0, 7.6, 6.8.

^{19}F-NMR (565 MHz, $CDCl_3$) δ -106.4 - -106.5 (m).

IR(neat): 3100, 3073, 3011, 2943, 2871, 1590, 1560, 1543, 1493, 1453, 1427, 1402, 1373, 1353, 1316, 1289, 1234, 1161, 1146, 1080, 1056, 945, 886, 839, 772, 756, 712, 693, 674, 660.

HRMS (APCI+) calcd. for $C_{18}H_{16}FNO_2S$ ($[M+H]^+$): 330.0959, found: 330.0959.

3-((4-Fluorophenyl)sulfonyl)-2-cyclopropyl-1-methyl-1H-indole (3l)

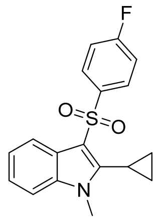

White solid. The pure form of **3l** was obtained more than 3 mg after recrystallization followed by GPC.

R_f = 0.33 (Hexane : EtOAc = 4:1).

^{1}H-NMR (400 MHz, $CDCl_3$) δ 8.22-8.19 (m, 1H), 8.01-7.96 (m, 2H), 7.32-7.28 (m, 3H), 7.13-7.07 (m, 2H), 3.84 (s, 3H), 1.95-1.88 (m, 1H), 1.22-1.17 (m, 2H), 0.86-0.80 (m, 2H).

^{13}C-NMR (101 MHz, $CDCl_3$) δ 166.0, 163.5, 145.2, 140.8, 135.8, 129.0, 128.9, 125.3, 123.3, 122.6, 120.2, 116.0, 115.8, 112.8, 109.7, 31.0, 7.8, 6.8.

^{19}F-NMR (565 MHz, $CDCl_3$) δ -107.2 (m).

IR(neat): 3102, 3073, 3019, 2952, 1589, 1542, 1515, 1494, 1469, 1435, 1389, 1361, 1339, 1318, 1305, 1287, 1228, 1173, 1160, 1137, 1098, 1081, 1031, 1017, 976, 932, 915, 836, 821, 781, 742, 703, 665.

HRMS (APCI+) calcd. for $C_{18}H_{16}FNO_2S$ ($[M+H]^+$): 330.0959, found: 330.0959.

6-((4-Fluorophenyl)sulfonyl)-2-cyclopropyl-1-methyl-1*H*-indole (4l)

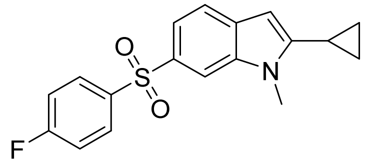

Light yellow solid. The pure form of **4l** was obtained more than 5 mg after recrystallization followed by GPC.

R_f = 0.41 (Hexane : EtOAc = 4:1).

^{1}H-NMR (400 MHz, $CDCl_3$) δ 7.96 (q, J = 4.6 Hz, 3H), 7.54 (dd, J = 13.1, 8.9 Hz, 2H), 7.12 (t, J = 8.7 Hz, 2H), 6.16 (s, 1H), 3.87 (s, 3H), 1.92-1.88 (m, 1H), 1.08-1.03 (m, 2H), 0.77 (q, J = 5.2 Hz, 2H).

^{13}C-NMR (101 MHz, $CDCl_3$) δ 148.2, 131.4, 130.0, 129.9, 120.5, 118.2, 116.4, 116.1, 109.1, 98.1, 30.1, 7.6, 6.8.

^{19}F-NMR (565 MHz, $CDCl_3$) δ -106.5 - -106.6 (m).

IR(neat): 3100, 3073, 3011, 2938, 2854, 1905, 1713, 1658, 1590, 1541, 1493, 1470, 1403, 1312, 1289, 1234, 1150, 1116, 1084, 1050, 1013, 939, 888, 836, 816, 768, 713, 693, 673, 657, 608.

HRMS (APCI+) calcd. for $C_{18}H_{16}FNO_2S$ ($[M+H]^+$): 330.0959, found: 330.0959.

4-((4-Fluorophenyl)sulfonyl)-1,2-dimethyl-1*H*-indole (2m)

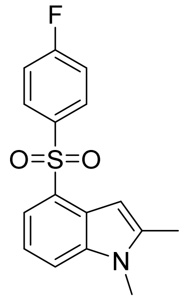

Light yellow solid. The pure form of **2m** was obtained more than 5 mg after recrystallization followed by GPC.

R_f = 0.28 (Hexane : EtOAc = 4:1).

^{1}H-NMR (400 MHz, $CDCl_3$) δ 8.00 (dd, J = 7.8, 5.5 Hz, 2H), 7.81 (d, J = 7.8 Hz, 1H), 7.45 (d, J = 8.2 Hz, 1H), 7.26-7.21 (m, 1H), 7.10 (t, J = 8.5 Hz, 2H), 6.71 (s, 1H), 3.67 (s, 3H), 2.45 (s, 3H).

^{13}C-NMR (101 MHz, $CDCl_3$) δ 166.3, 163.8, 140.5, 138.3, 129.9, 129.8, 129.6, 124.7, 120.8, 119.7, 116.2, 116.0, 114.2, 99.4, 29.7, 12.9.

^{19}F-NMR (565 MHz, $CDCl_3$) δ -106.4 - -106.5 (m).

IR(neat): 3102, 3070, 2926, 2860, 1908, 1719, 1672, 1589, 1561, 1542, 1493, 1444, 1426, 1397, 1343, 1314, 1288, 1232, 1161, 1144, 1124, 1095, 1080, 1035, 1012, 940, 838, 819, 771, 736, 712, 693, 665.

HRMS (APCI+) calcd. for $C_{16}H_{14}FNO_2S$ ($[M+H]^+$): 304.0802, found: 304.0802.

3-((4-Fluorophenyl)sulfonyl)-1,2-dimethyl-1*H*-indole (3m)

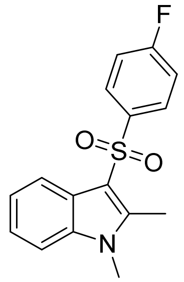

White solid. The pure form of **3m** was obtained more than 3 mg after recrystallization followed by GPC.

R_f = 0.23 (Hexane : EtOAc = 4:1).

^{1}H-NMR (400 MHz, $CDCl_3$) δ 8.10-8.08 (m, 1H), 7.99-7.95 (m, 2H), 7.29-7.24 (m, 3H), 7.12-7.07 (m, 2H), 3.68 (s, 3H), 2.74 (s, 3H).

^{13}C-NMR (101 MHz, $CDCl_3$) δ 163.5, 142.1, 140.6, 136.2, 128.7, 128.6, 124.8, 123.0, 122.4, 119.6, 116.2, 116.0, 110.9, 109.5, 29.9, 11.1.

^{19}F-NMR (565 MHz, $CDCl_3$) δ -107.1 - -107.2 (m).

IR(neat): 3101, 3070, 2925, 2854, 1715, 1590, 1520, 1493, 1472, 1397, 1337, 1323, 1308, 1287, 1238, 1185, 1157, 1138, 1094, 1080, 1036, 1016, 973, 897, 836, 818, 768, 750, 711, 686, 663, 635.

HRMS (APCI+) calcd. for $C_{16}H_{14}FNO_2S$ ($[M+H]^+$): 304.0802, found: 304.0802.

6-((4-Fluorophenyl)sulfonyl)-1,2-dimethyl-1*H*-indole (4m)

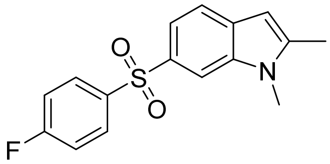

Yellow oil. The pure form of **4m** was obtained more than 3 mg after GPC.

R$_f$ = 0.33 (Hexane : EtOAc = 4:1).

^{1}H-NMR (400 MHz, $CDCl_3$) δ 7.97-7.94 (m, 3H), 7.55 (dd, *J* = 12.6, 8.5 Hz, 2H), 7.12 (t, *J* = 8.5 Hz, 2H), 6.31 (s, 1H), 3.74 (s, 3H), 2.46 (s, 3H).

^{13}C-NMR (101 MHz, $CDCl_3$) δ 166.2, 163.7, 141.9, 139.2, 136.2, 132.2, 131.7, 130.0, 129.9, 120.2, 118.3, 116.4, 116.1, 109.2, 100.7, 29.8, 13.0.

^{13}C-NMR (101 MHz, $CDCl_3$) δ 166.3, 163.8, 140.5, 138.3, 129.9, 129.8, 129.6, 124.7, 120.8, 119.7, 116.2, 116.0, 114.2, 99.4, 29.7, 12.9.

^{19}F-NMR (565 MHz, $CDCl_3$) δ -106.5 - -106.6 (m).

IR(neat): 3102, 3070, 2926, 2856, 1719, 1677, 1590, 1541, 1493, 1474, 1401, 1334, 1314, 1289, 1235, 1153, 1117, 1084, 1055, 839, 817, 777, 713, 692, 666, 616, 607.

HRMS (APCI+) calcd. for $C_{16}H_{14}FNO_2S$ ($[M+H]^+$): 304.0802, found: 304.0802.

4-((4-Fluorophenyl)sulfonyl)-1-methyl-2-phenyl-1H-indole (2n)

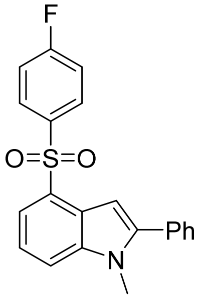

Light yellow oil. The pure form of **2n** was obtained more than 5 mg after GPC.

R$_f$ = 0.41 (Hexane : EtOAc = 4:1).

^{1}H-NMR (400 MHz, $CDCl_3$) δ 8.04-8.01 (m, 2H), 7.89 (d, *J* = 7.5 Hz, 1H), 7.58 (d, *J* = 8.2 Hz, 1H), 7.51-7.46 (m, 5H), 7.34 (t, *J* = 8.0 Hz, 1H), 7.10 (t, *J* = 8.5 Hz, 2H), 7.02 (s, 1H), 3.77 (s, 3H).

^{13}C-NMR (101 MHz, $CDCl_3$) δ 166.4, 163.8, 144.5, 139.2, 138.3, 131.5, 130.6, 130.0, 129.9, 129.5, 128.7, 128.7, 124.6, 121.4, 120.9, 116.3, 116.1, 115.2, 101.0, 31.5.

^{19}F-NMR (565 MHz, $CDCl_3$) δ -106.2 (m).

IR(neat): 3100, 3071, 2944, 2858, 2255, 1905, 1590, 1541, 1491, 1451, 1425, 1387, 1362, 1347, 1316, 1289, 1236, 1146, 1121, 1081, 1013, 929, 838, 768, 753, 735, 713, 703, 672, 625.

HRMS (APCI+) calcd. for $C_{21}H_{16}FNO_2S$ ($[M+H]^+$): 366.0959, found: 366.0959.

3-((4-Fluorophenyl)sulfonyl)-1-methyl-2-phenyl-1*H*-indole (3n)

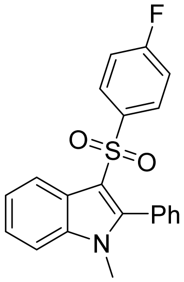

Colorless oil. The pure form of **3n** was obtained more than 5 mg after GPC.

R_f = 0.45 (Hexane : EtOAc = 4:1).

^{1}H-NMR (400 MHz, $CDCl_3$) δ 8.34-8.32 (m, 1H), 7.59-7.46 (m, 5H), 7.36 (dd, J = 6.2, 3.9 Hz, 3H), 7.29-7.27 (m, 2H), 6.96-6.91 (m, 2H), 3.49 (s, 3H).

^{13}C-NMR (101 MHz, $CDCl_3$) δ 165.9, 163.4, 144.3, 140.1, 136.0, 130.8, 129.8, 129.1, 129.1, 128.8, 128.1, 124.9, 123.6, 122.7, 120.6, 115.7, 115.5, 113.1, 110.1, 31.0.

^{19}F-NMR (565 MHz, $CDCl_3$) δ -107.2 (m).

IR(neat): 3101, 3070, 2946, 2888, 2853, 2254, 1903, 1590, 1529, 1492, 1467, 1443, 1381, 1325, 1288, 1231, 1145, 1116, 1080, 1016, 978, 910, 835, 818, 801, 746, 701, 687, 662, 634.

HRMS (APCI+) calcd. for $C_{21}H_{16}FNO_2S$ ($[M+H]^+$): 366.09585 found: 366.09589.

6-((4-Fluorophenyl)sulfonyl)-1-methyl-2-phenyl-1*H*-indole (4n)

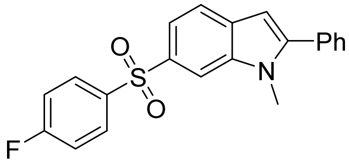

Light yellow oil. The pure form of **4n** was obtained more than 3 mg after GPC.

R_f = 0.51 (Hexane : EtOAc = 4:1).

^{1}H-NMR (400 MHz, $CDCl_3$) δ 8.07 (s, 1H), 8.00 (qd, J = 4.7, 2.4 Hz, 2H), 7.69 (d, J = 8.2 Hz, 1H), 7.62 (dd, J = 8.2, 1.4 Hz, 1H), 7.52-7.44 (m, 5H), 7.14 (t, J = 8.5 Hz, 2H), 6.60 (s, 1H), 3.82 (s, 3H).

^{13}C-NMR (101 MHz, $CDCl_3$) δ 166.3, 163.8, 145.9, 138.9, 137.1, 133.3, 131.5, 131.5, 130.1, 130.0, 129.3, 128.8, 128.7, 121.2, 118.7, 116.4, 116.2, 110.2, 102.2, 31.5.

^{19}F-NMR (565 MHz, $CDCl_3$) δ -106.3 (m).

IR(neat): 3101, 3070, 2937, 2883, 2853, 2255, 1901, 1703, 1589, 1537, 1491, 1469, 1427, 1388, 1358, 1312, 1288, 1233, 1147, 1116, 1084, 1056, 1012, 909, 836, 816, 773, 760, 732, 712, 691, 671, 644, 607.

HRMS (APCI+) calcd. for $C_{21}H_{16}FNO_2S$ ($[M+H]^+$): 366.0959, found: 366.0959.

1-benzyl-4-((4-fluorophenyl)sulfonyl)-2-propyl-1*H*-indole (2q)

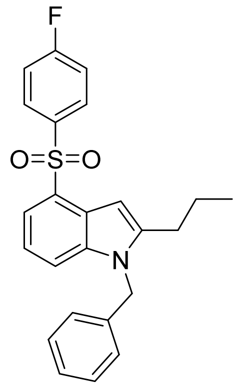

Light yellow solid. The pure form of **2q** was obtained more than 5 mg after recrystallization followed by GPC.

R_f = 0.65 (Hexane : EtOAc = 4:1)

^{1}H-NMR (400 MHz, $CDCl_3$) δ 8.03 (qd, J = 4.8, 2.5 Hz, 2H), 7.80 (d, J = 7.8 Hz, 1H), 7.36 (d, J = 8.2 Hz, 1H), 7.27-7.22 (m, 3H), 7.17-7.08 (m, 3H), 6.90-6.88 (m, 2H), 6.82 (s, 1H), 5.31 (s, 2H), 2.68-2.63 (m, 2H), 1.74 (td, J = 15.1, 7.3 Hz, 2H), 0.99 (t, J = 7.3 Hz, 3H).

^{13}C-NMR (101 MHz, $CDCl_3$) δ 166.3, 163.8, 144.9, 138.4, 138.1, 136.7, 130.0, 129.9, 129.8, 128.8, 127.6, 125.7, 124.9, 121.0, 120.1, 116.2, 116.0, 114.8, 99.0, 46.6, 28.8, 21.5, 13.9.

^{19}F-NMR (376 MHz, $CDCl_3$) δ -106.3 - -106.4 (m).

IR(neat): 3102, 3065, 3031, 2962, 2933, 2873, 1590, 1537, 1493, 1440, 1409, 1354, 1316, 1288, 1235, 1161, 1132, 1083, 1012, 931, 836, 774, 734, 672.

HRMS (APCI+) calcd. for $C_{24}H_{22}FNO_2S$ ($[M+H]^+$):408.1428, found:408.1428.

1-benzyl-3-((4-fluorophenyl)sulfonyl)-2-propyl-1*H*-indole (3q)

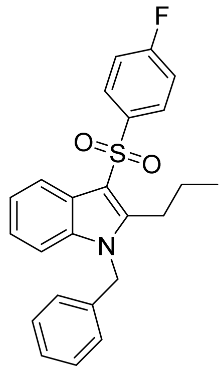

Light yellow oil. The pure form of **3q** was obtained more than 3 mg after GPC.

R_f = 0.67 (Hexane : EtOAc = 4:1)

^{1}H-NMR (400 MHz, $CDCl_3$) δ 8.11 (d, J = 7.8 Hz, 1H), 8.02-7.99 (m, 2H), 7.29-7.24 (m, 4H), 7.21-7.09 (m, 4H), 6.93-6.91 (m, 2H), 5.35 (s, 2H), 3.13-3.09 (m, 2H), 1.59-1.50 (m, 2H), 1.00 (t, J = 7.3 Hz, 3H).

^{13}C-NMR (101 MHz, $CDCl_3$) δ 166.1, 163.6, 146.5, 140.6, 136.1, 135.7, 129.0, 128.9, 128.8, 127.9, 125.7, 125.1, 123.3, 122.6, 119.9, 116.2, 116.0, 110.9, 110.4, 47.0, 27.0, 23.5, 14.3.

^{19}F-NMR (376 MHz, $CDCl_3$) δ -106.9 - -107.0 (m).

IR(neat): 3101, 3065, 3033, 2964, 2932, 2873, 1591, 1513, 1493, 1463, 1455, 1438, 1407, 1324, 1307, 1287, 1235, 1178, 1156, 1137, 1093, 1080, 1024, 1005, 967, 909, 836, 819, 785, 730, 691.

HRMS (APCI+) calcd. for $C_{24}H_{22}FNO_2S$ ($[M+H]^+$):408.1428, found:408.1428.

1-benzyl-6-((4-fluorophenyl)sulfonyl)-2-propyl-1*H*-indole (4q)

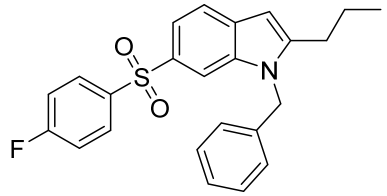

Light yellow solid. The pure form of **4q** was obtained more than 5 mg after recrystallization followed by GPC.

R_f = 0.68 (Hexane : EtOAc = 4:1)

^{1}H-NMR (400 MHz, $CDCl_3$) δ 7.86-7.82 (m, 3H), 7.61 (d, J = 8.2 Hz, 1H), 7.54 (dd, J = 8.5, 1.6 Hz, 1H), 7.26 (td, J = 5.4, 3.7 Hz, 3H), 7.09-7.04 (m, 2H), 6.91-6.88 (m, 2H), 6.40 (s, 1H), 5.38 (s, 2H), 2.66 (t, J = 7.6 Hz, 2H), 1.71 (td, J = 14.9, 7.5 Hz, 2H), 0.98 (t, J = 7.3 Hz, 3H).

^{13}C-NMR (101 MHz, $CDCl_3$) δ 166.1, 163.6, 146.3, 139.0, 136.7, 136.0, 132.5, 131.8, 129.9, 129.8, 128.8, 127.6, 125.8, 120.5, 118.4, 116.3, 116.0, 109.8, 100.3, 46.7, 28.8, 21.4, 13.9.

^{19}F-NMR (376 MHz, $CDCl_3$) δ -106.6 - -106.7 (m).

IR(neat): 3102, 3065, 3032, 2962, 2933, 2872, 1590, 1533, 1508, 1493, 1464, 1455, 1409, 1358, 1338, 1313, 1289, 1235, 1201, 1151, 1088, 1056, 1012, 837, 817, 734, 715, 700, 660, 607.

HRMS (APCI+) calcd. for $C_{24}H_{22}FNO_2S$ ($[M+H]^+$):408.1428, found:408.1428.

1-methyl-2-phenyl-4-tosyl-1H-indole (2r)

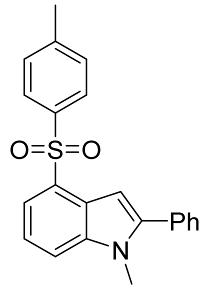

Light yellow solid. The pure form of **2r** was obtained more than 5 mg after recrystallization followed by GPC.

R_f = 0.53 (Hexane : EtOAc = 4:1).

^{1}H-NMR (400 MHz, $CDCl_3$) δ 7.89 (dt, J = 7.9, 1.3 Hz, 3H), 7.54-7.41 (m, 6H), 7.30 (t, J = 7.8 Hz, 1H), 7.21 (d, J = 8.2 Hz, 2H), 7.04 (d, J = 0.9 Hz, 1H), 3.73 (s, 3H), 2.31 (s, 3H).

^{13}C-NMR (101 MHz, $CDCl_3$) δ 144.2, 143.5, 139.3, 139.1, 131.6, 131.1, 129.5, 129.4, 128.6, 128.6, 127.3, 124.5, 121.2, 120.8, 114.9, 101.1, 31.4, 21.4.

IR(neat): 3061, 3026, 2948, 2922, 2866, 1908, 1597, 1563, 1541, 1486, 1450, 1425, 1386, 1361, 1346, 1312, 1300, 1289, 1210, 1183, 1145, 1120, 1081, 1037, 1017, 929, 855, 813, 765, 752, 708, 666, 625.

HRMS (APCI+) calcd. for $C_{22}H_{19}NO_2S$ ($[M+H]^+$): 362.1209, found: 362.1209.

1-methyl-2-phenyl-3-tosyl-1*H*-indole (3r)

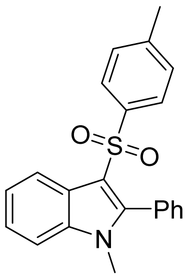

Colorless crystal. The pure form of **3r** was obtained more than 5 mg after recrystallization followed by GPC.

R_f = 0.57 (Hexane : EtOAc = 4:1).

^{1}H-NMR (400 MHz, $CDCl_3$) δ 8.34-8.31 (m, 1H), 7.53-7.44 (m, 5H), 7.35-7.22 (m, 5H), 7.06 (d, J = 8.2 Hz, 2H), 3.44 (s, 3H), 2.28 (s, 3H).

^{13}C-NMR (101 MHz, $CDCl_3$) δ 144.0, 142.7, 141.2, 136.0, 130.7, 129.6, 129.0, 129.0, 127.9, 126.3, 124.8, 123.4, 122.4, 120.5, 113.4, 110.0, 30.9, 21.3.

IR(neat): 3057, 3025, 2947, 2923, 1910, 1597, 1529, 1466, 1443, 1379, 1338, 1323, 1300, 1246, 1221, 1182, 1145, 1115, 1082, 1018, 977, 923, 843, 802, 745, 701, 687, 659, 633.

HRMS (APCI+) calcd. for $C_{22}H_{19}NO_2S$ ($[M+H]^+$): 362.1209, found: 362.1209.

1-methyl-2-phenyl-6-tosyl-1*H*-indole (4r)

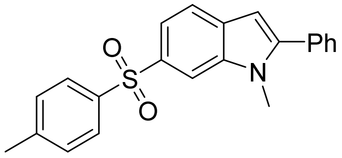

Light yellow solid. The pure form of **4r** was obtained more than 5 mg after recrystallization followed by GPC.

R_f = 0.61 (Hexane : EtOAc = 4:1).

^{1}H-NMR (400 MHz, $CDCl_3$) δ 8.07 (s, 1H), 7.86 (d, J = 8.2 Hz, 2H), 7.64 (dd, J = 20.4, 8.5 Hz, 2H), 7.49-7.43 (m, 5H), 7.26 (d, J = 7.8 Hz, 2H), 6.58 (s, 1H), 3.81 (s, 3H), 2.37 (s, 3H).

^{13}C-NMR (101 MHz, $CDCl_3$) δ 145.6, 143.4, 139.9, 137.1, 133.9, 131.6, 131.3, 129.7, 129.3, 128.7, 127.3, 121.0, 118.7, 110.1, 102.1, 31.5, 21.5.

IR(neat): 3060, 3028, 2948, 2924, 2881, 1598, 1536, 1469, 1388, 1358, 1331, 1312, 1300, 1211, 1149, 1116, 1085, 1057, 1016, 926, 817, 760, 706, 691, 665, 644, 608. HRMS (APCI+) calcd. for $C_{22}H_{19}NO_2S$ ($[M+H]^+$): 362.1209, found: 362.1209.

1-methyl-2-propyl-7-tosyl-*1H*-indole (5a)

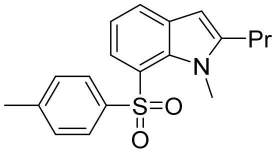

White solid. The pure form of **5a** was obtained more than 1 mg after recrystallization followed by GPC.

R_f = 0.62 (Hexane : EtOAc = 4:1). IR(neat): 3060, 2961, 2931, 2873, 1724, 1597, 1538, 1453, 1415,

1299, 1205, 1153, 1136, 1082, 1066, 1018, 942, 913, 814, 734, 707, 683. HRMS (APCI+) calcd. for $C_{19}H_{21}NO_2S$ ([M+H]$^+$): 328.13658 found: 328.13662.

^{1}H-NMR (400 MHz, $CDCl_3$) δ 7.77-7.73 (m, 3H), 7.57 (dd, *J* = 7.8, 0.9 Hz, 1H), 7.32 (d, *J* = 8.2 Hz, 2H), 7.05 (t, *J* = 7.8 Hz, 1H), 6.38 (s, 1H), 3.87 (s, 3H), 2.65 (t, *J* = 7.6 Hz, 2H), 2.44 (s, 3H), 1.73 (td, *J* = 15.1, 7.3 Hz, 2H), 1.03 (t, *J* = 7.3 Hz, 3H).

^{13}C-NMR (101 MHz, $CDCl_3$) δ 144.0, 143.8, 139.3, 132.2, 131.4, 129.8, 127.4, 125.9, 123.8, 123.7, 117.7, 100.6, 34.0, 29.5, 21.6, 21.4, 14.0.

7-((4-fluorophenyl)sulfonyl)-1-methyl-2-propyl-*1H*-indole (5f)

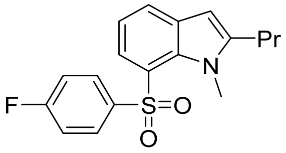

White solid. The pure form of **5f** was obtained more than 1 mg after recrystallization followed by GPC.

R

R_f = 0.70 (Hexane : EtOAc = 4:1). IR(neat): 3102, 3073, 2961, 2932, 2873, 1730, 1668, 1589, 1538, 1493, 1454, 1415, 1309, 1289, 1236, 1152, 1136, 1081, 1066, 943, 840, 801, 734, 711, 686. HRMS (APCI+) calcd. for $C_{18}H_{18}FNO_2S$ ([M+H]$^+$): 332.11150 found: 328.11153.

^{1}H-NMR (400 MHz, $CDCl_3$) δ 7.90 (tt, *J* = 7.3, 2.3 Hz, 2H), 7.76 (dd, *J* = 7.8, 0.9 Hz, 1H), 7.56 (dd, *J* = 7.8, 0.9 Hz, 1H), 7.24-7.18 (m, 2H), 7.07 (t, *J* = 7.8 Hz, 1H), 6.39 (s, 1H), 3.87 (s, 3H), 2.66 (t, *J* = 7.6 Hz, 2H), 1.74 (td, *J* = 14.9, 7.5 Hz, 2H), 1.04 (t, *J* = 7.3 Hz, 3H)

^{13}C-NMR (101 MHz, $CDCl_3$) δ 166.6, 164.0, 144.0, 138.4, 132.1, 131.6, 130.2, 130.1, 126.2, 123.8, 123.2, 117.8, 116.6, 116.4, 100.8, 34.0, 29.5, 21.4, 14.0.

^{19}F-NMR (565 MHz, $CDCl_3$) δ -105.2 (m).

10. NMR charts of $SPhosAuBF_4$

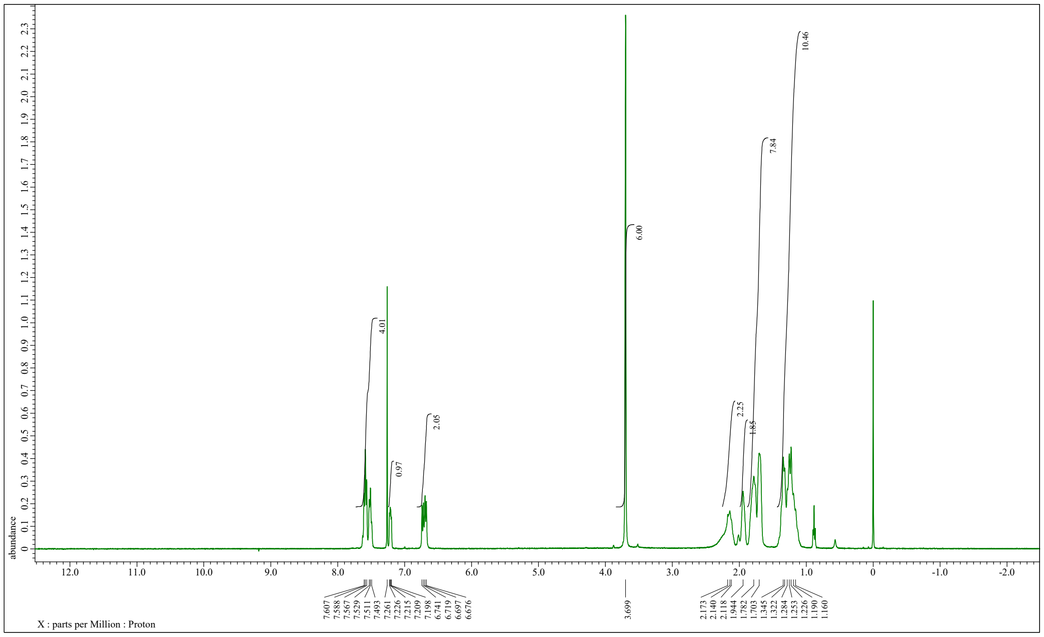

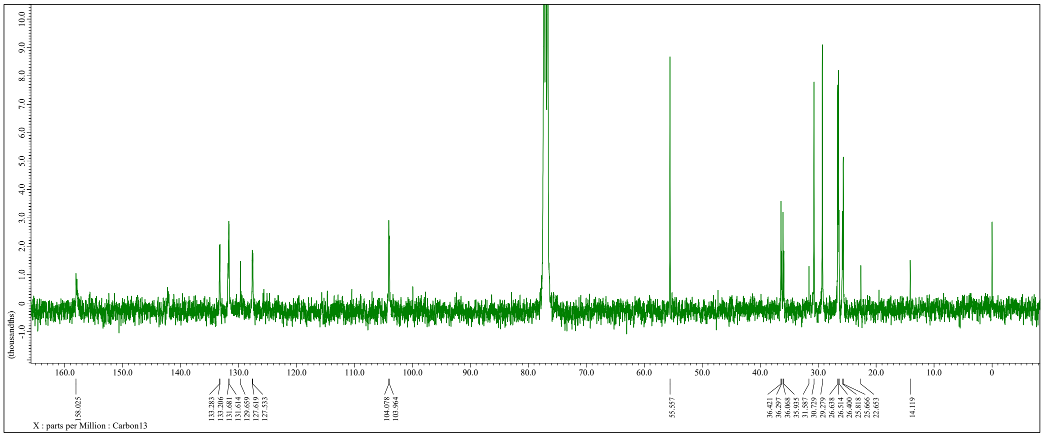

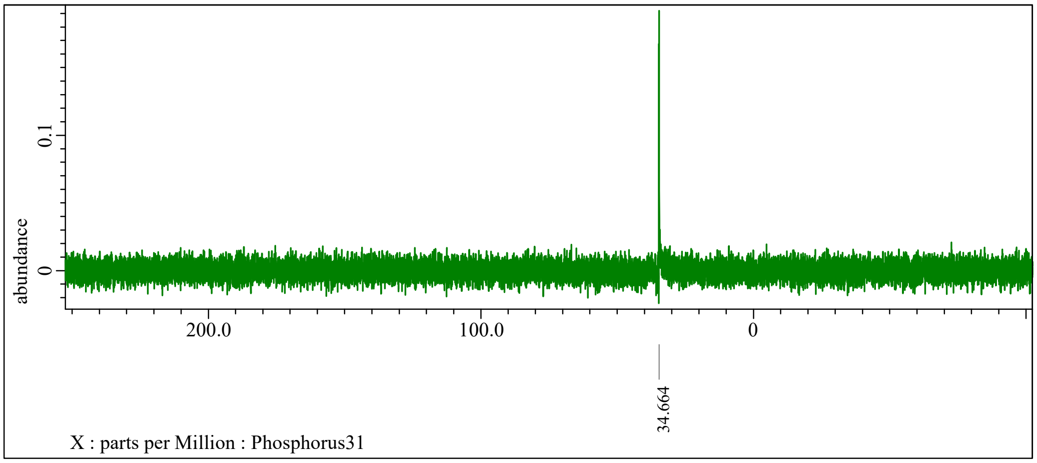

11. ^{1}H, ^{13}C and 1D NOESY NMR charts of substrates and products

N,4-Dimethyl-*N*-(2-(pent-1-yn-1-yl)phenyl)benzenesulfonamide (1a)

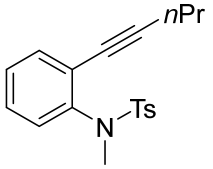

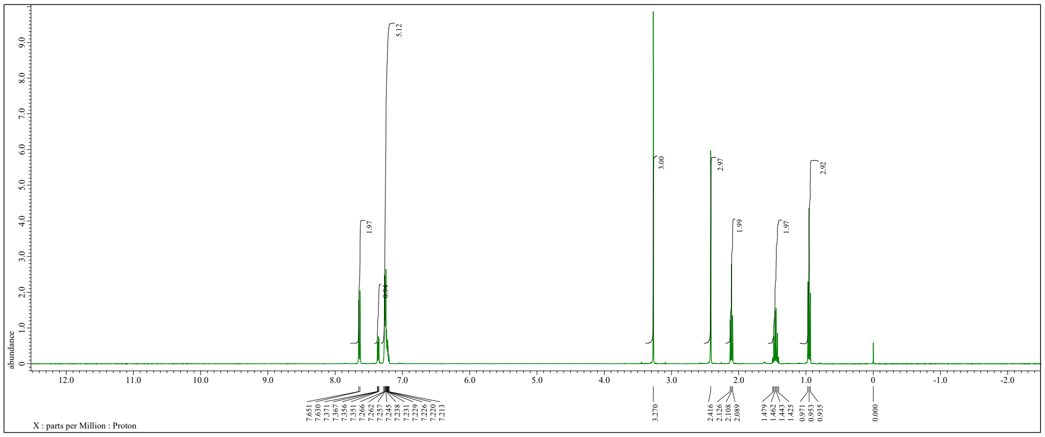

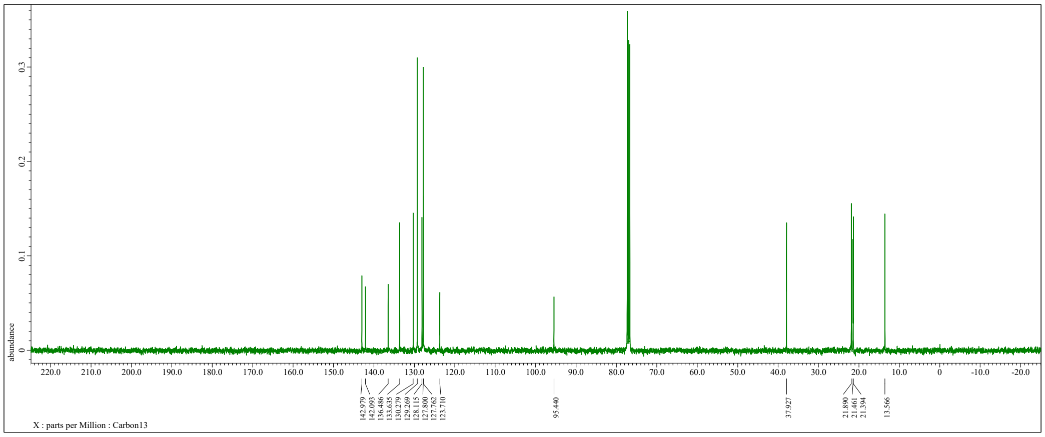

4-Methoxy-*N*-methyl-*N*-(2-(pent-1-yn-1-yl)phenyl)benzenesulfonamide 1b

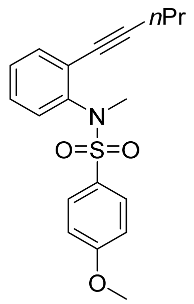

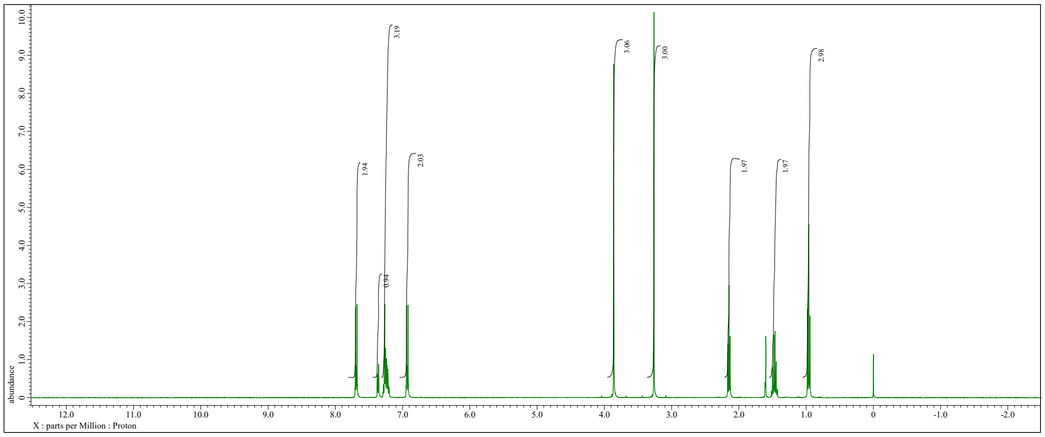

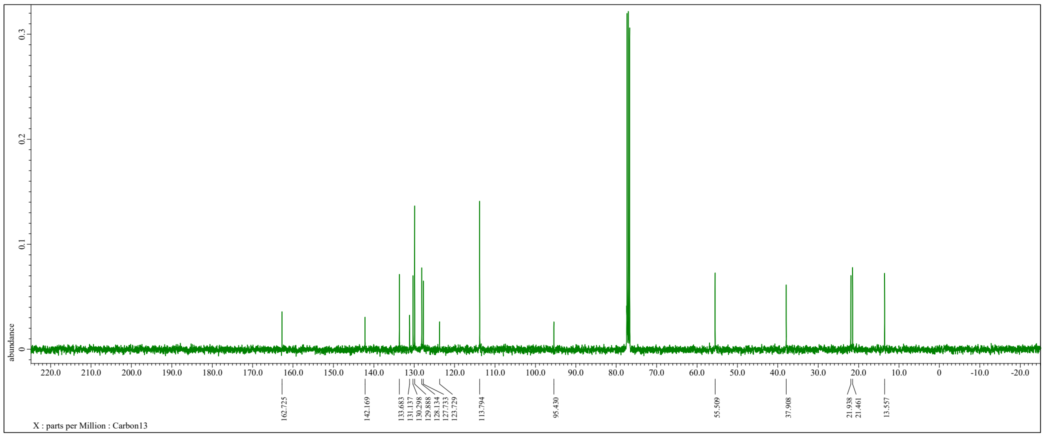

N,3-Dimethyl-N-(2-(pent-1-yn-1-yl)phenyl)benzenesulfonamide 1c

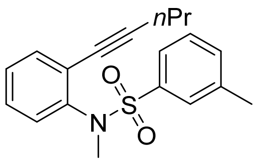

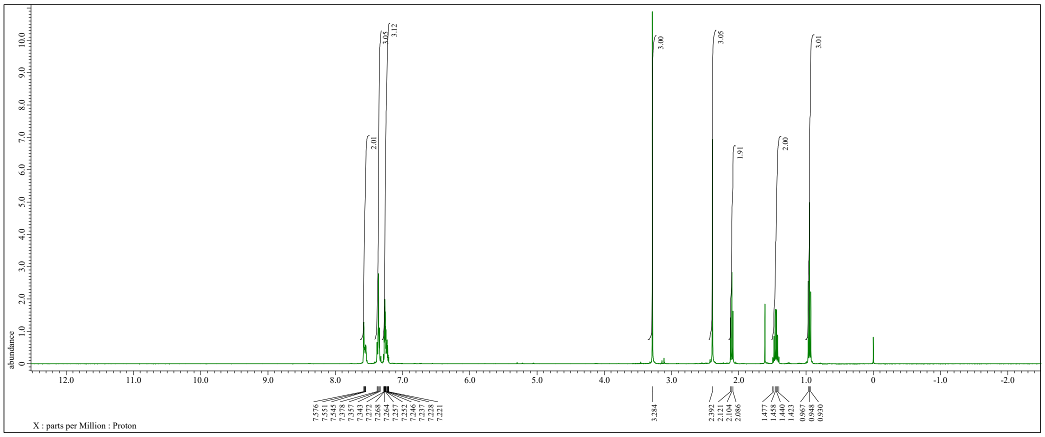

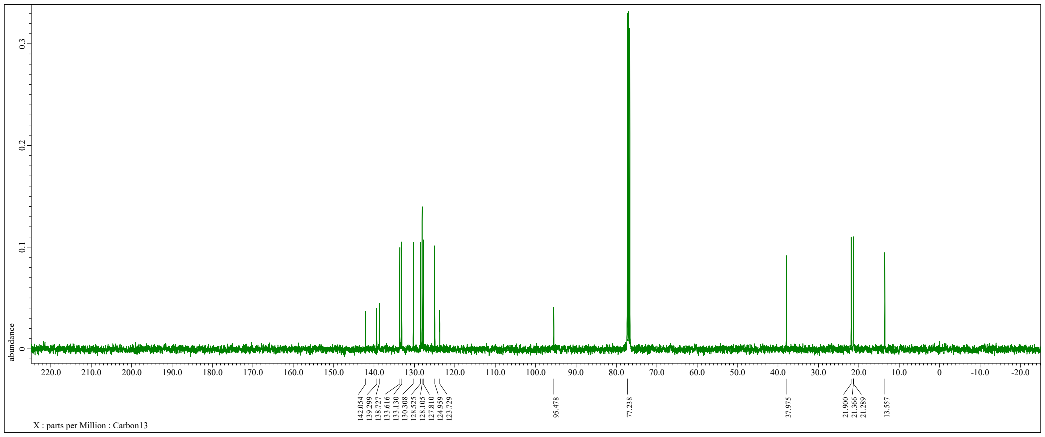

N,2-Dimethyl-N-(2-(pent-1-yn-1-yl)phenyl)benzenesulfonamide 1d

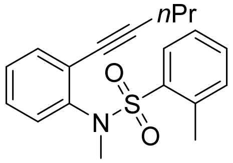

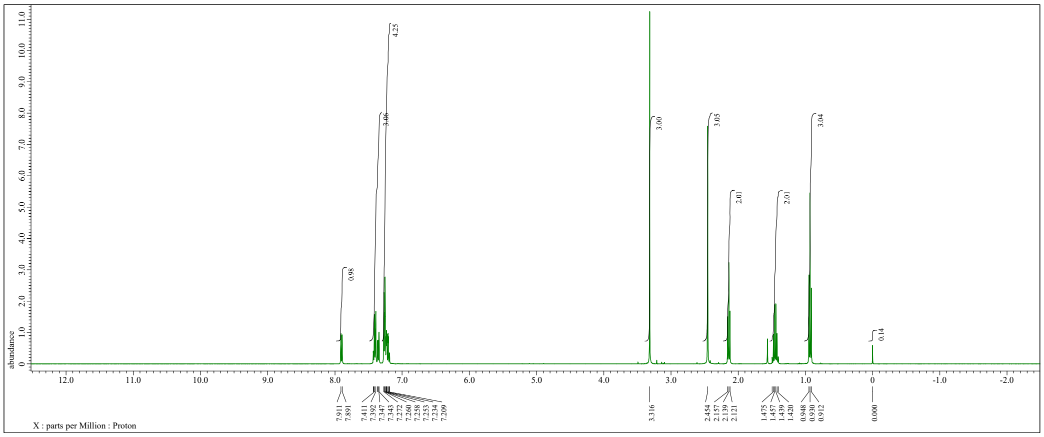

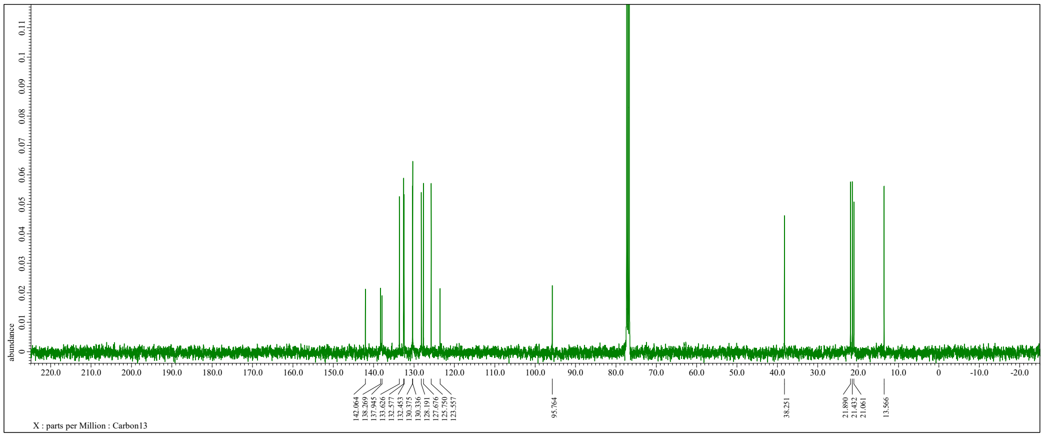

4-Fluoro-*N*-methyl-*N*-(2-(pent-1-yn-1-yl)phenyl)benzenesulfonamide 1e

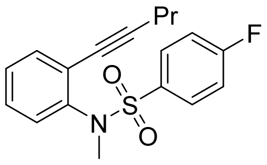

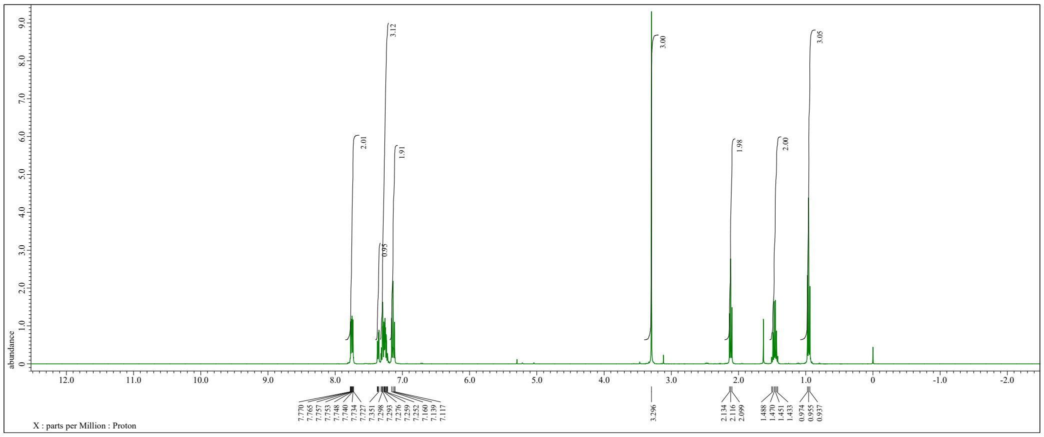

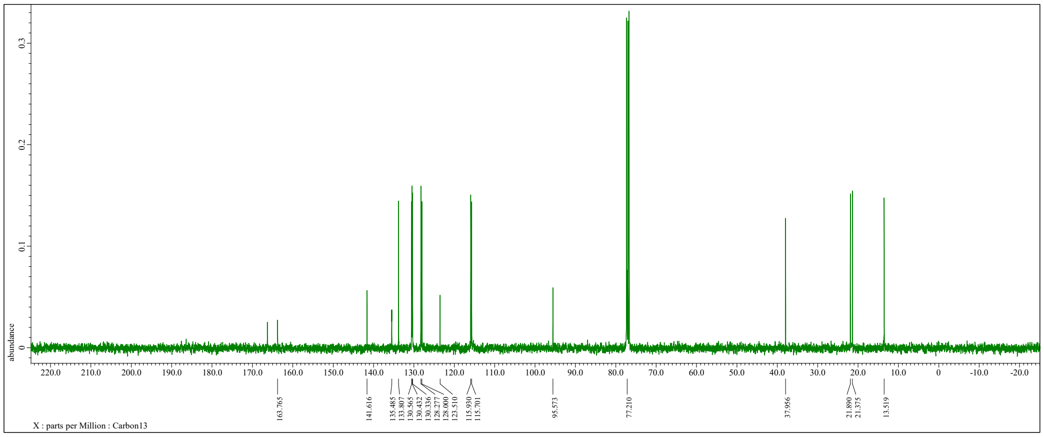

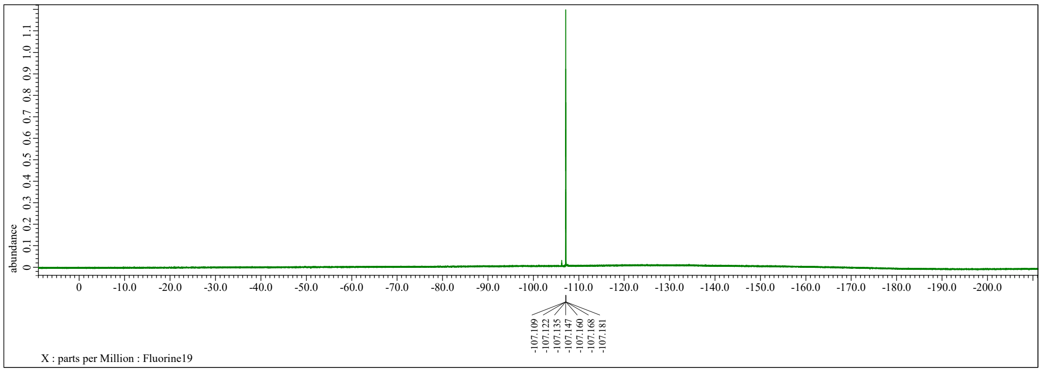

4-chloro-*N*-methyl-*N*-(2-(pent-1-yn-1-yl)phenyl)benzenesulfonamide 1f

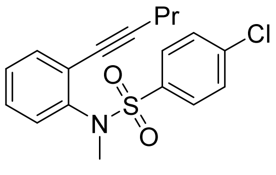

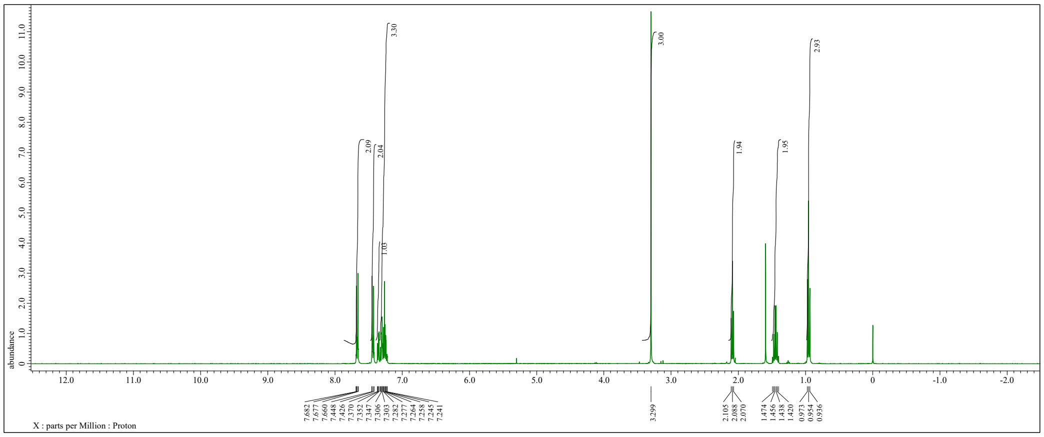

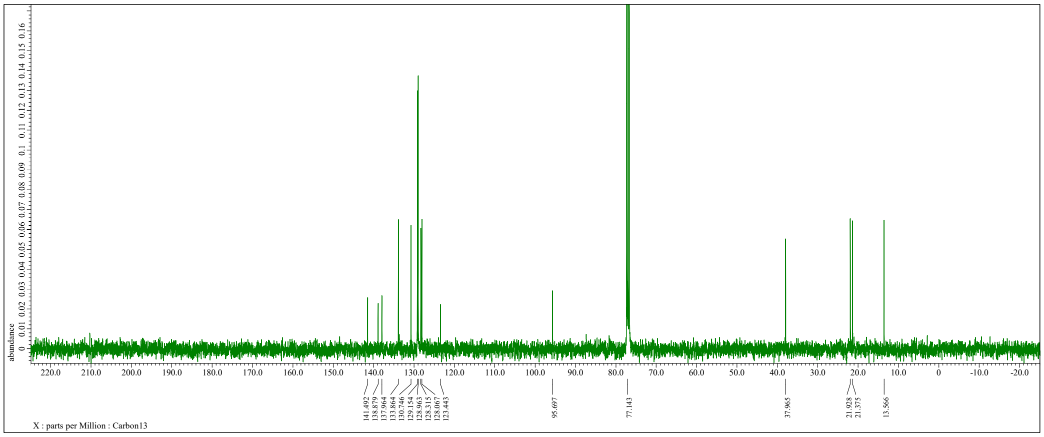

_N_-Methyl-_N_-(2-(pent-1-yn-1-yl)phenyl)-4-(trifluoromethyl)benzene-sulfonamide 1g

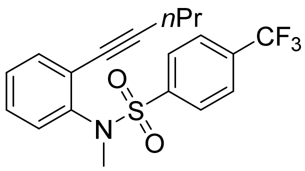

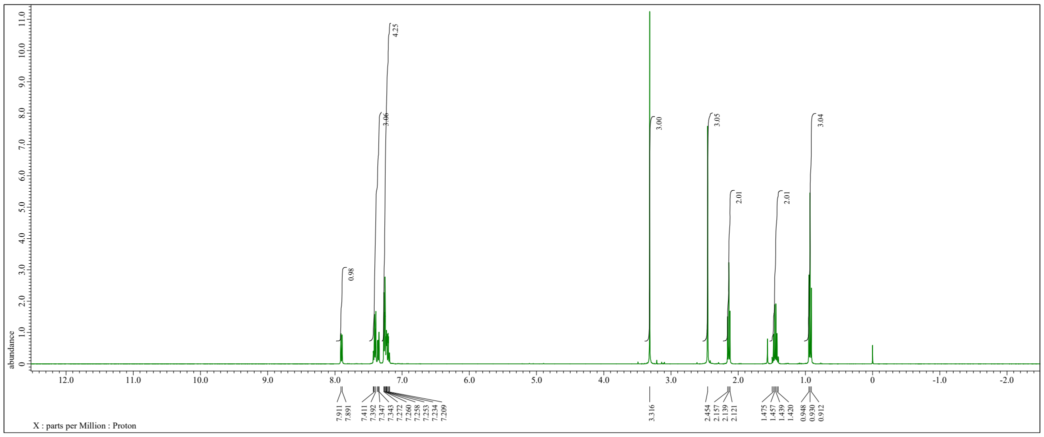

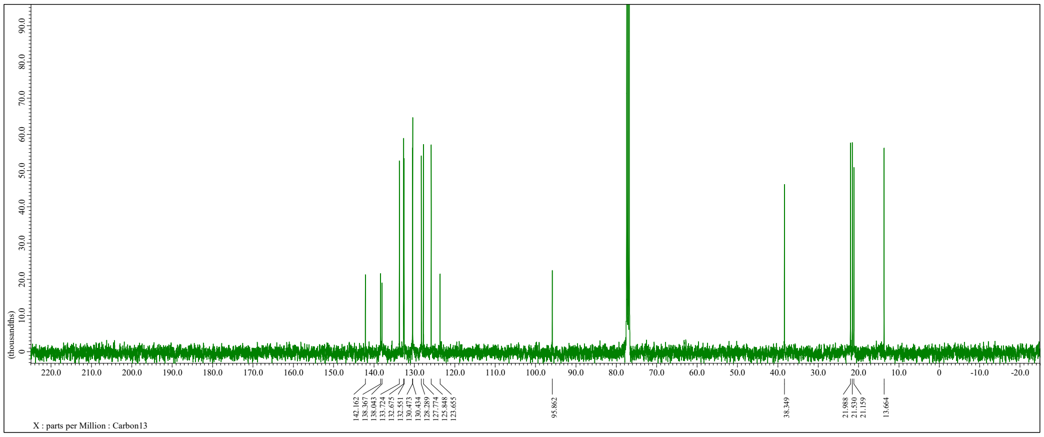

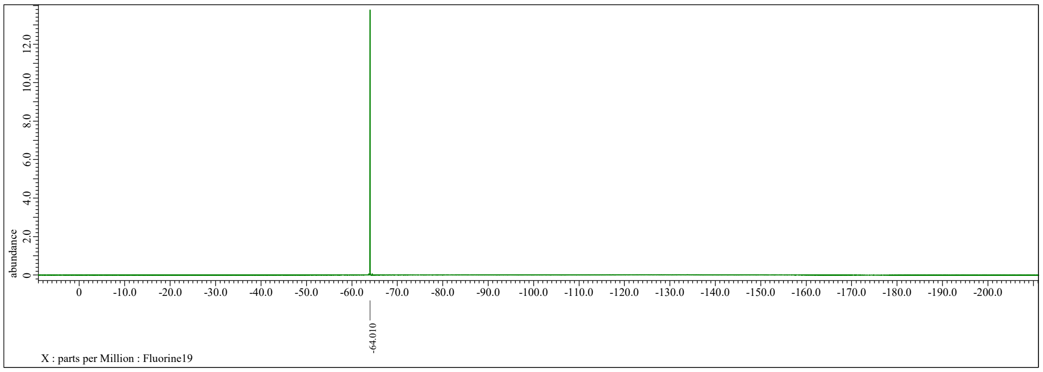

_N_-Methyl-4-nitro-_N_-(2-(pent-1-yn-1-yl)phenyl)benzenesulfonamide 1h

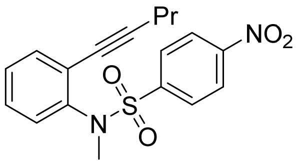

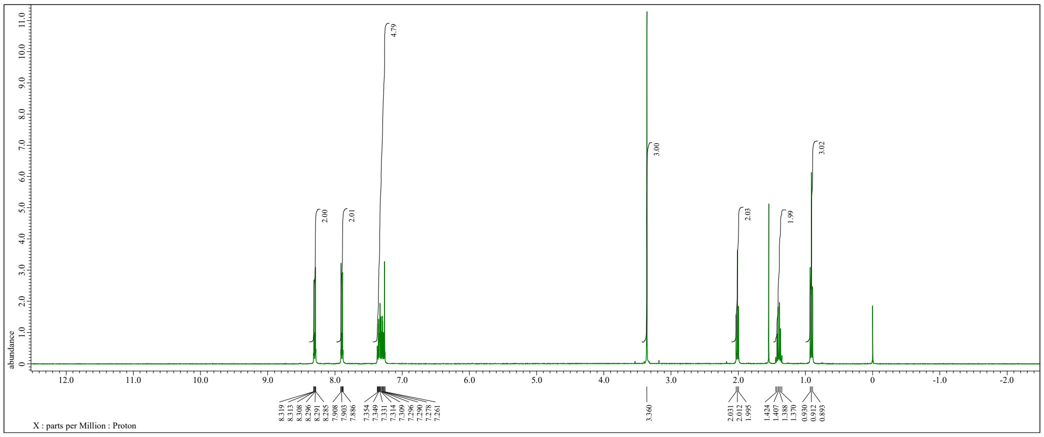

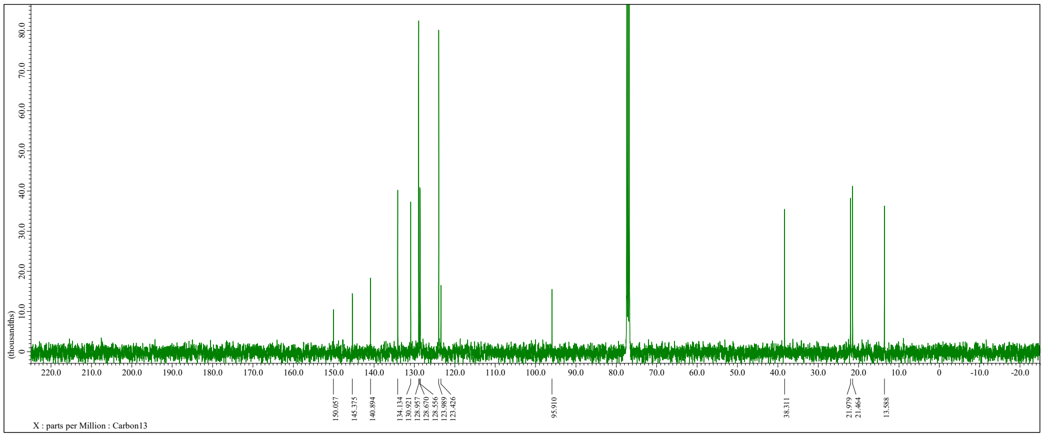

N-Methyl-N-(2-(pent-1-yn-1-yl)phenyl)methanesulfonamide 1i

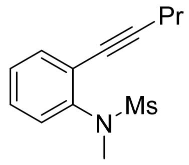

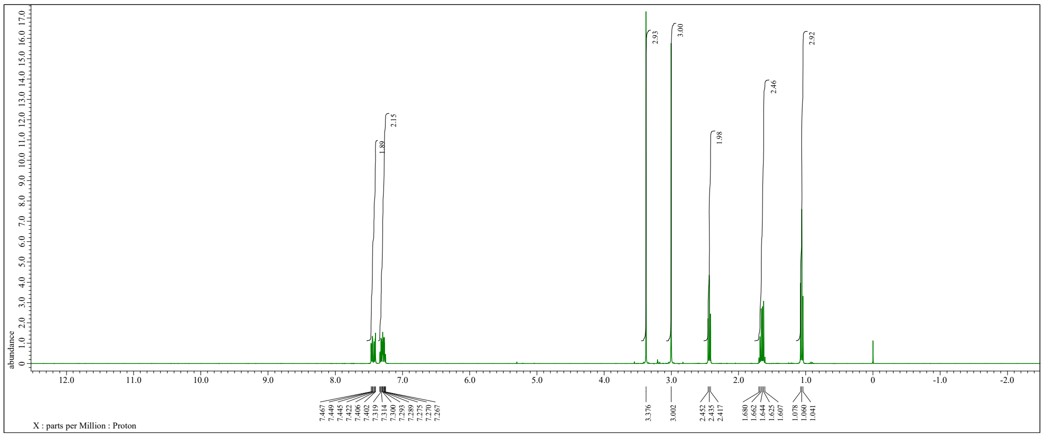

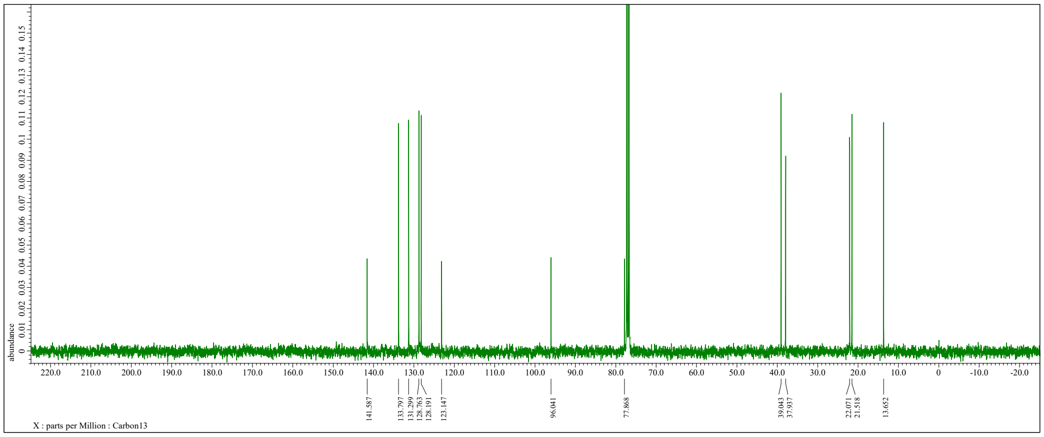

4-Fluoro-*N*-methyl-*N*-(2-(oct-1-yn-1-yl)phenyl)benzenesulfonamide 1j

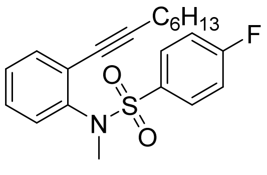

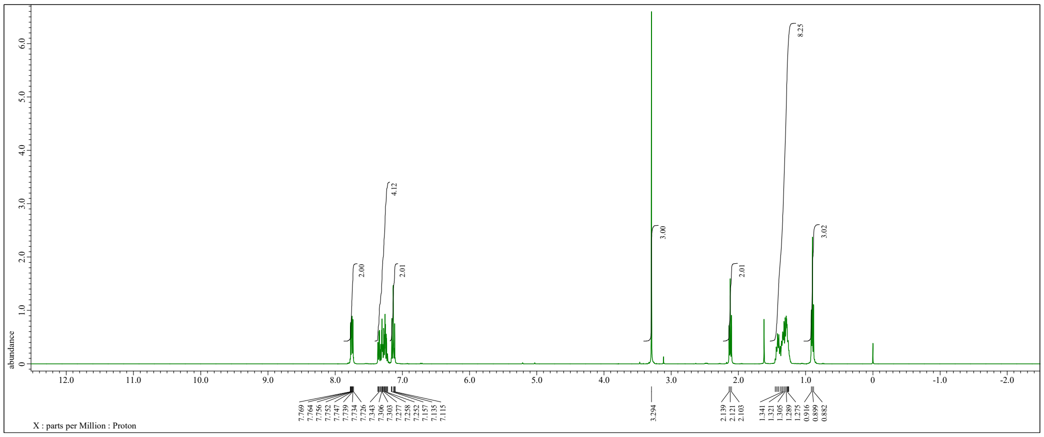

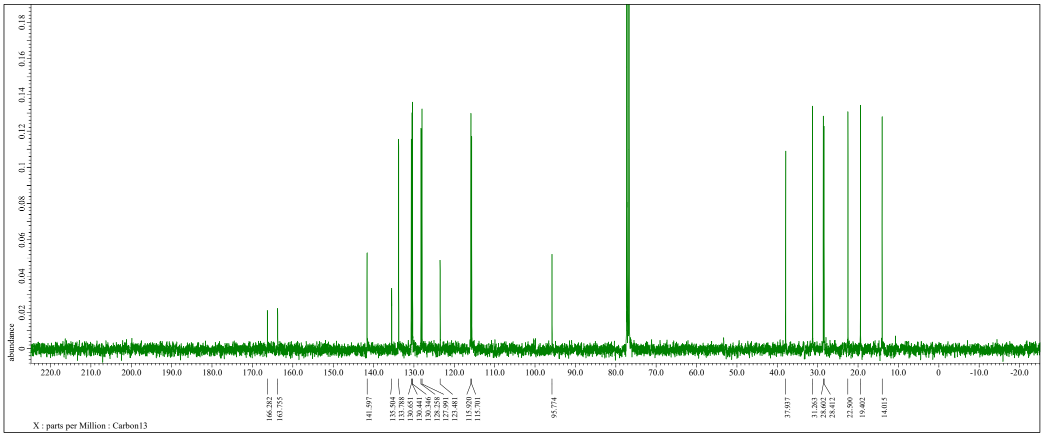

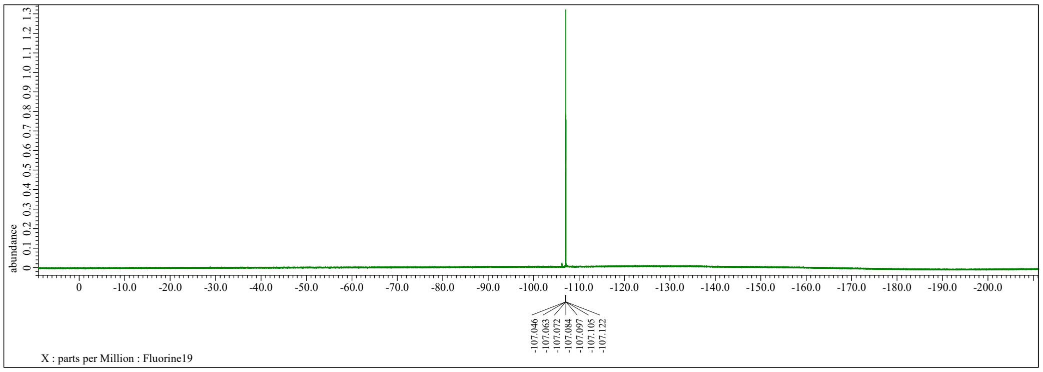

N-(2-(Cyclohexylethynyl)phenyl)-4-fluoro-N-methylbenzenesulfonamide 1k

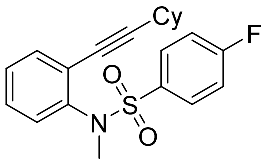

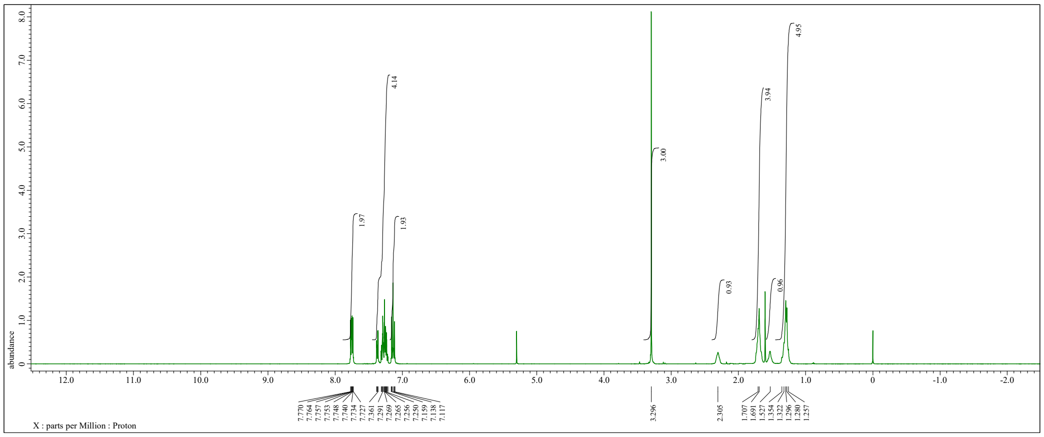

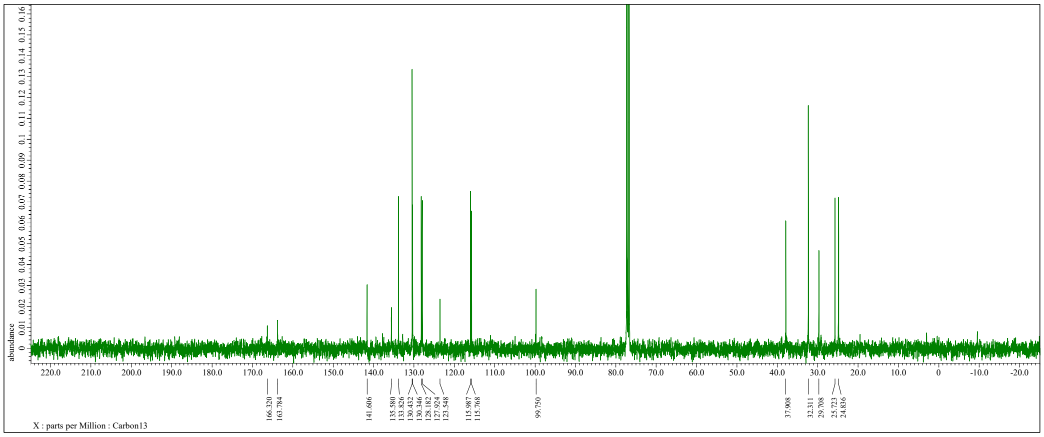

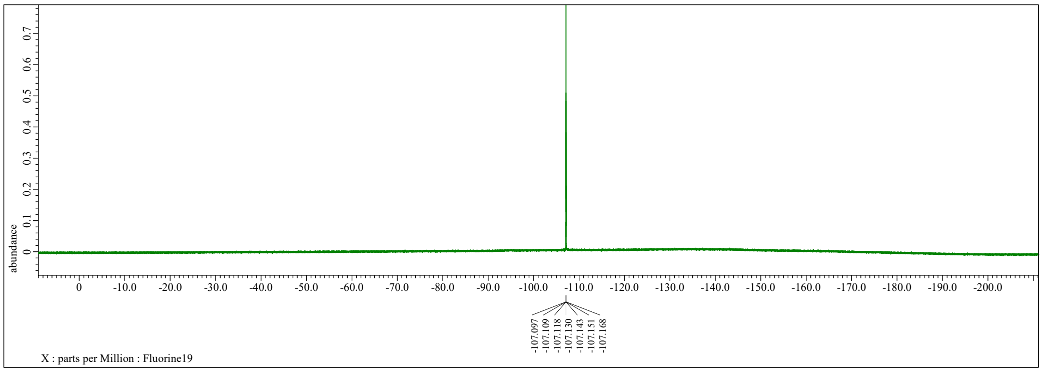

N-(2-(Cyclopropylethynyl)phenyl)-4-fluoro-N-methylbenzenesulfonamide 1l

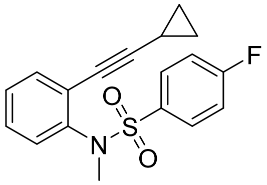

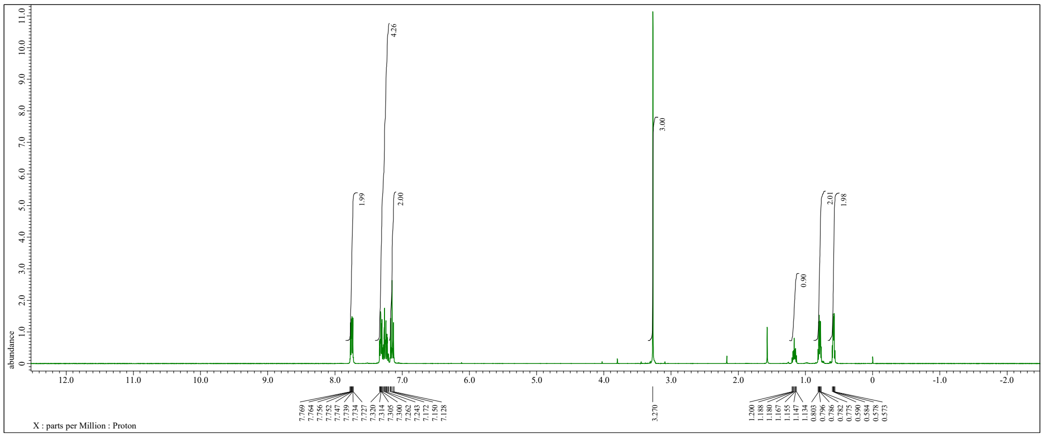

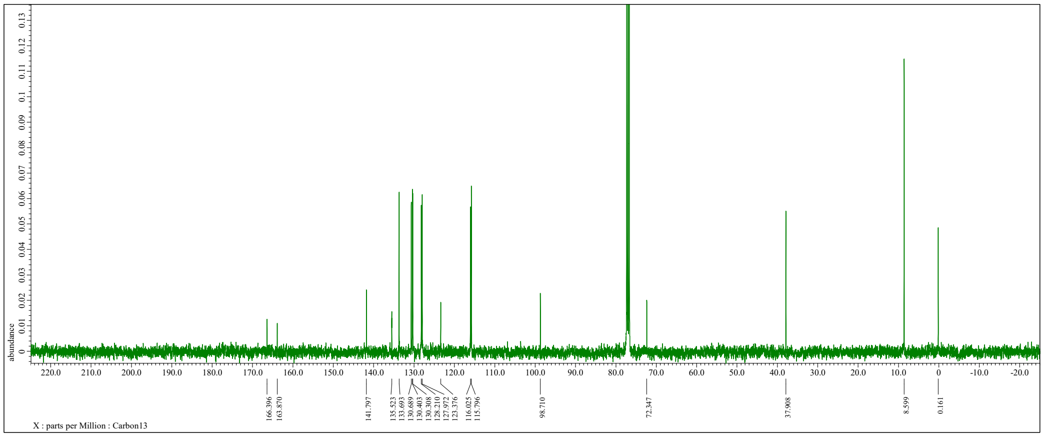

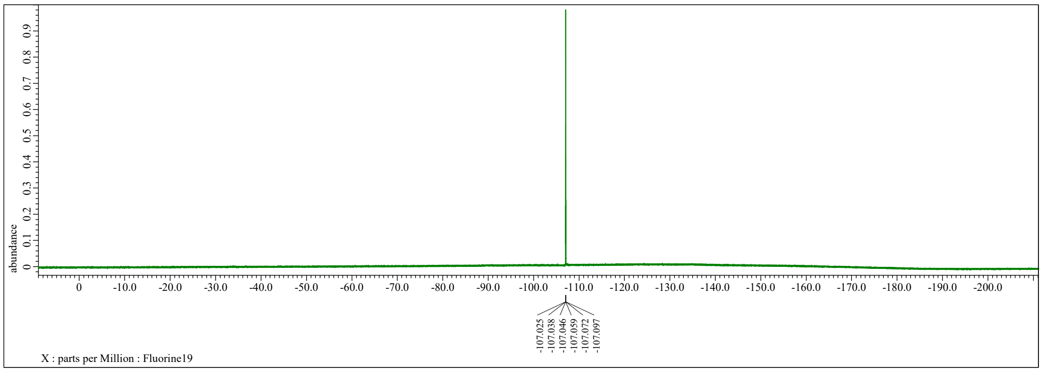

4-Fluoro-*N*-methyl-*N*-(2-(pent-1-yn-1-yl)phenyl)benzenesulfonamide 1m

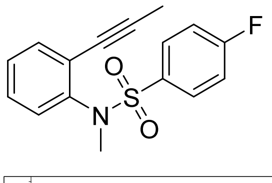

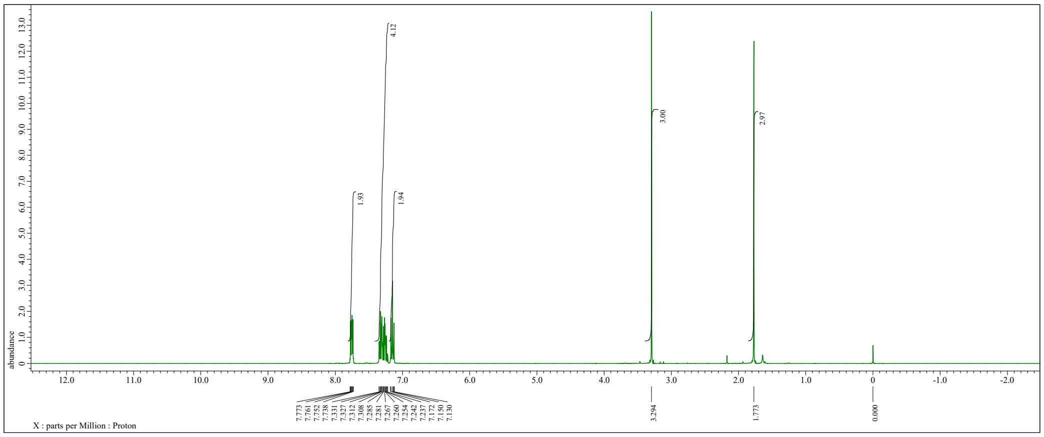

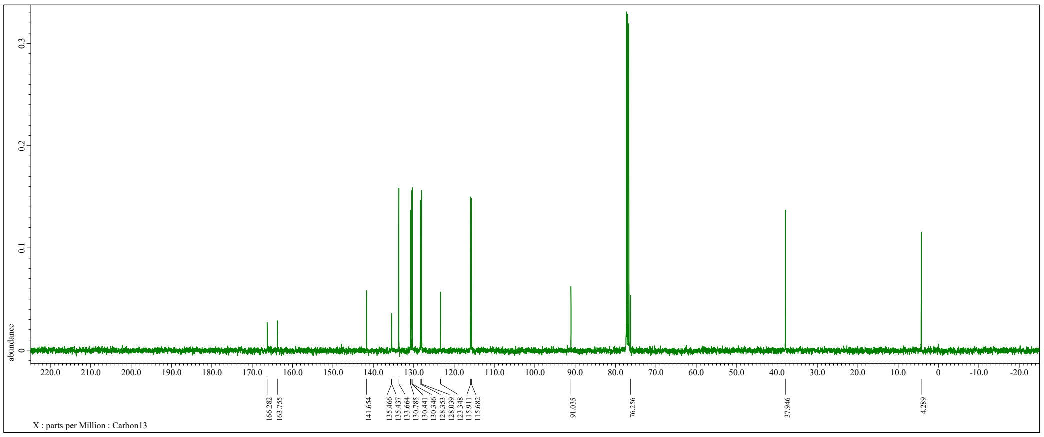

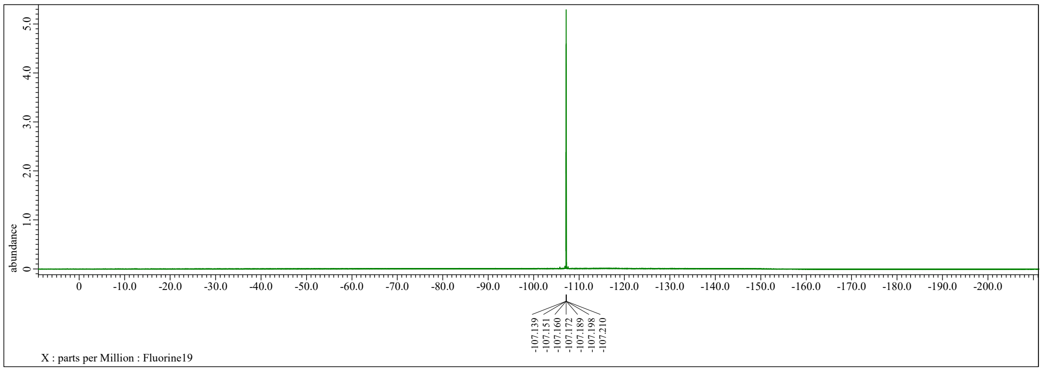

4-Fluoro-*N*-methyl-*N*-(2-(phenylethynyl)phenyl)benzenesulfonamide 1n

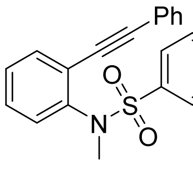

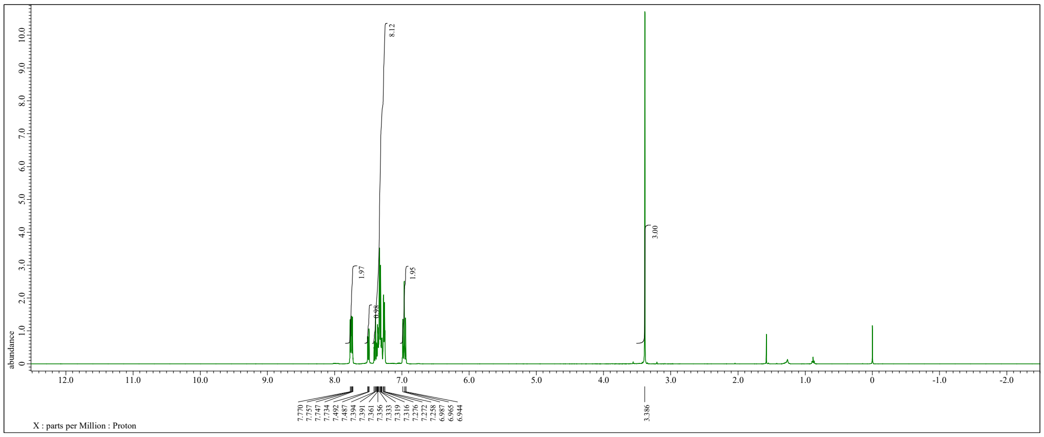

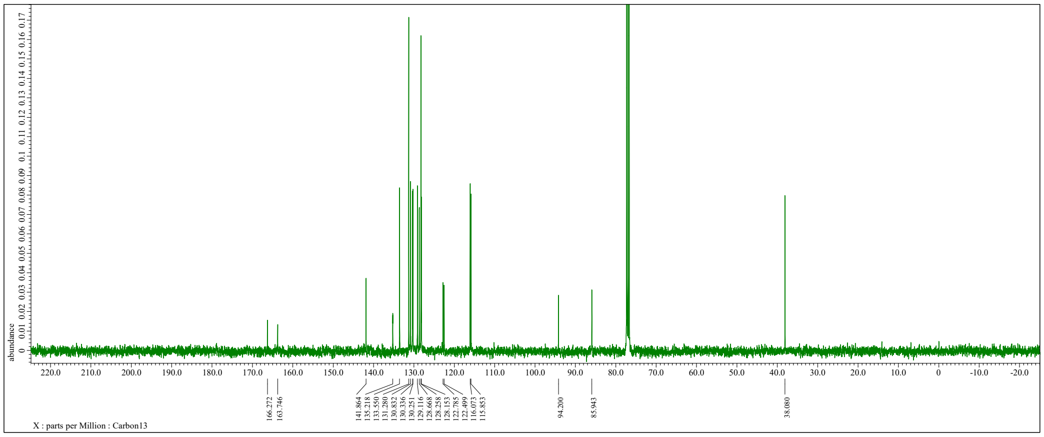

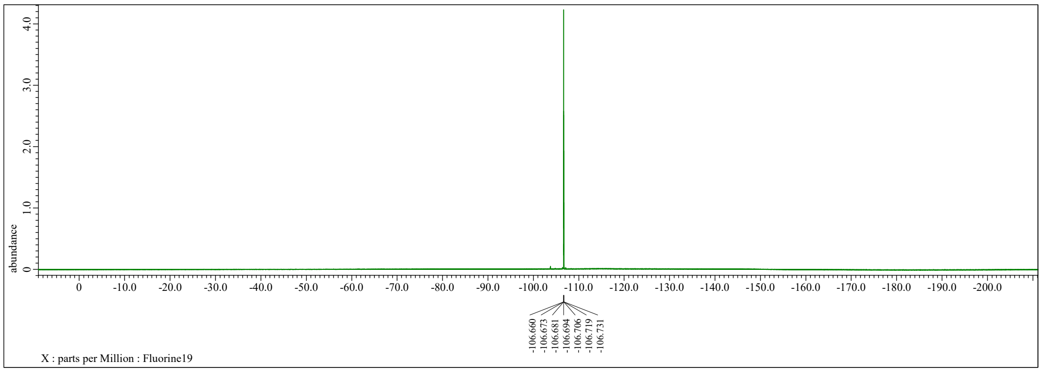

4-Fluoro-N-methyl-N-(2-((trimethylsilyl)ethynyl)phenyl) benzenesulfon-amide 1o

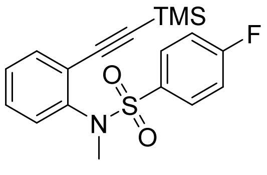

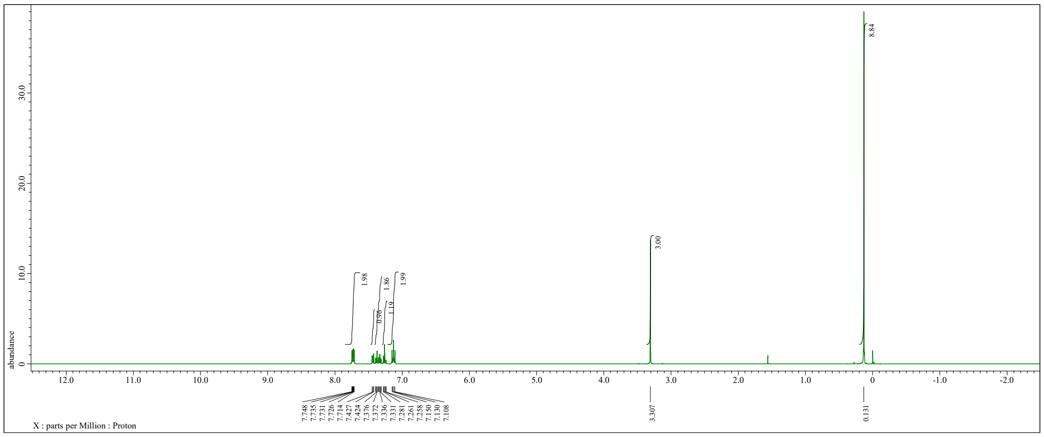

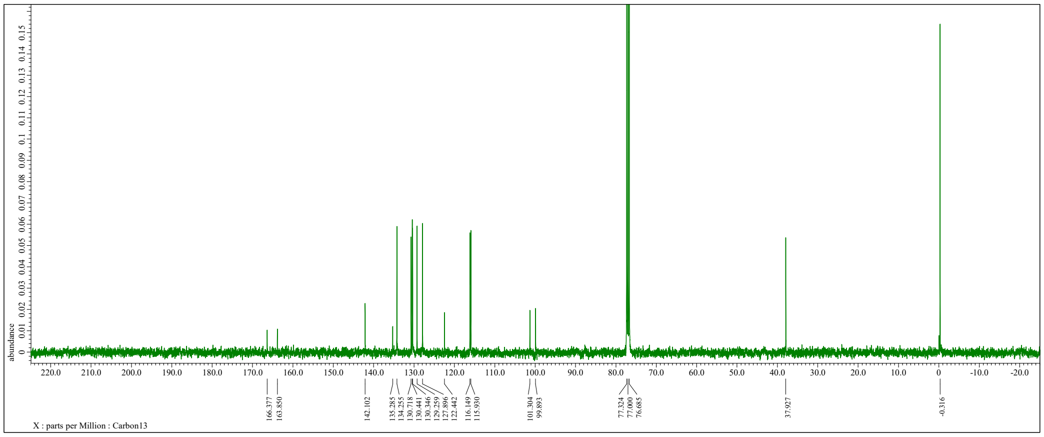

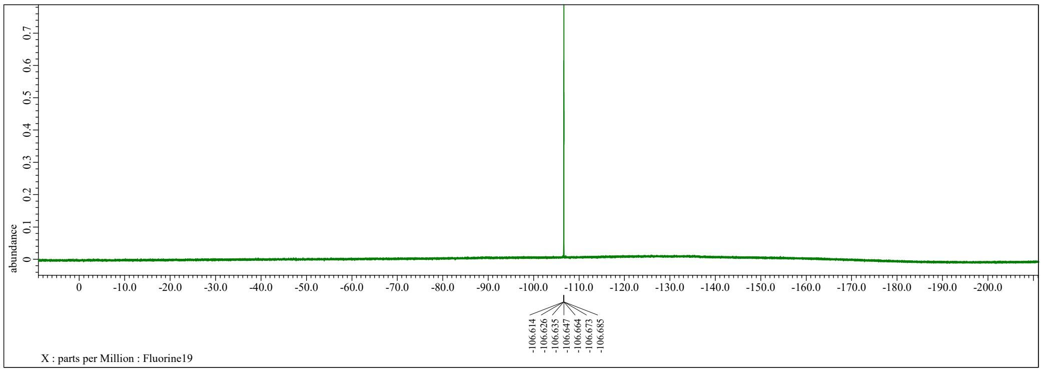

N-(2-Ethynylphenyl)-4-fluoro-*N*-methylbenzenesulfonamide 1p

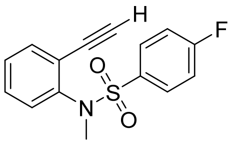

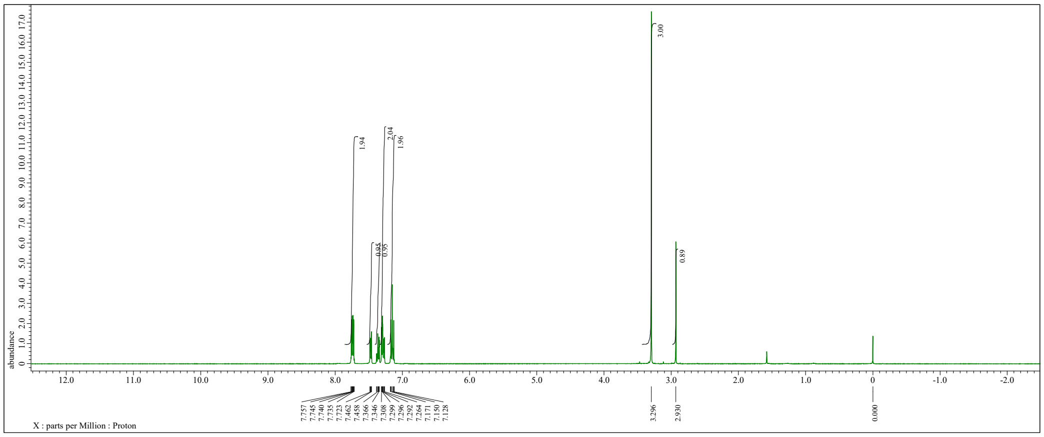

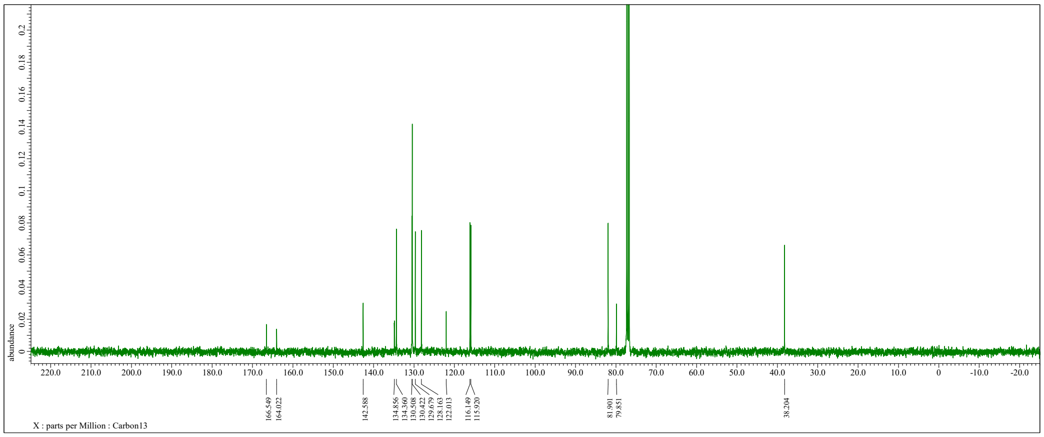

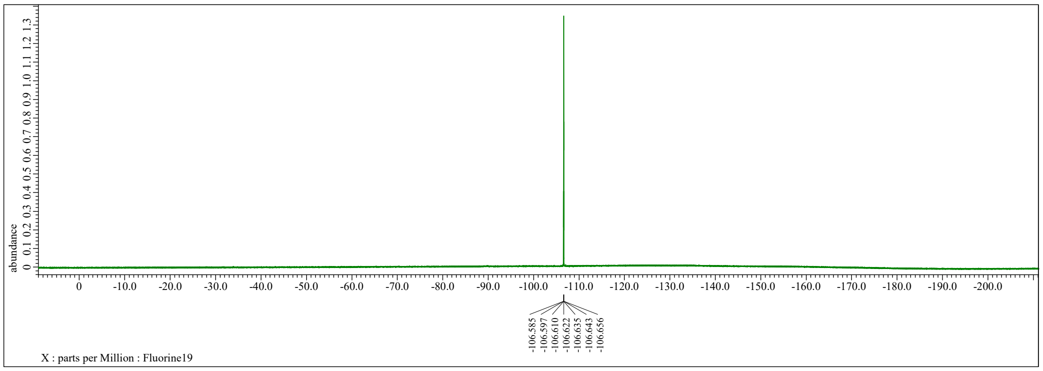

***N*-benzyl-4-fluoro-*N*-(2-(pent-1-yn-1-yl)phenyl)benzenesulfonamide 1q**

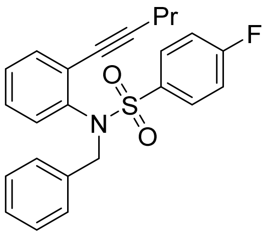

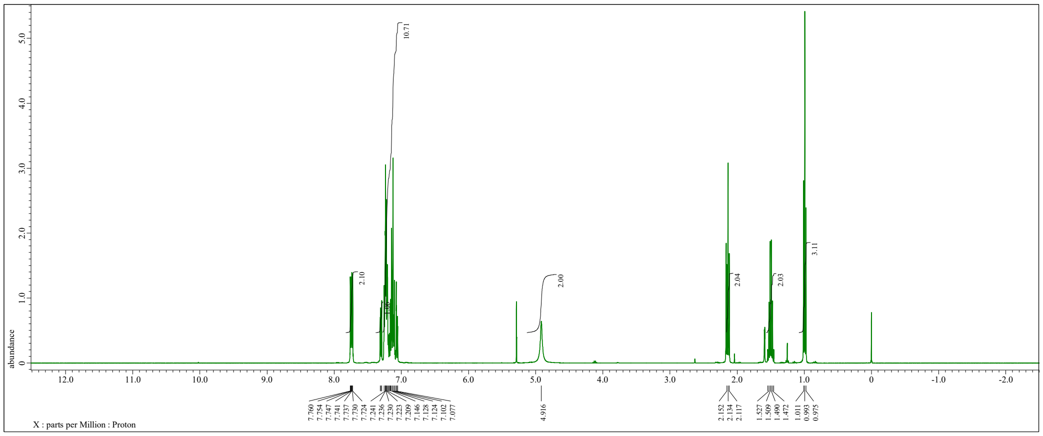

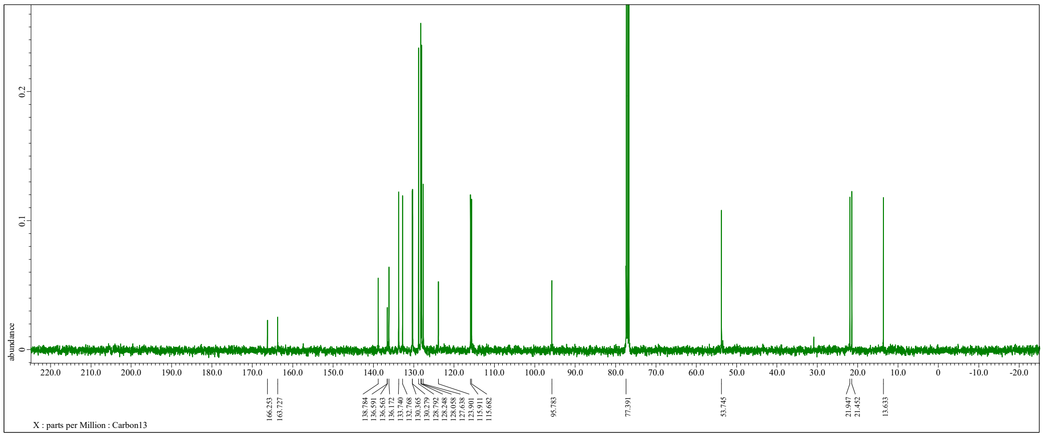

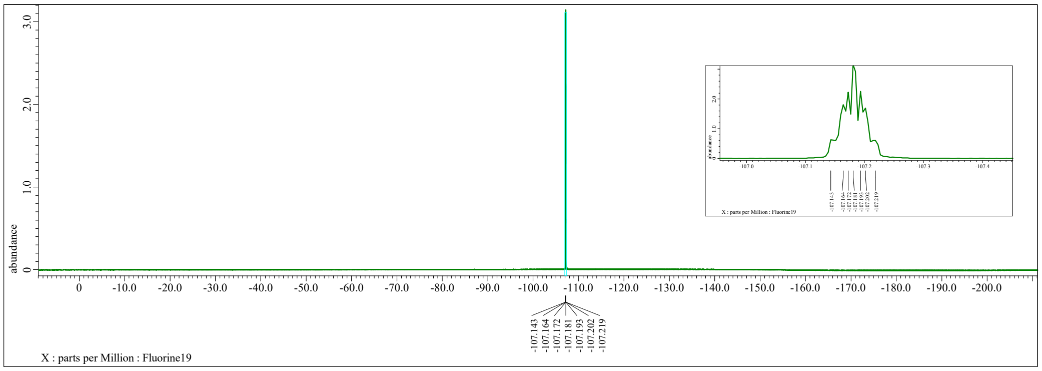

_N_,4-dimethyl-_N_-(2-(phenylethynyl)phenyl)benzenesulfonamide 1r

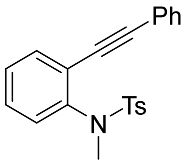

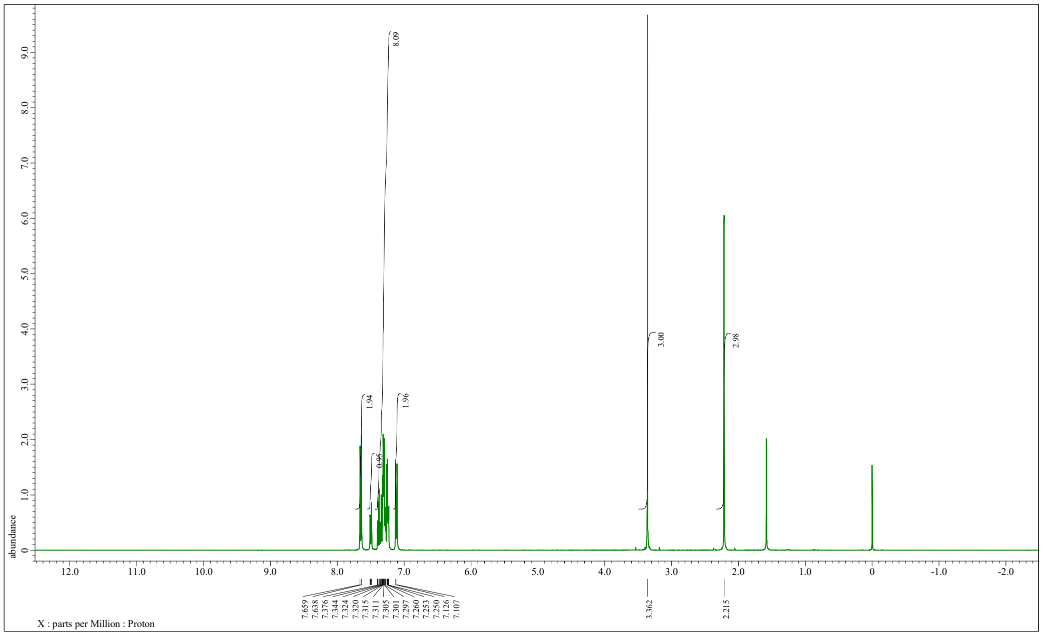

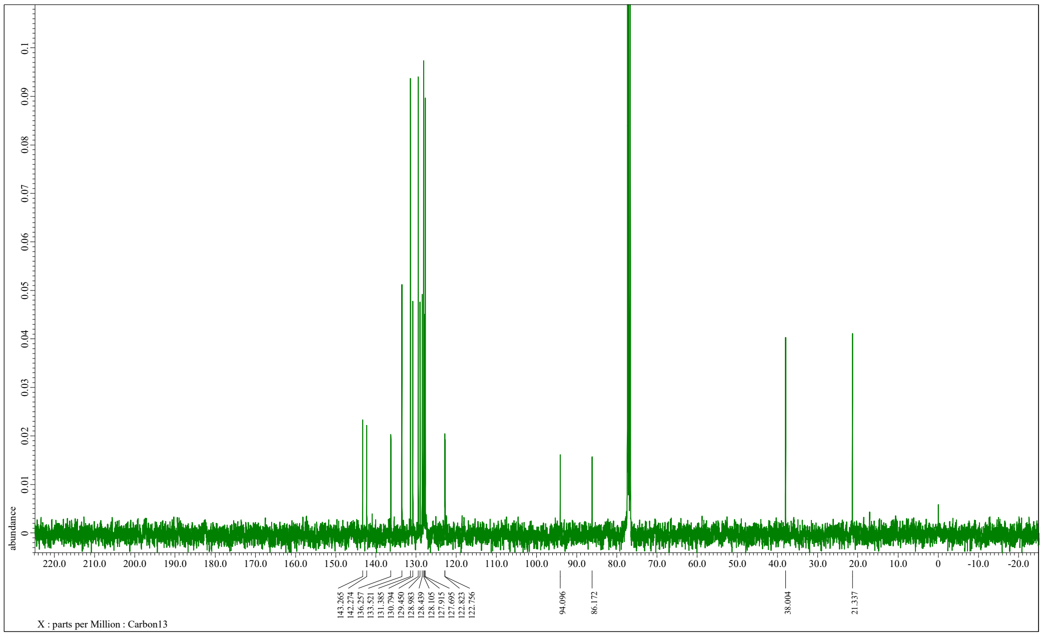

1-Methyl-2-propyl-4-tosyl-1H-indole (2a)

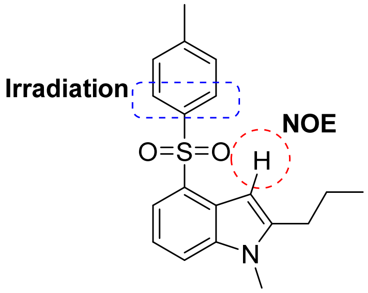

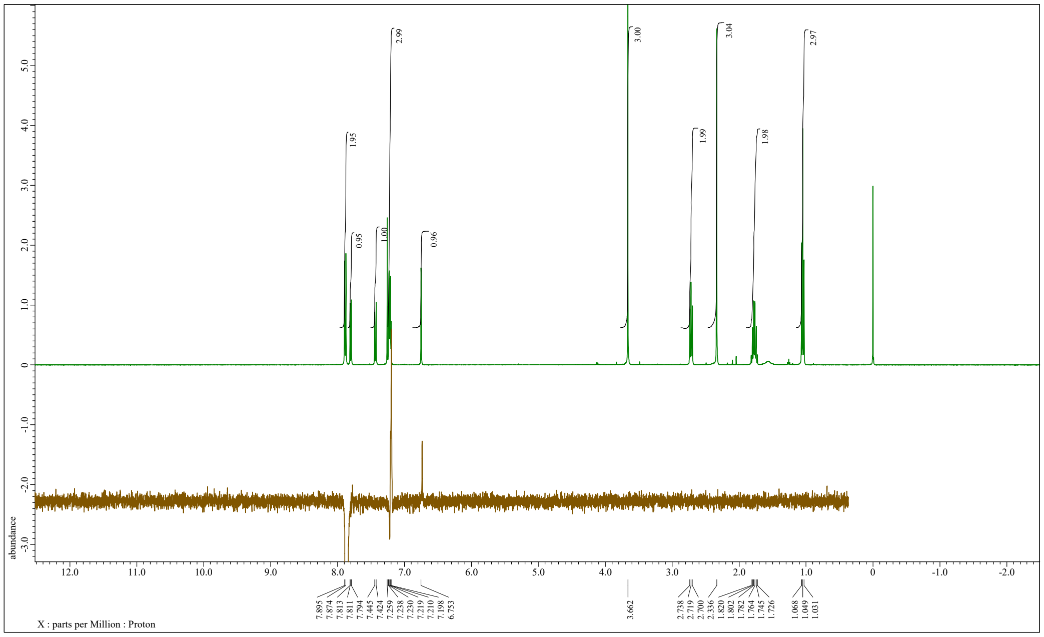

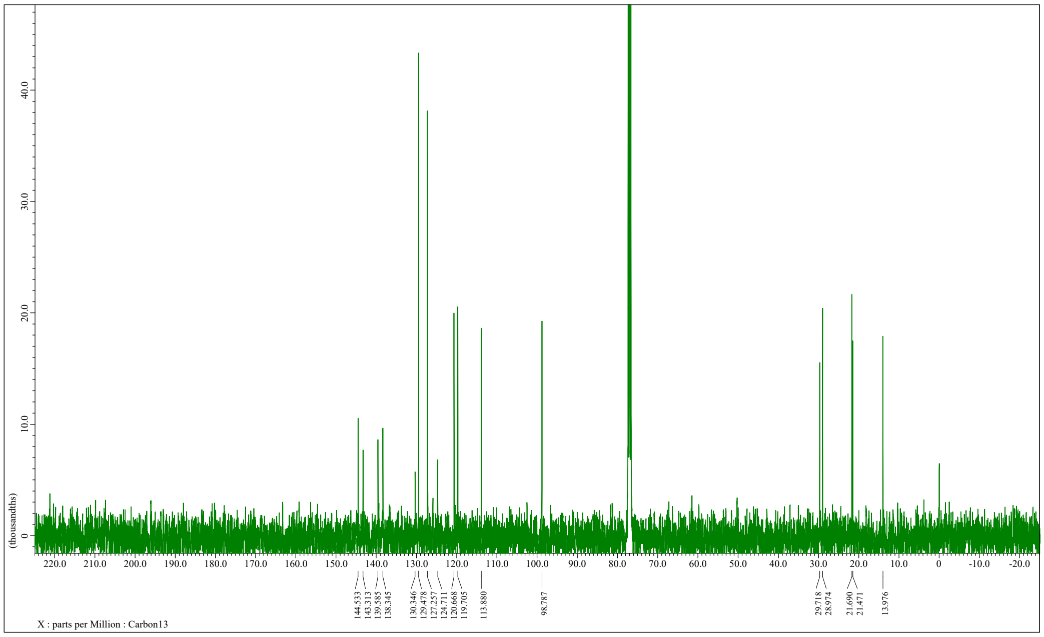

1-Methyl-2-propyl-3-tosyl-1H-indole (3a)

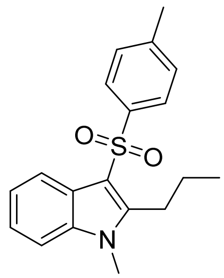

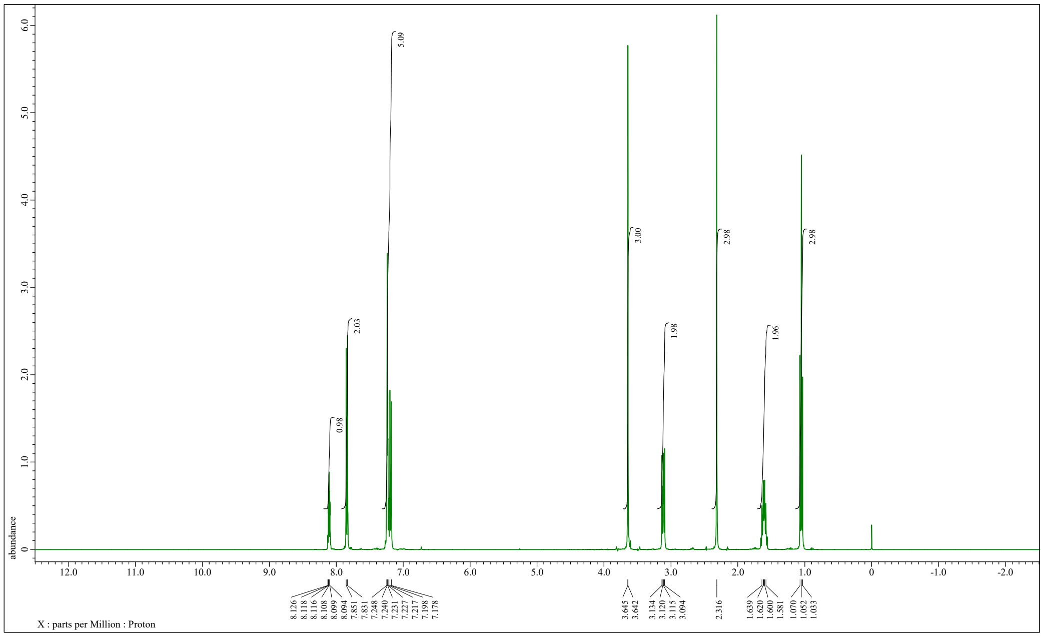

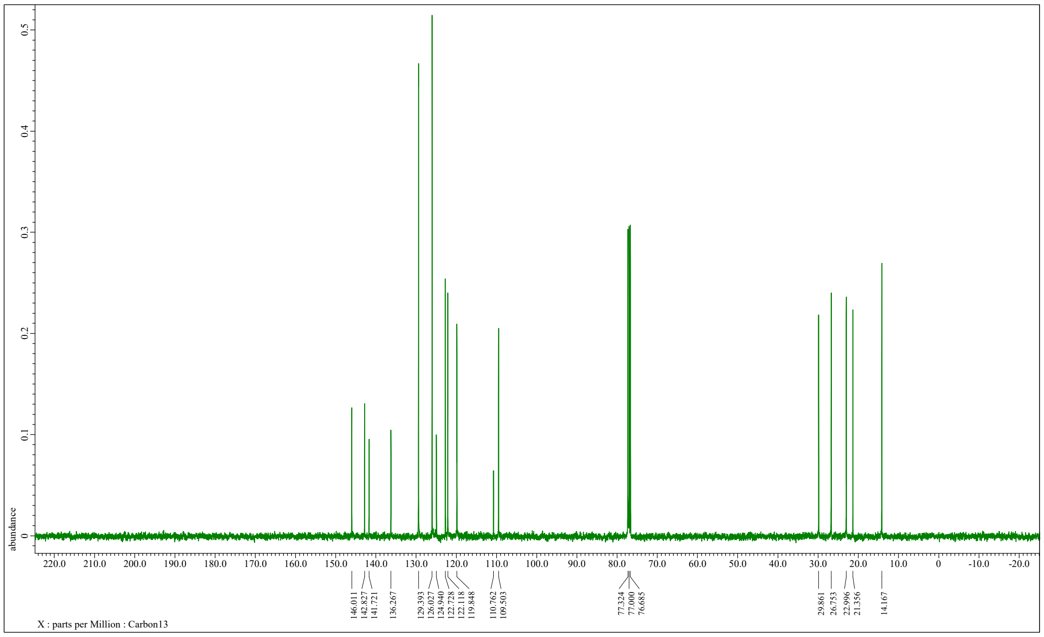

1-Methyl-2-propyl-6-tosyl-1H-indole (4a)

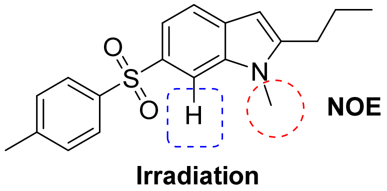

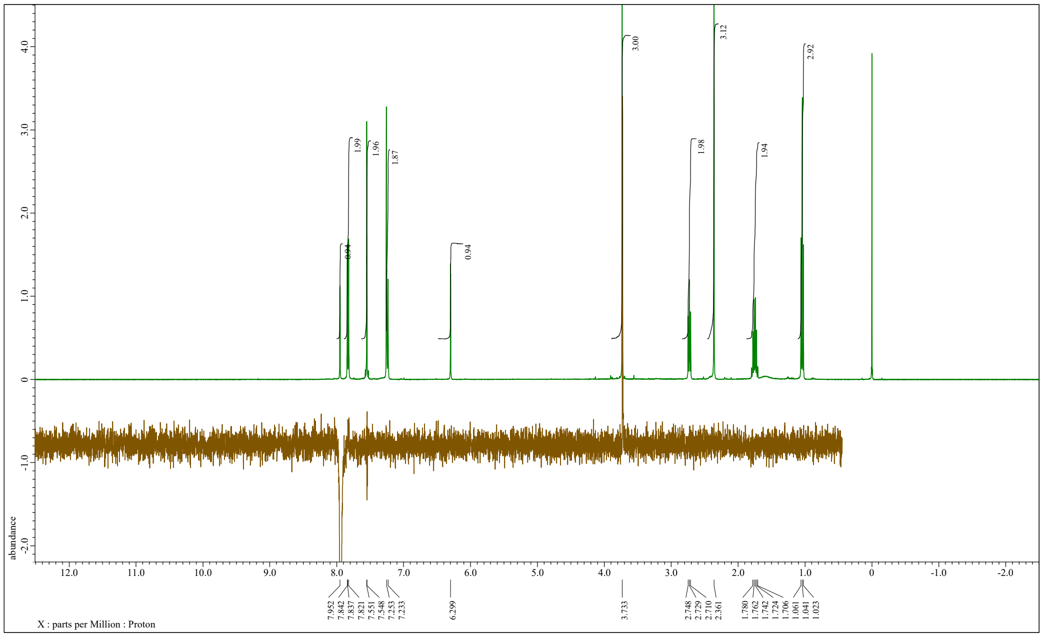

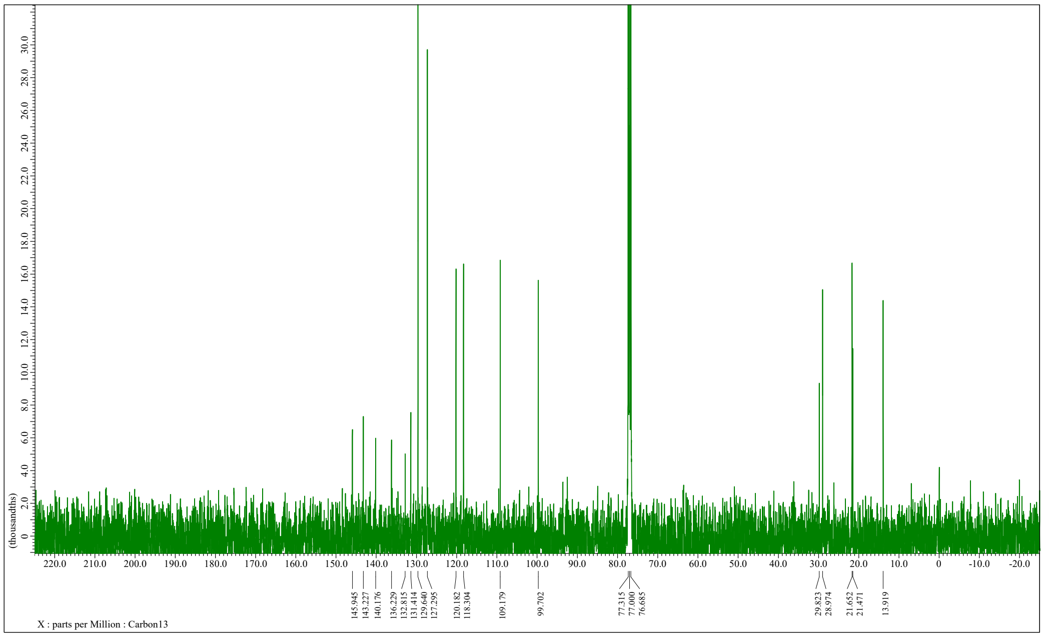

4-((4-Methoxyphenyl)sulfonyl)-1-methyl-2-propyl-1*H*-indole (2b)

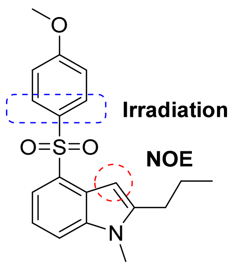

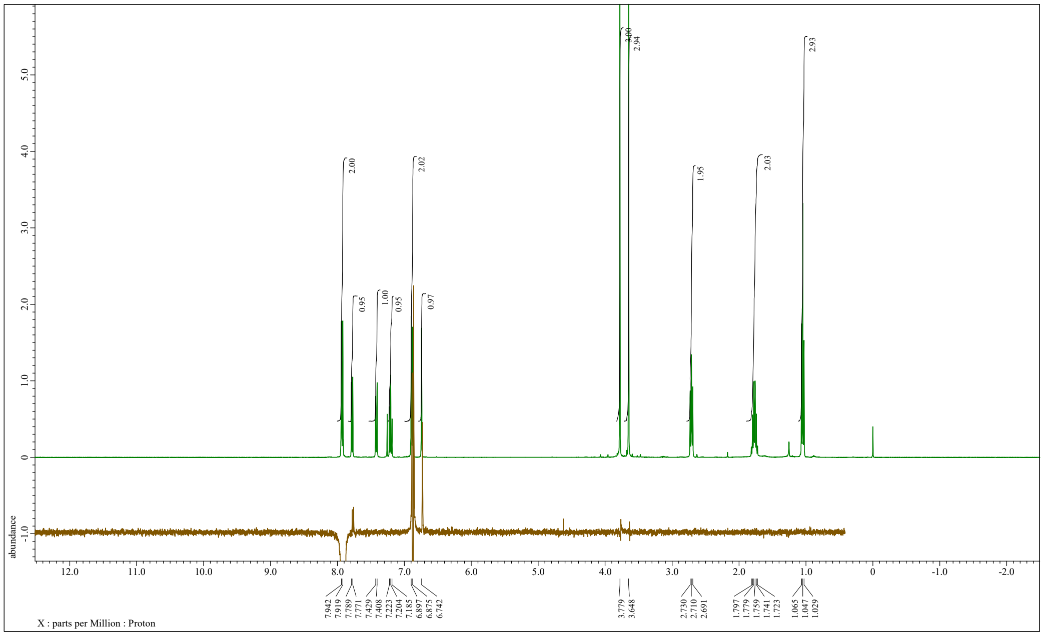

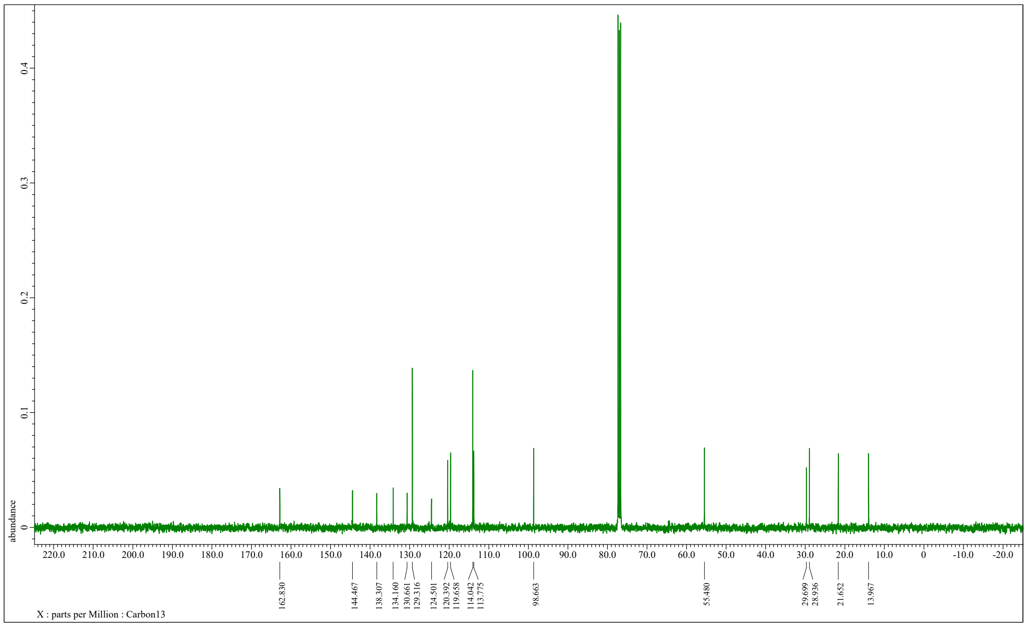

3-((4-Methoxyphenyl)sulfonyl)-1-methyl-2-propyl-1*H*-indole (3b)

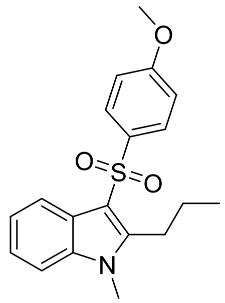

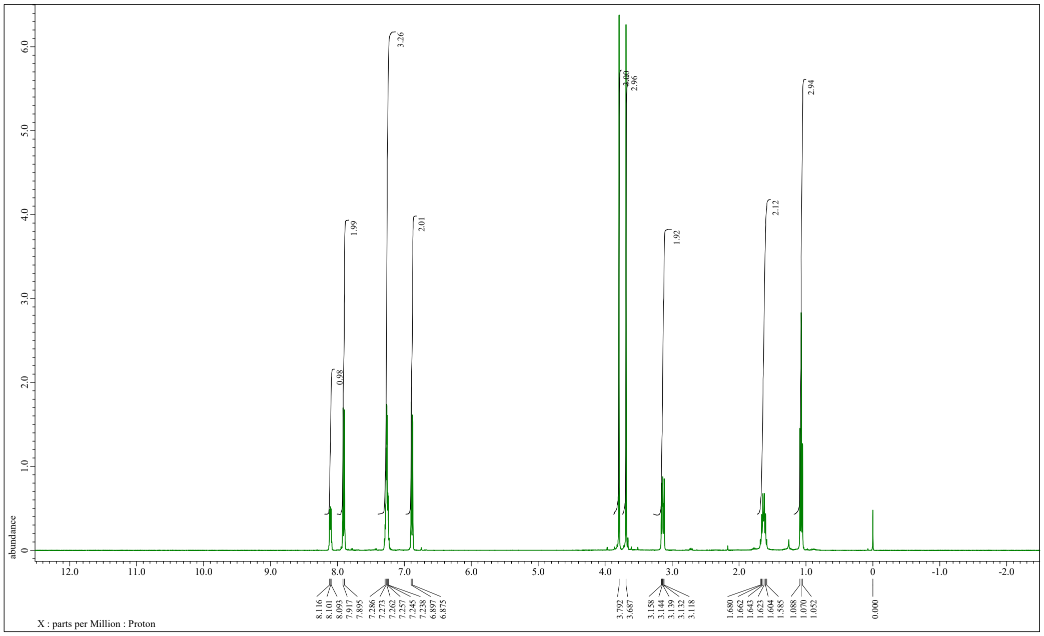

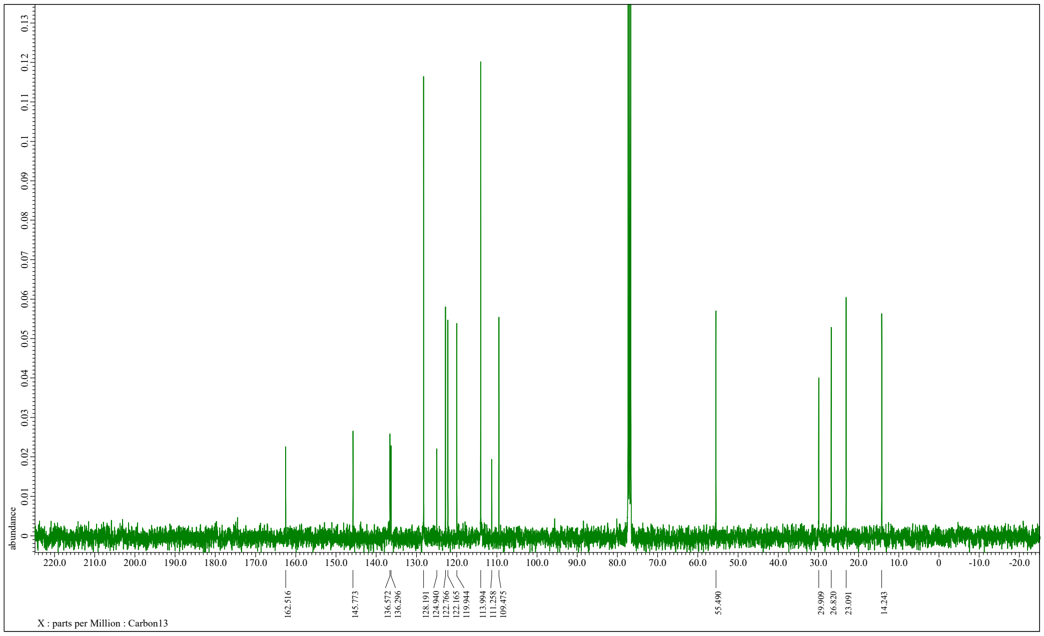

6-((4-Methoxyphenyl)sulfonyl)-1-methyl-2-propyl-1*H*-indole (4b)

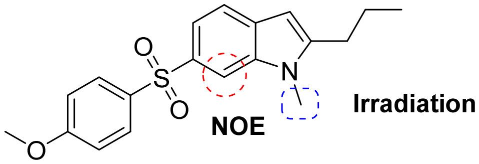

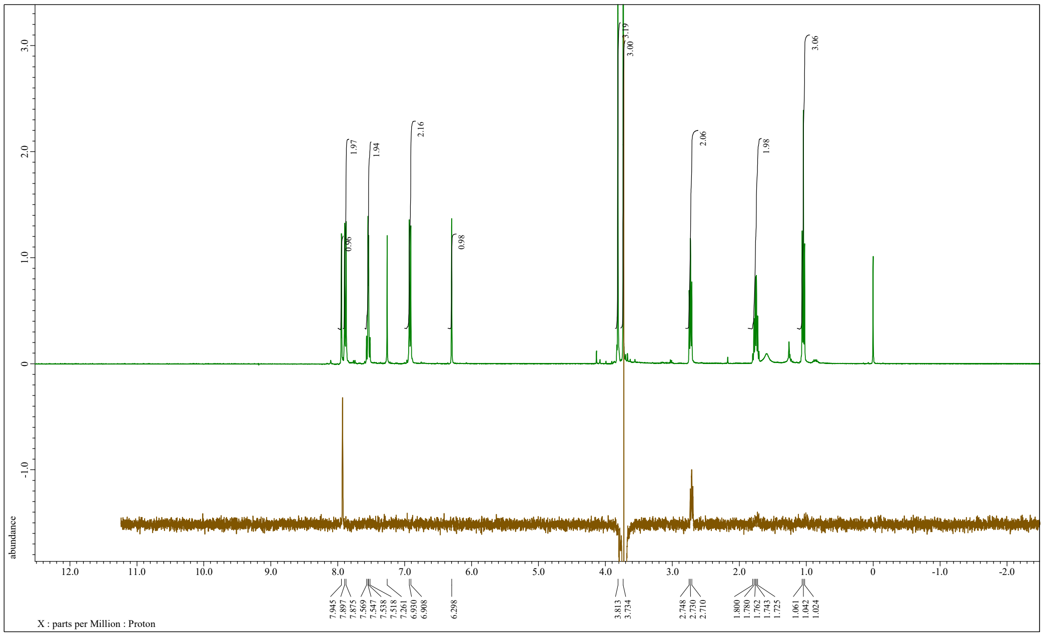

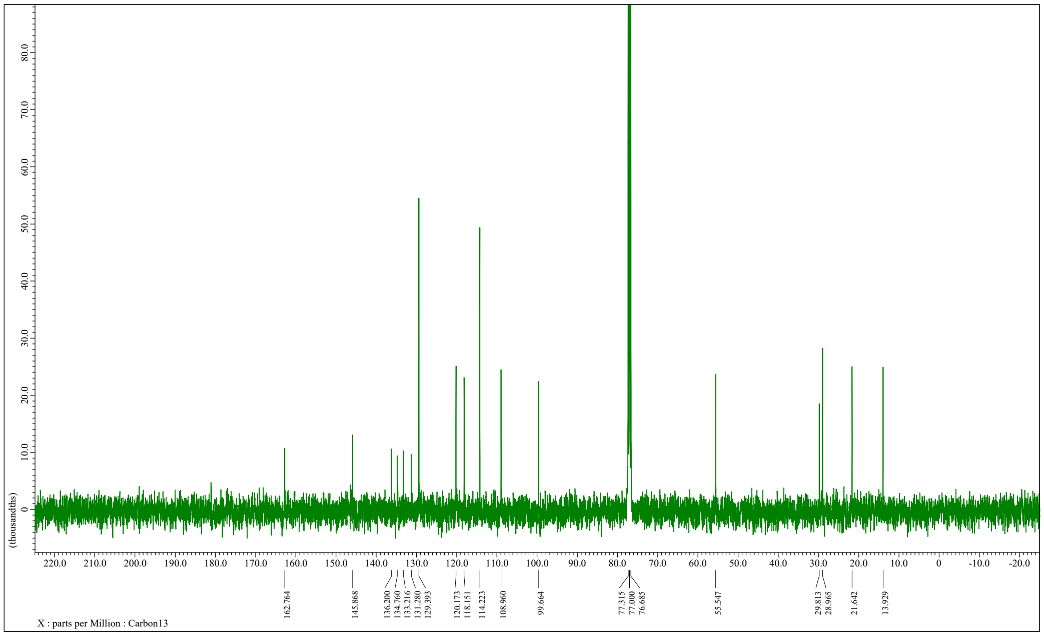

1-methyl-2-propyl-4-(*m*-tolylsulfonyl)-1*H*-indole (2c)

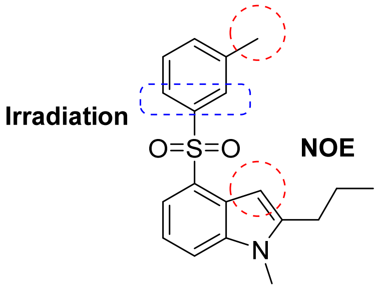

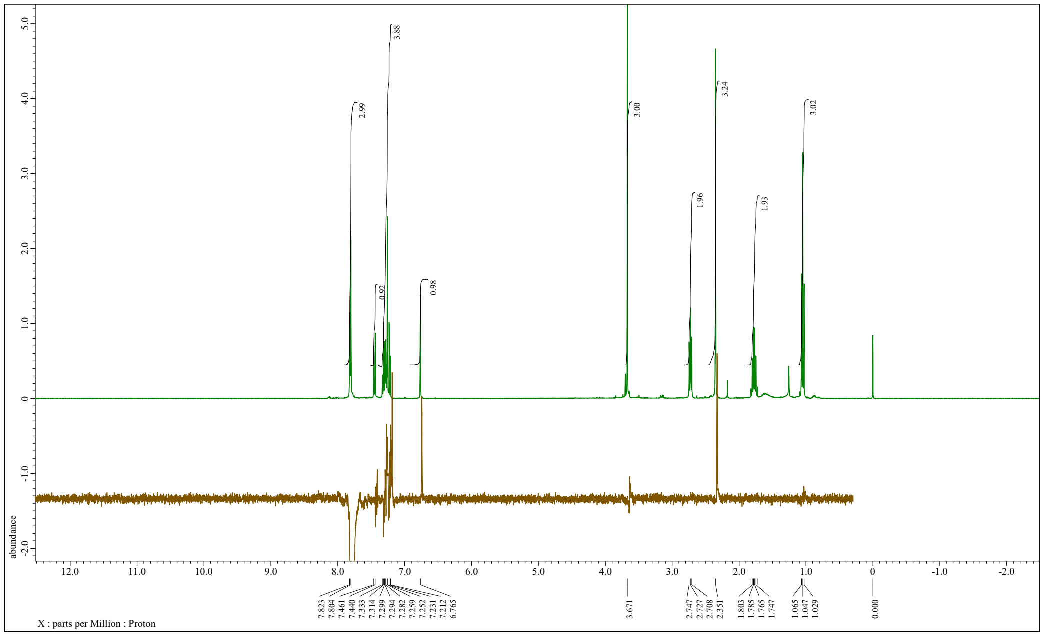

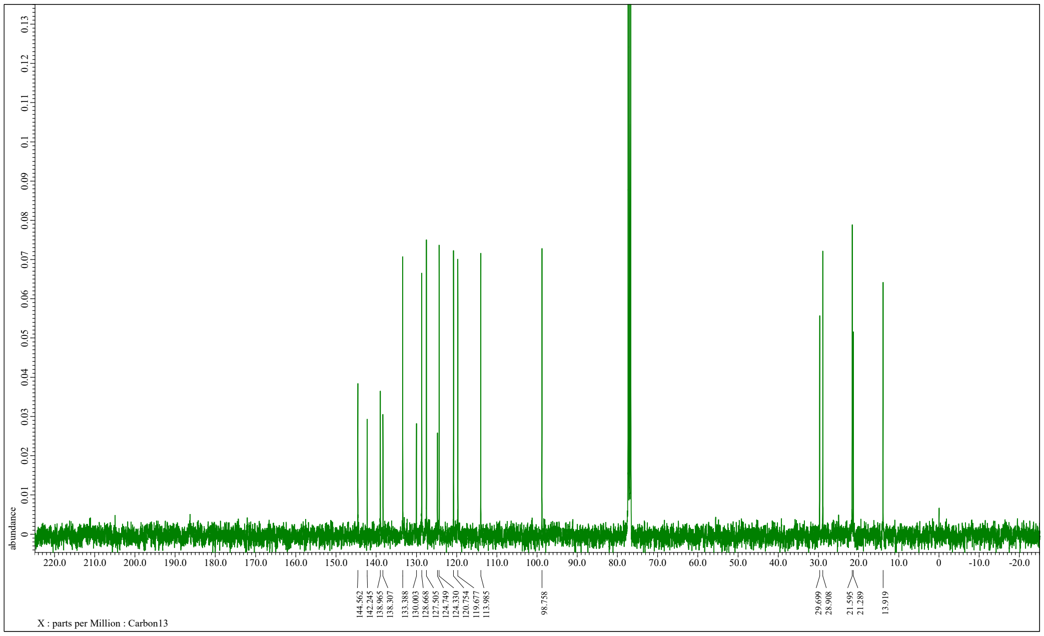

1-Methyl-2-propyl-3-(*m*-tolylsulfonyl)-1*H*-indole (3c)

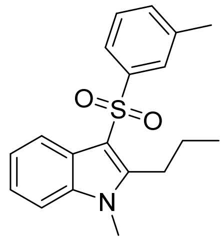

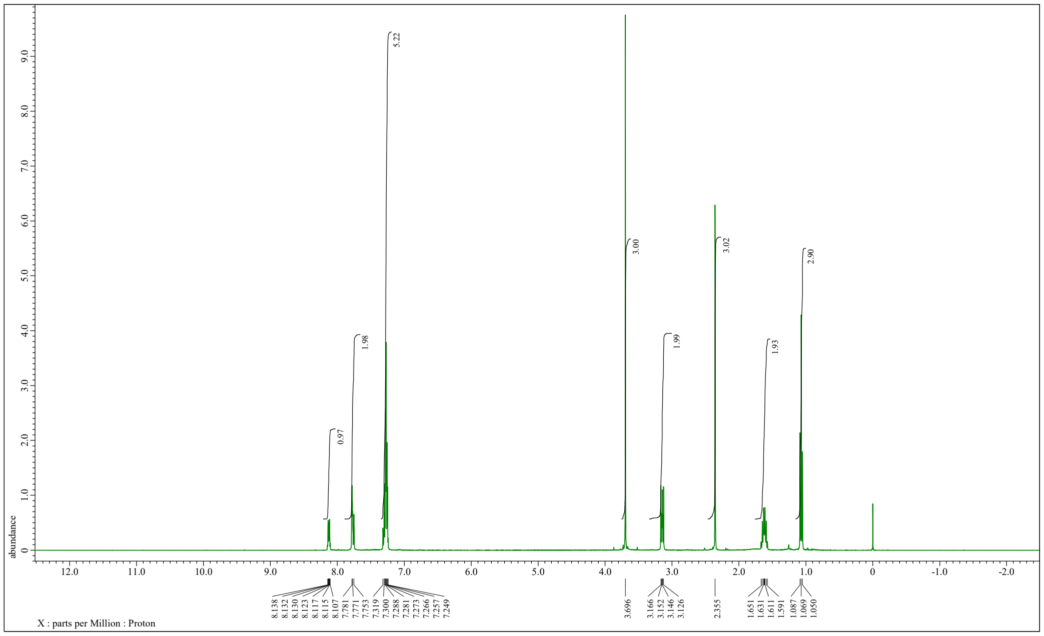

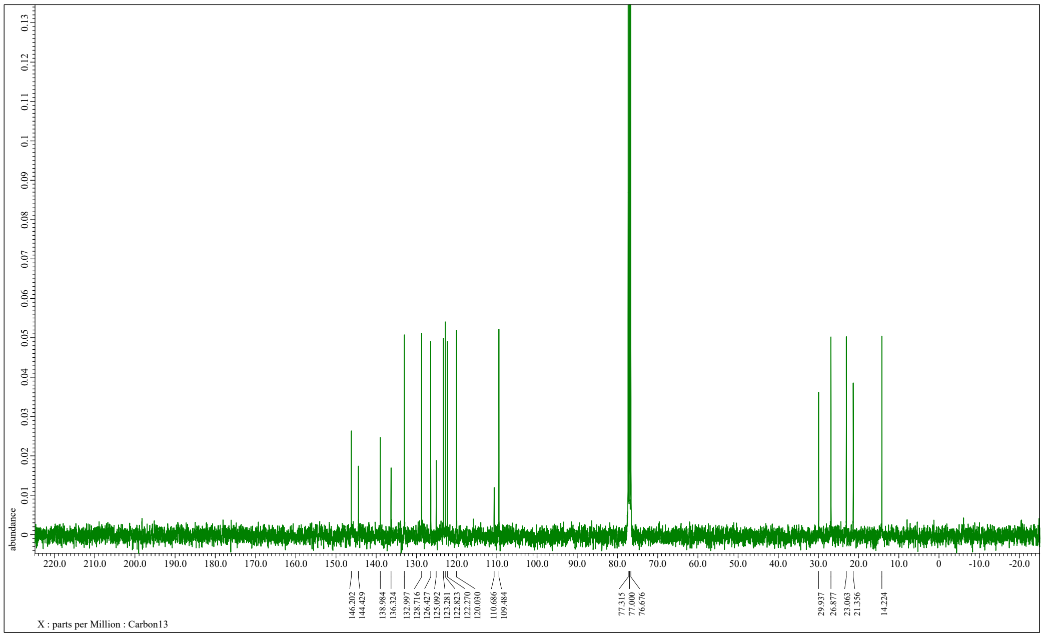

1-Methyl-2-propyl-6-(*m*-tolylsulfonyl)-1*H*-indole (4c)

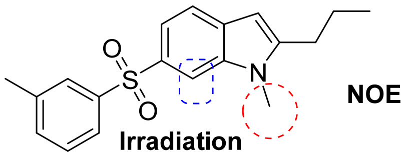

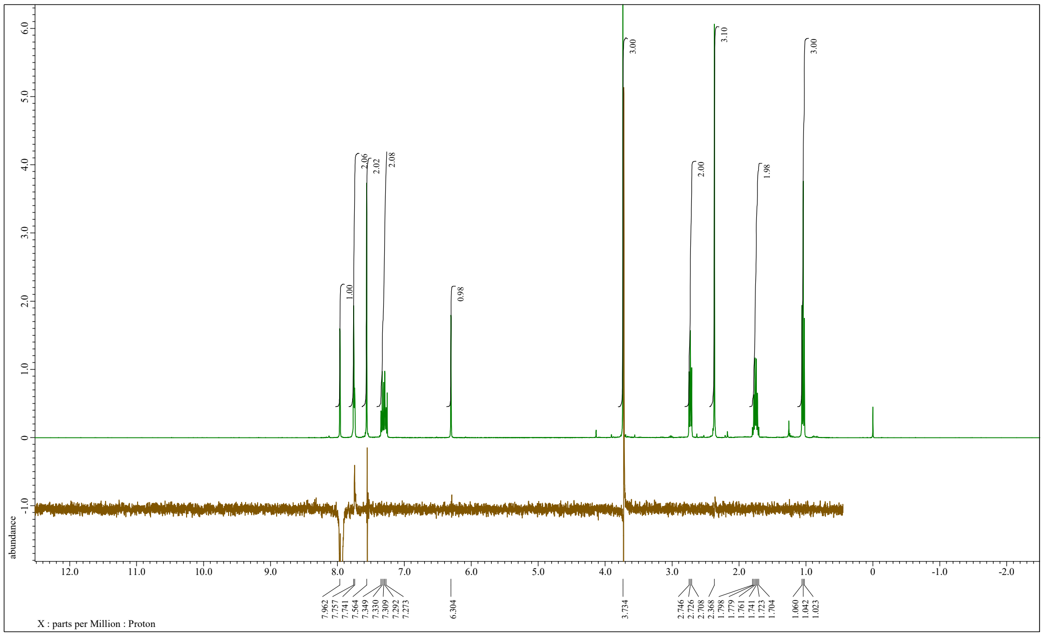

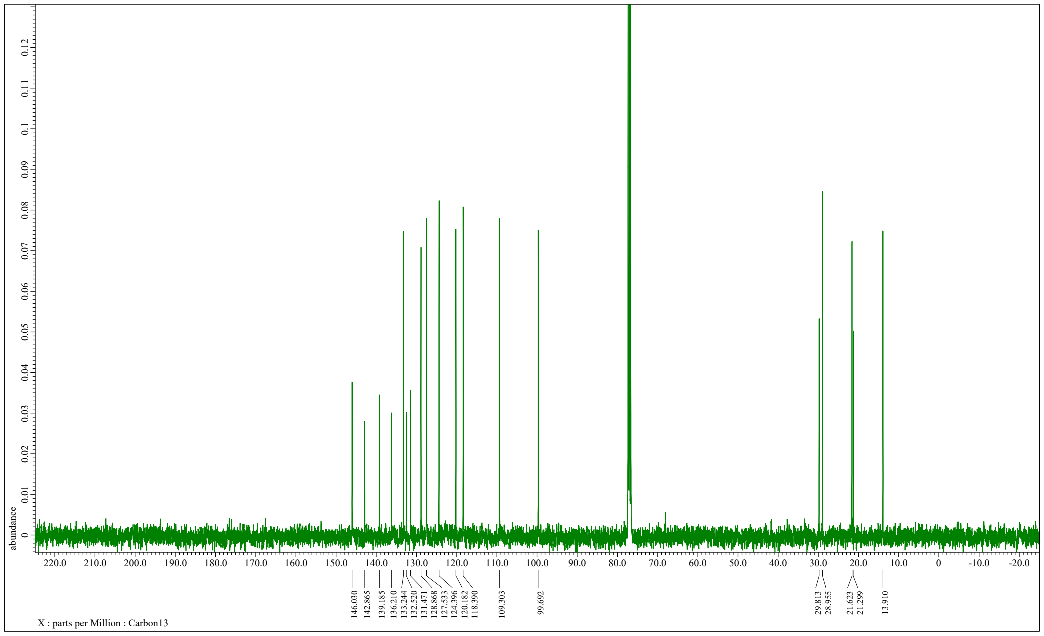

1-Methyl-2-propyl-4-(*o*-tolylsulfonyl)-1*H*-indole (2d)

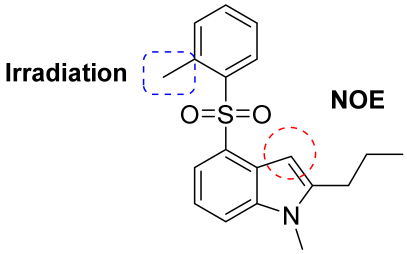

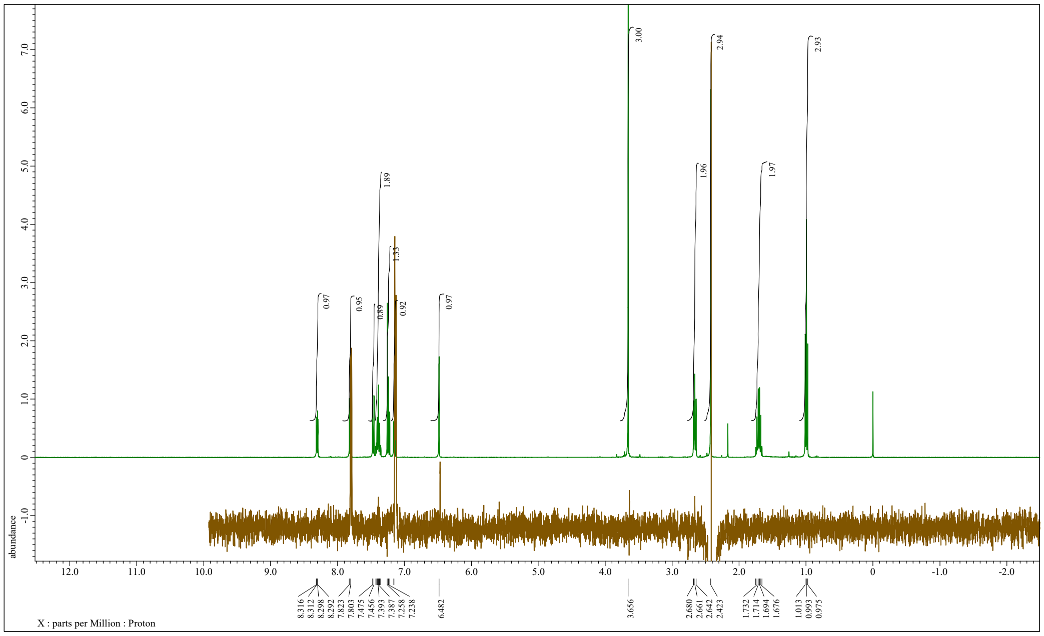

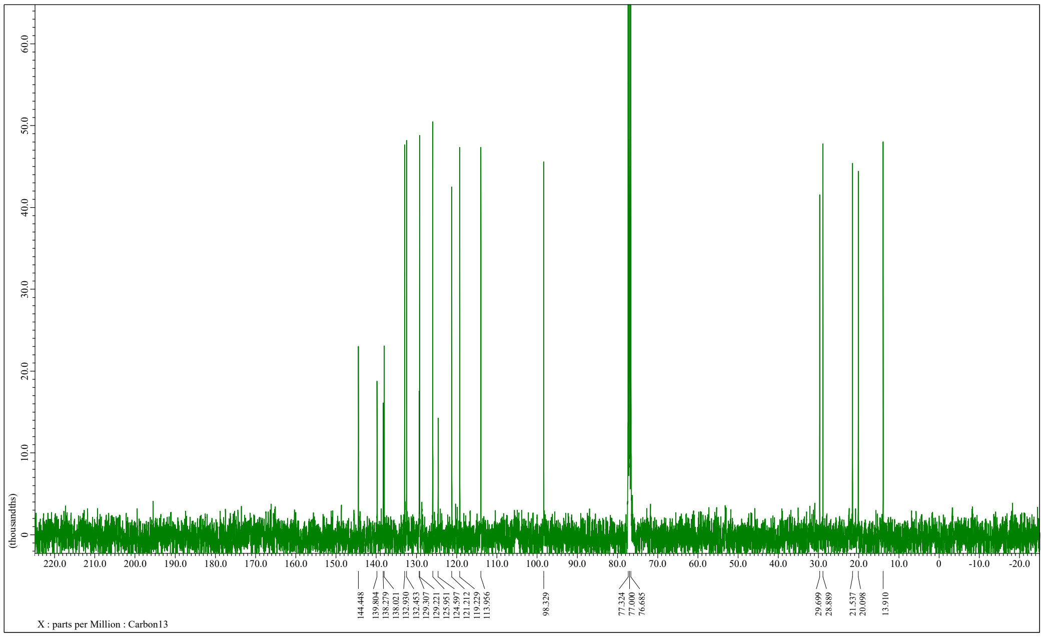

1-Methyl-2-propyl-3-(*o*-tolylsulfonyl)-1*H*-indole (3d)

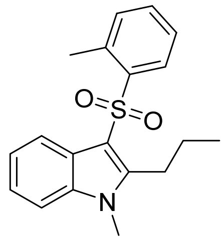

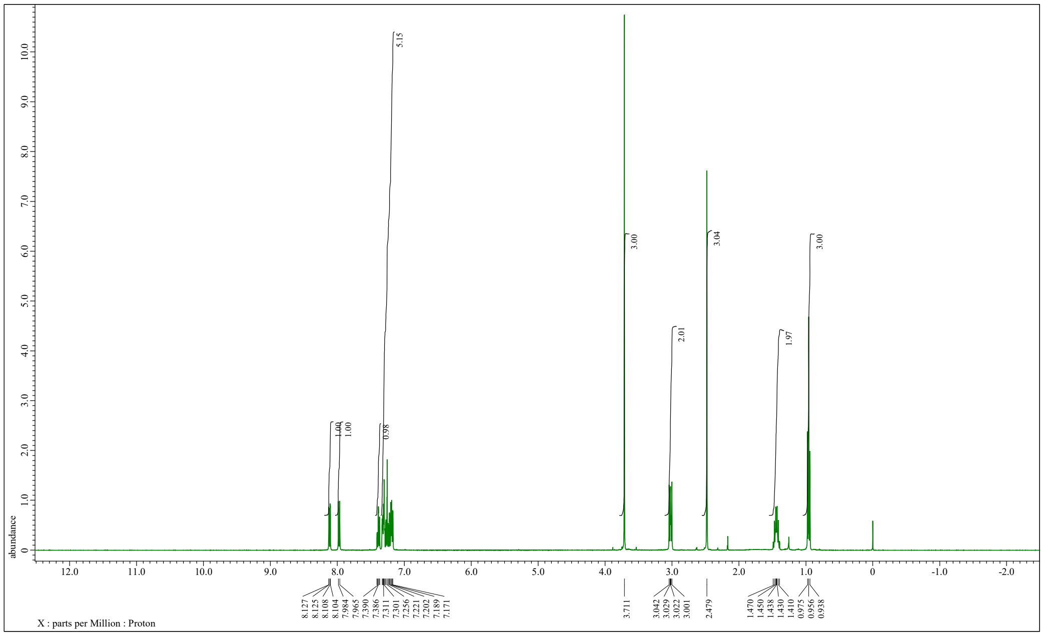

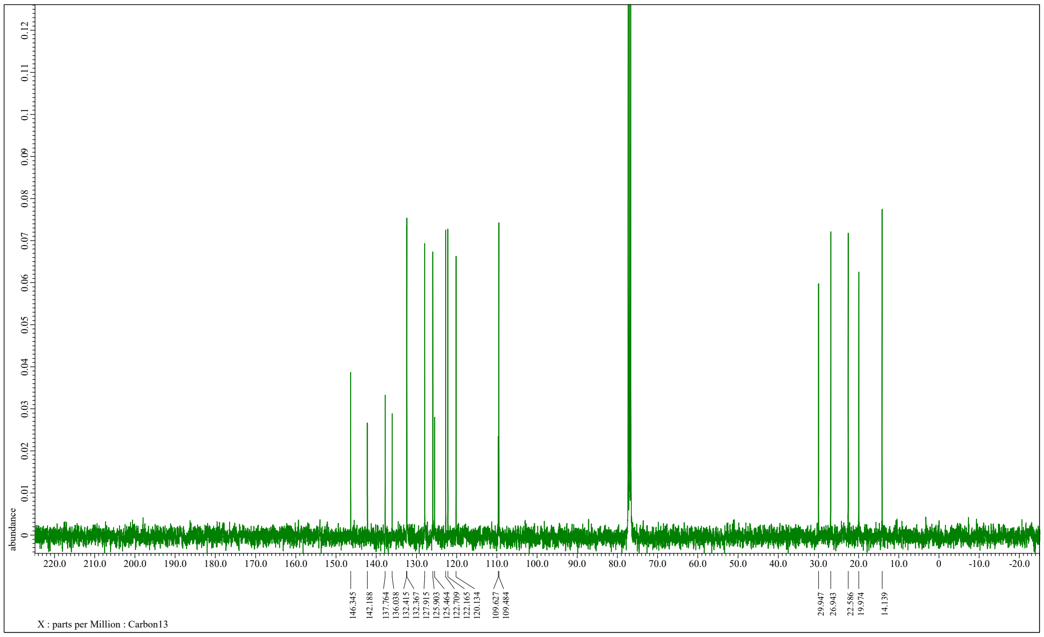

1-methyl-2-propyl-4-(*o*-tolylsulfonyl)-1*H*-indole (4d)

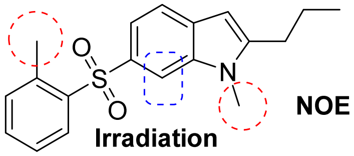

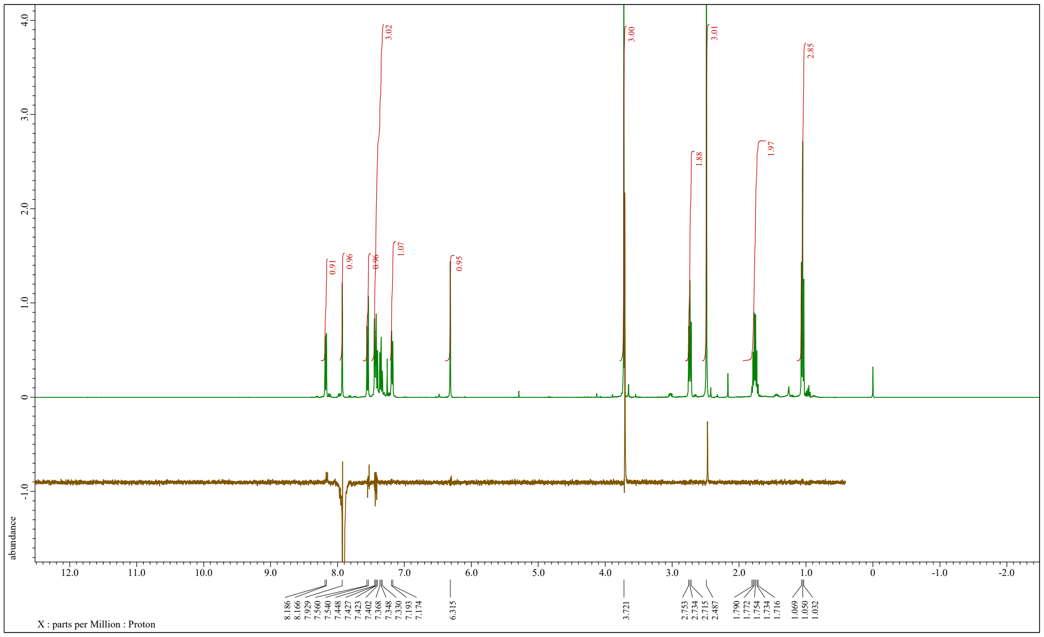

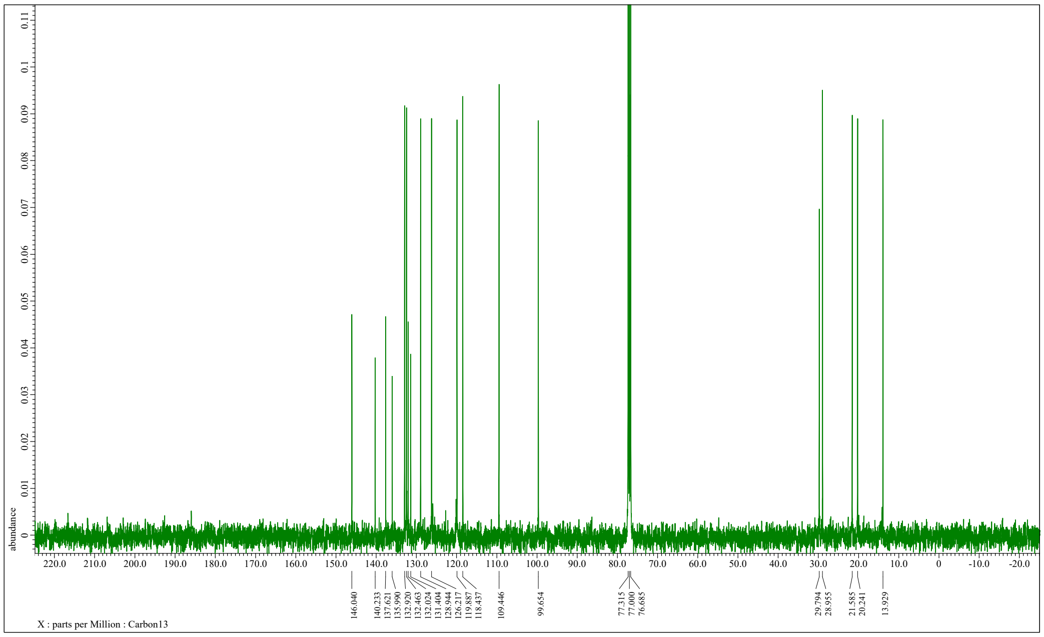

1-Methyl-2-propyl-4-((4-(trifluoromethyl)phenyl)sulfonyl)-1*H*-indole (2e)

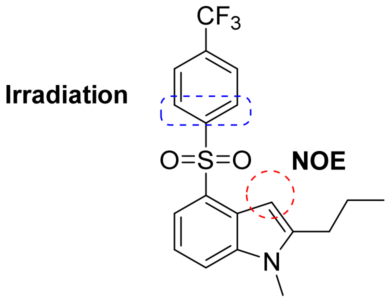

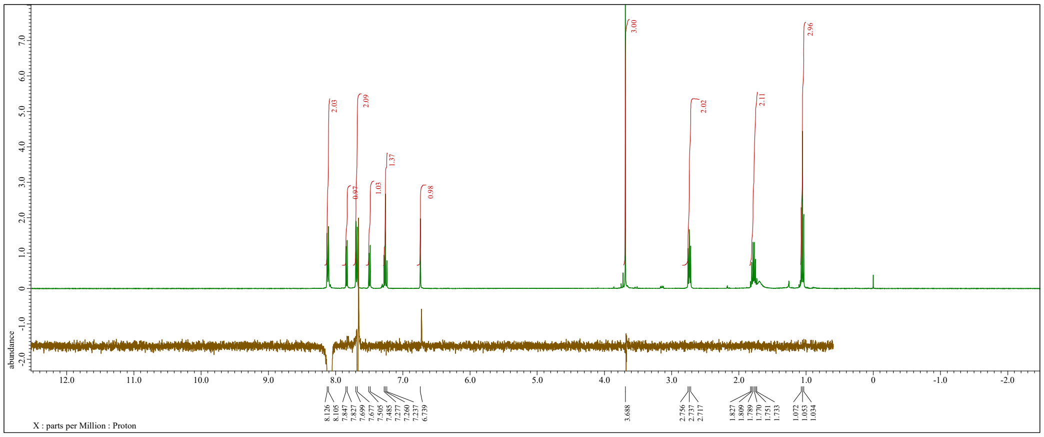

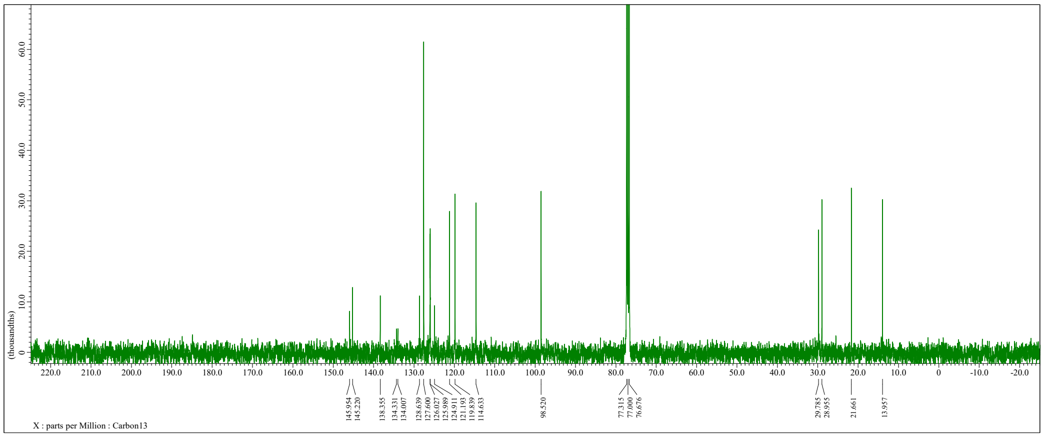

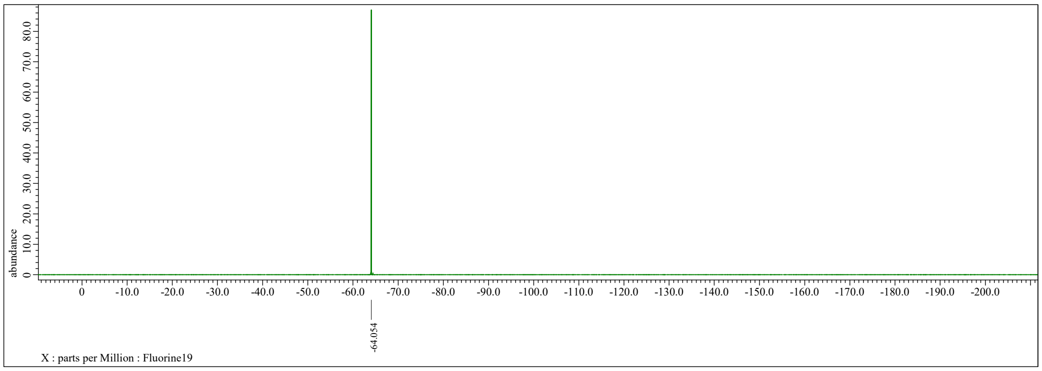

1-Methyl-2-propyl-3-((4-(trifluoromethyl)phenyl)sulfonyl)-1*H*-indole (3e)

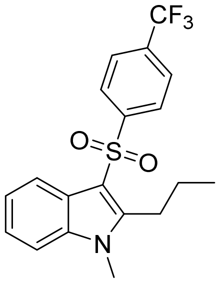

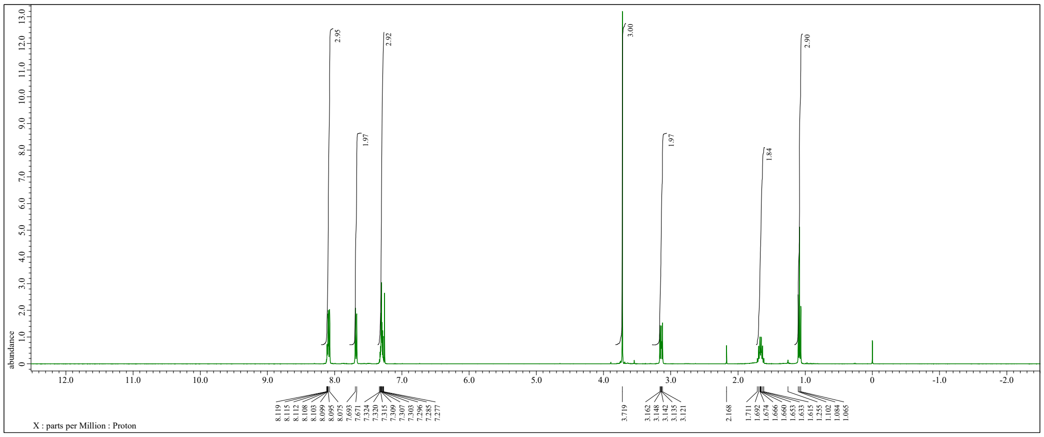

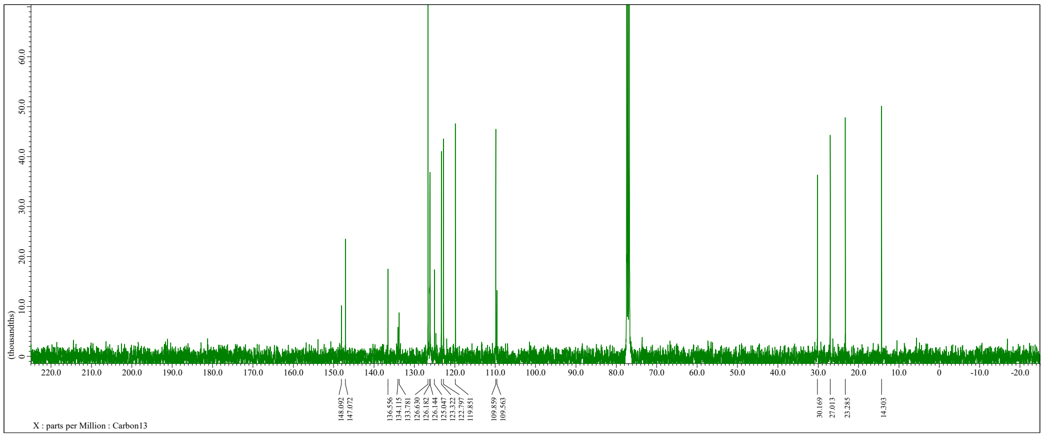

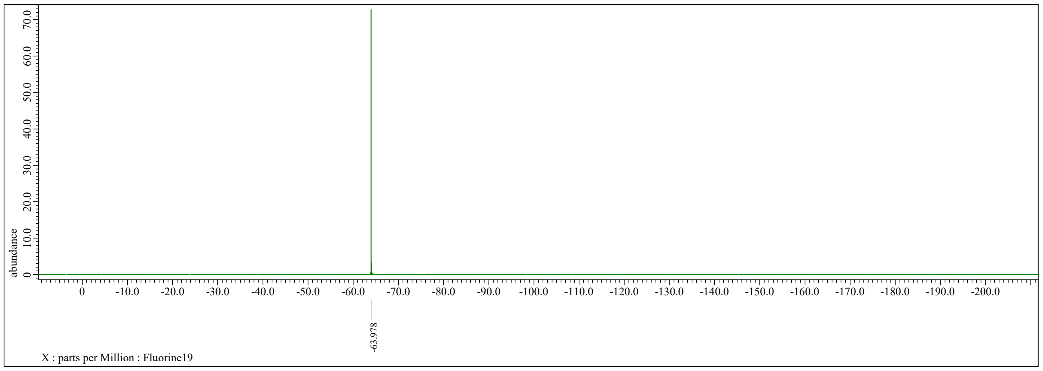

1-Methyl-2-propyl-6-((4-(trifluoromethyl)phenyl)sulfonyl)-1*H*-indole (4e)

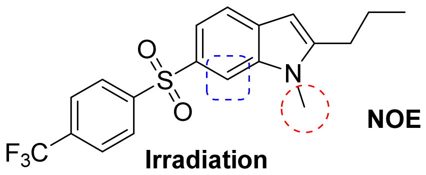

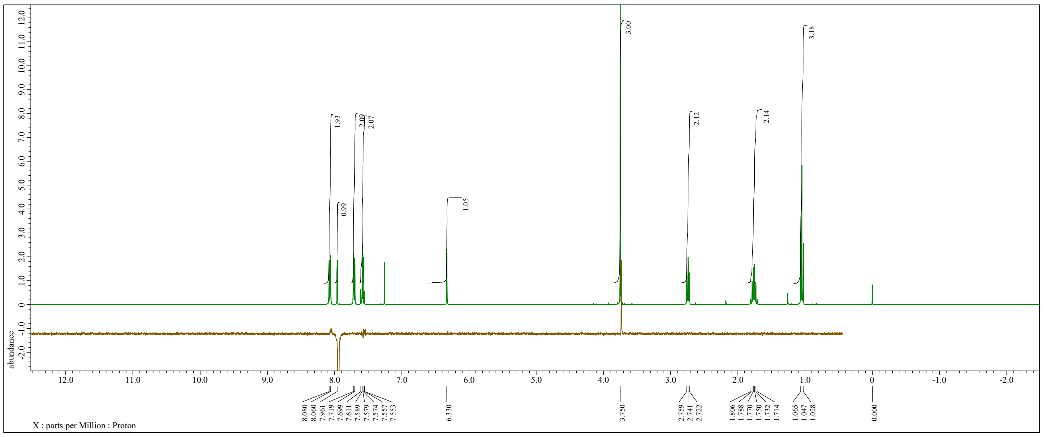

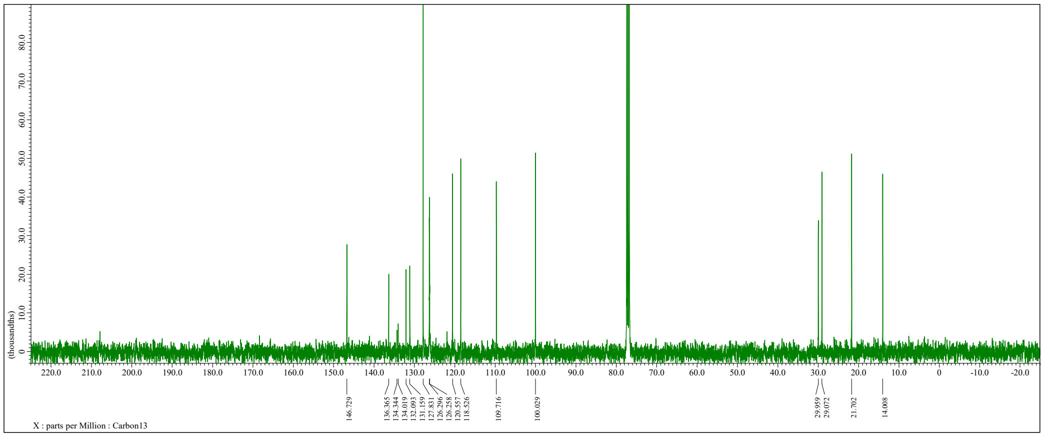

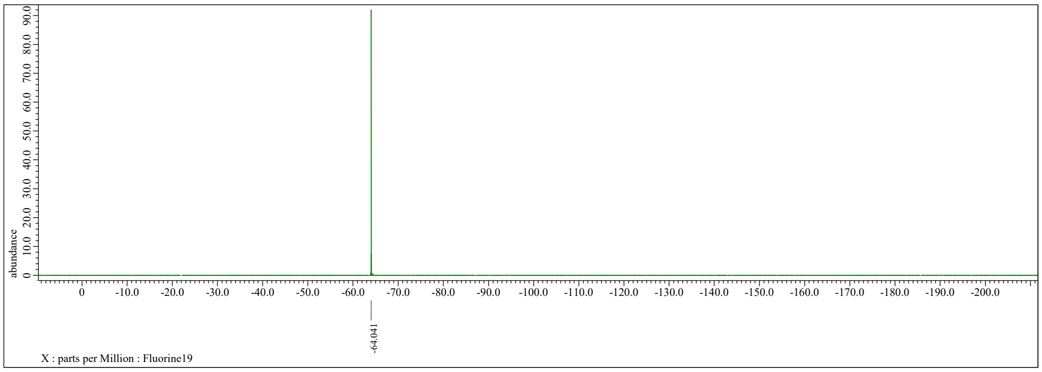

4-((4-Fluorophenyl)sulfonyl)-1-methyl-2-propyl-1*H*-indole (2f)

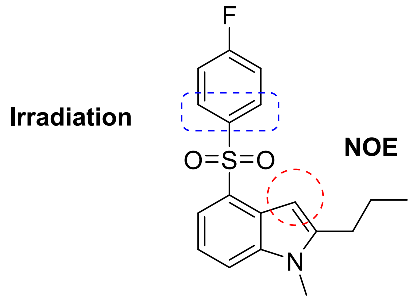

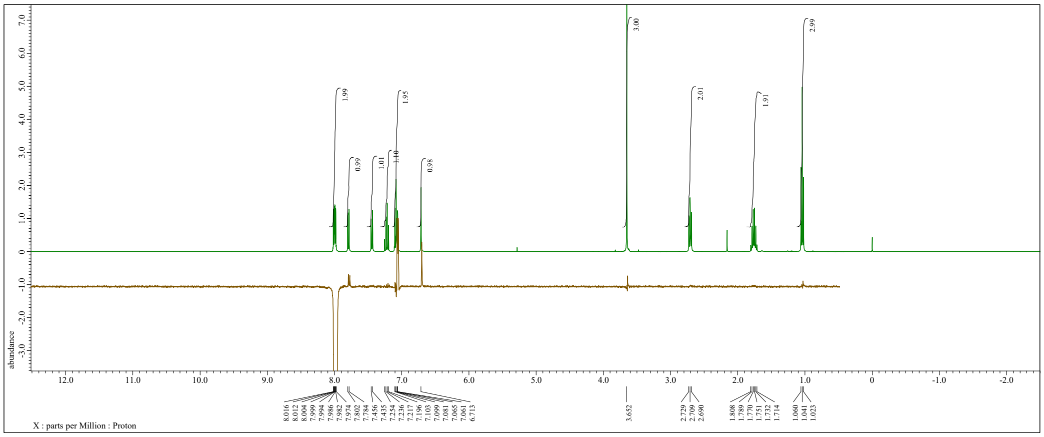

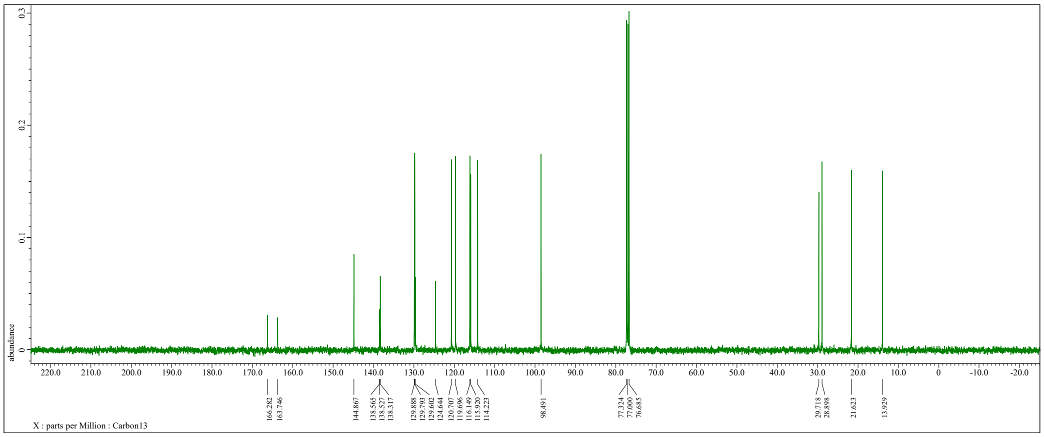

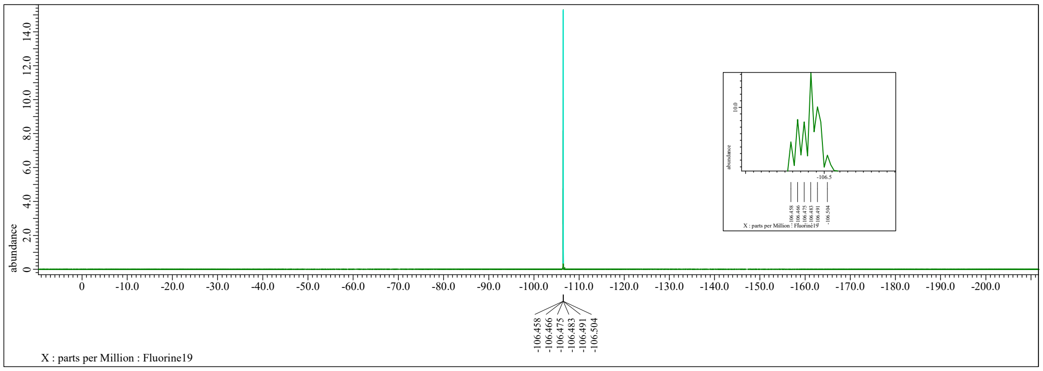

3-((4-Fluorophenyl)sulfonyl)-1-methyl-2-propyl-1*H*-indole (3f)

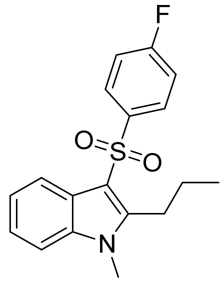

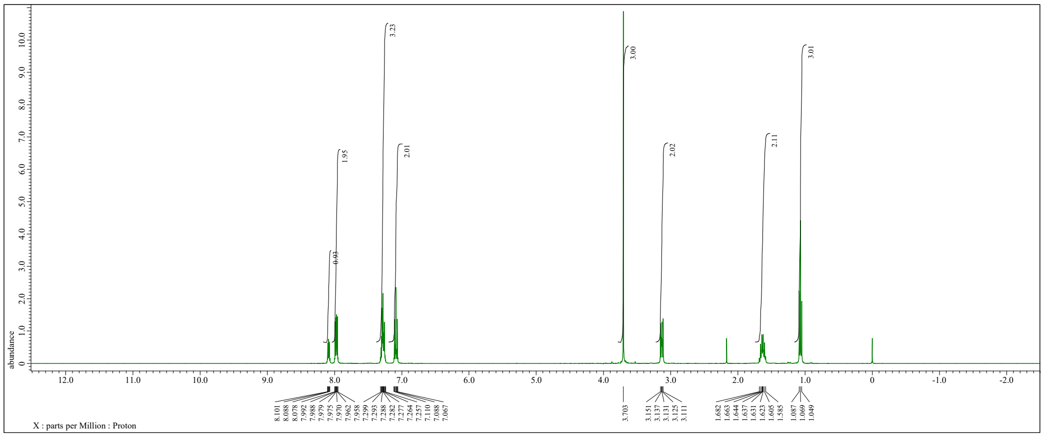

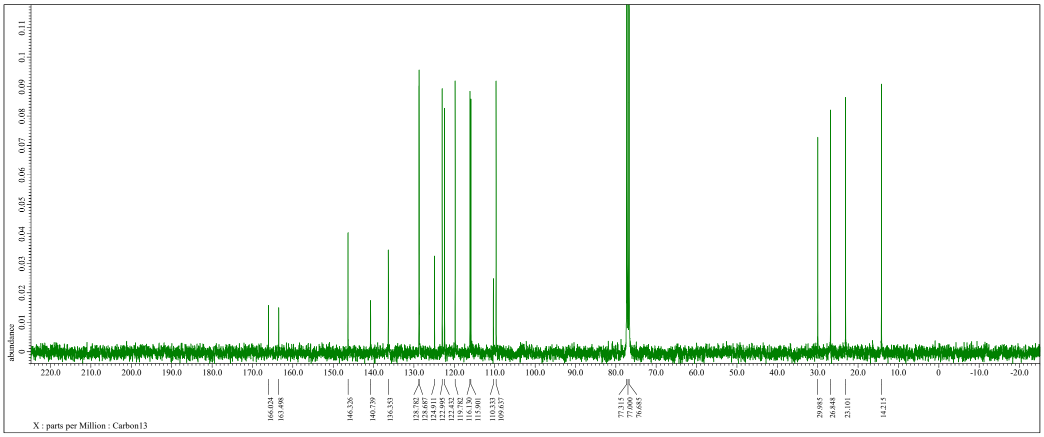

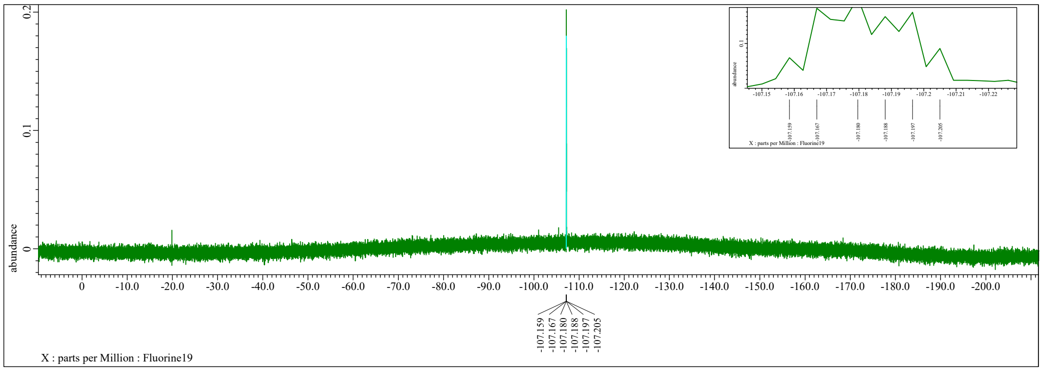

6-((4-fluorophenyl)sulfonyl)-1-methyl-2-propyl-1*H*-indole (4f)

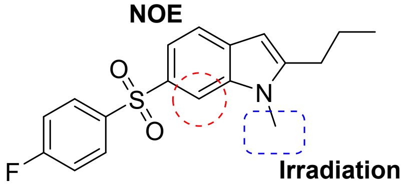

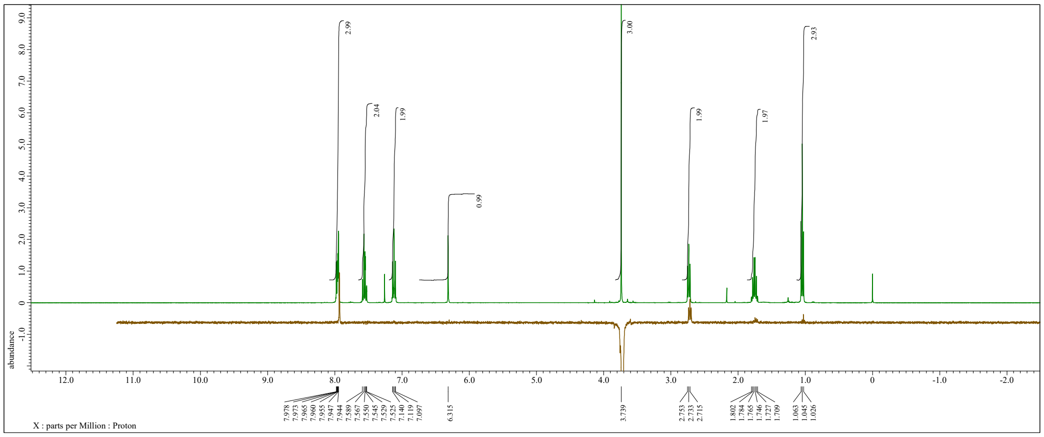

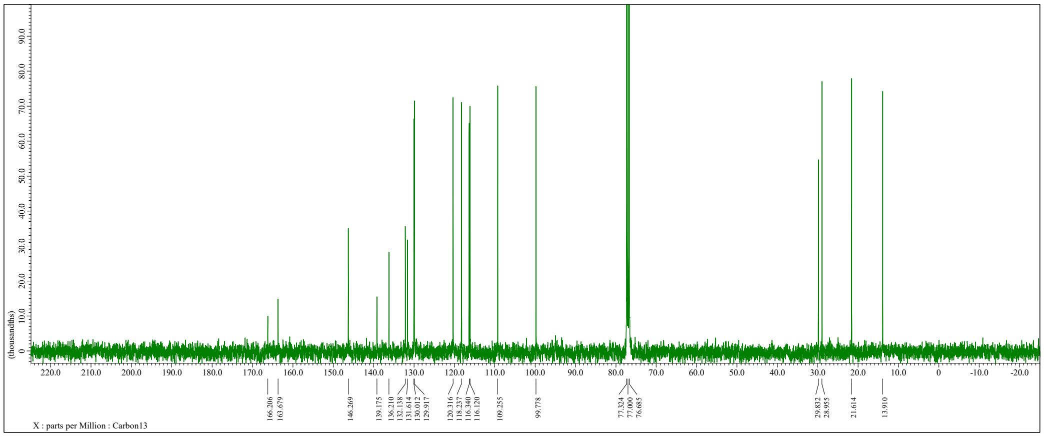

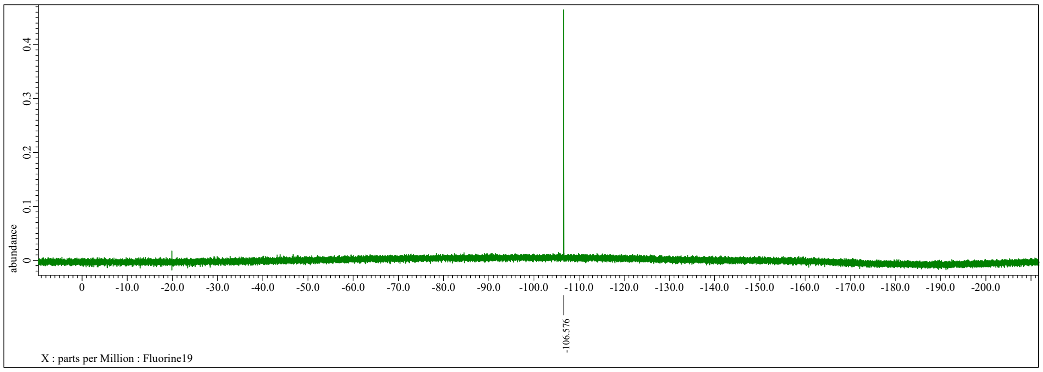

4-((4-Chlorophenyl)sulfonyl)-1-methyl-2-propyl-1*H*-indole (2g)

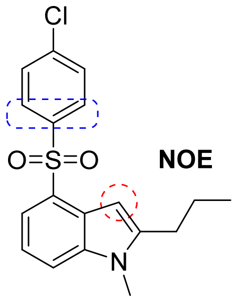

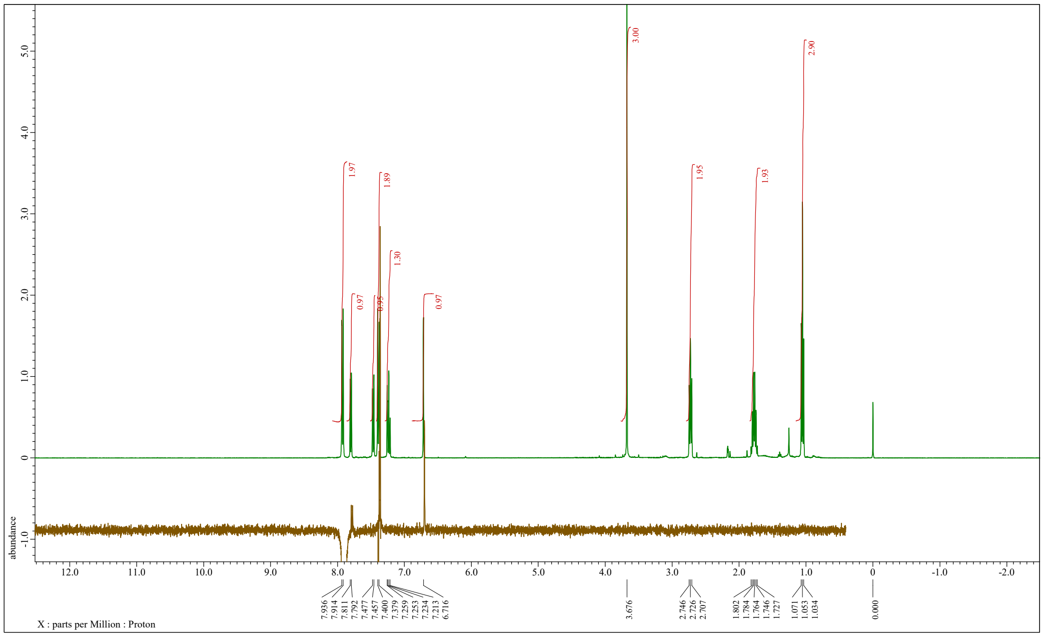

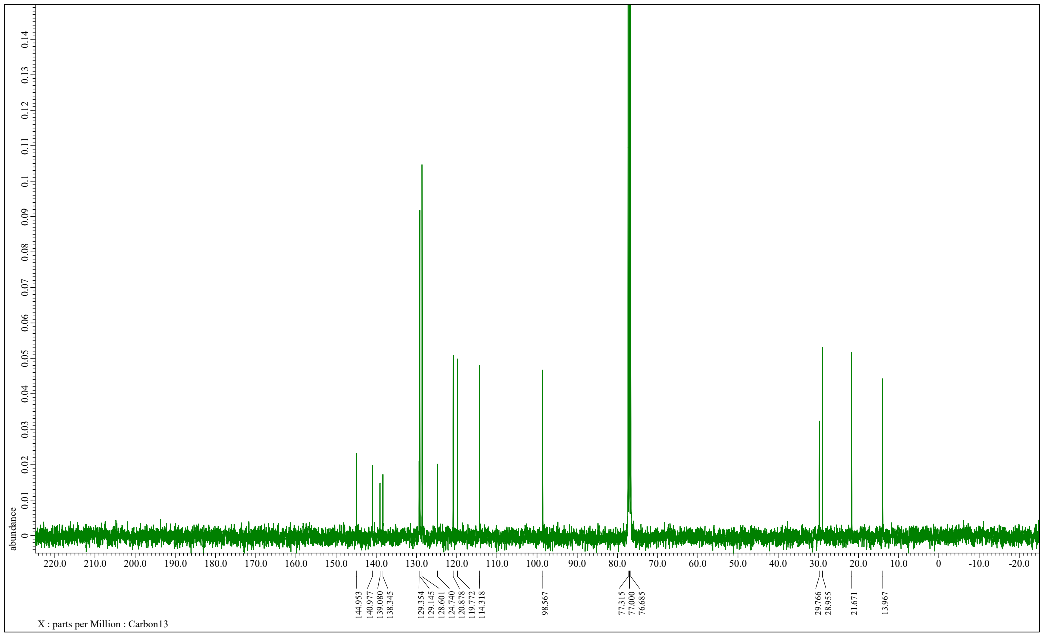

3-((4-Chlorophenyl)sulfonyl)-1-methyl-2-propyl-1*H*-indole (3g)

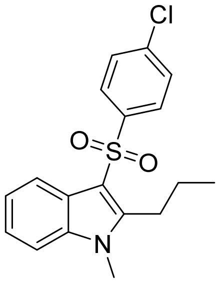

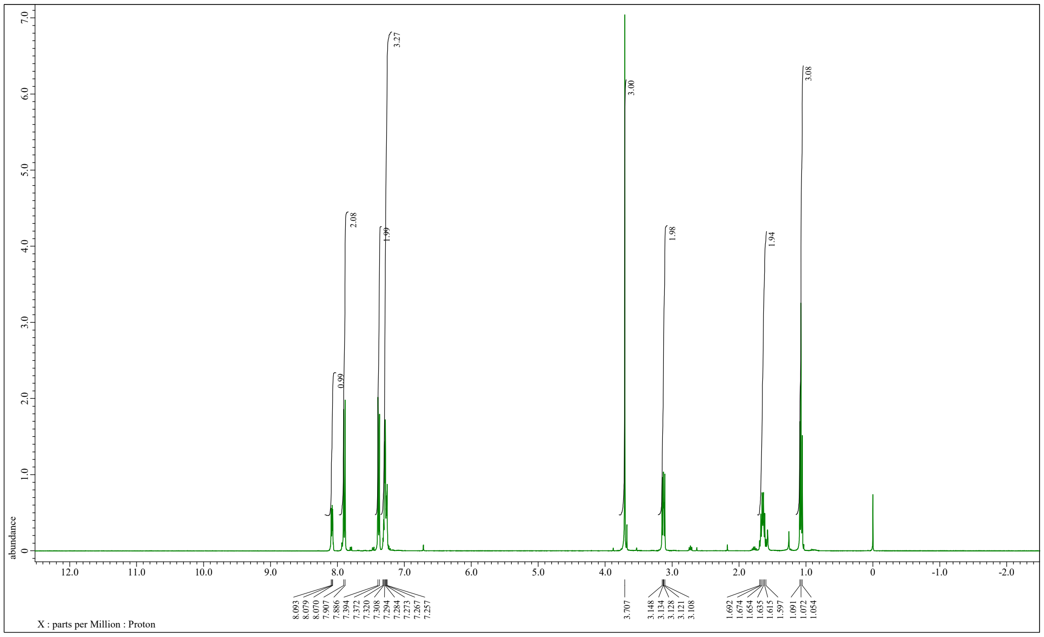

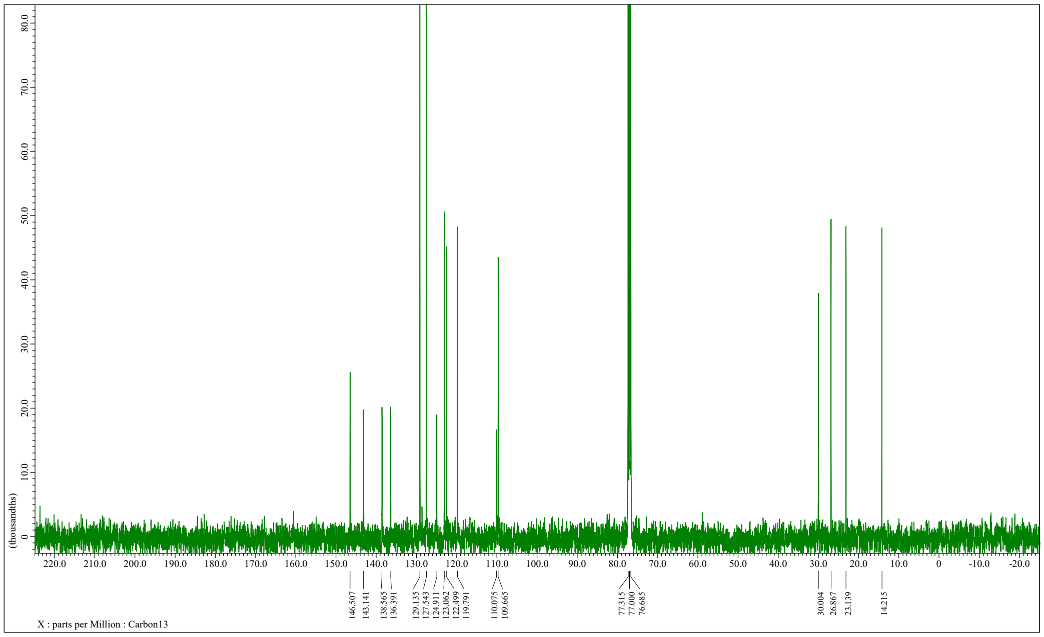

6-((4-Chlorophenyl)sulfonyl)-1-methyl-2-propyl-1H-indole (4g)

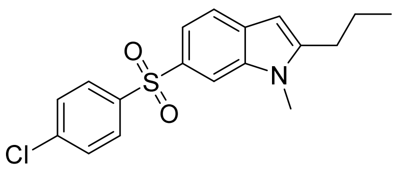

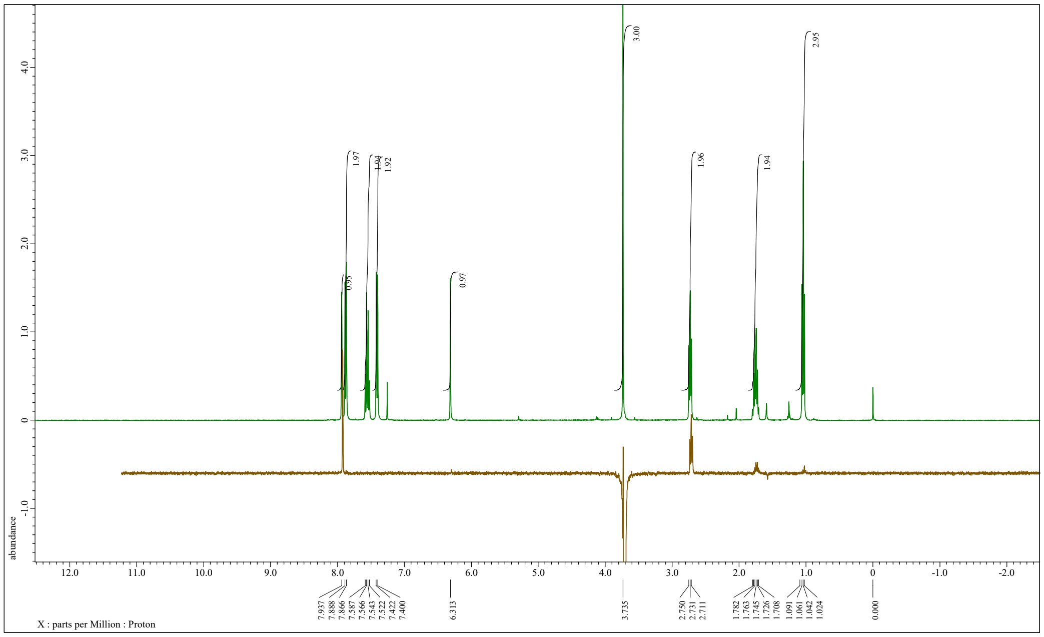

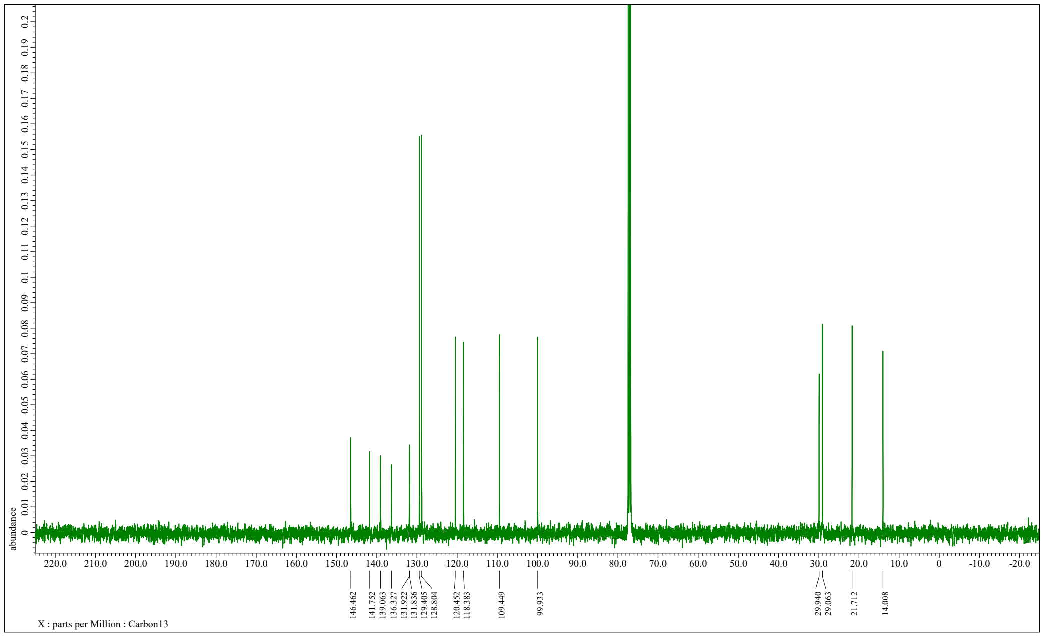

1-Methyl-4-((4-nitrophenyl)sulfonyl)-2-propyl-1*H*-indole (2h)

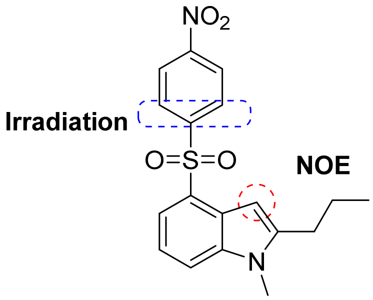

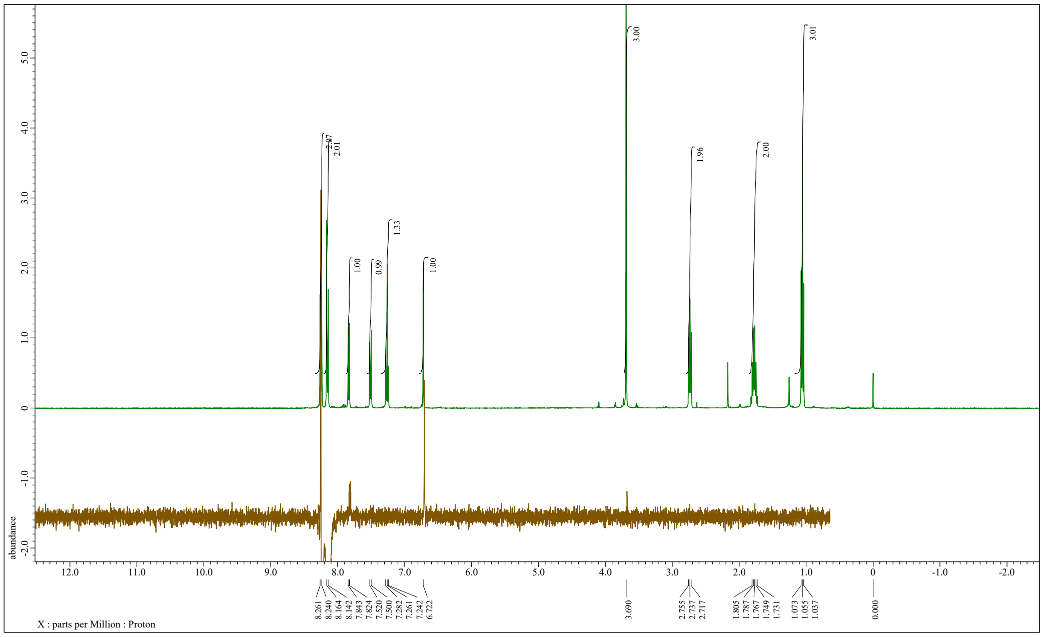

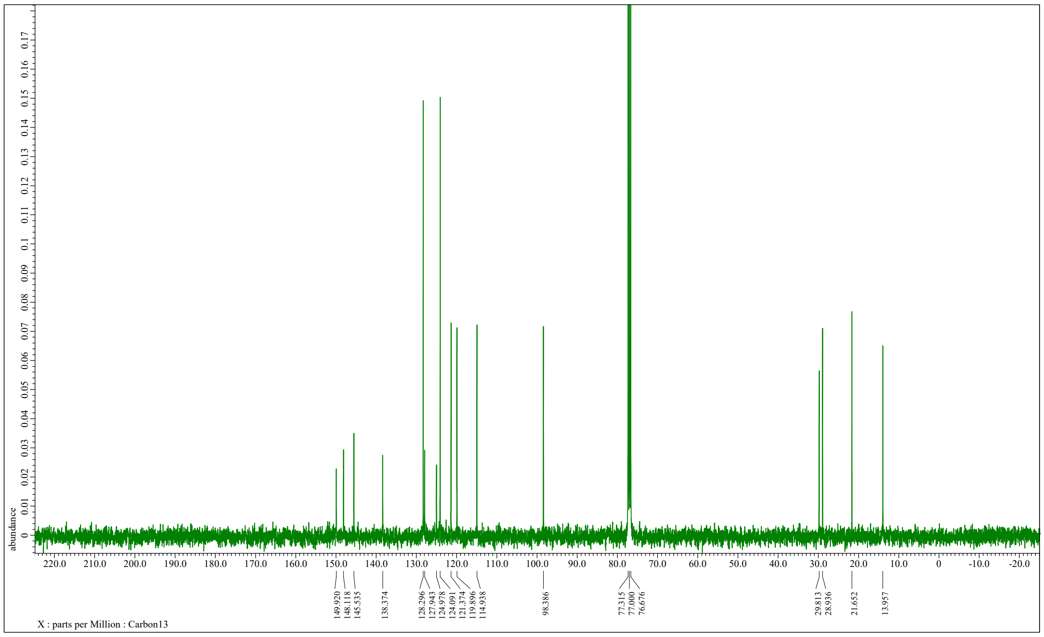

1-Methyl-3-((4-nitrophenyl)sulfonyl)-2-propyl-1*H*-indole (3h)

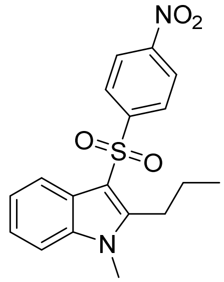

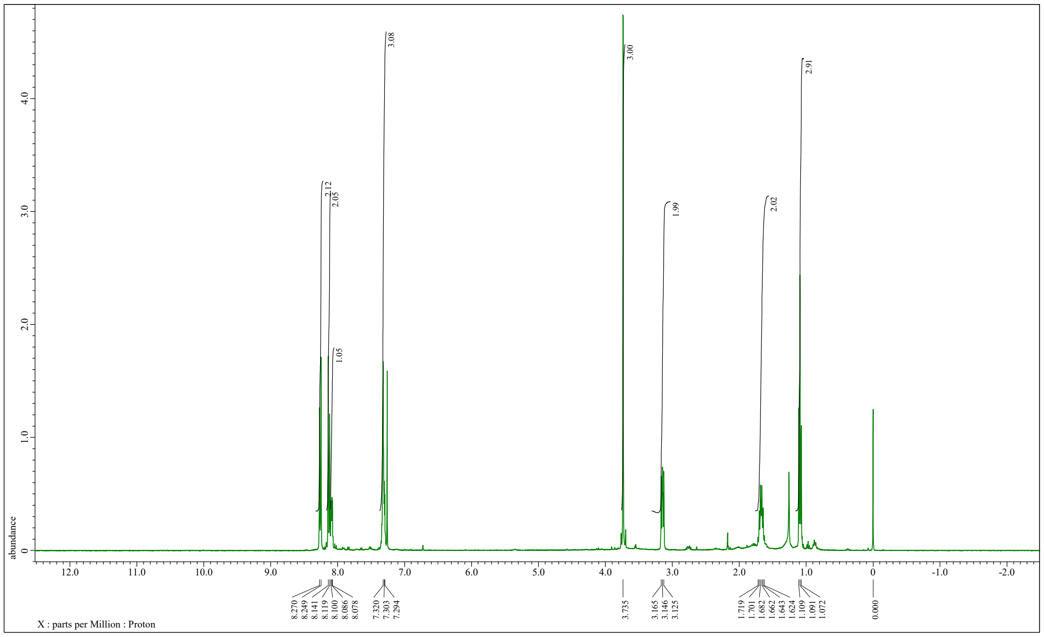

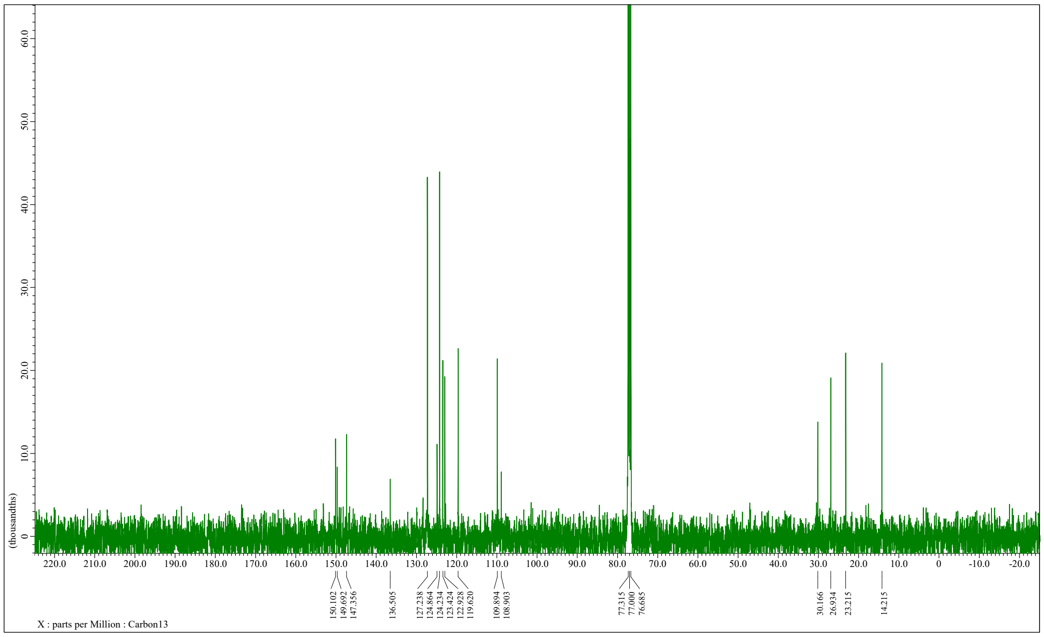

1-Methyl-6-((4-nitrophenyl)sulfonyl)-2-propyl-1*H*-indole (4h)

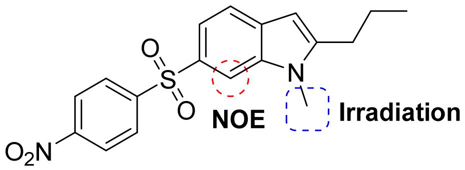

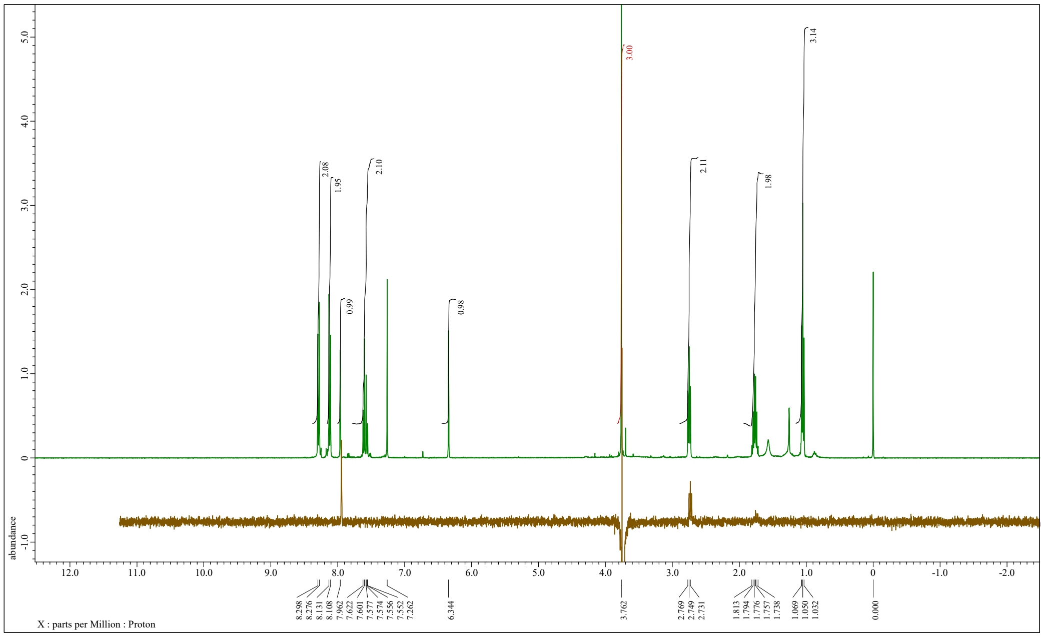

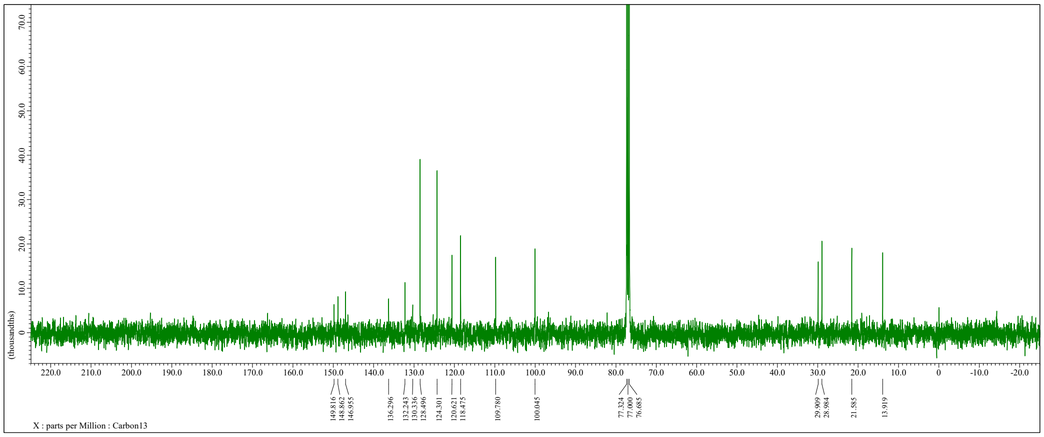

1-Methyl-4-(methylsulfonyl)-2-propyl-1*H*-indole (2i)

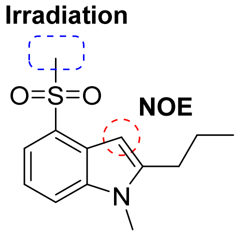

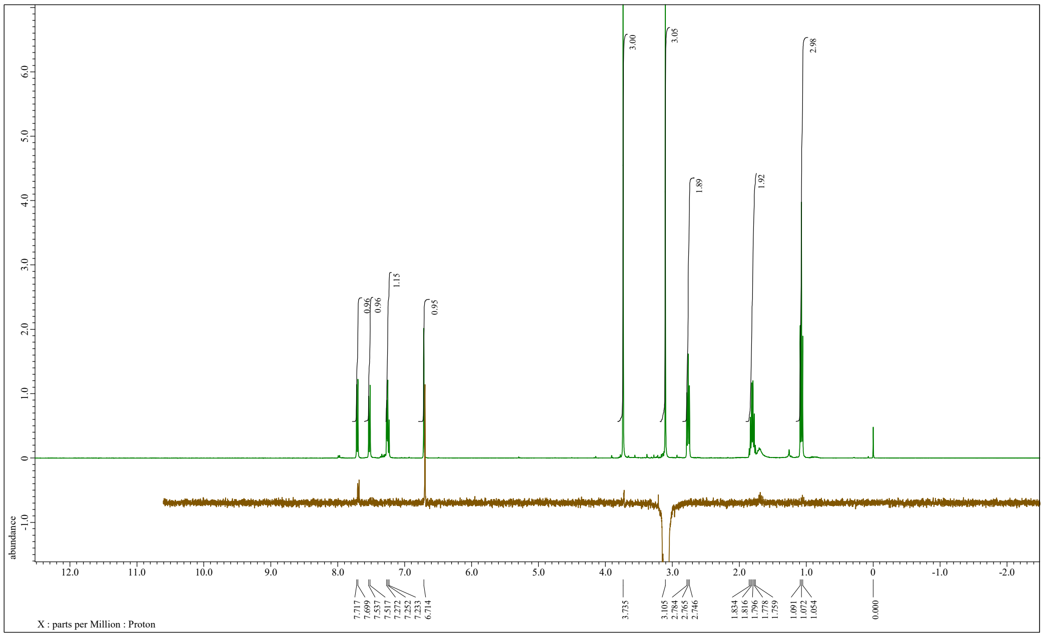

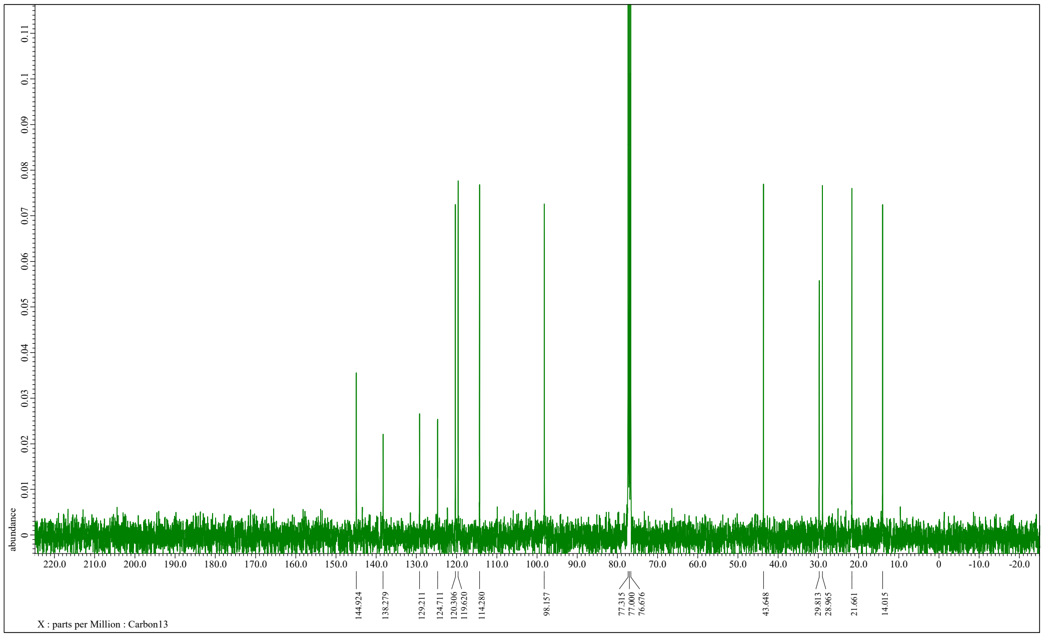

1-Methyl-3-(methylsulfonyl)-2-propyl-1*H*-indole (3i)

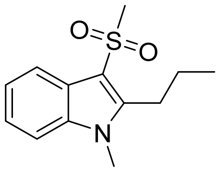

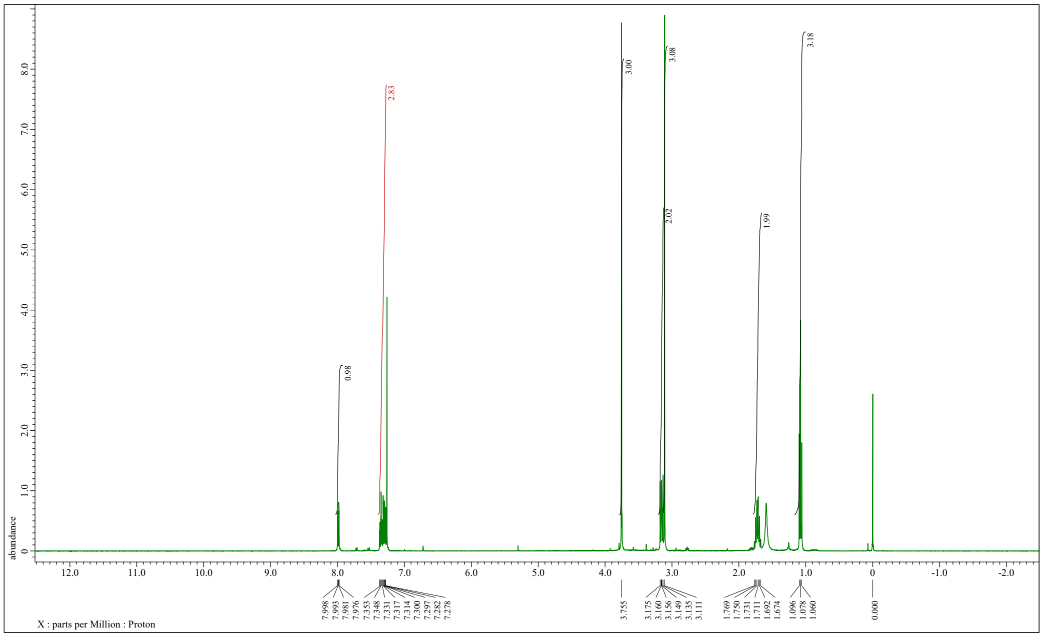

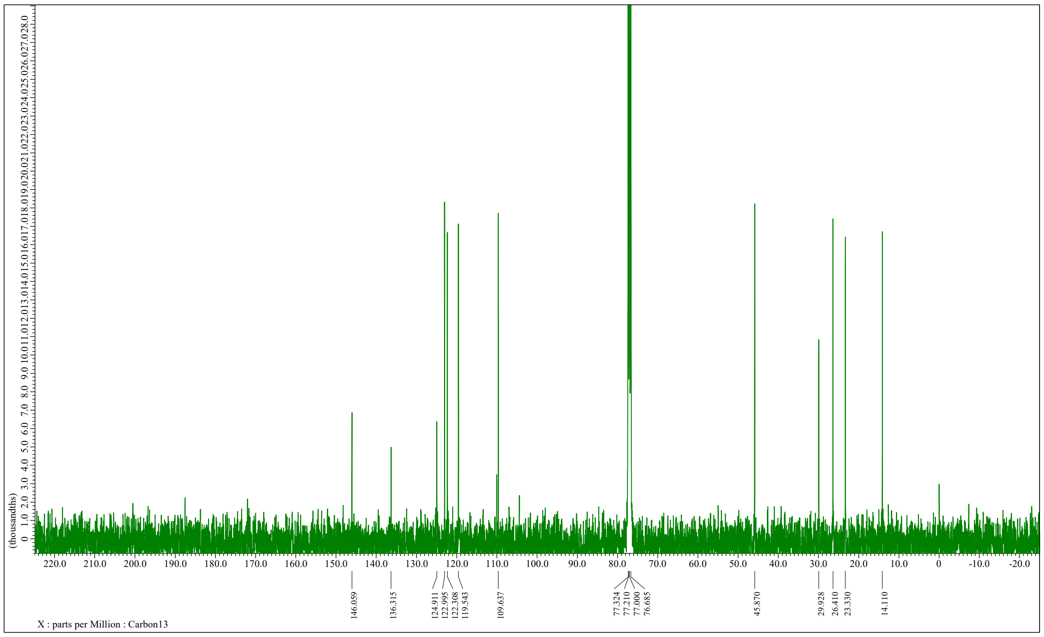

1-Methyl-6-(methylsulfonyl)-2-propyl-1*H*-indole (4i)

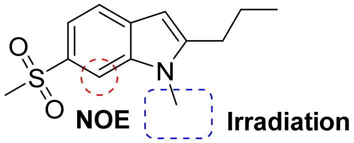

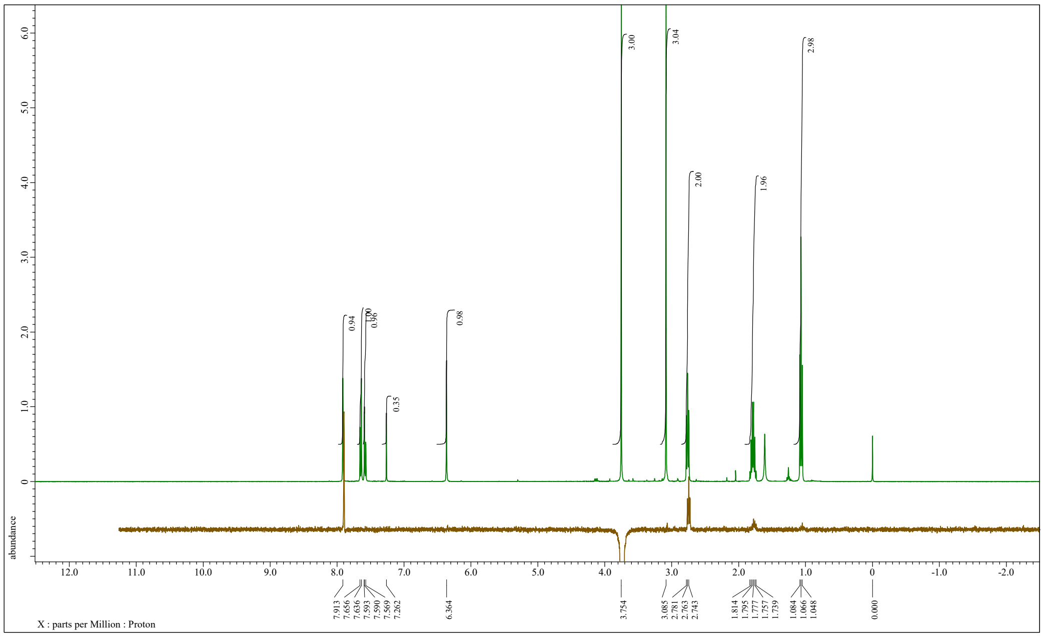

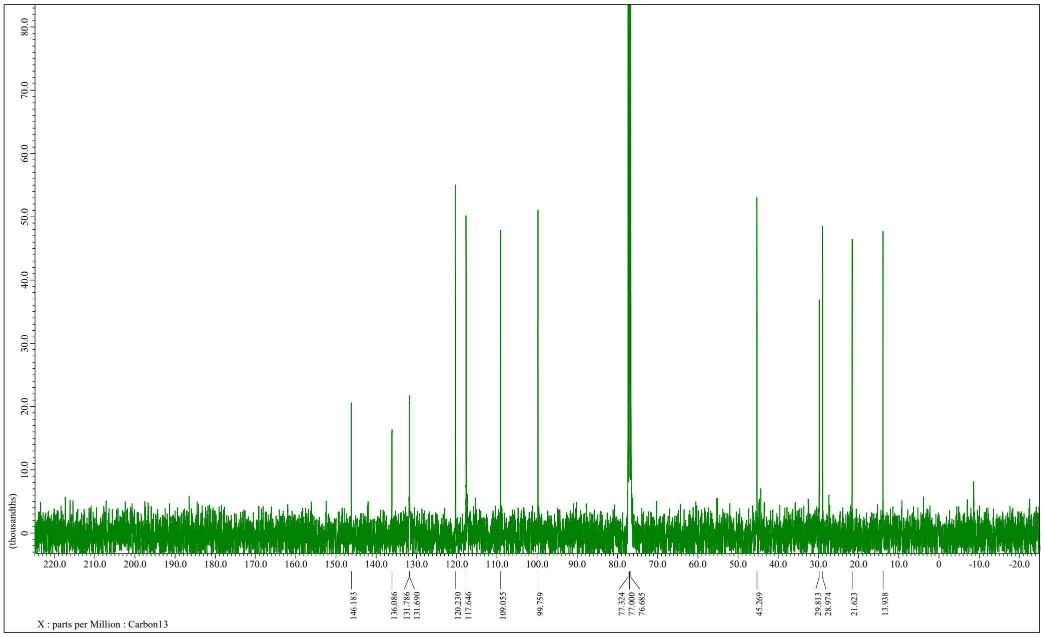

4-((4-Fluorophenyl)sulfonyl)-2-hexyl-1-methyl-1*H*-indole (2j)

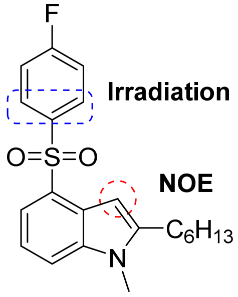

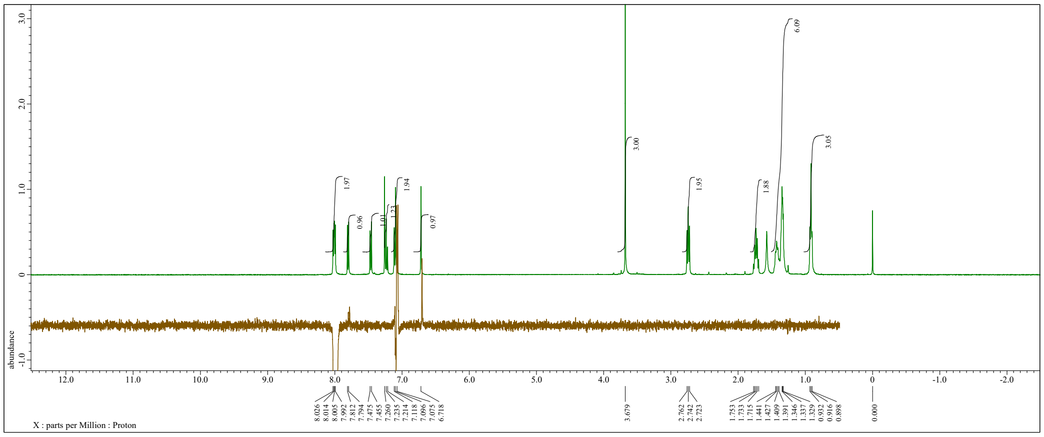

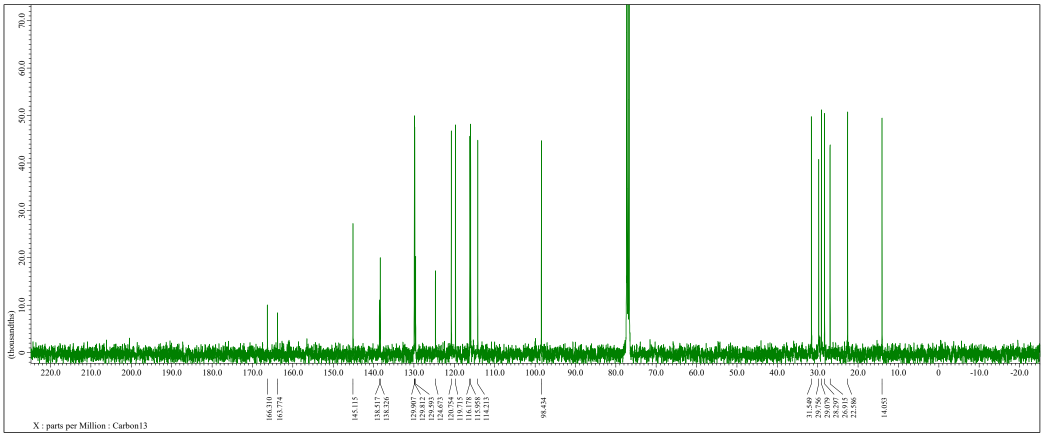

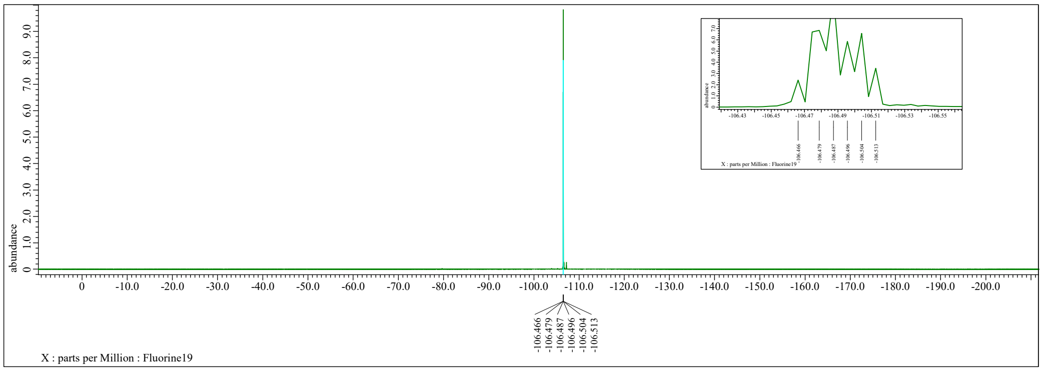

3-((4-Fluorophenyl)sulfonyl)-2-hexyl-1-methyl-1*H*-indole (3j)

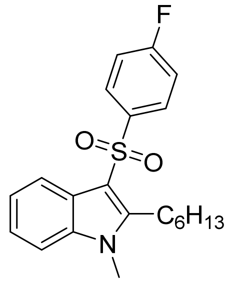

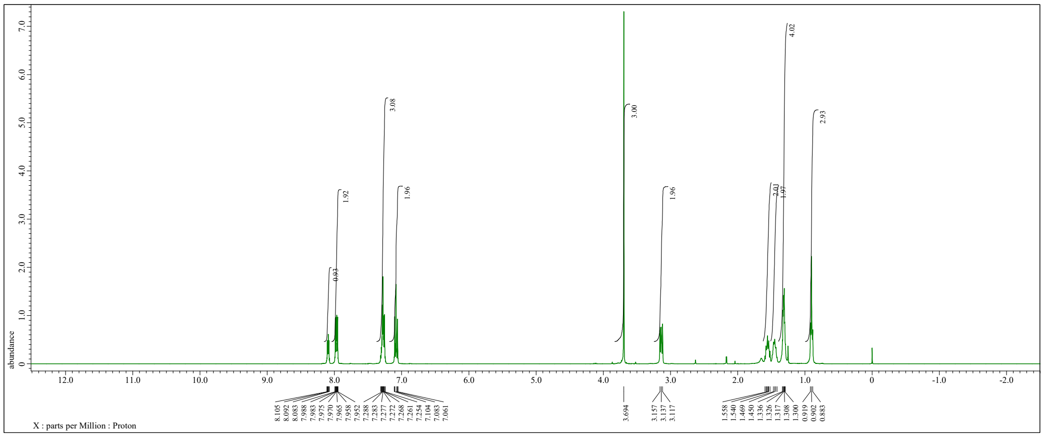

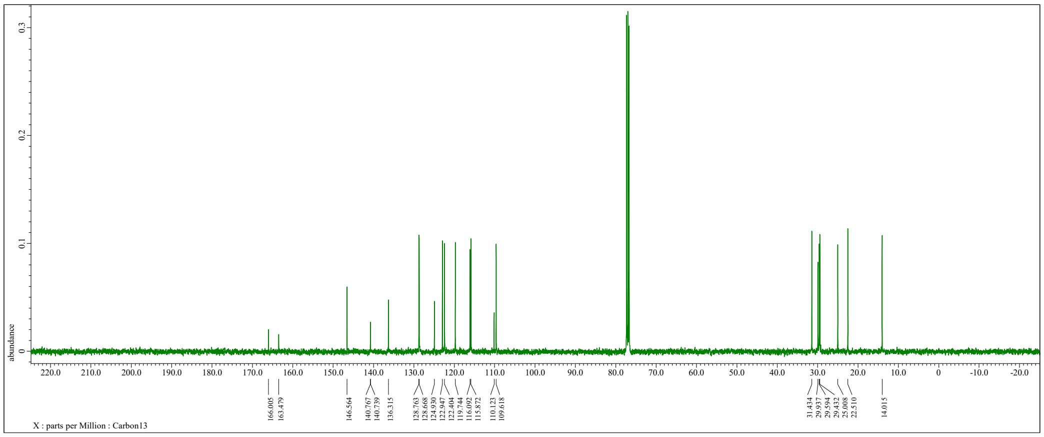

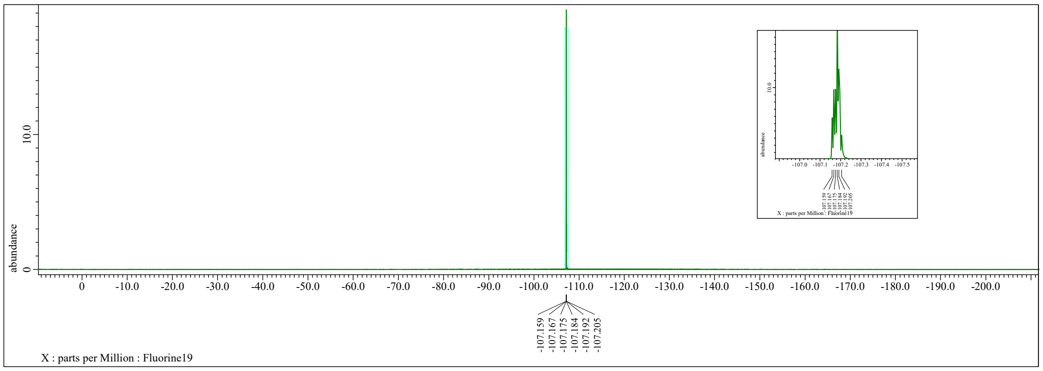

6-((4-fluorophenyl)sulfonyl)-2-hexyl-1-methyl-1*H*-indole (4j)

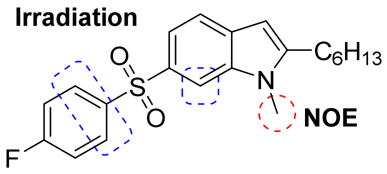

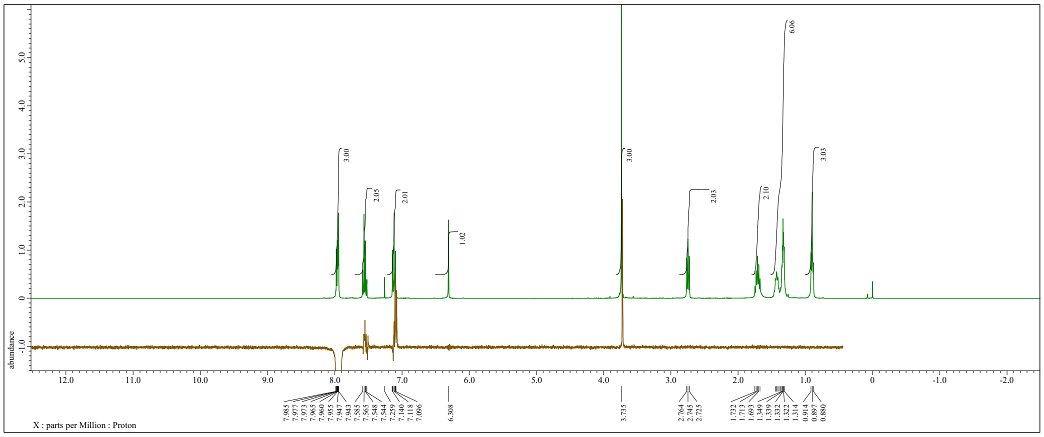

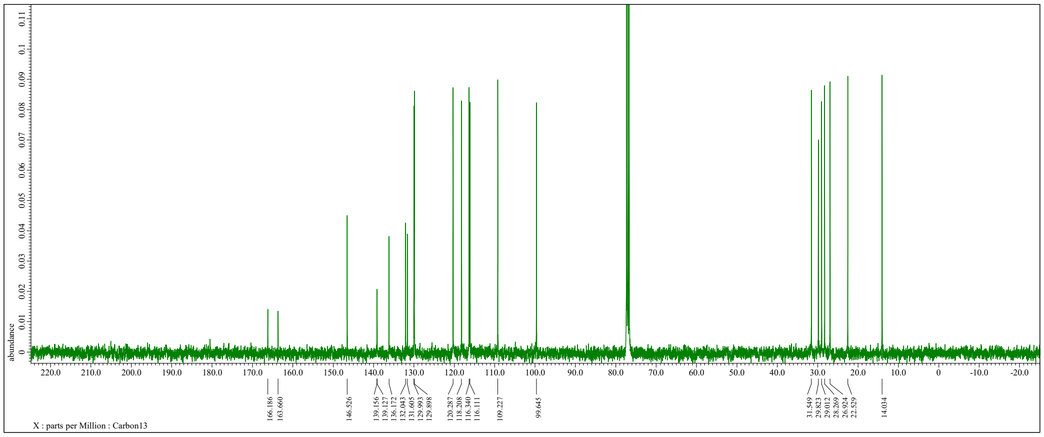

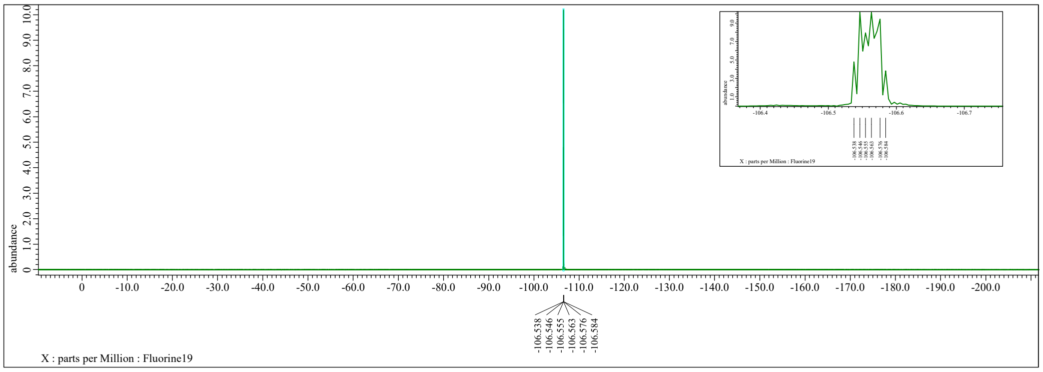

4-((4-Fluorophenyl)sulfonyl)-2-cyclohexyl-1-methyl-1*H*-indole (2k)

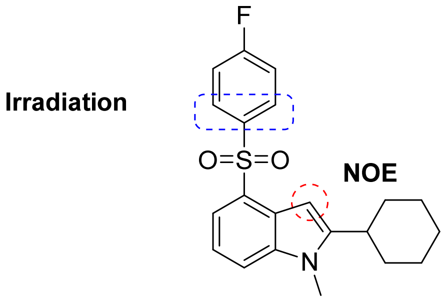

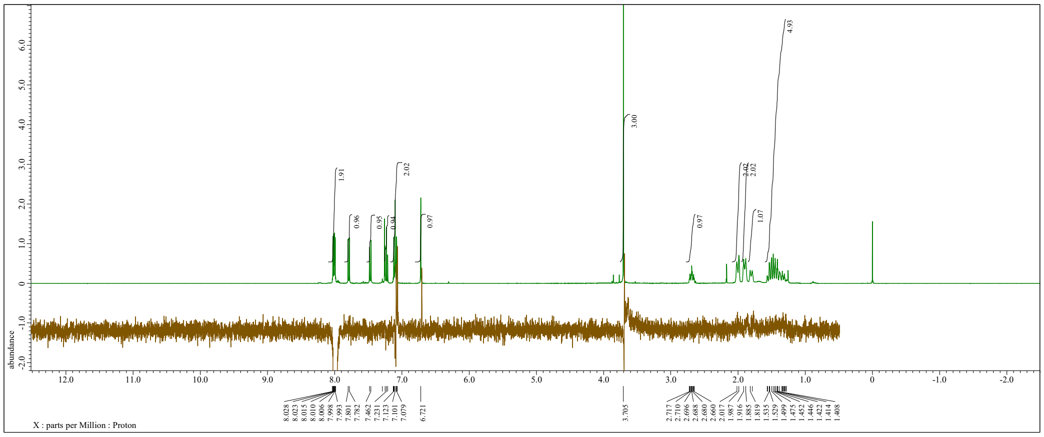

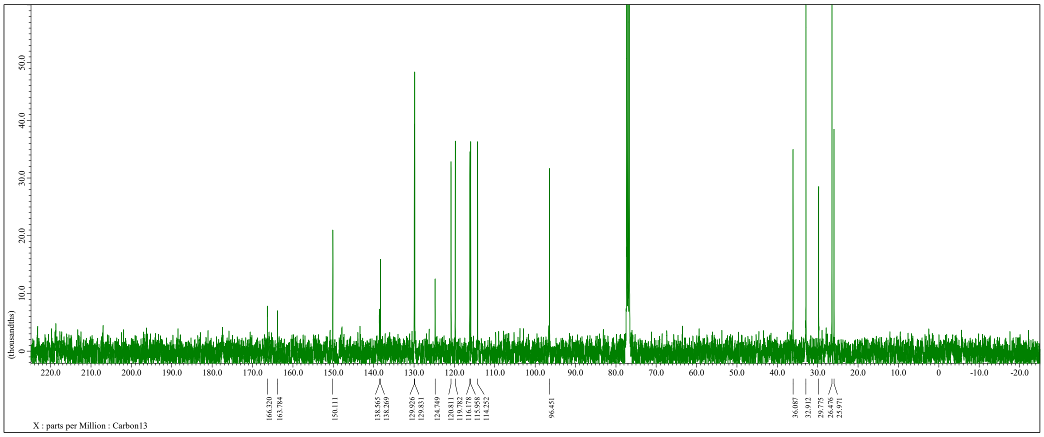

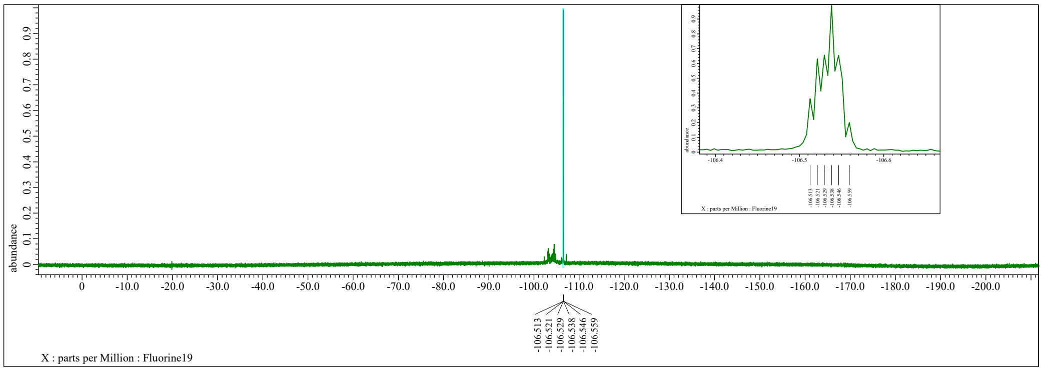

3-((4-Fluorophenyl)sulfonyl)-2-cyclohexyl-1-methyl-1*H*-indole (3k)

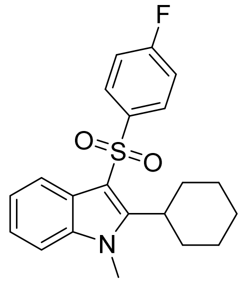

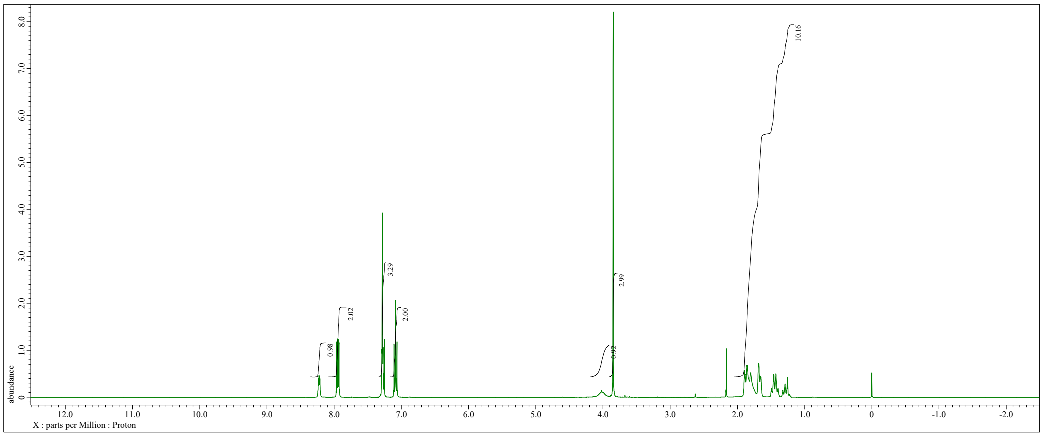

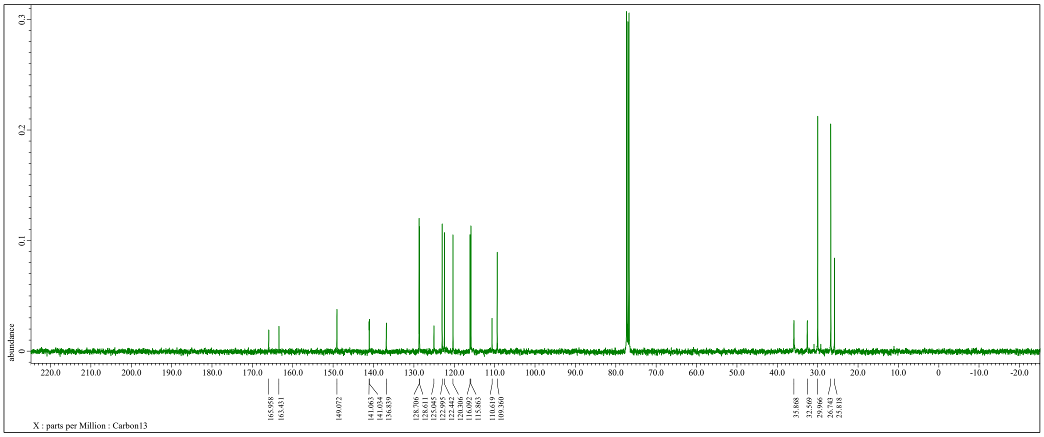

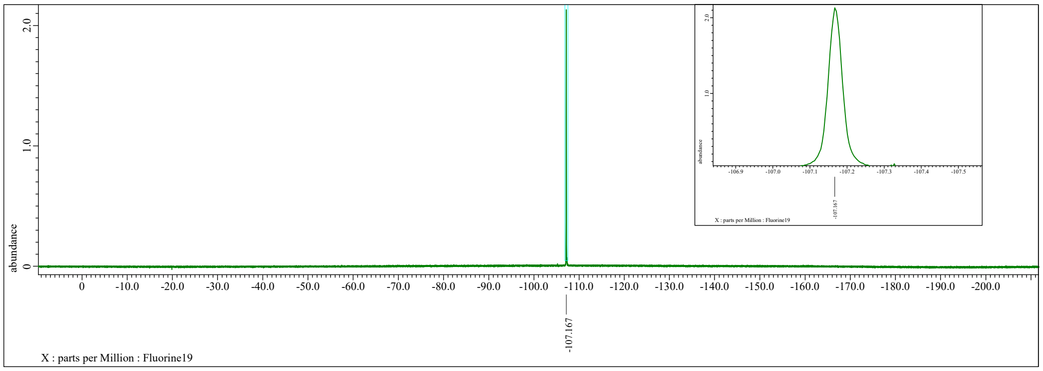

6-((4-Fluorophenyl)sulfonyl)-2-cyclohexyl-1-methyl-1*H*-indole (4k)

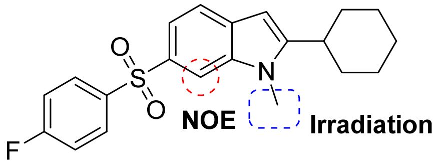

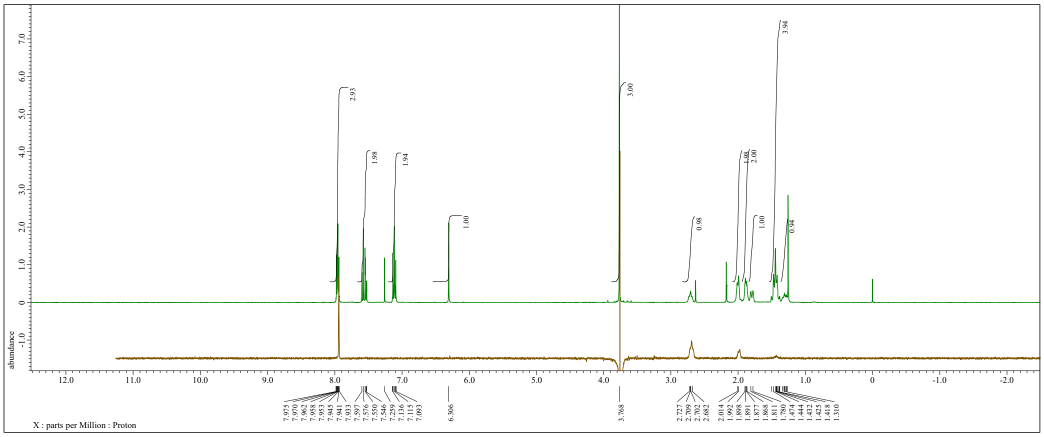

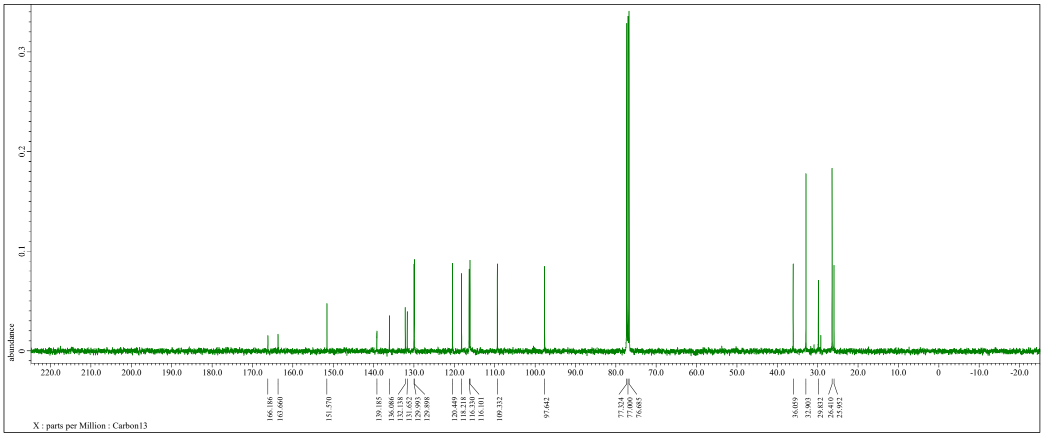

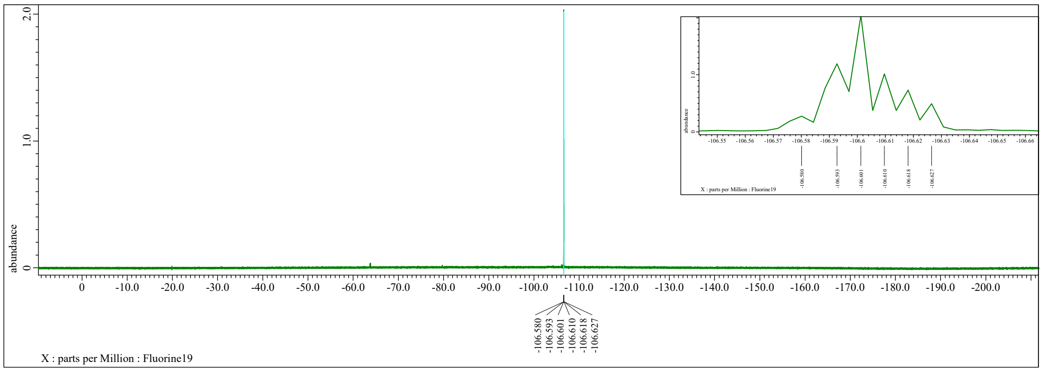

4-((4-Fluorophenyl)sulfonyl)-2-cyclopropyl-1-methyl-1*H*-indole (2l)

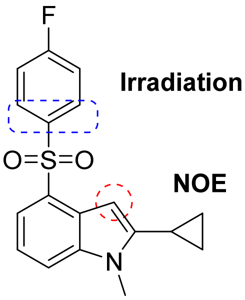

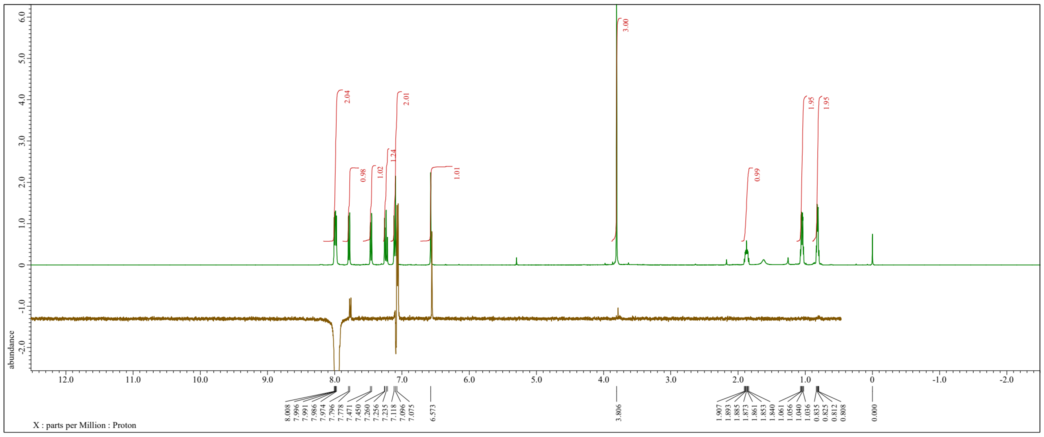

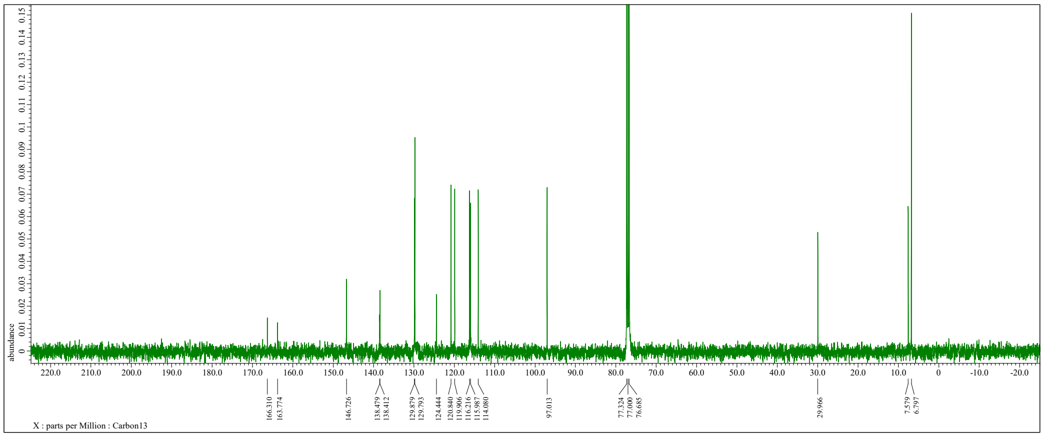

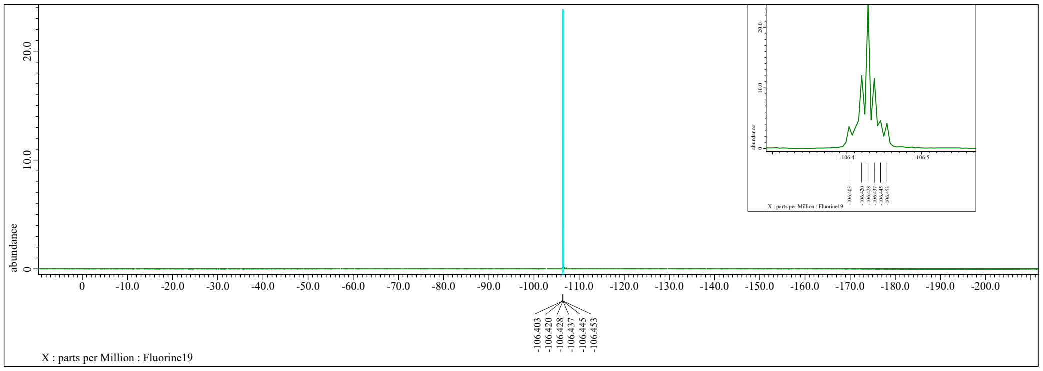

3-((4-Fluorophenyl)sulfonyl)-2-cyclopropyl-1-methyl-1*H*-indole (3l)

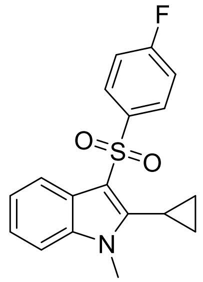

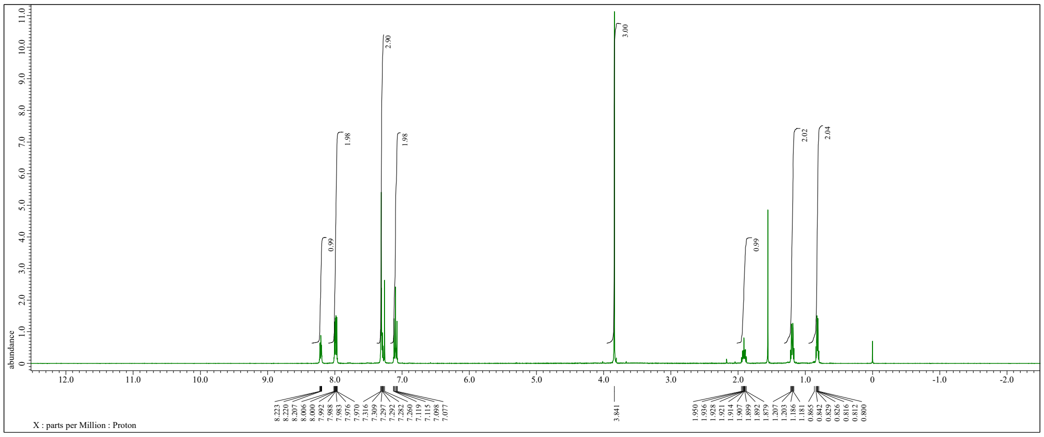

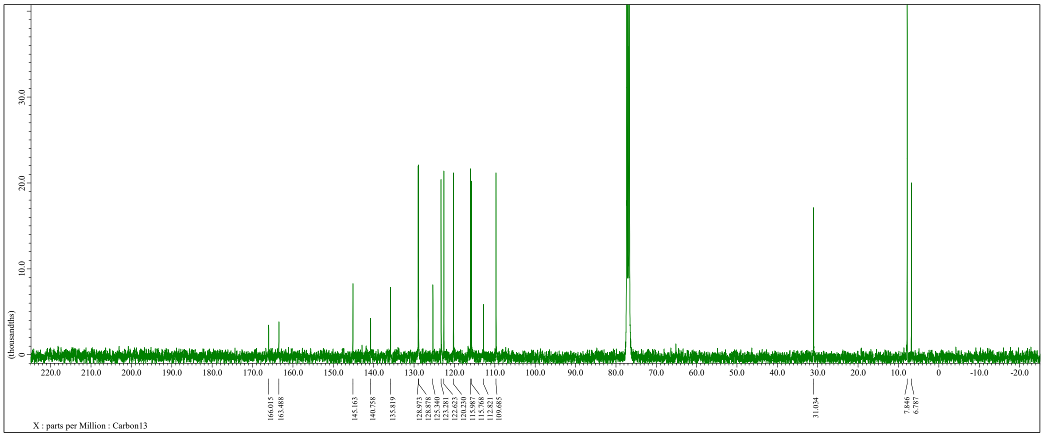

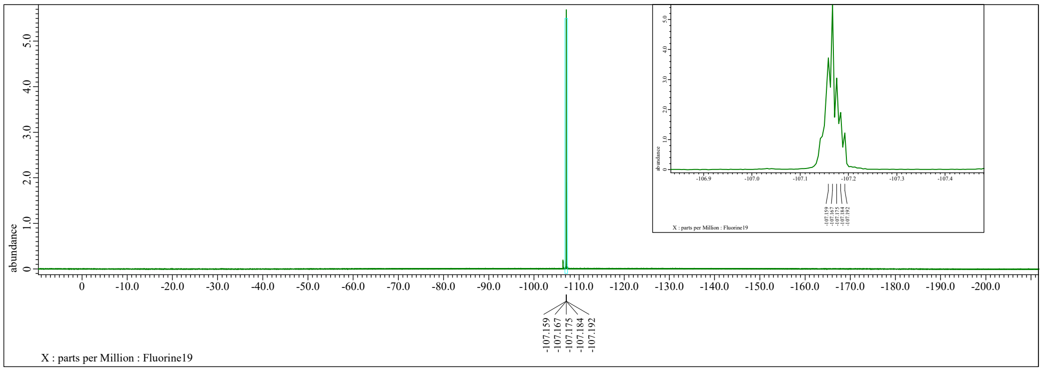

6-((4-Fluorophenyl)sulfonyl)-2-cyclopropyl-1-methyl-1*H*-indole (4l)

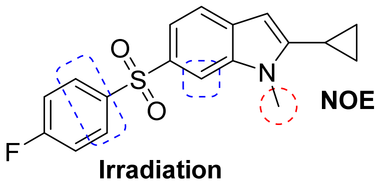

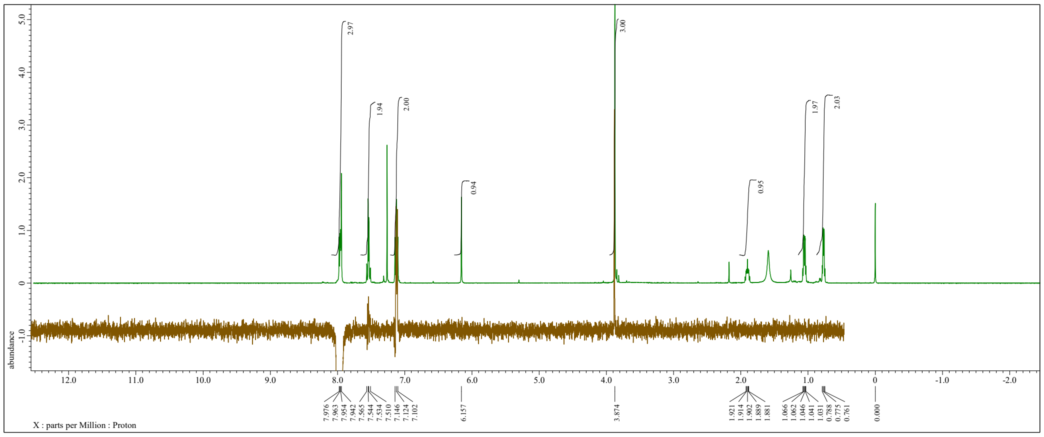

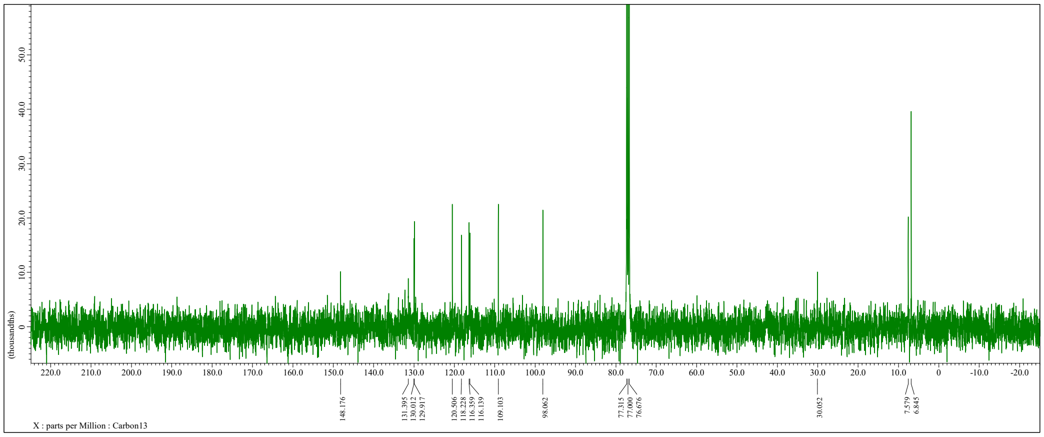

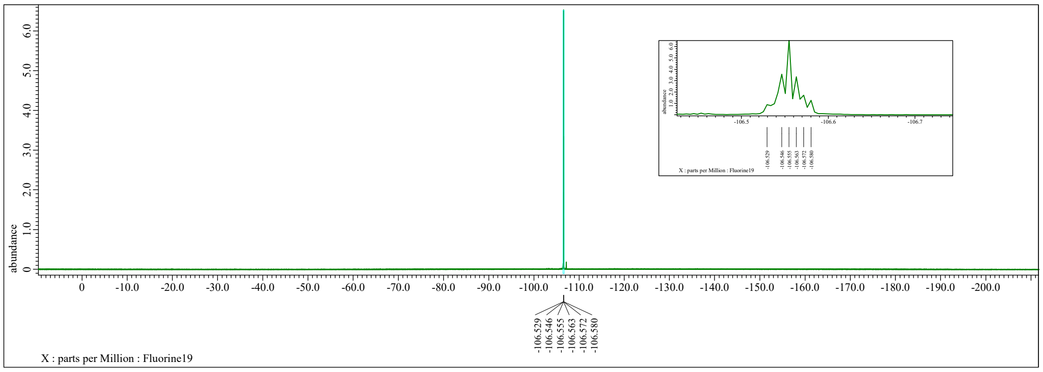

4-((4-Fluorophenyl)sulfonyl)-1,2-dimethyl-1*H*-indole (2m)

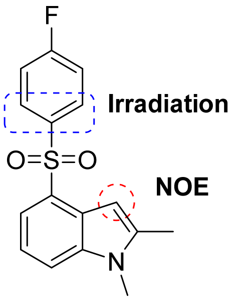

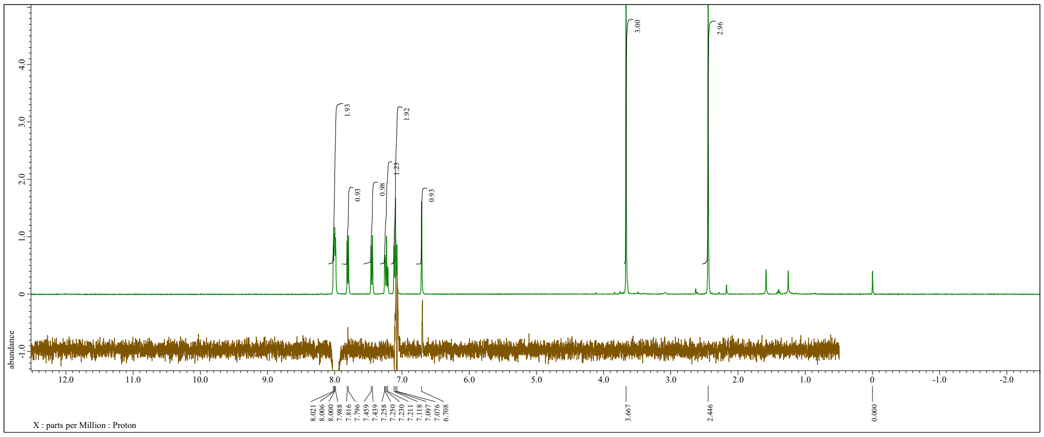

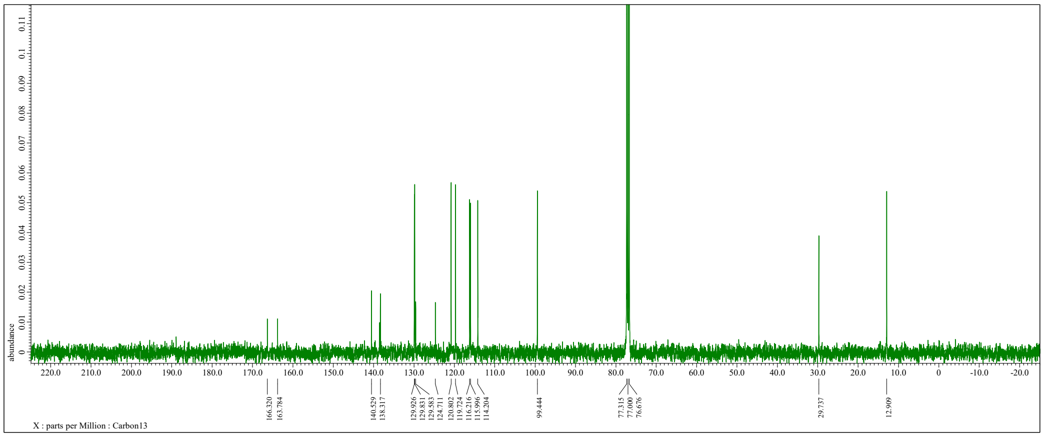

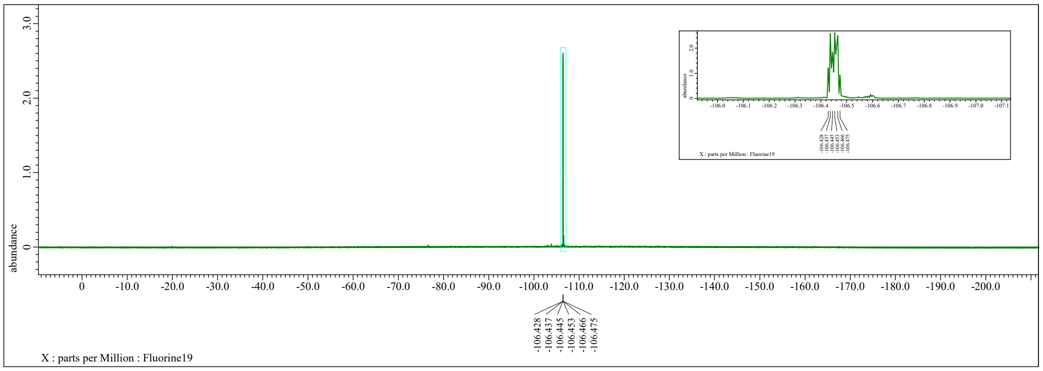

3-((4-Fluorophenyl)sulfonyl)-1,2-dimethyl-1*H*-indole (3m)

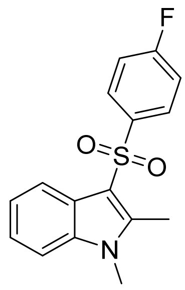

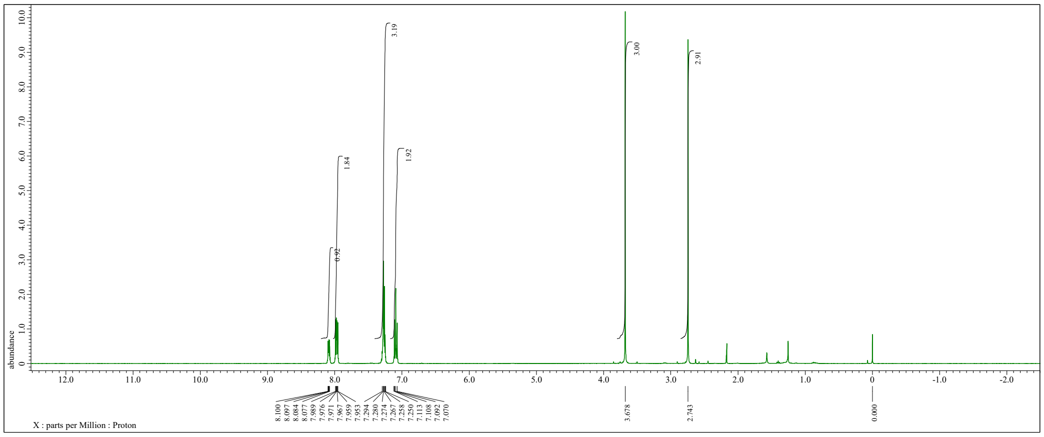

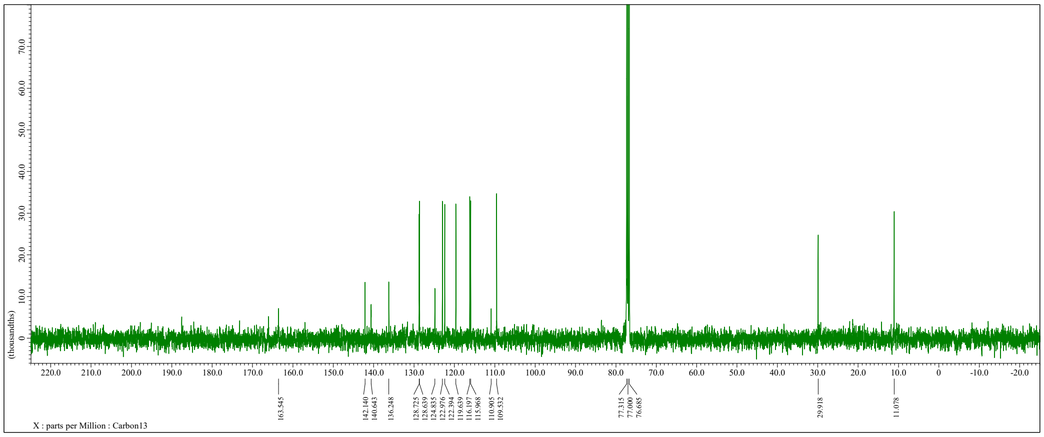

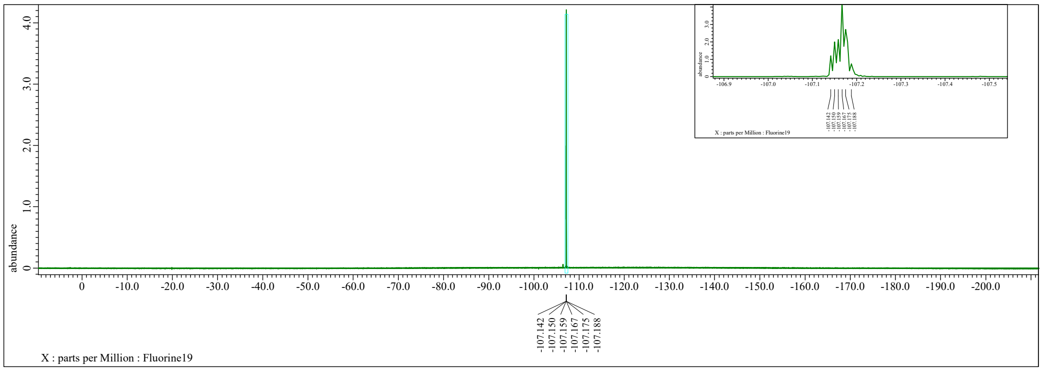

6-((4-Fluorophenyl)sulfonyl)-1,2-dimethyl-1*H*-indole (4m)

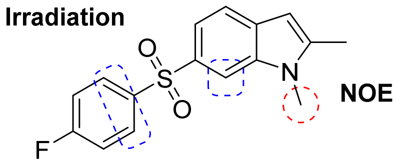

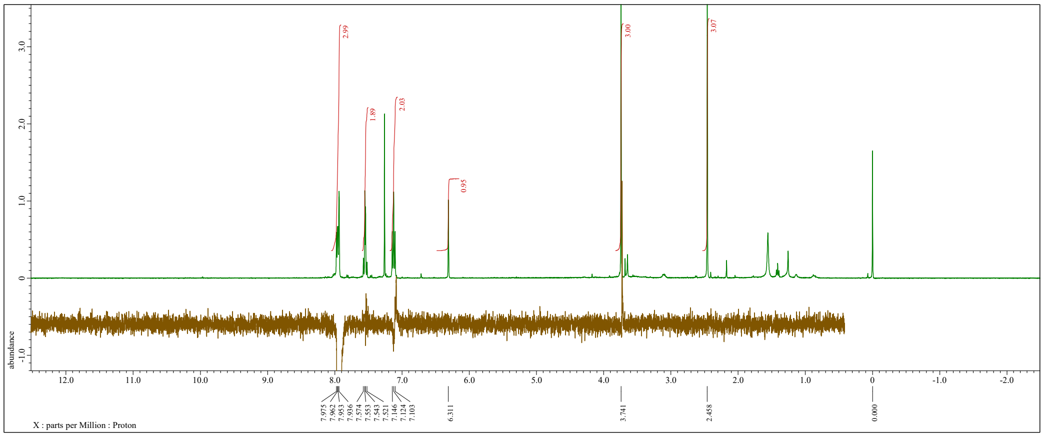

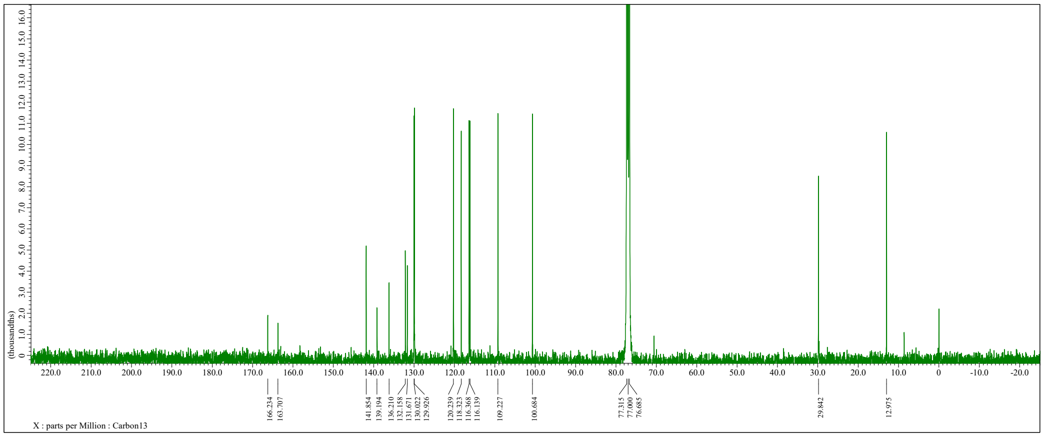

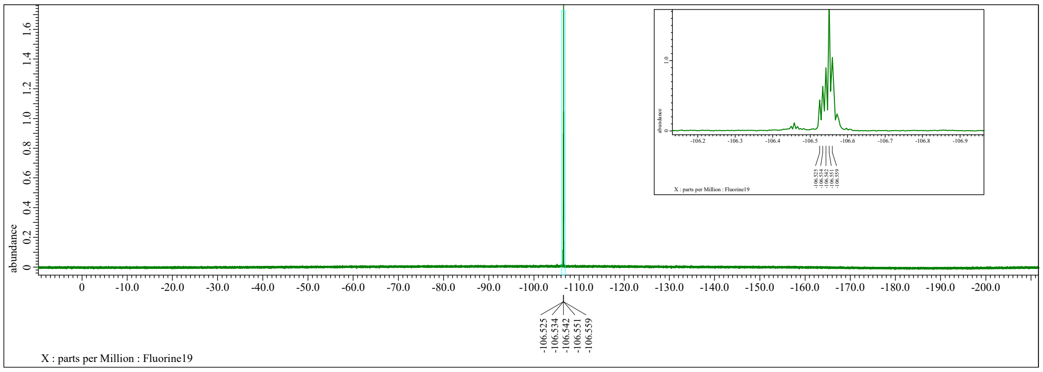

4-((4-Fluorophenyl)sulfonyl)-1-methyl-2-phenyl-1*H*-indole (2n)

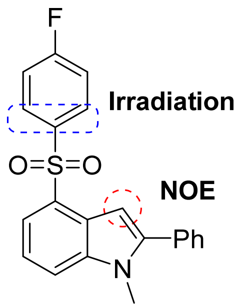

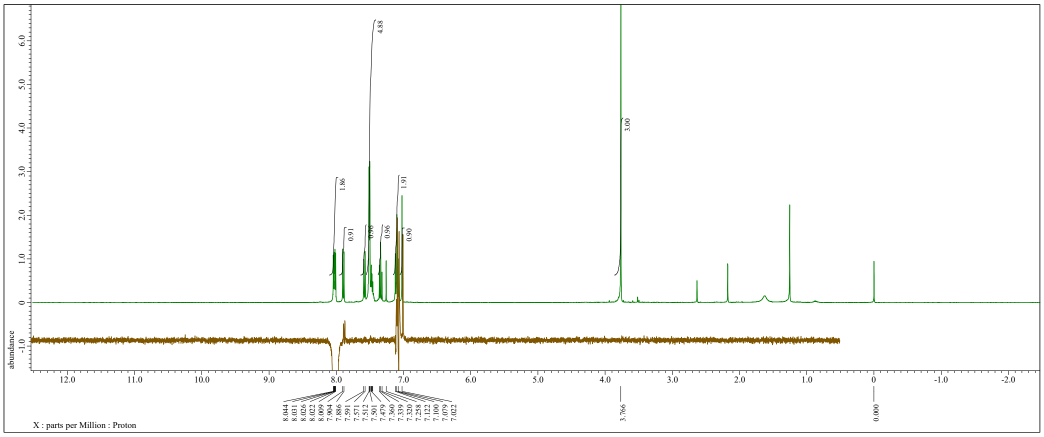

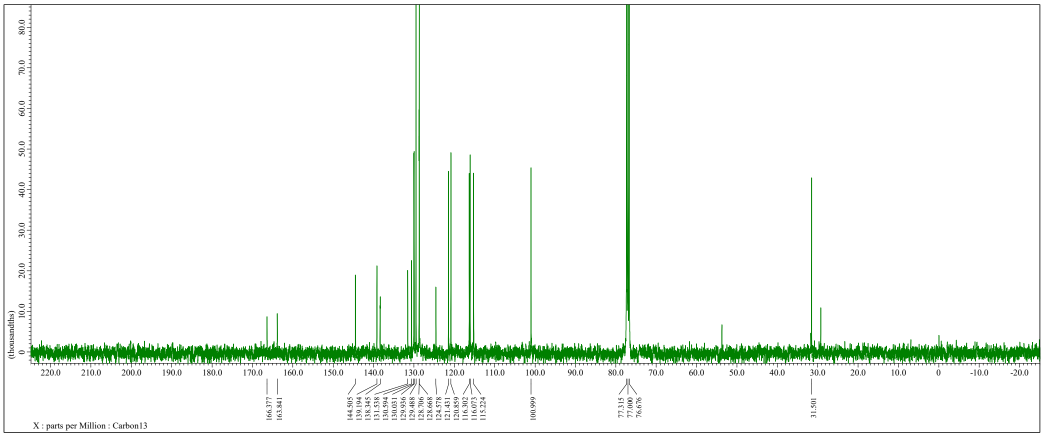

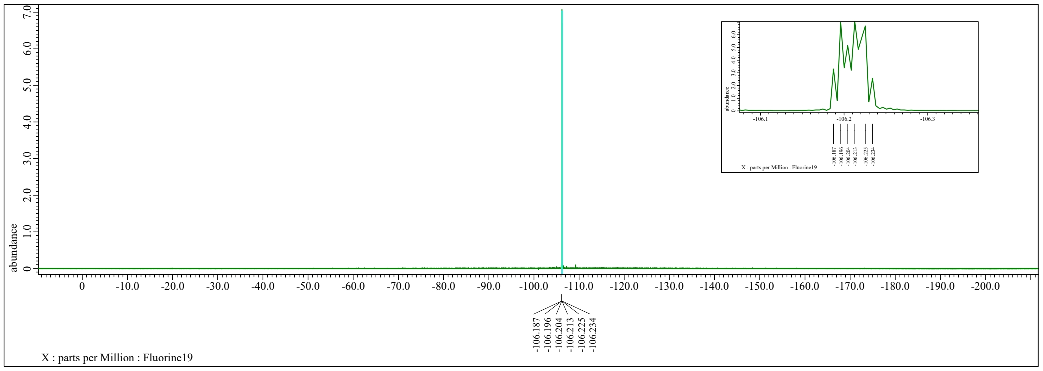

3-((4-Fluorophenyl)sulfonyl)-1-methyl-2-phenyl-1*H*-indole (3n)

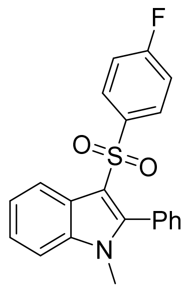

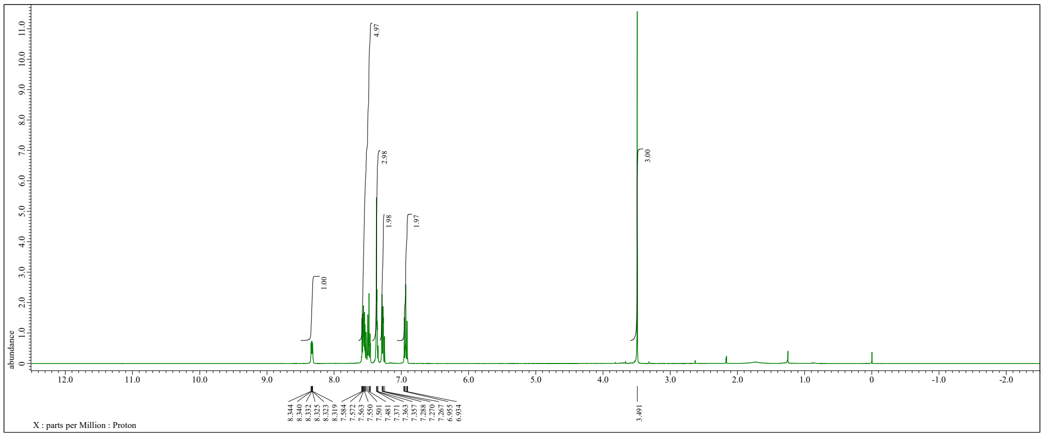

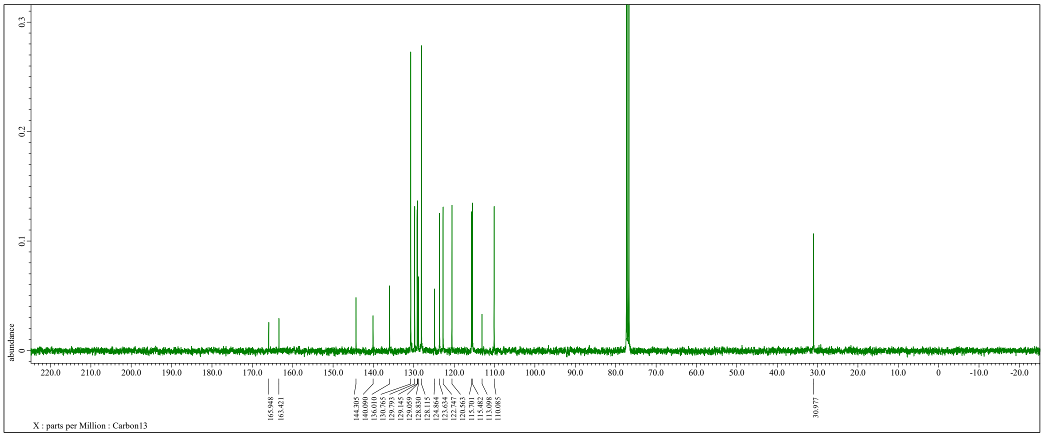

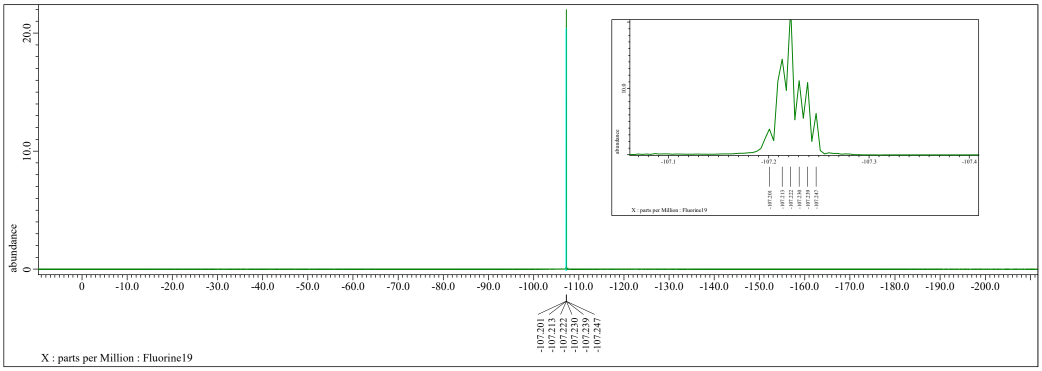

6-((4-Fluorophenyl)sulfonyl)-1-methyl-2-phenyl-1*H*-indole (4n)

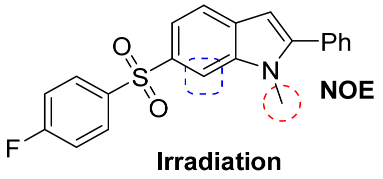

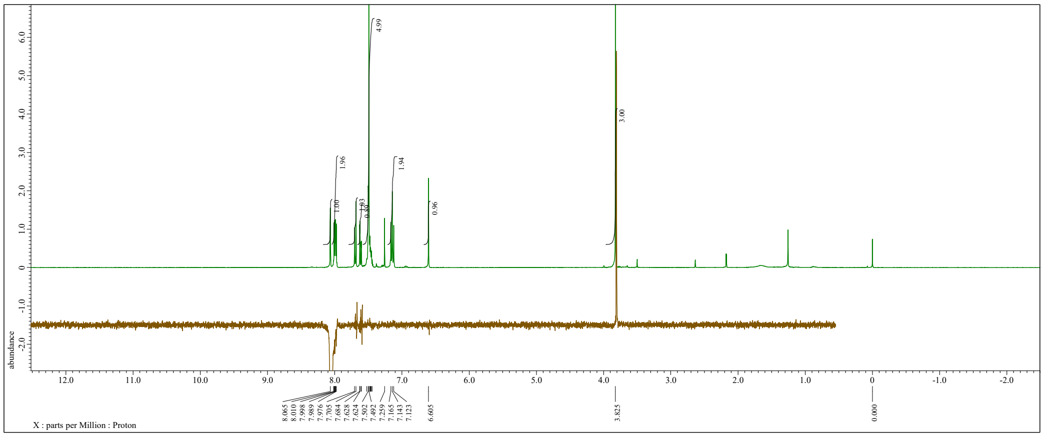

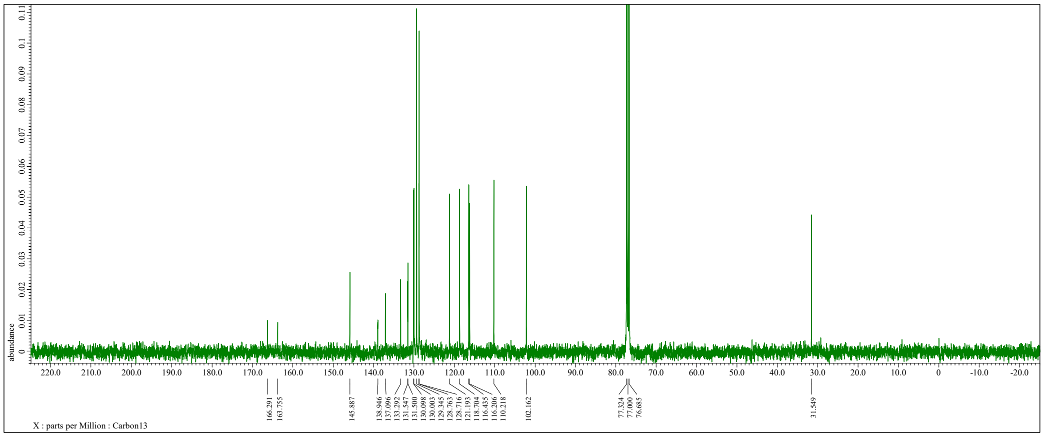

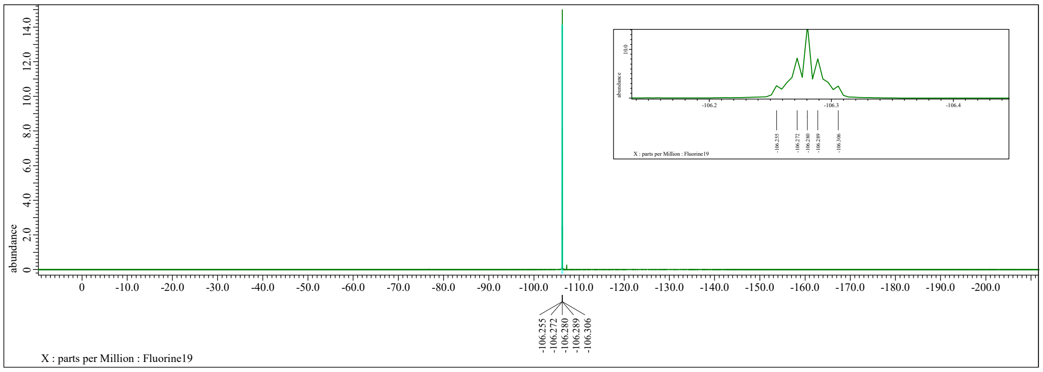

1-benzyl-4-((4-fluorophenyl)sulfonyl)-2-propyl-1*H*-indole (2q)

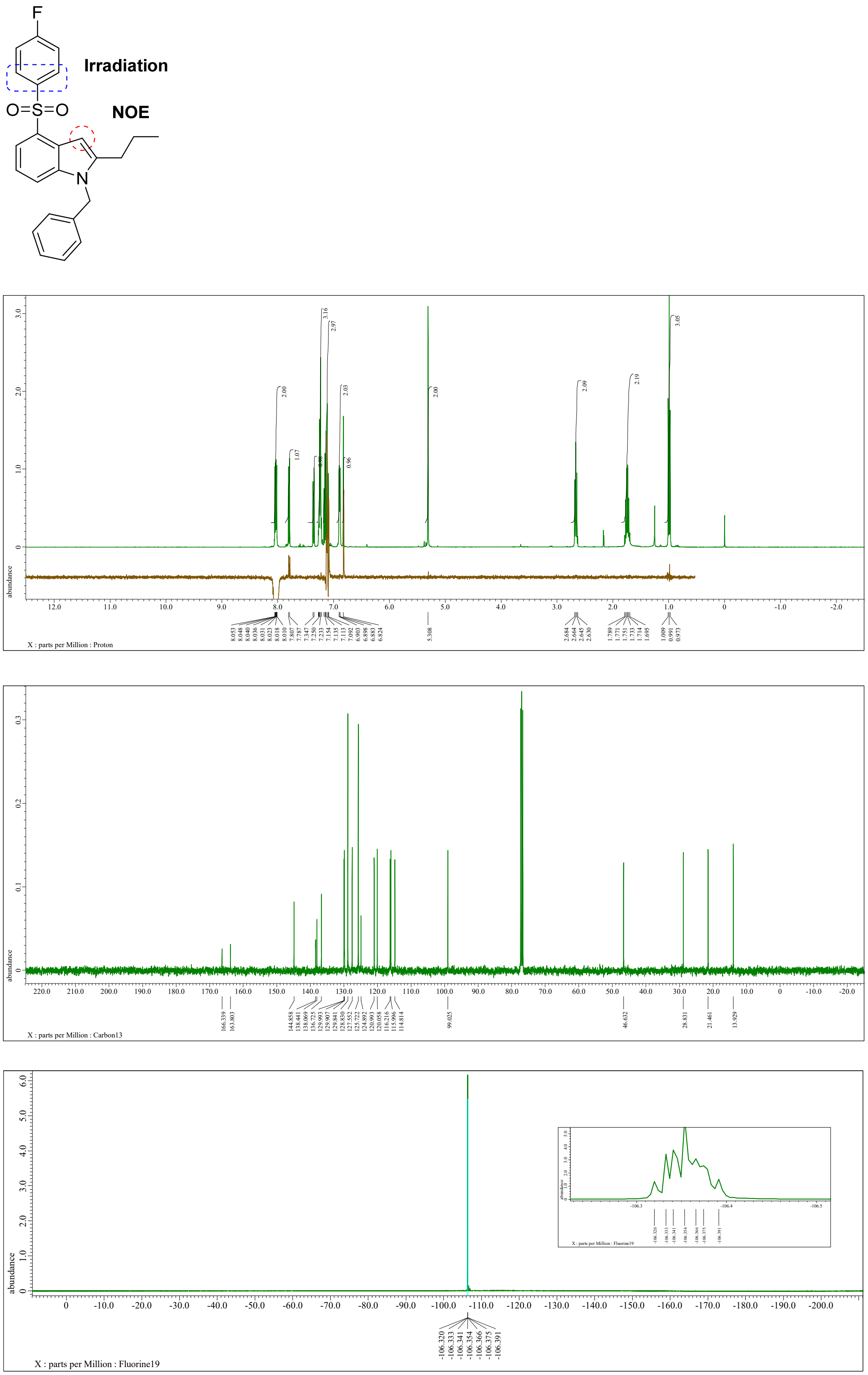

1-benzyl-3-((4-fluorophenyl)sulfonyl)-2-propyl-1*H*-indole (3q)

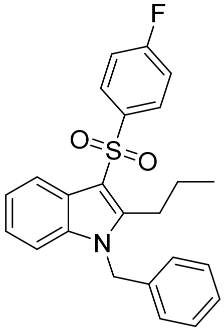

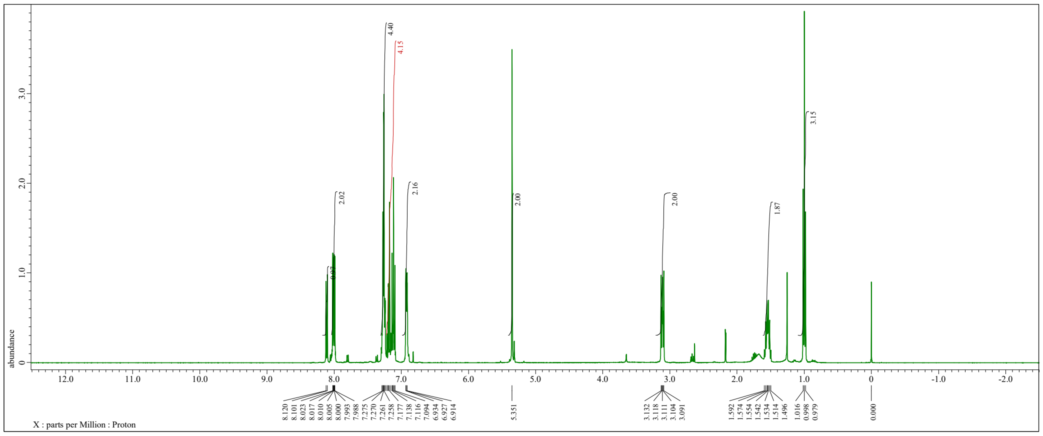

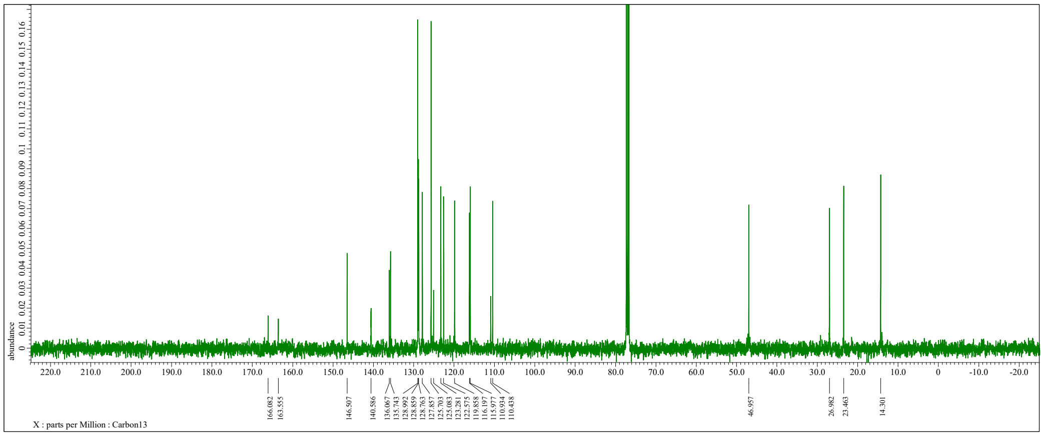

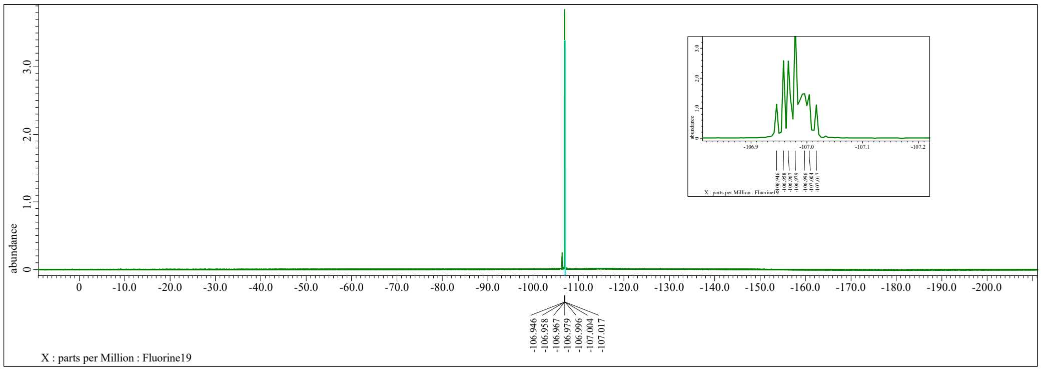

1-benzyl-6-((4-fluorophenyl)sulfonyl)-2-propyl-1*H*-indole (4q)

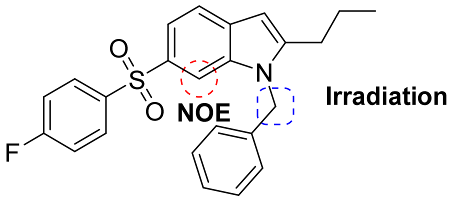

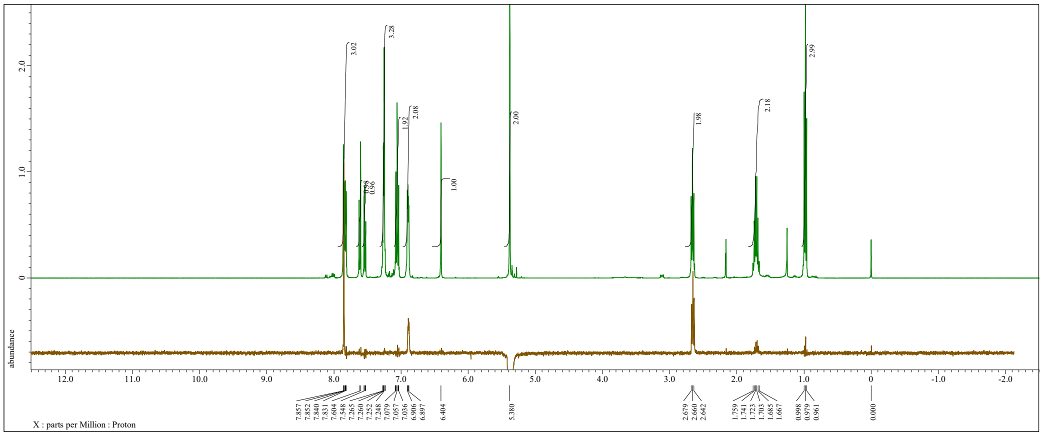

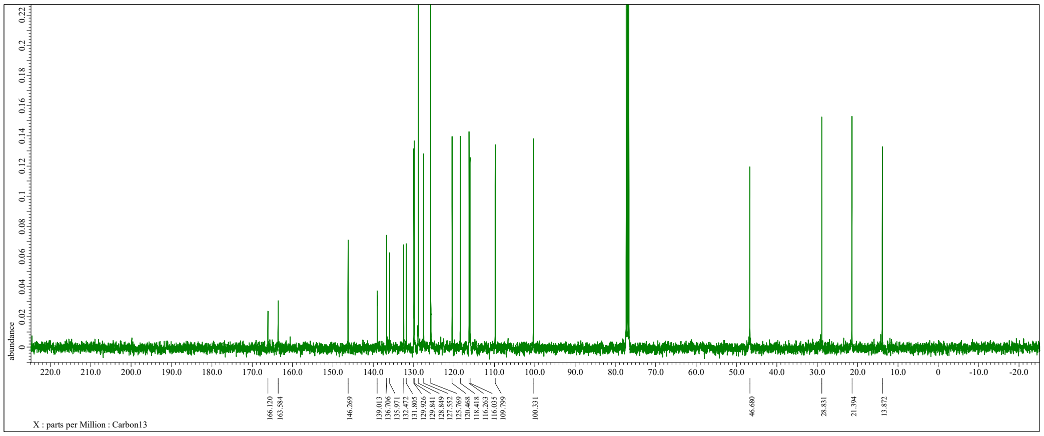

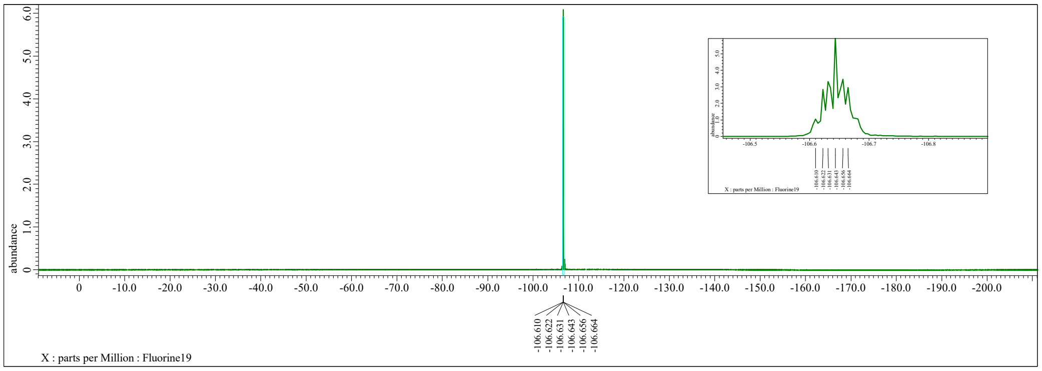

1-methyl-2-phenyl-4-tosyl-1*H*-indole (2r)

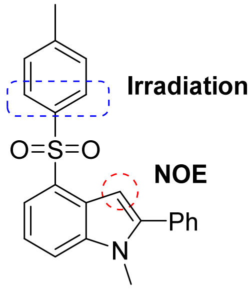

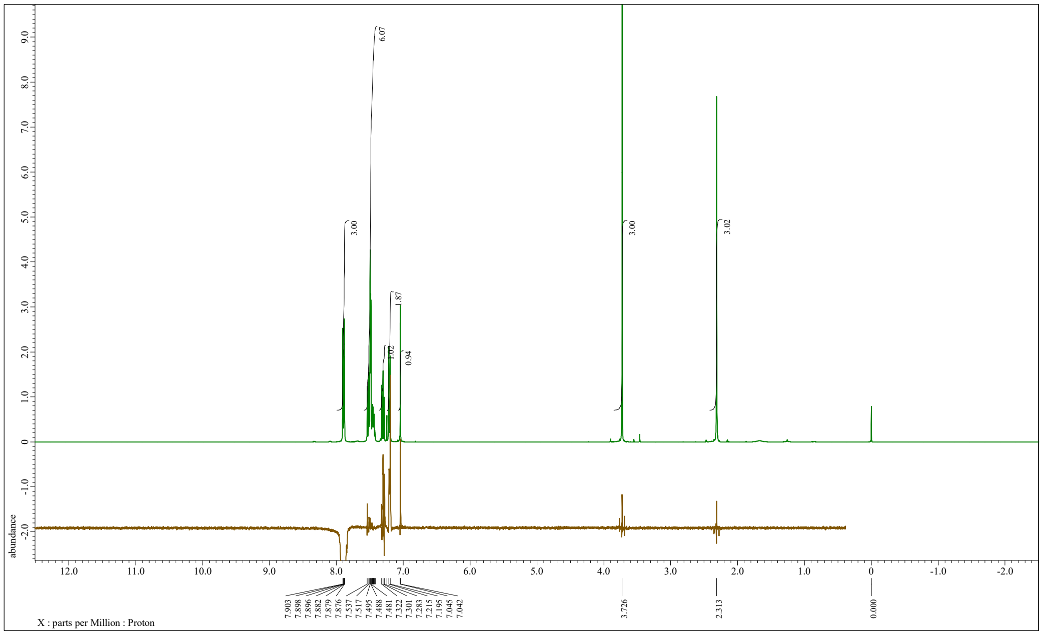

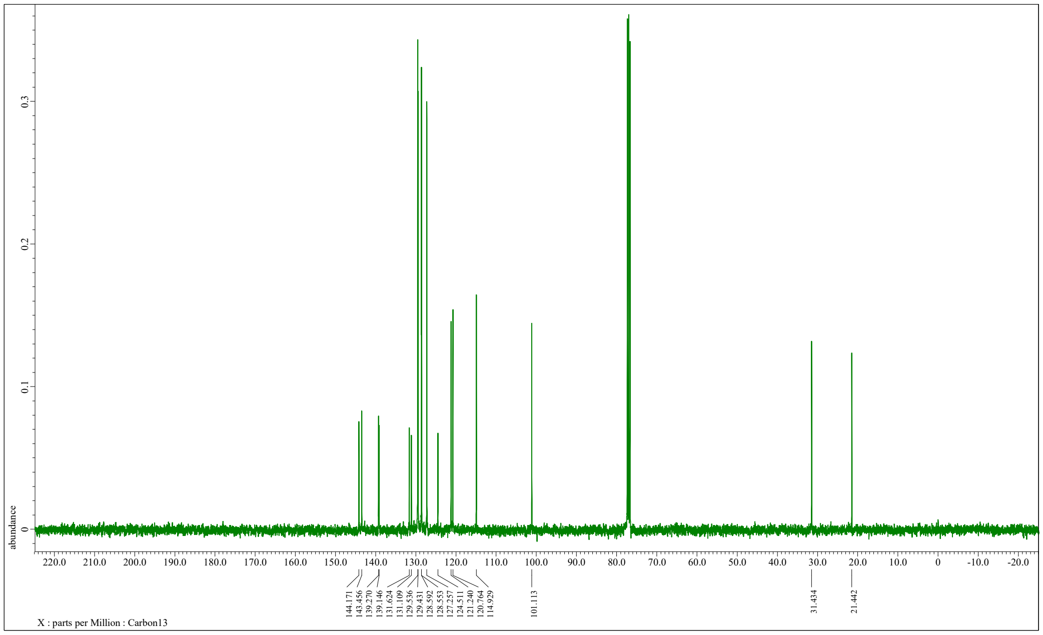

1-methyl-2-phenyl-3-tosyl-1*H*-indole (3r)

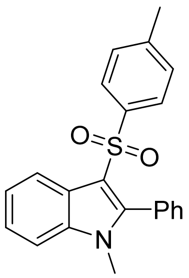

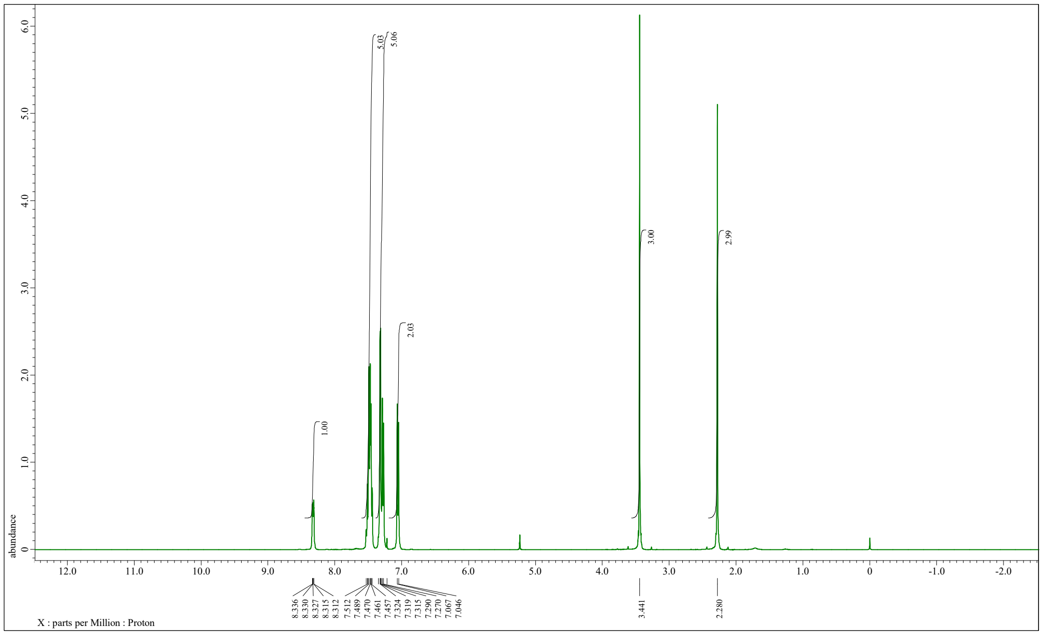

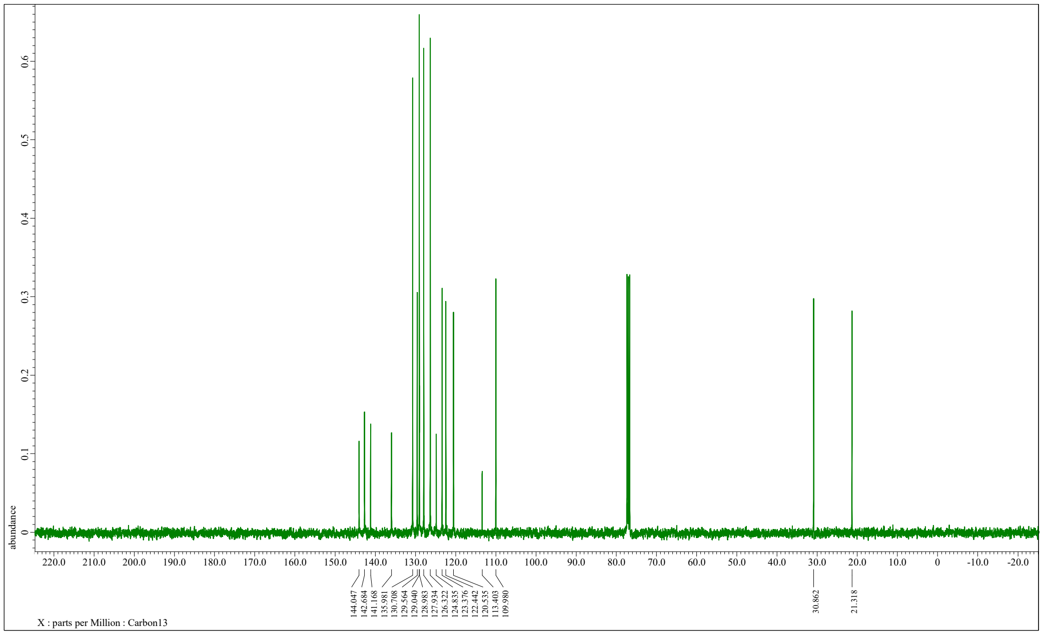

1-methyl-2-phenyl-6-tosyl-1*H*-indole (4r)

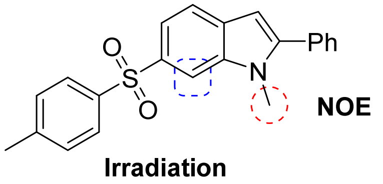

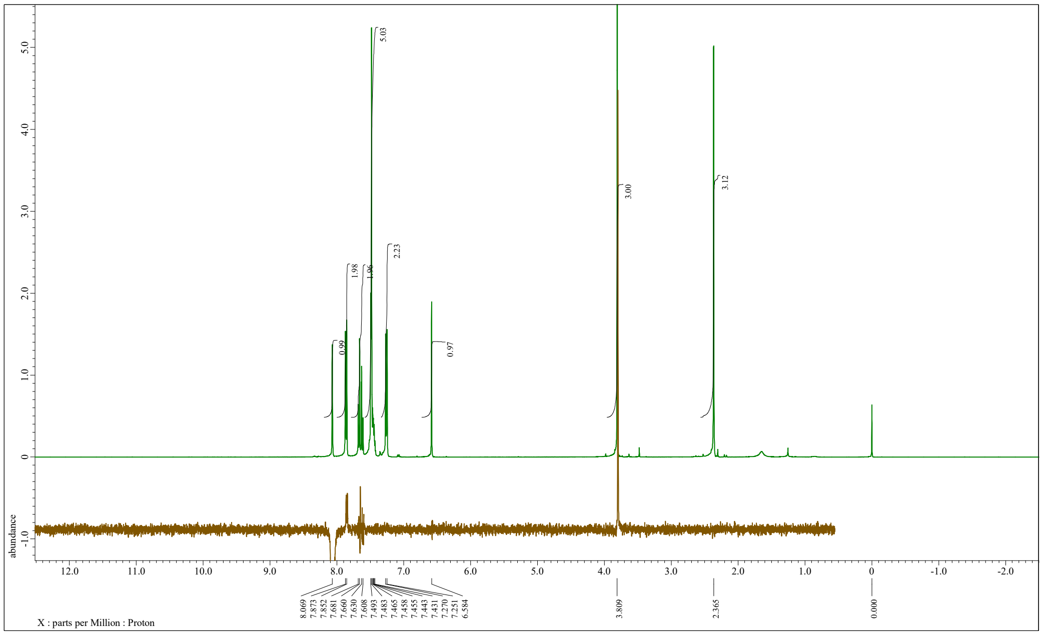

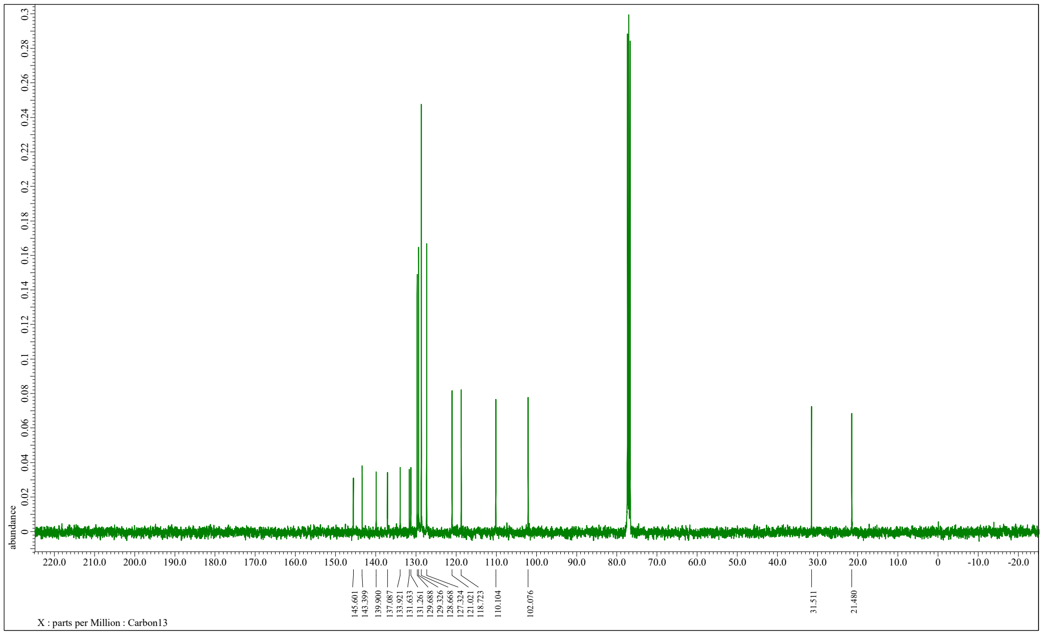

Characterization of 7-sulfonylindoles

1-methyl-2-propyl-7-tosyl-*1H*-indole (5a)

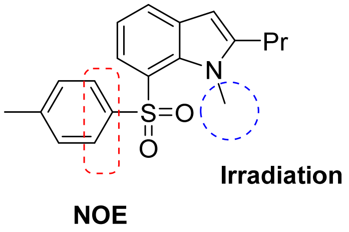

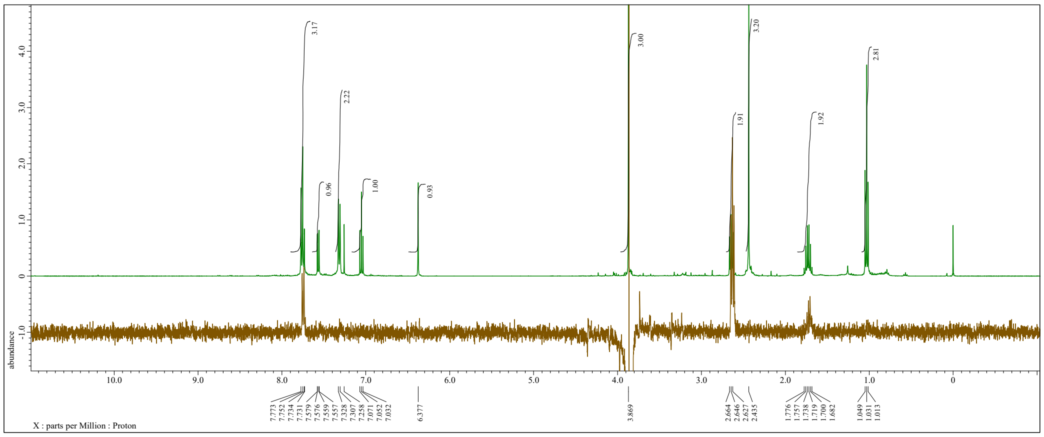

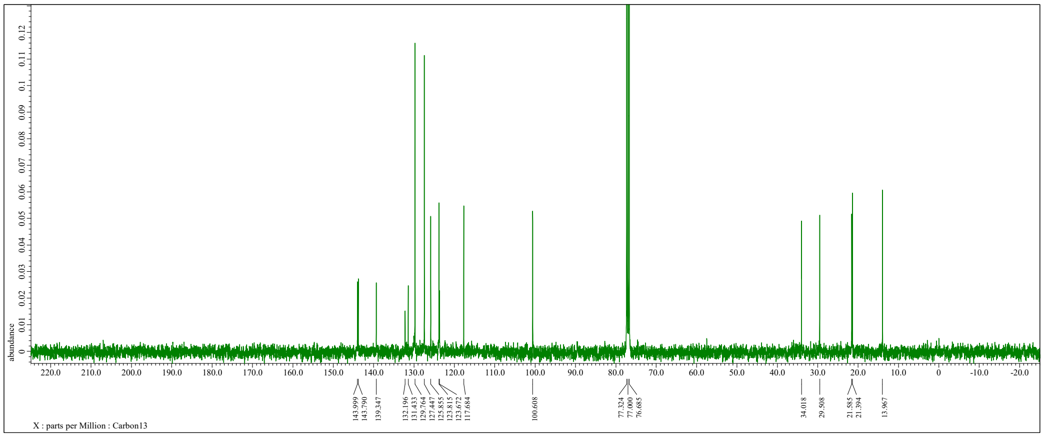

7-((4-fluorophenyl)sulfonyl)-1-methyl-2-propyl-*1H*-indole (5f)

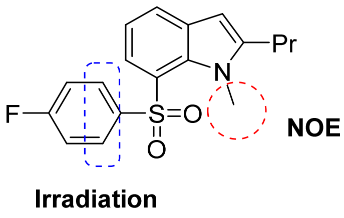

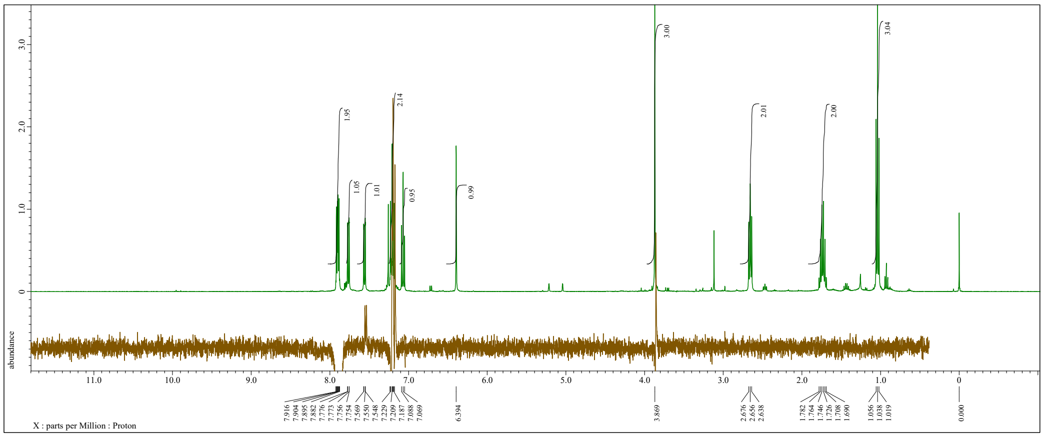

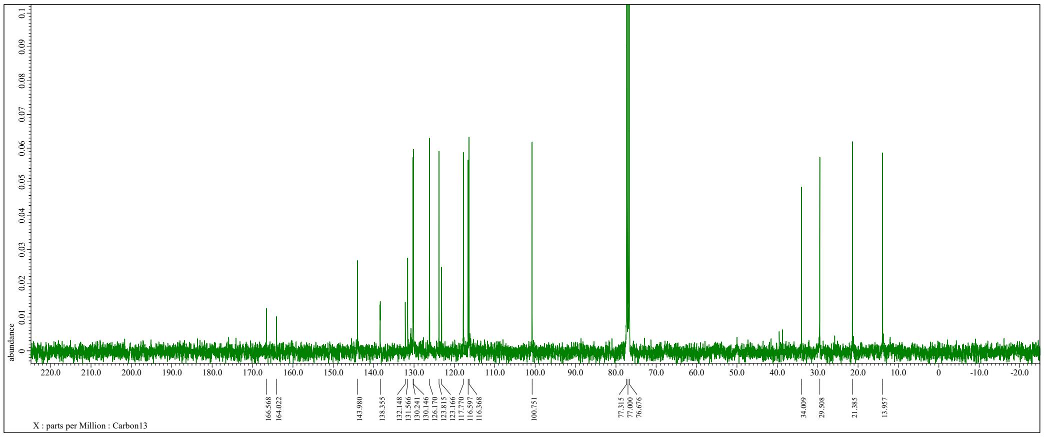

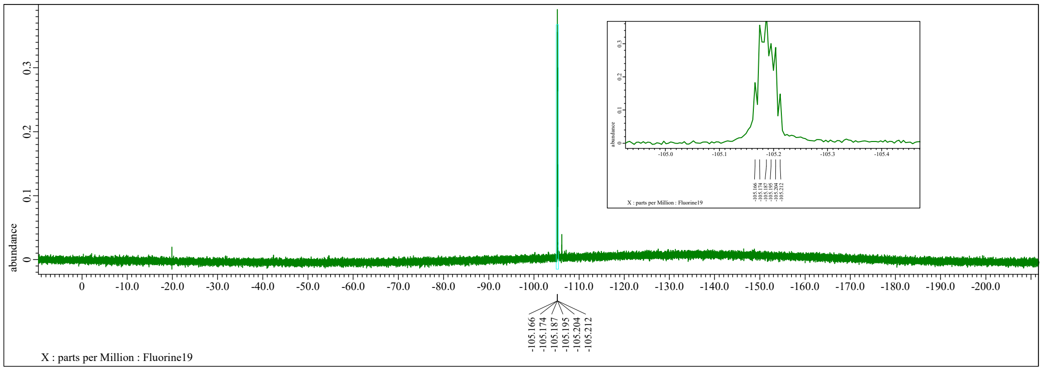

12. Determination of ^{1}H NMR yield

After designated reaction time, the reaction was quenched using 1 mL of 1N $Na_2S_2O_3$ solution, extracted with EtOAc, dried over Na_2SO_4 and passed through a short silica gel pad to remove catalyst. After the removal of solvent under vacuum, the residue was dissolved in 1 mL of $CDCl_3$ and 17.5 μL of CH_2Br_2 was quickly added to the mixture using a micro syringe. After thorough mixing, the sample is rapidly transferred to an NMR tube for ^{1}H-NMR measurements.

The yields of each product were calculated based on the integral area of indole-3-H (corresponding to 4-sulfonylindole **2** and 6-sulfonylindole **4**) or N-CH_3 (corresponding to 3-sulfonylindole **3** and recovered **1**).

1. Standarization of CH_2Br_2 integral area

0.25 mmol of **1a** and **1o** (which can be obtained in large crystalline form) was subject to above-mentioned process and using 17.5 μL of CH_2Br_2 as internal standard. Integral of N-CH_3 was standardized to 300, and the response of CH_2Br_2 was calculated based on the average value of integral area.

The integral area of CH_2Br_2 is 185.24 for **1a** and 183.04 for **1o**, as a result, the integral area for 17.5 μL CH_2Br_2 internal standard is determined to be 184.

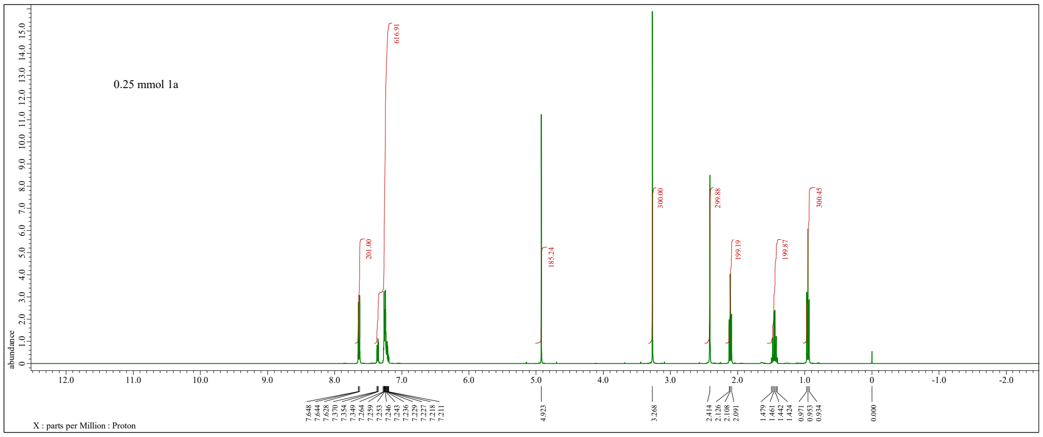

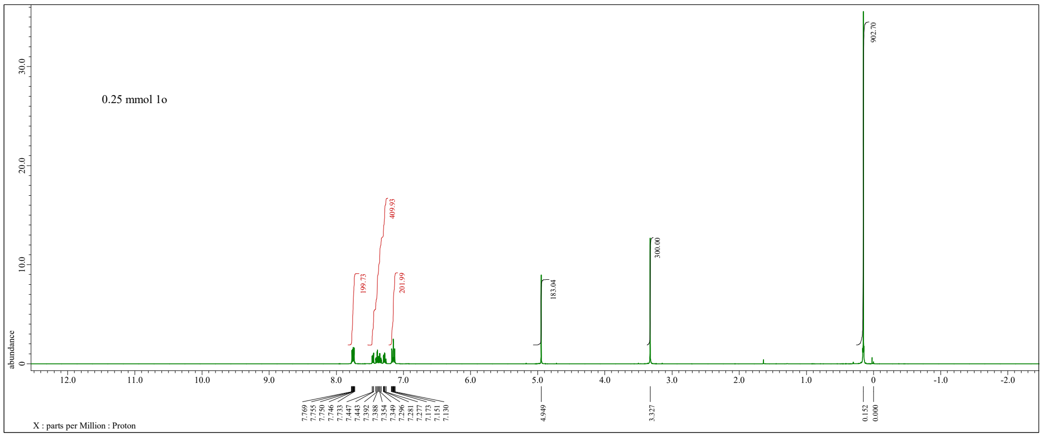

2. Determination of ^{1}H-NMR yield of each product

Yield was determined based on the integral area of on the integral area of indole-3-H (corresponding to 4-sulfonylindole **2** and 6-sulfonylindole **4**) or N-CH_3 (corresponding to 3-sulfonylindole **3** and recovered **1**). For product **2** and **4**, the integral area represents the NMR yield. For product **3** and recovered **1**, the integral area divided by 3 represents the NMR yield. For **1r**, which its NMR signal will overlap with CH_2Br_2, yield was determined by normalization of total N-CH_2-Ph area as 200.

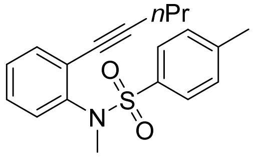

1a

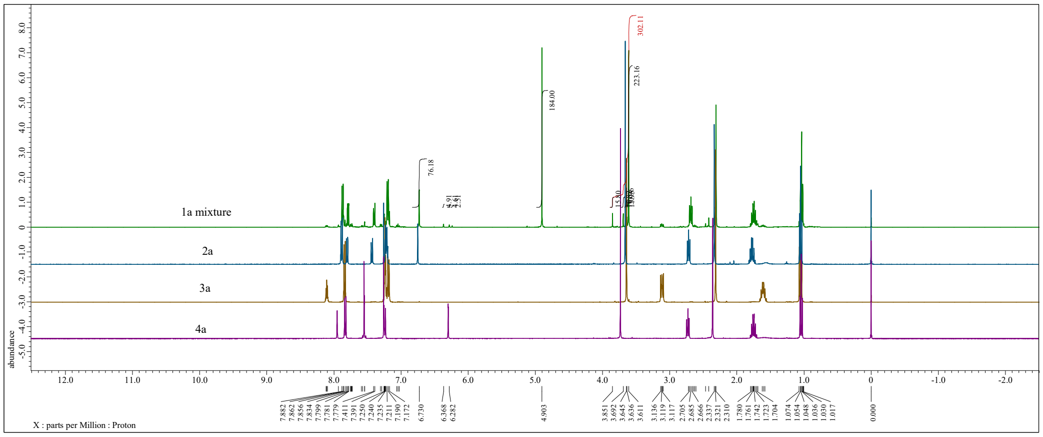

Product	Peak position (ppm)	Integral area (a.u.)	NMR yield (%)
**2a**	6.73 (1H)	76.18	76
**3a**	3.64 (3H)	30.96	10
**4a**	6.28 (1H)	4.61	5

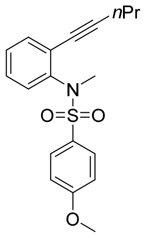

1b

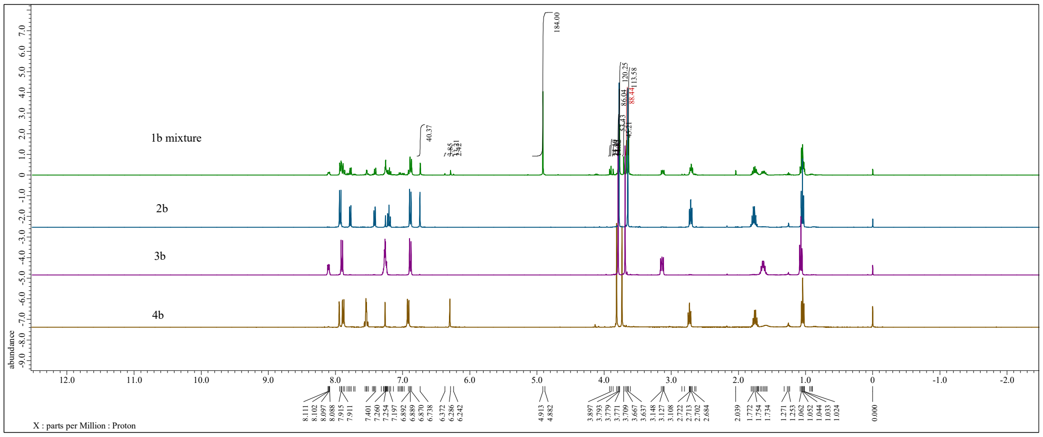

Product	Peak position (ppm)	Integral area (a.u.)	NMR yield (%)
**2b**	6.74 (1H)	40.37	40
**3b**	3.67 (3H)	88.44	29
**4b**	6.29 (1H)	13.21	13

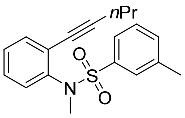

1c

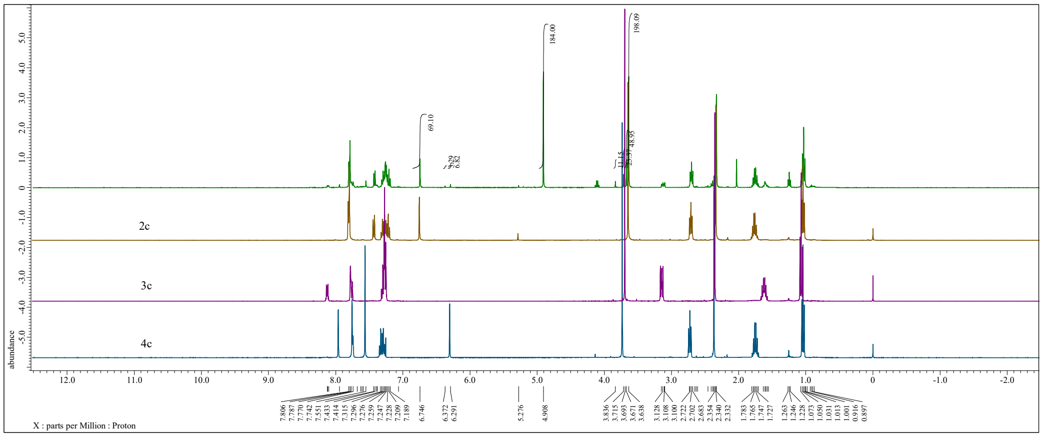

Product	Peak position (ppm)	Integral area (a.u.)	NMR yield (%)
**2c**	6.75 (1H)	69.10	69
**3c**	3.67 (3H)	48.95	16
**4c**	6.29 (1H)	6.82	7

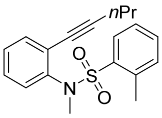

1d

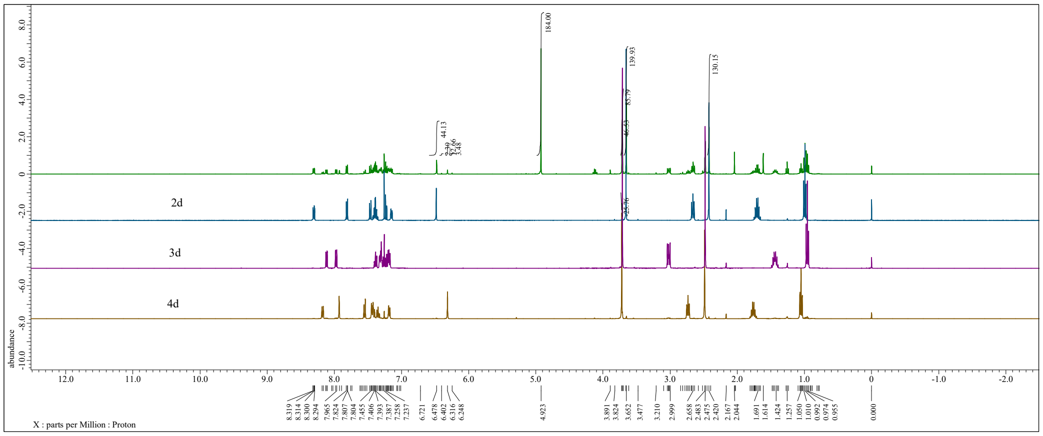

Product	Peak position (ppm)	Integral area (a.u.)	NMR yield (%)
**2d**	6.48 (1H)	44.13	44
**3d**	3.71 (3H)	85.71	29
**4d**	6.32 (1H)	12.66	13

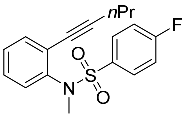

1e

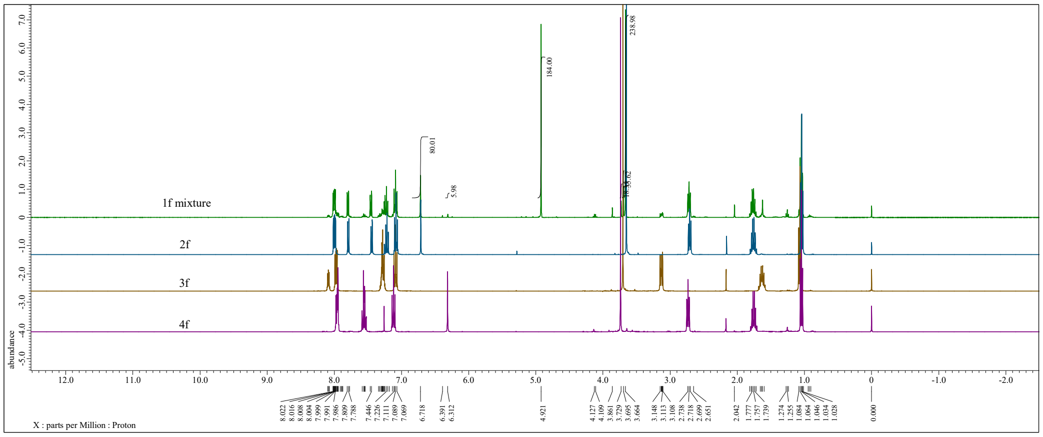

Product	Peak position (ppm)	Integral area (a.u.)	NMR yield (%)
**2e**	6.72 (1H)	80.01	80
**3e**	3.70 (3H)	35.62	12
**4e**	6.31 (1H)	5.98	6

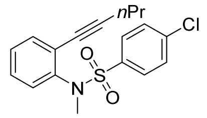

1f

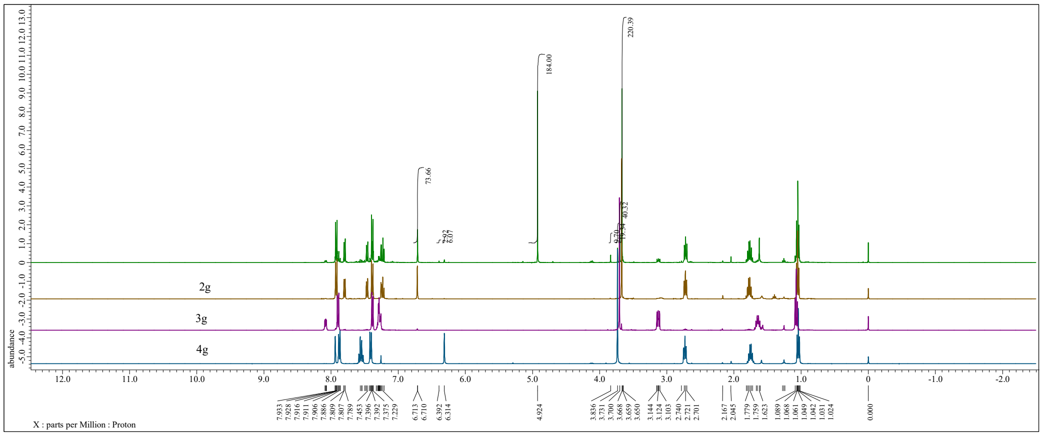

Product	Peak position (ppm)	Integral area (a.u.)	NMR yield (%)
**2f**	6.71 (1H)	73.66	74
**3f**	3.70 (3H)	40.32	13
**4f**	6.31 (1H)	6.07	6

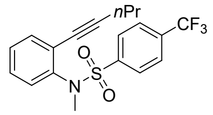

1g

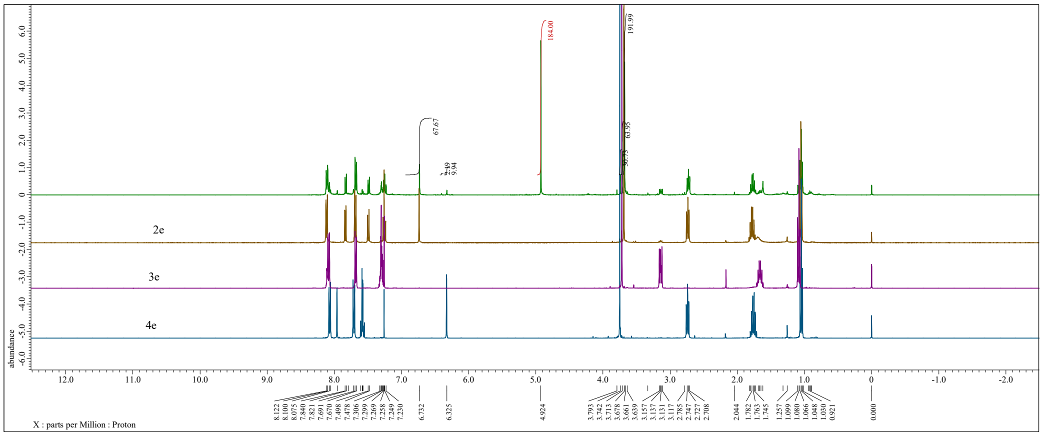

Product	Peak position (ppm)	Integral area (a.u.)	NMR yield (%)
**2g**	6.73 (1H)	67.67	68
**3g**	3.71 (3H)	63.95	21
**4g**	6.32 (1H)	9.94	10

1h

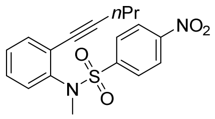

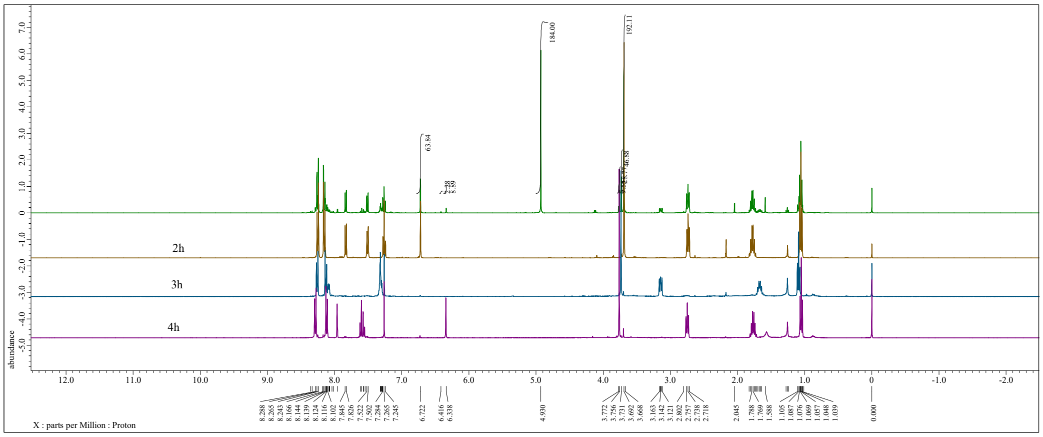

Product	Peak position (ppm)	Integral area (a.u.)	NMR yield (%)
**2h**	6.72 (1H)	63.84	64
**3h**	3.73 (3H)	46.88	16
**4h**	6.34 (1H)	8.89	9

1i

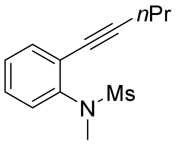

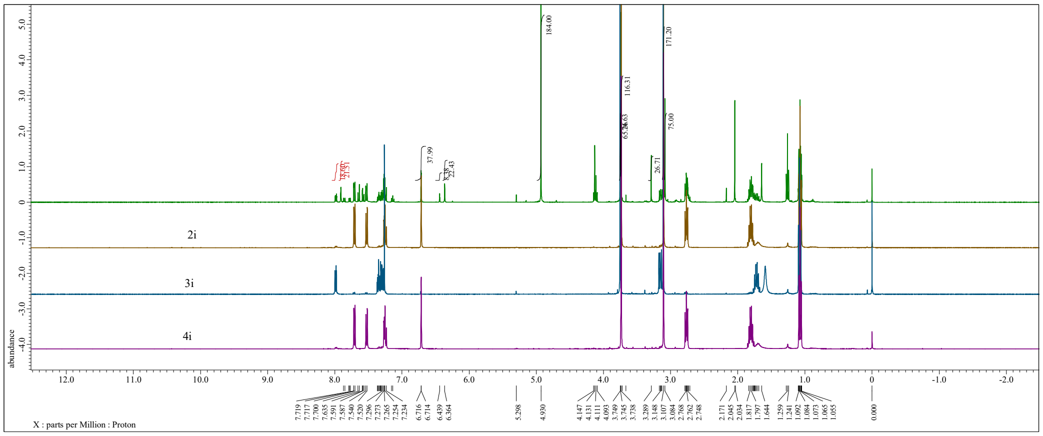

Product	Peak position (ppm)	Integral area (a.u.)	NMR yield (%)
**2i**	6.72 (1H)	37.99	38
**3i**	7.97-7.99 (1H)	18.60	19
**4i**	6.36 (1H)	22.43	22

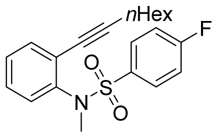

1j

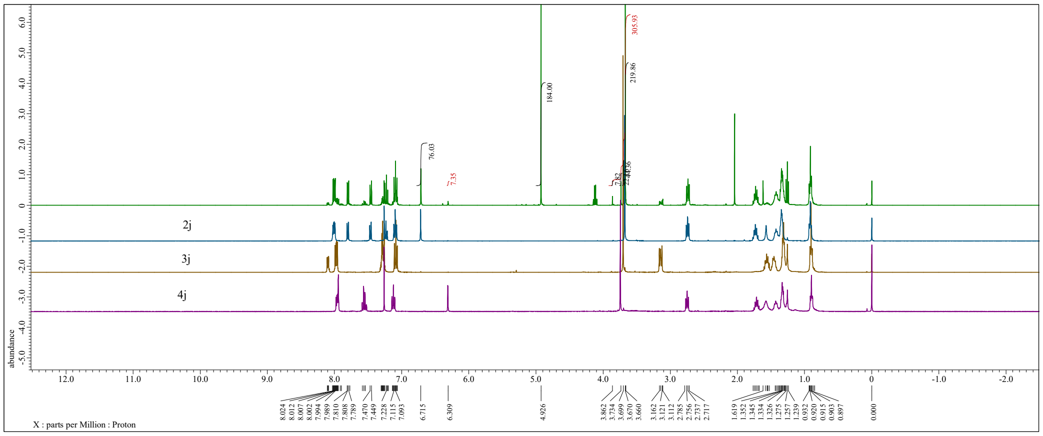

Product	Peak position (ppm)	Integral area (a.u.)	NMR yield (%)
**2j**	6.72 (1H)	76.03	76
**3j**	3.70 (3H)	44.36	15
**4j**	6.31 (1H)	7.35	7

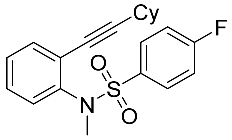

1k

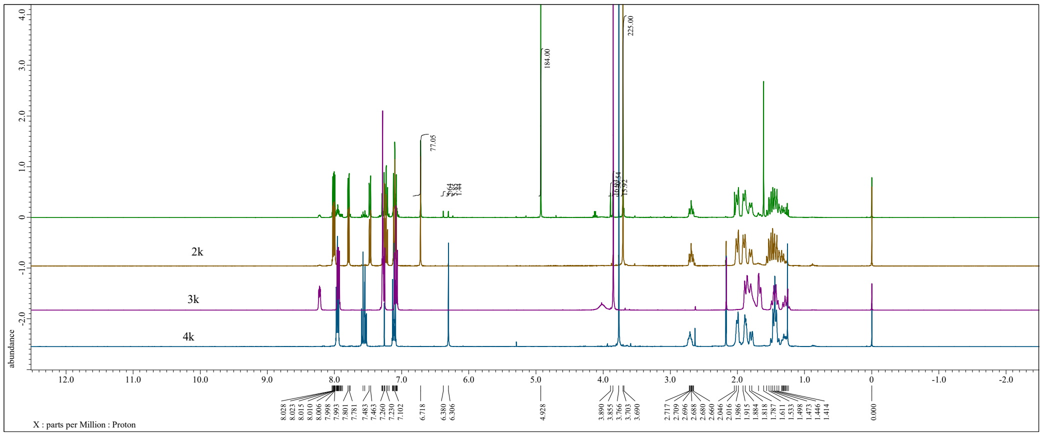

Product	Peak position (ppm)	Integral area (a.u.)	NMR yield (%)
**2k**	6.72 (1H)	77.05	77
**3k**	3.86 (3H)	30.54	10
**4k**	6.31 (1H)	5.85	6

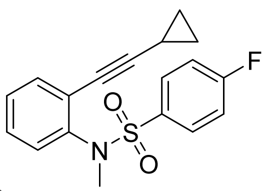

1l

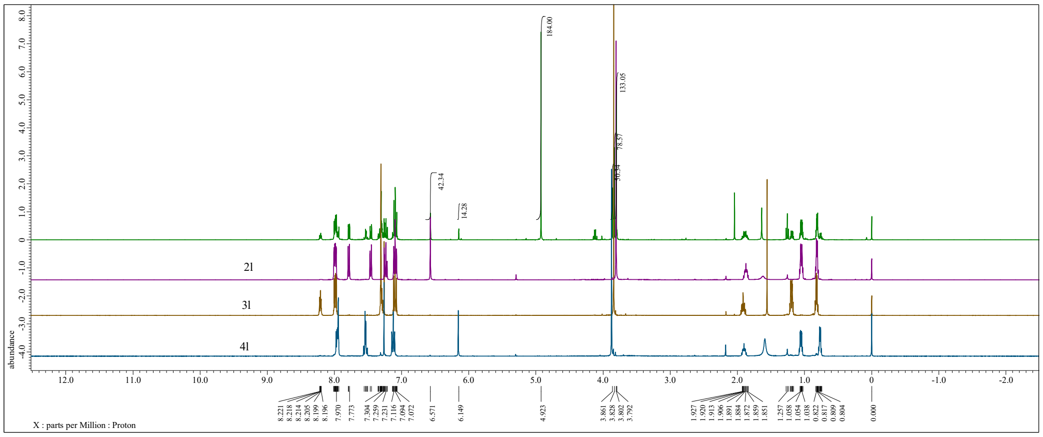

Product	Peak position (ppm)	Integral area (a.u.)	NMR yield (%)
**2l**	6.57 (1H)	42.34	42
**3l**	3.83 (3H)	78.57	26
**4l**	6.15 (1H)	14.28	14

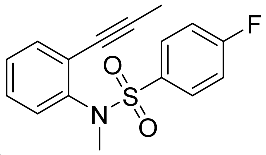

1m

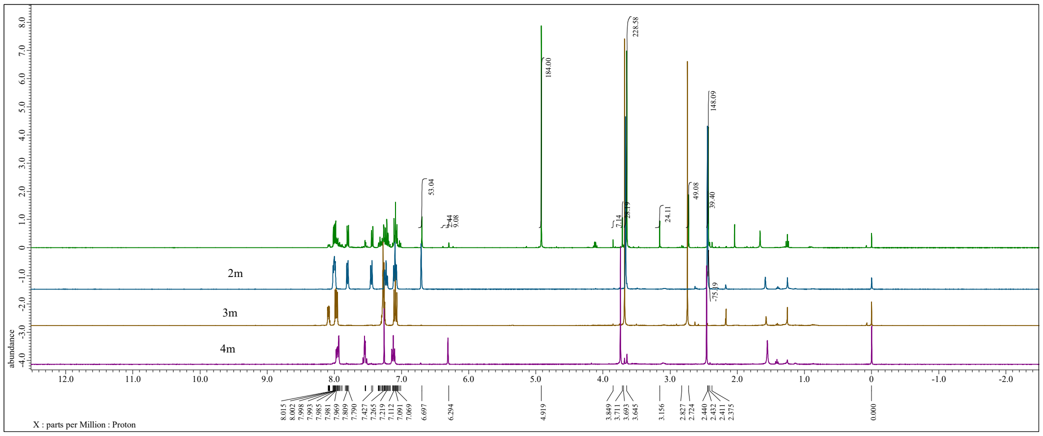

Product	Peak position (ppm)	Integral area (a.u.)	NMR yield (%)
**2m**	6.70 (1H)	53.04	53
**3m**	2.72 (3H)	49.08	16
**4m**	6.29 (1H)	9.08	9

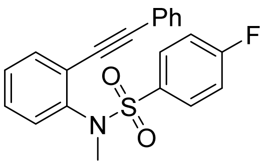

1n

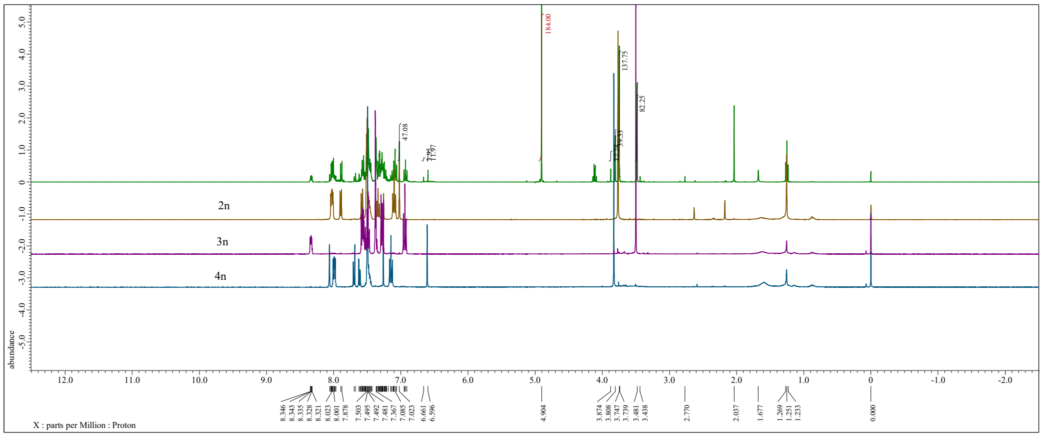

Product	Peak position (ppm)	Integral area (a.u.)	NMR yield (%)
**2n**	7.02 (1H)	47.08	47
**3n**	3.48 (3H)	82.25	27
**4n**	6.60 (1H)	11.97	12

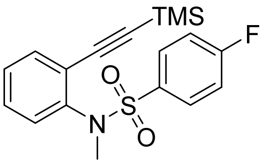

1o

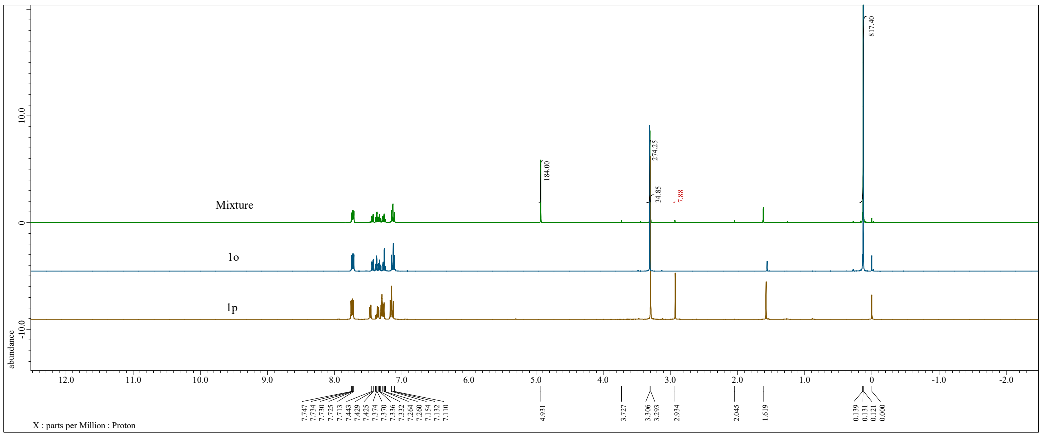

Product	Peak position (ppm)	Integral area (a.u.)	NMR yield (%)
**1o**	0.13 (9H)	817.40	91
**1p**	2.93 (1H)	7.88	8

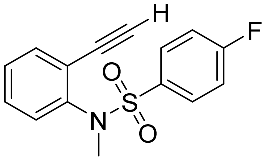

1p

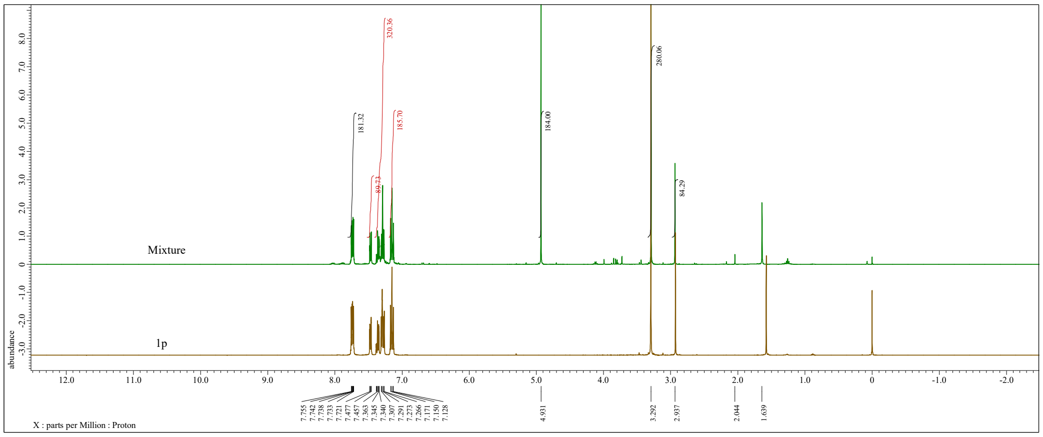

Product	Peak position (ppm)	Integral area (a.u.)	NMR yield (%)
**1p**	4.93 (3H)	280.06	93

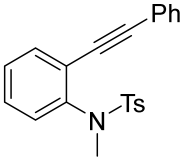

1q

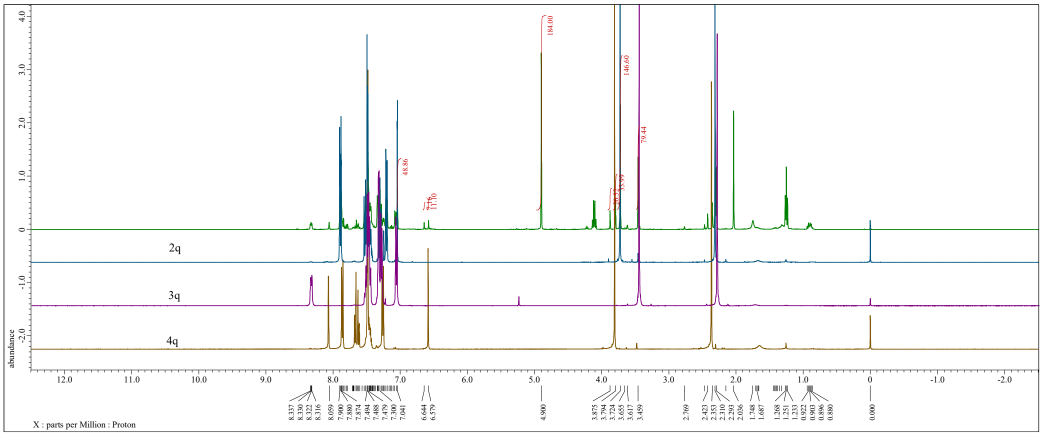

Product	Peak position (ppm)	Integral area (a.u.)	NMR yield (%)
**2q**	7.04 (1H)	48.81	49
**3q**	3.46 (3H)	79.44	26
**4q**	6.58 (1H)	11.10	11

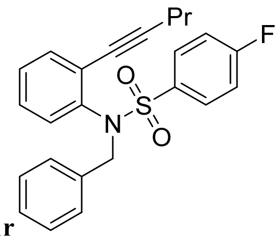

1r

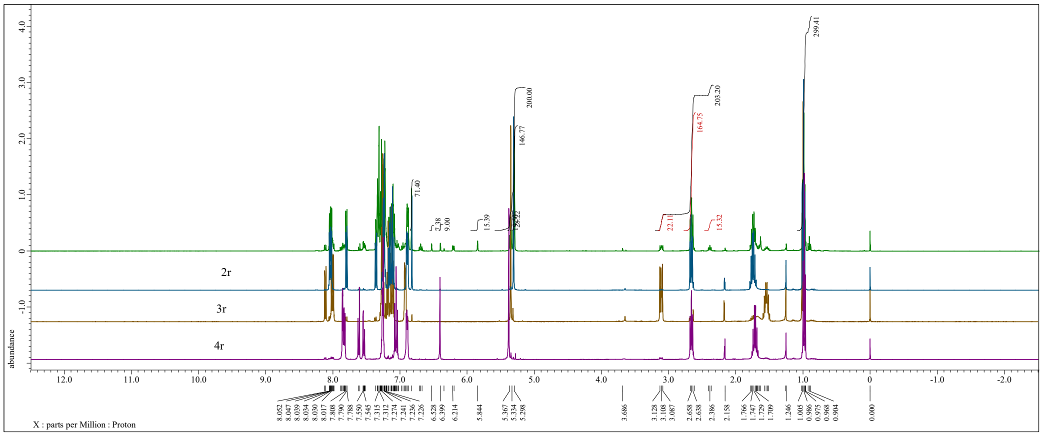

Product	Peak position (ppm)	Integral area (a.u.)	NMR yield (%)
**2r**	6.83 (1H)	71.40	71
**3r**	5.33 (2H)	28.22	14
**4r**	6.40 (1H)	9.00	9
